# Impact of Influenza A Virus Infection on Growth and Metabolism of Suspension MDCK Cells Using a Dynamic Model

**DOI:** 10.3390/metabo12030239

**Published:** 2022-03-12

**Authors:** João Rodrigues Correia Ramos, Thomas Bissinger, Yvonne Genzel, Udo Reichl

**Affiliations:** 1 Bioprocess Engineering, Max Planck Institute for Dynamics of Complex Technical Systems, Sandtorstrasse 1, 39106 Magdeburg, Germany; tbissinger86@gmail.com (T.B.); genzel@mpi-magdeburg.mpg.de (Y.G.); ureichl@mpi-magdeburg.mpg.de (U.R.); 2 Institute of Process Engineering, Faculty of Process & Systems Engineering, Otto-von-Guericke University, Universitätsplatz 2, 39106 Magdeburg, Germany

**Keywords:** dynamic model, metabolism, glycolysis, tricarboxylic acid cycle (TCA), metabolomics, influenza A virus, MDCK cells, virus replication, modeling

## Abstract

Cell cultured-based influenza virus production is a viable option for vaccine manufacturing. In order to achieve a high concentration of viable cells, is requirement to have not only optimal process conditions, but also an active metabolism capable of intracellular synthesis of viral components. Experimental metabolic data collected in such processes are complex and difficult to interpret, for which mathematical models are an appropriate way to simulate and analyze the complex and dynamic interaction between the virus and its host cell. A dynamic model with 35 states was developed in this study to describe growth, metabolism, and influenza A virus production in shake flask cultivations of suspension Madin-Darby Canine Kidney (MDCK) cells. It considers cell growth (concentration of viable cells, mean cell diameters, volume of viable cells), concentrations of key metabolites both at the intracellular and extracellular level and virus titers. Using one set of parameters, the model accurately simulates the dynamics of mock-infected cells and correctly predicts the overall dynamics of virus-infected cells for up to 60 h post infection (hpi). The model clearly suggests that most changes observed after infection are related to cessation of cell growth and the subsequent transition to apoptosis and cell death. However, predictions do not cover late phases of infection, particularly for the extracellular concentrations of glutamate and ammonium after about 12 hpi. Results obtained from additional in silico studies performed indicated that amino acid degradation by extracellular enzymes resulting from cell lysis during late infection stages may contribute to this observed discrepancy.

## 1. Introduction

Seasonal influenza epidemics and global pandemics can have a significant economic impact on societies and result in a very high death toll. It is only due to the availability of vaccines and antivirals that more severe consequences can be averted, as seen in the current COVID-19 outbreak. The majority of influenza vaccines are still produced in embryonated hens’ eggs. However, to overcome certain disadvantages of this production system and to meet rising demands, various cell culture-derived vaccine manufacturing processes have been established [1] and key aspects regarding the pros and cons of both production systems have been studied [2,3,4,5]. Madin-Darby canine kidney (MDCK) cells are one of the substrates used for production of influenza A virus (IAV) [6,7,8]. Typically, this continuous cell line is cultivated and infected with IAV near the end of the exponential cell growth phase with a low multiplicity of infection (moi). The virus replicates intracellularly after entering the cells, and the first virions are released approximately 4–6 h post infection (hpi). The virus yield in cell cultures is influenced by a variety of factors including the cell substrate used, the cell concentration at time of infection, moi, medium composition and pH value [1]. In comparison to other cell culture-based processes, most notably large-scale recombinant protein production with bioreactor harvests in the gram per liter range, typical virus yields are rather low. Due to the complexity of virus–host cell interaction, numerous explanations and hypotheses exist regarding these low cell-specific virus yields (CSVY). These include the interferon-mediated antiviral response, a high rate of cell death as a result of virus-induced apoptosis and rapid cell degradation [9,10,11,12,13,14,15,16,17] as well as numerous host cell factors [18,19]. Recently, it was demonstrated that by combining model-based analysis with experimental data collected on genetically engineered cells, it is possible to investigate the effect of selected host cell factors on individual virus replication steps and to predict measures to increase virus yields [20]. So far, the majority of studies performed to improve virus production processes focused on cell metabolism since the synthesis of viral components requires precursors and energy from the host cell. Quantitative changes in extracellular metabolite concentrations observed during the progression of infection for several viruses, including IAV, included changes in glucose consumption, lactate production and ammonium release, among other effects [21,22,23,24,25]. These changes have been primarily attributed to cell growth arrest, virus-induced apoptosis, breakdown of intracellular carbon and energy metabolism, and cell damage [21,25]. While some viruses appear to induce changes in aerobic glycolysis, many viruses also seem to stimulate fatty acid synthesis or influence amino acid metabolism, i.e., glutaminolysis—most likely to meet specific virus replication requirements [26]. Nevertheless, the cumulative effect of these changes on virus yields is still poorly understood. Additionally, it is largely unclear whether the metabolic changes observed during virus production in cell culture are caused directly by virus-specific mechanisms or are influenced indirectly by the transition of infected cells to apoptosis and cell lysis. Even for cultivations infected with low moi of IAV, changes are observed as early as 6–8 hpi. These changes include a rapid decrease in viable cell concentration and a decrease in the average cell-specific volume, as well as changes in substrate consumption, metabolic by-product release, and in cell death rate.

Quantitative studies on the impact of virus infections on cell growth and metabolism require comprehensive sets of experimental data, ideally collected for both infected and mock-infected cells. This includes viable cell counts, cell size and viability, extracellular substrates and metabolic by-products, and, ideally, intracellular metabolite concentrations and enzyme activity measurements. The establishment of dynamic mechanistic models is crucial for evaluating such complex and high-dimensional data [27]. Typically, these models are composed of a set of ordinary differential equations (ODEs) with defined initial conditions that make biologically plausible assumptions about cell growth, metabolism and infection kinetics [28,29]. In particular, the formulated mathematical relationships should establish a direct link between experimental data and cellular behavior [30,31,32]. This enables the identification of complex mechanisms underlying the metabolic network [33]. Additionally, such models can be used for simulation, prediction and optimization studies for process design and optimization [34]. In this instance, the developed models should enable a detailed analysis of cell growth and changes in the central carbon and energy metabolism of both mock-infected and infected cells. Based on this, a deeper understanding of the direct and indirect impact of virus replication on its host cells can be obtained. Furthermore, possible bottlenecks can be identified to take measures to increase cell-specific or overall virus yields. Despite their usefulness, such modeling approaches frequently face limited data availability and computational constraints [35,36,37] and should be combined with other omics measurements [38] and hybrid approaches that complement what is mechanistically known [39] in order to confirm identified hypotheses. Nevertheless, various complex models describing cellular metabolism have been implemented for *E. coli* and yeasts [40,41,42]. For animal cells, fewer dynamic large-scale models have been established that describe not only cell growth but also the central carbon and energy metabolism. Often, the latter are limited to either glycolysis [43,44,45] or tricarboxylic acid cycle (TCA) [46,47] and only few attempts have been made to incorporate aspects of virus replication. Regarding the latter, the majority of modeling approaches have focused on estimation of cellular resources required for virus production [48] or on metabolic flux analysis [49,50,51].

In this study, a dynamic mathematical model was established for IAV production in suspension MDCK cells, which combines a segregated cell growth model with a structured model of intracellular metabolism. The model structure is based on a previous approach established for a human designer cell line (AGE1.HN) [52], and takes into account additional aspects related to IAV propagation. Only few aspects of the intracellular metabolic network relating to lactate and ammonium accumulation as well as some enzyme kinetics were modified. The model is composed of 35 ODEs that account for cell growth (concentration of viable cells, mean cell diameter, volume of viable cells), virus production (virus titer) and concentrations of key metabolites both at the intracellular and extracellular level. The majority of model parameters were estimated using experimental data from a mock-infected culture. Using the identified set of parameters and specific initial conditions for each experiment, model simulations accurately captured the overall dynamics of the mock-infected culture and largely predicted the dynamics of the cultivation where cells were infected. For the first 24 hpi, IAV infection seemed to have a negligible effect on the intracellular metabolism, with the majority of changes in metabolic rates occurring as a direct result of cell growth arrest, virus-induced apoptosis, cell damage and cell lysis. A few notable exceptions were the dynamics of glutamate and ammonium release at later infection time points (12 hpi). In essence, findings based on model simulations indicate that IAV infection has only a minor impact on central carbon metabolism and energy metabolism of suspension MDCK cells. The majority of metabolic changes seem to be directly related to cessation of cell growth and the subsequent transition to apoptosis and cell death. Additional in silico studies were conducted to investigate reasons for the discrepancy between experimental data and model simulations for glutamate and ammonium during late infection phases.

## 2. Results and Discussion

The model used was based on a dynamic model for suspension AGE1.HN cells used for recombinant protein production established by Ramos et al. [52]. It combines a segregated cell growth model with a structured model of the central carbon metabolism taking into consideration the viable cell concentration, mean cell diameter, viable cell volume, concentration of extracellular substrates and intracellular concentrations of key metabolites from the central carbon and energy metabolism. This model describes the dynamic of several metabolites, which requires the kinetic descriptions of various enzymes, increasing its biological relevance and also its overall complexity. The robustness of this modeling approach is demonstrated by using the same set of parameters to describe stirred tank bioreactors batch cultivations at different scales. Additionally, the model allowed detailed in silico studies to link intracellular rates to physiological states of the cell. In this study, this modeling approach was transferred to describe the growth of suspension MDCK cells used in IAV vaccine production and extended to assess the impact of IAV replication on cellular metabolism. As expected, changing the cell line, the cultivation medium and the operation mode from stirred tank to shake flask required specific changes in the previously established model. Furthermore, virus production (virus particle accumulation in the supernatant) and virus-induced cell death had to be considered. In addition, with the introduction of new states in the structured part of the model relating to central carbon and energy metabolism, various reactions/transporter kinetics had to be modified. For a detailed description of the extended model, please refer to the model definition section in the supplement (Supplementary File S1).

### 2.1. Simulation of Cell Growth and Virus Production

**Figure 1:**
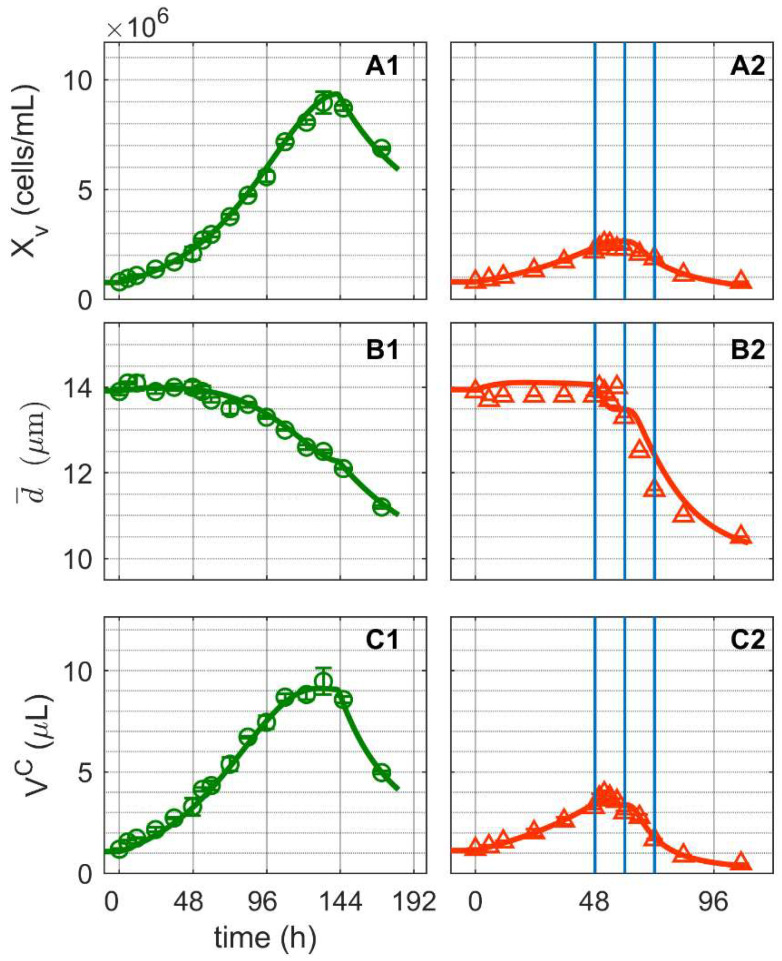
Dynamics of cell growth in mock-infected and infected suspension MDCK cells. (**A1**,**A2**) Viable cell concentration, (**B1**,**B2**) mean cell diameter and (**C1**,**C2**) total volume of viable cells. Data and error bars represent the mean and standard deviation of technical triplicates for two independent experiments (mock-infected 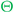 and infected 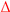). Lines: model simulations. Vertical blue lines correspond to 0, 12 and 24 h post infection. Experimental data used for parameter estimation: **A1**, **B1**, **C1** (see Supplementary Files S4 and S5).

After inoculation with 7.6 × 10^5^ cells/mL, cell concentrations increased exponentially in both shake flask cultivations (Figure 1), reaching a maximum cell growth rate (µ_max_) of 0.0025 h^−1^ (Figure 1(A1,A2)). The first cultivation (Cultivation 1, mock-infected) reached a maximum cell concentration of 9.47 × 10^6^ cells/mL at around 130 h before cells began to die due to substrate depletion. The second cultivation (Cultivation 2, infected) reached a cell concentration of 2.1 × 10^6^ cells/mL at around 48 h and was infected with IAV at moi = 10 (infectivity based on TCID_50_ assay). As soon as 3 hpi, cell concentrations, mean diameter of cells and consequently viable cell volumes started to decrease. In comparison, the mean cell diameters of Cultivation 1 decreased only gradually from a maximum of 14 µm (around 22 h post inoculation) to 11 µm (end of cultivation, Figure 1B). Similar findings for changes in the mean cell diameter of mock-infected cultures have been reported for adherent MDCK cells [45,53,54] and other suspension cell lines [52], though to a lesser degree. Overall, model simulations effectively reproduced the dynamics of cell concentrations, mean cell diameters and viable cell volumes for both mock-infected and infected cells. Accordingly, it can be safely assumed that the segregated model and the structured model were linked with good accuracy. The model simulations for both cultivations cover changes in the mean cell diameter of about 20%, resulting in up to 50% variation in the mean cell-specific volume ($V_{s}^{c}$, Equation (9) in Supplementary File S1) and up to a 40% variation in volumetric enzyme activities (Equation (2) in Supplementary File S1). These changes are consistent with previous findings for lower volumetric enzyme activities during exponential cell growth compared to later cultivation phases for adherent MDCK cells [45,55] and other suspension cell lines [56].

**Figure 2:**
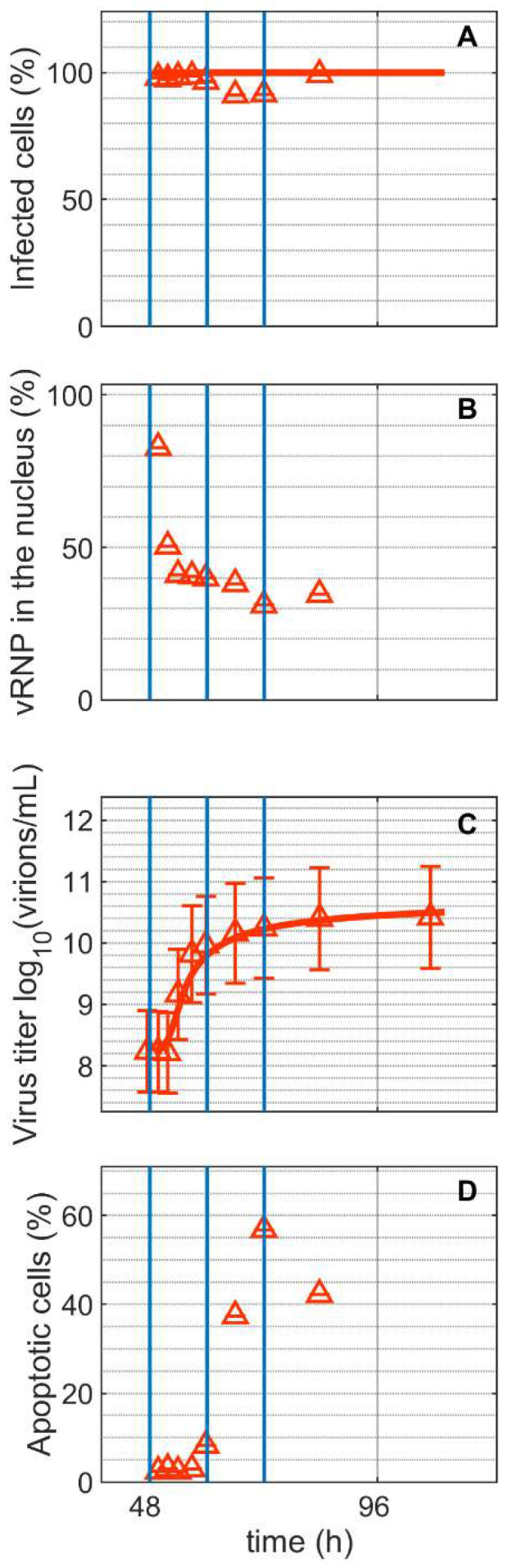
Dynamics of influenza A virus replication in suspension MDCK cells after synchronous infection at 48 h post inoculation. (**A**) Percentage of infected cells, (**B**) percentage of viral ribonucleoproteins (vRNP) in the cell nucleus, (**C**) virus titer, and (**D**) percentage of apoptotic cells. Vertical blue lines correspond to 0, 12 and 24 h post infection, respectively. Data and error bars represent the mean and standard deviation of technical triplicates for one experiment (infected 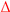).

Staining with a monoclonal antibody directed against the IAV nucleoprotein (NP) demonstrated that all cells in Cultivation 2 were infected concurrently, as expected for an infection with moi = 10 (Figure 2A). Viral ribonucleoproteins (vRNP) accumulated strongly in the cell nucleus after infection (Figure 2B). Shortly after (1.8 hpi), the percentage of vRNP dropped to about 31% (Figure 2B), indicating the export of viral genomes to the cytoplasm for budding and virus release. In the supernatant of Cultivation 2, first virions could be quantified by the HA assay at around 6 hpi and they reached a maximum of 10.24 log_10_(virions/mL) at 24 hpi (Figure 2C). The percentage of apoptotic cells started to increase at around 12 hpi (Figure 2D). Model simulations accurately describe the increase in number of infected cells and increase in the total number of virions.

### 2.2. Simulation of Substrate and Metabolic By-Product Dynamics

**Figure 3:**
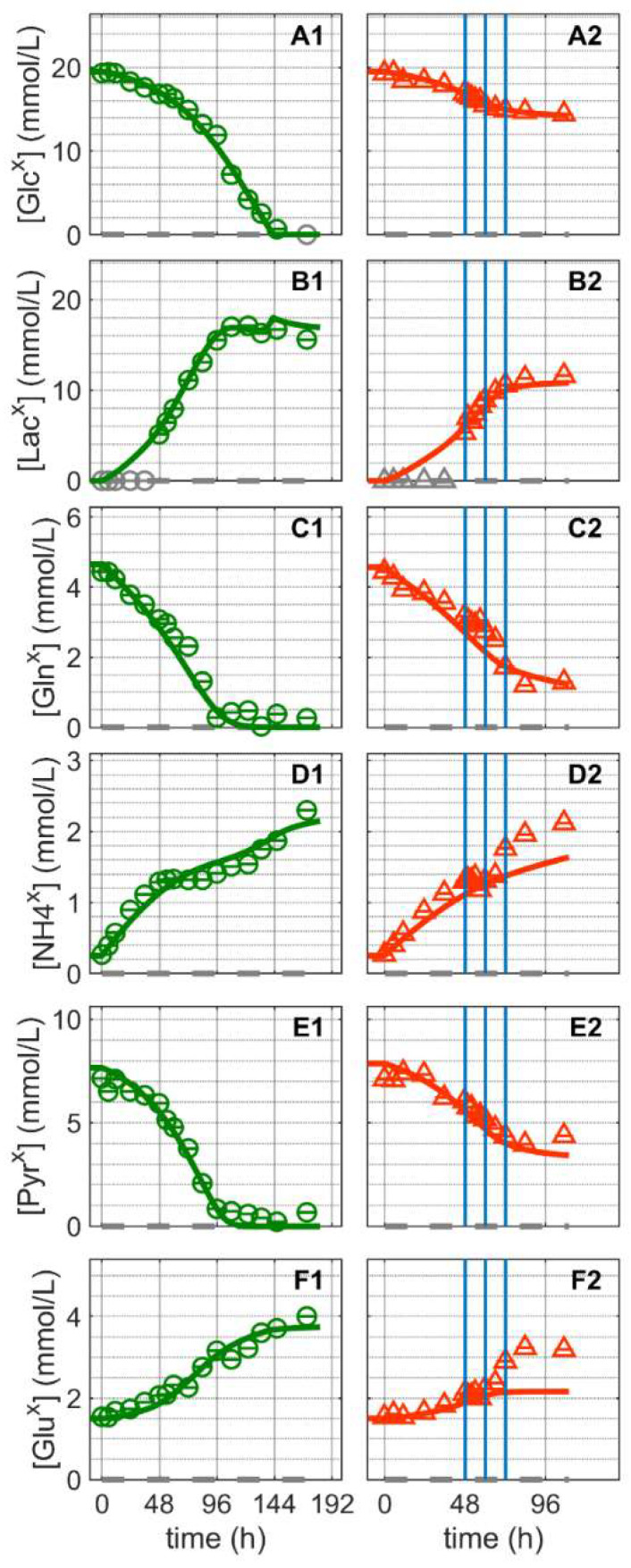
Dynamics of extracellular substrates and metabolic by-products in mock-infected and infected suspension MDCK cells. (**A1**,**A2**) Glucose, (**B1**,**B2**) lactate, (**C1**,**C2**) glutamine, (**D1**,**D2**) ammonium, (**E1**,**E2**) pyruvate, and (**F1**,**F2**) glutamate. Data and error bars represent the mean and standard deviation of technical triplicates for two independent experiments (mock-infected 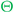 and infected 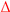). Lines: model simulations. Vertical blue lines correspond to 0, 12 and 24 h post infection, respectively. The grey dashed lines indicate the limit of quantification for each metabolite and grey data points are under the limit of quantification. Experimental data used for parameter estimation: **A1**, **B1**, **C1**, **D1**, **E1** and **F1** (see Supplementary Files S4 and S5).

The substrates and metabolic products considered in this study were glucose, lactate, glutamine, ammonium pyruvate and glutamate (Figure 3). Extracellular glucose (Figure 3A) was rapidly consumed until depletion approximately 144 h after Cultivation 1 was inoculated (Figure 3(A1)). Glutamine and pyruvate were consumed even faster and depleted at around 100 h (Figure 3(C1,E1)). Similar to other suspension cell lines, glucose depletion occurred concomitantly with the onset of the cell death phase of the mock-infected cells (Figure 1(A1)), confirming its critical role as a key substrate. For Cultivation 2, which was infected at around 48 h post inoculation, the cells initially consumed glucose at a similar rate as for Cultivation 1 (mock-infected), but consumption ceased as virus replication progressed, which subsequently led to cell death (Figure 1(A2) and Figure 3(A2). Notably, model simulations using the same set of parameters for both cultures allowed a good description of the dynamics of glucose, pyruvate and glutamine not only during the first 24 h post inoculation, but also during later phases.

The concentration of extracellular lactate (Figure 3B) increased in the bioreactor until glucose was depleted. As previously reported for MDCK cells [57,58], the stoichiometric ratio of lactate to glucose was approximately 1:1 for both cultivations. Typically, in continuous cell lines, the majority of glucose is converted to pyruvate via glycolytic enzymes, which in turn is converted to lactate to regenerate NAD^+^ to maintain a high ATP generation rate [59,60]. The previous model used lumped reaction to describe extracellular lactate production directly from intracellular pyruvate [52]. However, in this study, to better describe the extracellular lactate dynamics, lactate metabolism had to be considered in greater detail. In particular, it was assumed that intracellular lactate is produced via lactate dehydrogenase (LDH) in a reversible reaction, and an equation was added (Equation (61) in Supplementary File S1) to connect intracellular lactate to its extracellular form. LDH is a highly regulated enzyme with a very fast turnover. Additionally, depending on the metabolic state of the cell, it can favor either lactate production or lactate consumption. Lactate metabolism is complex and different theories exist regarding the control of lactate production and consumption [61,62,63,64,65,66,67,68]. Here, a reversible hill kinetic with two modifiers (Equation (61) in Supplementary File S1) was used and was sufficient to account for the inherent complexity. Furthermore, for the uptake of lactate, a reversible hill equation was used (Equation (45) in Supplementary File S1), which considers a minor lactate consumption after glucose depletion (Figure 3(B1) and $r_{Lac_{trans}^{x}}$ in Figure S4). As a result, model simulations accurately reproduced the lactate dynamics in mock-infected and infected cells. Note that the accumulation of lactate in the bioreactor supernatant was estimated directly from the intracellular rates.

Mock-infected cells consumed glutamine and pyruvate from the extracellular environment until their depletion at around 100–110 h (Figure 3(C1,E1)). However, both metabolites were not depleted when the cells were infected at 48 h post inoculation since cell growth was halted and cells started to die soon after infection (Figure 1(A2) and Figure 3(C2,E2)). Similar to the previous model [52], their transport into the intracellular environment was not considered to be growth-related, as both substrates were depleted before the exponential cell growth phase ended (Figure 1(A1) and Figure 3(C1,E1)). Their consumption rates take into account different kinetics as they might be governed by homeostasis or involve other mechanisms unrelated to cell growth [69]. More specifically, their mechanism of transport was described using Michaelis–Menten kinetics or a direct binding equation [70] (Equations (43) and (46) in Supplementary File S1). Overall, the model accurately describes the dynamics in the concentration of these metabolites in the mock-infected cell culture and accurately predicts their dynamics in infected cells.

Ammonium and glutamate accumulated until the end of Cultivation 1, even after the depletion of glutamine, their primary source (Figure 3(D1,F1)). Glutamate is a non-essential amino acid that is synthesized from glutamine and other amino acids, e.g., via proline and lysine catabolism [71]. In contrast, the production of ammonium is closely linked to the metabolism of various amino acids. Apart from glutamine and glutamate, no additional amino acids were quantified in this study, but we have previously shown that the majority of amino acids are not depleted at the end of the exponential growth phase of suspension MDCK cell cultivations [72]. Therefore, and as reported previously for other suspension cell lines [52,73], an intracellular accumulation of glutamate and ammonium appears to occur even during late stages of cultivation, followed by their release into the supernatant. Accordingly, model simulations are also in good agreement with the experimental data in the mock-infected cells cultivation (Figure 3(D1,F1)). For Cultivation 2, however, significant discrepancies between model predictions and experimental data are observed (Figure 3(D2,F2)). Starting about 12 hpi, ammonium and glutamate concentrations are clearly underestimated. Assuming that assumptions in the model about glutamate and ammonium metabolism are justified, it must be concluded that virus infection either results in drastic changes in cellular metabolism during the late phase of IAV infection or there are other sources in which virus-induced cell death and cell lysis play a significant role. In particular, either enzymes released into the extracellular environment following cell lysis retain a high level of activity or both metabolites leak into the supernatant due to cell lysis. The latter, however, can be safely excluded as in silico model simulations clearly demonstrated that the complete release of intracellular glutamate and ammonium would not result in more than a 1.5% increase in their extracellular concentrations (an increase of about 0.025 mmol/L for glutamate and 0.009 mmol/L for ammonium, respectively; see Section 2.1 in Supplementary File S6). On the other hand, it cannot be ruled out that enzymes released into the extracellular environment because of cell lysis retain a significant activity. According to results obtained from in silico model simulations, taking the amino acid degradation/conversion rate based on viable cell volume (microscale) and converting it to the bioreactor volume scale (macroscale) would be enough to explain the increase in extracellular ammonium and glutamate concentrations (see Section 2.2 in Supplementary File S6). Similar mechanisms might also apply for other metabolites including lactate and pyruvate. However, as these events take place in a time window where most virus particles have been released into the supernatant and the number of productive cells is declining rapidly, these findings are more or less irrelevant for process optimization.

### 2.3. Simulation of Intracellular Metabolism

#### 2.3.1. Glycolysis, Pentose Phosphate Cycle and Uridine Diphosphate Sugar Metabolism

**Figure 4:**
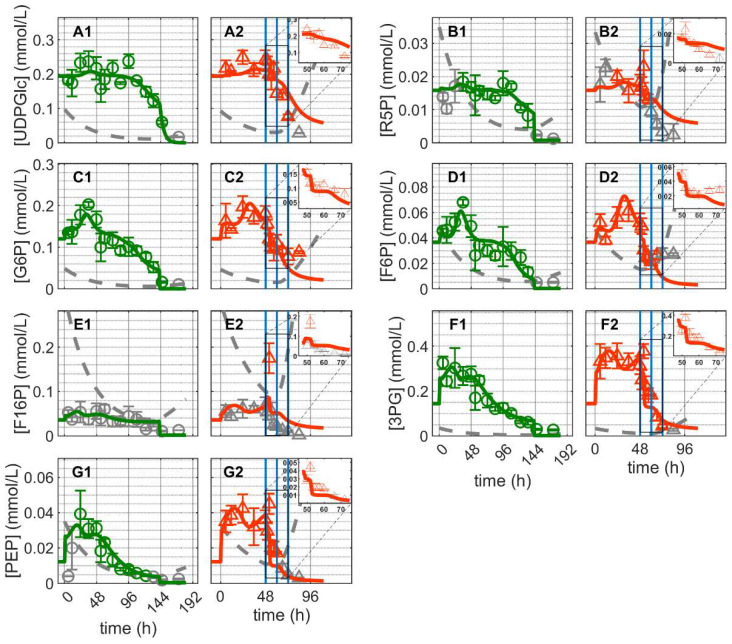
Dynamics of metabolites from glycolysis and pentose phosphate pathway in mock-infected and infected suspension MDCK cells (insert: 48–72 h of infected cultivation). (**A1**,**A2**) Uridine diphosphate glucose, (**B1**,**B2**) ribose–5-phosphate, (**C1**,**C2**) glucose-6-phosphate, (**D1**,**D2**) fructose-6-phosphate, (**E1**,**E2**) fructose-1,6-biphosphate, (**F1**,**F2**) 3-phosphoglutarate and (**G1**,**G2**) phosphoenolpyruvate. Data and error bars represent the mean and standard deviation of technical triplicates for two independent experiments (mock-infected 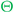 and infected 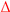). Lines: model simulations. Vertical blue lines correspond to (0, 12 and 24 h post infection, respectively). The grey lines indicate the limit of quantification for each metabolite and the grey data points are under the limit of quantification. Experimental data used for parameter estimation: **A1**, **B1**, **C1**, **D1**, **E1**, **F1** and **G1** (see Supplementary Files S4 and S5).

Similar dynamics were observed for the majority of glycolytic metabolites: a short, more or less peak-like initial accumulation of metabolites followed by a gradual decrease over the cultivation time for both cultivations (Figure 4). Similar dynamics occurred for the pentose phosphate pathway and uridine diphosphate metabolites, with relatively stable concentrations initially followed by rapid depletion over time (Figure 4). Prior to depletion, glucose-6-phosphate (G6P) and fructose-6-phosphate (F6P) exhibited comparable dynamics; the same was true for 3-phosphoglutarate (3GP) and phosphoenolpyruvate (PEP). On the other hand, the dynamics of fructose-1,6-biphosphate (F16P), ribose-5-phosphate (R5P) and uridine diphosphate glucose (UDPGlc) appeared to be not related to those of any other metabolite. In Cultivation 1, glycolytic metabolites were depleted at around 144 h concomitant with glucose depletion from the medium (Figure 3(A1) and Figure 4). In Cultivation 2, the concentrations of these metabolites decreased to low levels shortly after virus infection. Model simulations of glycolytic metabolite concentrations closely capture their dynamics in mock-infected cells. This indicates that reasonable assumptions about glycolysis and pentose phosphate shunt reactions kinetics were made, particularly regarding the enzymes involved in feedback control, namely hexokinase (HK), phosphofructokinase (PFK) and LDH [74,75,76]. Model predictions of glycolytic metabolite concentrations in infected cells are also very much in agreement with the experimental data, especially for the first 24 hpi (before onset of cell lysis and degradation). However, the model slightly underestimated the concentrations of G6P and F6P, though their concentrations started to drop below the limit of detection around 12 hpi. Additionally, the model did not predict the peak-like dynamic of F16P after virus infection. The latter may be considered an outlier since it is a single measurement. Given the precision with which the dynamics of the majority of metabolites were described, model assumptions regarding the kinetics and control of glycolysis appear to be sufficiently justified. These results, in combination with the fact that the same set of parameters was used for infected and non-infected cells, suggest that virus infection had a relatively minor impact on glycolysis, especially during the first 24 hpi when virus-induced apoptosis and cell lysis are negligible. This is consistent with the fact that the total virus particle volume (of 12,000 virions/cell in this instance) accounts for around 0.55% of the volume of a single cell. Even considering that some of the viral components (protein, RNA) synthesized in infected cells are not used for progeny virus production, the overall burden of virus replication on cellular metabolism can be considered low. This implies that in theory a single cell can produce many more virions. Note that the limit of quantification refers to the intracellular concentration and not to the concentration in the sample. As the sample volume was constant, the limit of quantification is inversely proportional to the viable cell volume per milliliter; increasing the number of cells per sample reduces the concentration of intracellular metabolites required to reach the limit of quantification and vice versa.

#### 2.3.2. TCA Cycle

**Figure 5:**
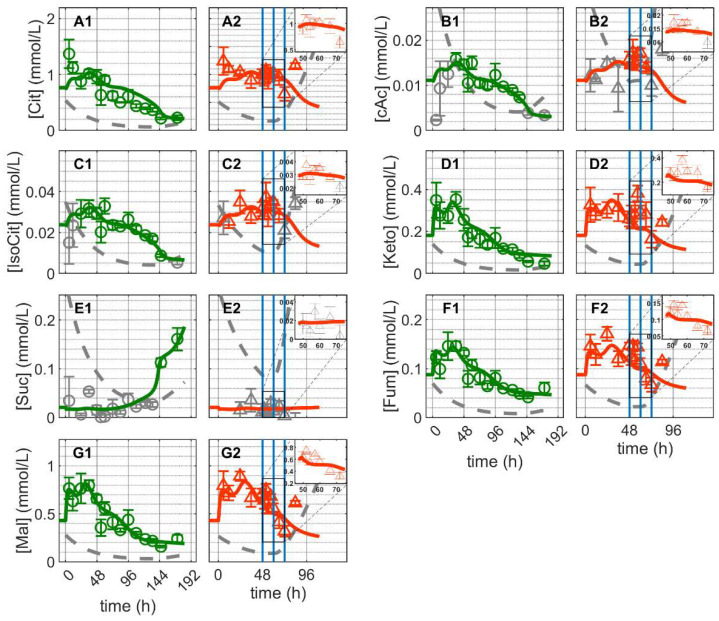
Dynamics of metabolites from the citric acid cycle in mock-infected and infected suspension MDCK cells (insert: 48–72 h of infected cultivation). (**A1**,**A2**) Citrate, (**B1**,**B2**) cis-aconitate, (**C1**,**C2**) iso-citrate, (**D1**,**D2**) alpha-ketoglutarate, (**E1**,**E2**) succinate, (**F1**,**F2**) fumarate and (**G1**,**G2**) malate. Data and error bars represent the mean and standard deviation of technical triplicates for two independent experiments (mock-infected 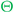 and infected 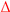). Lines: model simulations. Vertical blue lines correspond to (0, 12 and 24 h post infection, respectively). The grey lines indicate the limit of quantification for each metabolite and the grey data points are under the limit of quantification. Experimental data used for parameter estimation: **A1**, **B1**, **C1**, **D1**, **E1**, **F1** and **G1** (see Supplementary Files S4 and S5).

As for glycolytic metabolites, the concentration of the majority of TCA cycle metabolites showed an initial peak-like behavior and then decreased until about 144 h, when they remained practically constant until the end of Cultivation 1 (Figure 5). The exception was succinate (Suc), which did not exhibit the initial peak-like accumulation and its concentration remained almost constant initially (Figure 5(E1)). The concentration of citrate (Cit) remained approximately 100-fold that of cis-aconitate (cAc) and iso-citrate suggesting that the enzyme aconitate (ACO) favored citrate production (Figure 5(A1,B1,C1)). Other TCA cycle intermediates, such as alpha-ketoglutarate (Keto), fumarate (Fum) and malate (Mal) showed similar dynamics with concentrations exceeding the detection limit (Figure 5(D1,F1,G1)). The model simulation captures the dynamics of these metabolites in Cultivation 1 reasonably well. This implies that reasonable assumptions were made about the reactions kinetics of the TCA cycle, glutaminolysis and transamination. This occurred in particular, for example, by accounting for the inhibitory effect of oxaloacetate on succinate dehydrogenase [77,78], where the model simulation was able to capture the increase in Suc concentration near the end of the cultivation.

For Cultivation 2 (infected approximately 48 h post inoculation), concentrations of Cit, cAc and iso-citrate decreased immediately after infection and increased again around 24 hpi (Figure 5(A2,B2,C2)). Contrary to mock-infected cells, the concentration of Suc remained below the detection limit of after infection. Additionally, the concentrations of Keto, Fum and Mal increased rapidly immediately after infection (on average about 21%), decreased between 12–24 hpi and subsequently increased again with the onset of cell lysis and degradation (Figure 5(D2,F2,G2)). Using the same set of parameters estimated for mock-infected cells, the model predicts the dynamics of these metabolites reasonably well for about 24 hpi. The discrepancies between model predictions and the peak-like increase in Keto, Fum and Mal immediately after infection are difficult to interpret. Metabolic changes at early infection stages might be related to virus-induced cessation of cell growth [21], and/or early virus protein production [26], which in turn can lead to changes in the control of enzymes [25,26,79,80,81,82,83]. In this case, the noticeable accumulation of some metabolites of TCA cycle is either a side effect of virus infection or due to specific changes in related enzymes induced by early infection events. However, in any scenario, because of the characteristics of the metabolic network established (in particular its structural robustness and small-world property considering only a low number of reactions linking intracellular metabolites) [84], the dynamics of the reactions involved would allow a fast transition towards its inherent “normal behavior” where model assumptions are valid again. Nonetheless, the observed differences between model prediction and experimental data are generally small in the first 24 hpi, implying that virus replication had only minor impact on intracellular metabolite concentrations of the TCA cycle and closely related metabolic pathways. Discrepancies beginning at around 24 hpi are most likely due to virus-induced apoptosis, which results in the disintegration of mitochondrial membranes and cell lysis. In addition to this, the discrepancies due to the increase in the concentrations of certain metabolites of the TCA cycle starting about 24 h post infection may indicate a partial shutdown of the central carbon and energy metabolism. In addition, limitations concerning certain model assumptions and enzyme kinetics cannot be completely ruled out.

#### 2.3.3. Energy Metabolism

**Figure 6:**
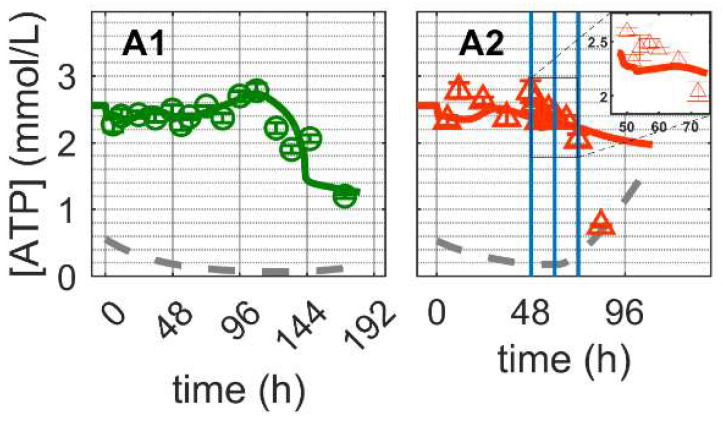
Dynamics of ATP in mock-infected and infected suspension MDCK cells. (**A1**,**A2**) Adenosine tri-phosphate (insert: 48–72 h of infected cultivation). Data and error bars represent the mean and standard deviation of technical triplicates for two independent experiments (mock-infected 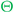 and infected 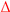). Lines: model simulations. Vertical blue lines correspond to (0, 12 and 24 h post infection, respectively). The grey lines indicate the limit of quantification for each metabolite and the grey data points are under the limit of quantification. Experimental data used for parameter estimation: **A1** (see Supplementary Files S4 and S5).

As expected, the ATP concentration (Figure 6) in mock-infected cells (Cultivation 1) remained high throughout the exponential cell growth phase (Figure 6(A1)) and decreased shortly after glutamine and pyruvate depletion (Figure 3(C1,E1)), at approximately 100 h post inoculation. The model simulation accurately reproduces the dynamics of ATP, implying that a good balance between consumption and production was achieved (Equations (38), (84) and (88) in Supplementary File S1). ATP is generated in glycolysis, TCA cycle, oxidative phosphorylation and other related metabolic pathways (Equations (82)–(90) in Supplementary File S1). Given the enymatic reactions for ATP production previously discussed, according to model simulations, glycolysis ($r_{glycolysis}$, Equation (82) in Supplementary File S1) accounts for approximately 20% of the total ATP production ($r_{glycolysis}$ in Figure S5), while glucose is present in the medium (until about 144 h). This is well within the range of 1–64% reported previously for several animal cell lines [85]. Additionally, the estimated theoretical oxygen consumption ranged from 62–113 fmol/cell/h ($r_{o2}$ in Figure S5), which is comparable to the consumption of other continuous cell lines with 7–97 fmol/cell/h [86,87].

For Cultivation 2, ATP concentrations remained high, even for the first 24 hpi, but subsequently decreased fast approaching the limit of quantification. Model simulations using the same set of parameters estimated for mock-infected cells resulted in a reasonable prediction of ATP concentration dynamics during the first 24 hpi. As specific mechanisms for the shutdown of metabolic pathways associated with apoptosis and cell deterioration have not been implemented (only cell death has been considered so far), the model significantly overestimates the concentration of ATP at later time points. In fact, the sharp decrease in ATP concentration starting at 24 hpi also supports the hypothesis of at least a partial metabolic shutdown, as ATP production via TCA cycle ($r_{TCA}$, Equation (83) in Supplementary File S1) accounted for approximately 80% of the estimated ATP production ($r_{TCA}$, Figure S5). Note that the amount of ATP produced from the TCA is obtained as described in Equations (38), (84) and (88) in Supplementary File S1. Generally, as for glycolysis and TCA cycle, model assumptions for ATP generation and consumption seem to be sufficiently justified. Furthermore, model parameters estimated for mock-infected cells enabled a good prediction of the dynamics in IAV infected cells as long as the shutdown of intracellular pathways does not play a significant role, i.e., for the first 12–24 h after virus entry, onset of intracellular virus replication and virus release.

Overall, the previously discussed changes in extracellular and intracellular metabolite dynamics seem to be predictable not only for mock-infected but also for infected cells. Reasonable predictions of the dynamics of key metabolites of both non-infected and infected cells for the period relevant for IAV replication and release were achieved. In particular, taking into account that a single set of parameters estimated for mock-infected cells was used and only kinetics for cell growth arrest and cell death after virus infection were implemented. This strongly suggests that the description of metabolic changes in IAV-infected cells primarily requires a reasonable description of cell growth arrest and transition to cell death, rather than specific enzymes kinetics. The fact that simulations of the metabolism dynamics of infected cells do not require a new set of parameters (compared to mock-infected cells), and suggests that IAV-specific mechanisms affecting the host cell’s central metabolic pathways do not play a significant role. This is further supported by the fact that the total volume of IAV virus particles released per cell is negligible (0.55%) and the theoretical energy cost of IAV replication is negligible (1%) in comparison to the host cell’s synthesis capacity [88]. Furthermore, previous studies also showed only minor differences in the specific oxygen uptake of mock-infected and infected cells during early stages of virus replication [89]. However, the effects of infection may be cell line- and/or virus-specific as contradictory findings have been reported for other virus–host cell systems, for instance in baculovirus production using insect cells [25].

#### 2.3.4. Analysis of Intracellular Rates

**Figure 7:**
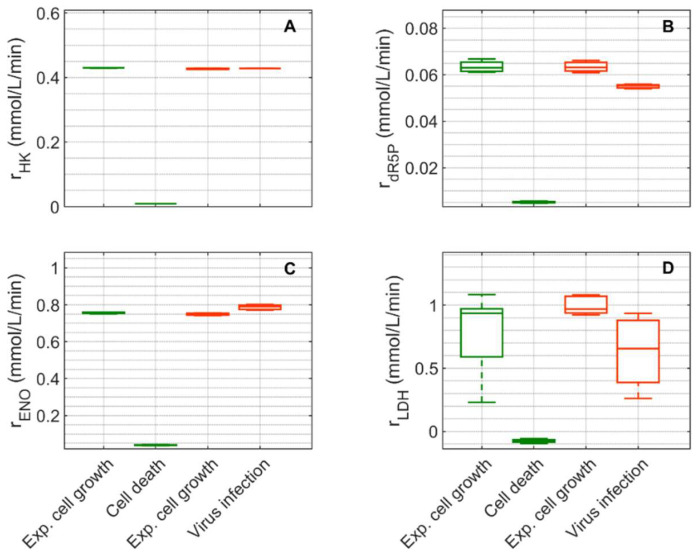
Box-and-whisker plot for selected intracellular rates of glycolysis and pentose phosphate pathway estimated from model simulations for mock-infected and infected suspension MDCK cells. (**A**) Hexokinase, (**B**) ribose-5-phosphate, (**C**) enolase, and (**D**) lactate dehydrogenase. Calculated from model simulations of the exponential growth phase of Cultivation 1 (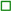, 6–108 h), the death phase of Cultivation 1 (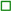, 146–169 h), the exponential growth phase of Cultivation 2 (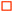, 6–48 h) and the virus replication phase of Cultivation 2 (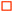, 49.9–107 h). The bar represents the median, the box is the first and third quartile, and the whisker the minimum and maximum of the rates from the model simulations of the corresponding cultivation phase.

**Figure 8:**
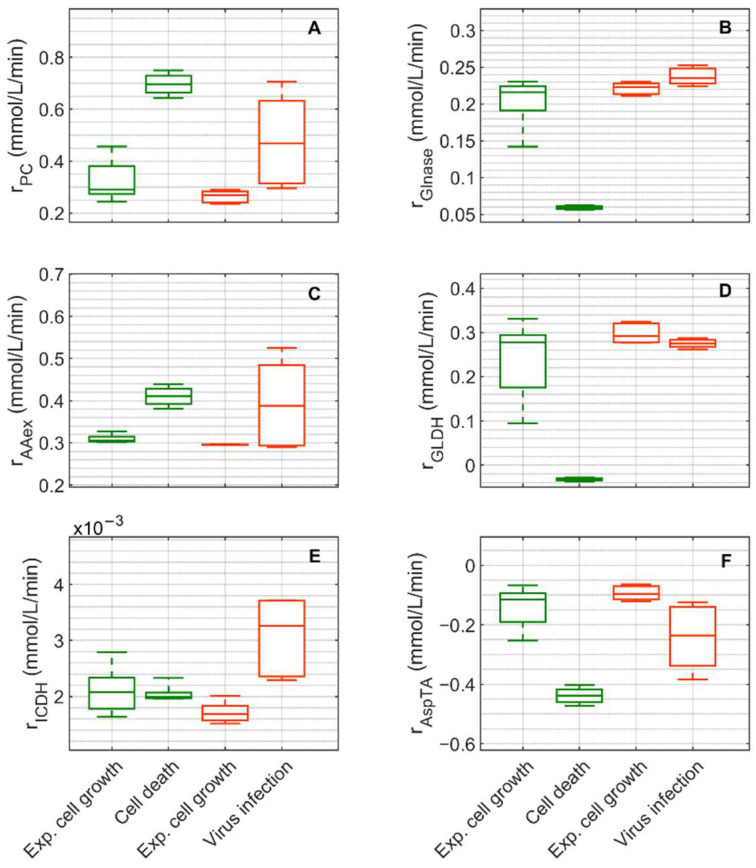
Box-and-whisker plot for selected intracellular rates of citric acid cycle, glutaminolysis and transamination estimated from model simulations for mock-infected and infected suspension MDCK cells. (**A**) pyruvate carboxylase, (**B**) glutaminase, (**C**) amino acid degradation, (**D**) glutamate dehydrogenase, (**E**) isocitrate dehydrogenase and (**F**) aspartate transaminase. Calculated from model simulations of the exponential growth phase of Cultivation 1 (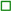, 6–108 h), the death phase of Cultivation 1 (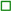, 146–169 h), the exponential growth phase of Cultivation 2 (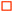, 6–48 h) and the virus replication phase of Cultivation 2 (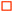, 49.9–107 h). The bar represents the median, the box is the first and third quartile, and the whisker the minimum and maximum of the rates from the model simulations of the corresponding cultivation phase.

In the following, a brief description of metabolism based on model simulations is provided for glycolysis and pentose phosphate pathway (Figure 7), and for TCA cycle, glutaminolysis and transamination (Figure 8).

Extracellular glucose (Figure 3A) was transported into the intracellular environment and rapidly converted to G6P through the hexokinase enzyme (HK) during the exponential cell growth phase in both cultivations (Figure 7A). After glucose was depleted in Cultivation 1 (mock-infected cells) at approximately 144 h (Figure 3(A1)) and the cell entered death phase (Figure 1(A1)), the estimated HK rate decreased to zero. Cells of Cultivation 2 (infected at around 48 h) still consumed glucose (Figure 3(A2)) at a similar rate after infection (Figure 7A). Approximately 14–16% of the intracellular glucose (after conversion by HK, percentage of $r_{dR5P}$ divided by the HK rate, Figure 7B) was further processed to R5P via glucose-6-phophate dehydrogenase or via transaldolase and transketolase during the exponential cell growth phase of both cultivations (6–108 h mock-infected, 6–48 h infected). This is well within the previously reported range of 0–40% for glucose conversion to R5P [90,91,92]. In the established model, the usage of R5P in unspecified reactions is taken into account using a general consumption rate ($r_{dR5P}$, Equation (56) in Supplementary File S1), and its rate is zero after glucose depletion in mock-infected cells (Figure 7B). This rate also slightly decreased after viral infection (Figure 7B). The upper glycolytic metabolites that are not channeled to R5P reach enolase (ENO, Figure 7C), which has a time course similar to HK in both cultivations. Except for lactate dehydrogenase (LDH, Figure 7D), all rates addressed thus far have a relatively small standard deviation. Cells in Cultivation 1 had a high LDH rate during exponential cell growth. During the cell death phase, this rate became negative since glucose was depleted, indicating lactate conversion to pyruvate. Similarly, in Cultivation 2, a high LDH rate was observed during the exponential phase of cell growth (Figure 7D). After virus infection, however, these cells still consumed glucose (Figure 3(A2)) and glycolysis remained active (see HK and ENO, Figure 7A,C), which led to a relatively high lactate production rate (Figure 7D). This high LDH rate allowed a very good prediction of the extracellular lactate accumulation in the bioreactor after infection (Figure 3(B2)). Pyruvate that was produced in glycolysis was converted via a variety of enzymes including PDH ($r_{PDH}$, Figure S5), transaminase ($r_{AlaTA}$, Figure S5), and pyruvate carboxylase (PC, Figure 8A). As previously reported for other cell lines [68,93], conversion of pyruvate to oxaloacetate (OAA) via PC resulted in a significant carbon supply to the TCA cycle from glycolysis. In fact, a relatively high PC rate was estimated during both cultivations’ exponential cell growth phases, which increased significantly not only during the cell death phase (Cultivation 1), but also after virus infection (Cultivation 2). Another important carbon source for the TCA cycle of animal cells is glutamine, following its conversion to glutamate via glutaminase. For mock-infected cells, the glutaminase rate was relatively high during exponential cell growth phase, but decreases after substrate depletion and subsequent cell death (GLNase, Figure 8B). However, glutaminase activity was not zero during this phase since glutamine synthetase ($r_{GS}$, in Figure S5 was still active). For Cultivation 2, a similar rate was estimated during the exponential phase of cell growth and after viral infection (Figure 8B) as the cells were infected before depletion of extracellular glutamine (Figure 3 (C2)). Another possible source of glutamate in the model is the degradation of other amino acids ($r_{AAex}$, Figure 8C). For both cultivations, the estimated amino degradation rate was high during the exponential cell growth phase and further increased during the cell death phase (Cultivation 1) and after virus infection (Cultivation 2). During the exponential phase of both cultivations, the resulting glutamate was converted to Keto via glutamate dehydrogenase (GLDH, Figure 8D). However, a stark difference was observed between these cultivations during the cell death phase (Cultivation 1) and after virus infection (Cultivation 2). The GLDH rate was estimated to be negative for Cultivation 1, indicating that glutamate was produced from Keto. As a result, glutamate accumulated intracellularly and was exported to the supernatant during this phase, leading to good agreement between model simulations and experimental data (Figure 3(F1)). On the other hand, the GLDH rate was estimated to remain high after virus infection in Cultivation 2. This prevented the accumulation of glutamate on the intracellular level, and likely contributed to the discrepancy between experimental data and model simulations after virus infection (Figure 3(F2)). Interestingly, in all scenarios, Keto was mostly produced from glutamate since the isocitrate dehydrogenase rates estimated were low (Figure 8E). The fact that the aspartate transaminase rate (AspTA) was estimated to be negative in all scenarios (Figure 8F) implies that the TCA cycle was truncated and only half of the TCA cycle was active in addition to the transamination reactions for energy production, as previously reported [52]. The other half of the TCA cycle, as usual, provided intermediates for biosynthesis through citrate [94]. Additional figures from model simulation and prediction of rates are provided in Figures S1 and S3–S5.

## 3. Materials and Methods

### 3.1. Shake Flask Cultivations

Pre-cultures of MDCK.SUS2 suspension cells were grown in shaker flasks (125 mL polycarbonate Erlenmeyer flasks, #431143, Corning^®^, New York city, NY, USA) with 50 mL working volume (wv), in a Multitron Pro incubator (Infors HT, Bottmingen, Switzerland) at 37 °C and 5% CO_2_ atmosphere with a shaking frequency of 180 rpm. Cells were passaged every 3–4 days with a seeding density of 0.5–0.8 × 10^6^ cells/mL.

IAV A/Puerto Rico/8/34 (H1N1) seed virus was used for infection, generated in adherent MDCK cells (ECACC # 84121903). The seed virus had a titer of 1.1 × 10^9^ TCID_50_/mL.

Cells were cultivated in a chemically defined, protein-free and animal component-free medium, Smif8 (Smif8 PGD 2×, supplemented with 5 mM glutamine, and 8 mM pyruvate), specifically developed for the cultivation of suspension MDCK cells [95].

Growth and infection experiments for model validation were performed using 500 mL shaking flasks (#4113-0500, Nalgene™, Thermo Scientific, Waltham, MA, USA) with an initial cultivation volume of 200 mL at 150 rpm. Two cultivations were performed, one mock-infection (Cultivation 1), where cells grew for about 200 h, and one infection (Cultivation 2), where cells were infected with IAV at about 49 h post inoculation. To achieve a synchronous infection of the cell population, a moi = 10 was used. Due to the relatively low cell concentration at time of infection (2.1 × 10^6^ cells/mL) and the high moi used, neither medium replacement nor trypsin addition (for virus activation) was necessary. In both cultivations, sterile Milli-Q water was added before sampling to compensate for water evaporation (1–2 mL/day) since the experiment was performed in a non-hydrated incubator.

### 3.2. Analytics

#### 3.2.1. Cell Count and Cell Volume

A Vi-Cell counter (XR #731050, Beckman Coulter, Krefeld, Germany) was used to determine viable cell counts and cells diameters. Due to the presence of cell aggregates, MDCK.SUS2 cells were trypsinized before counting. Therefore, 1 mL of the cell suspension was centrifuged using a tabletop centrifuge (800× *g*, 1 min, RT), 900 µL of supernatant was removed and the cell pellet was resuspended by adding 500 µL of trypsin-EDTA solution (1×). In a next step, cells were incubated for 10 min at 37 °C, mixed with 400 µL of fetal bovine serum, triturated and analyzed. The average cell diameter was determined by taking 1000–18,000 stationary pictures of individual cells using the Vi-Cell counter. The total cell volume was determined from the product of the mean cell volume (assuming a spherical cell shape) and the viable cell concentration.

#### 3.2.2. Hemagglutination Activity Assay

The virus titer was estimated using the hemagglutination activity (HA) assay as described by Kalbfuss et al. [96]. The cell-free virus samples and standards were serially diluted (two rows, 2^1–12^ and 2^0.5–12^) in the wells of a 96-round-bottom-well plate (of 100 µL) with PBS. Afterwards, a chicken erythrocyte solution (100 µL) was added with a concentration of 2 × 10^7^ erythrocytes/mL (Ery, erythrocytes/mL) and incubated for 3–8 h at RT. The erythrocyte agglutination was evaluated using a plate reader (Infinite^®^ M200 microplate reader, Tecan Group, Männedorf, Switzerland) measuring the extinction at 700 nm. A curve function was fitted to the data and used to determine the dilution at which the agglutination stops, which corresponds to the HA activity. The virus titer is commonly expressed as logarithm of the hemagglutination units (HAU) per analysis volume: log_10_(HAU/100 µL). Assuming that the virus and erythrocyte concentration are equal at the highest diluted sample showing agglutination, the concentration of the total number of virus particles ($V_{p}$, virions per mL) can be calculated using Equation (1). The standard deviation of this assay is 0.08 log_10_(HAU/100 µL) [96].

(1) $$V_{p}=\left[Ery\right]\cdot HAU=2\cdot 10^{7}\frac{1}{mL}\cdot 10^{\log_{10}\left(HAU\right)}$$

#### 3.2.3. Imaging Flow Cytometry

The relative amount of infected cells, the percentage of vRNP in the cell nucleus and the percentage of apoptotic cells were determined using imaging flow cytometry, based on previously established assays [97]. Cell fixation was carried out using 1 mL of infected MDCK cells, which were mixed with paraformaldehyde to a final concentration of 2% and incubated at 4 °C for 30 min. Thereafter, the cells were washed with PBS (300× *g*, 10 min, 4 °C), added to 5 mL 70% ethanol (−20 °C) and stored at −20 °C. For staining, fixed cells in ethanol were centrifuged (300× *g*, 10 min, 4 °C) to remove the storage solution. The cell pellet was washed twice with FACS-buffer (PBS containing 0.1% BSA and 2% glycine) and blocked in PBS containing 1% BSA (30 min, 37 °C). vRNP positive cells were stained with a monoclonal mouse anti-NP antibody mAb61A5 [98] as a primary antibody, and Alexa Fluor 647-conjugated goat anti-mouse pAb (#A21235, Thermo Scientific, Waltham, MA, USA,) as a secondary antibody. All antibodies were incubated for 60 min at 37 °C in FACS-buffer. Between each incubation step, cells were washed twice with FACS-buffer (300× *g*, 10 min, 4 °C). Before analysis, nucleic DNA was stained with DAPI (50 mg/L, #6843.2, Carl Roth, Karlsruhe, Germany). Ten thousand single cells were analyzed with an ImageStream X Mark II (#100220, Merck, Darmstadt, Germany) using a 60× objective lens. Image processing was carried out with the IDEAS software (version 6.1). The vRNP-positive cells were considered infected and nucleic condensation and fragmentation were used as signs of apoptosis.

#### 3.2.4. Extracellular Metabolites

Samples were centrifuged at 300× *g* for 5 min at RT and the supernatant was used for the quantification of extracellular metabolites. Virus-containing samples were inactivated in a heat block at 80 °C for 3 min prior to analysis. In some cases, the cell-free supernatant was stored at −80 °C until their respective analysis. Glucose, lactate, glutamine, glutamate, and ammonium were quantified using a BioProfile 100 Plus analyzer (Nova Biomedical, Waltham, MA, USA) using external standards. Pyruvate was quantified using a Cedex Bio Analyzer (#06395554001, Roche Diagnostics, Mannheim, Germany).

#### 3.2.5. Intracellular Metabolites

Samples for the quantification of intracellular metabolites require a quenching step to limit metabolite degradation. Therefore, MDCK cells were quenched in a methanol ammonium bicarbonate (MeOH-AMBIC) solution, and separated by centrifugation using a method adapted from Selick et al. [99]. A thermostat was used (FP89-HL, JULABO, Seelbach, Germany) with silicone oil as a heat transfer liquid (KRYO 90, JULABO, Seelbach, Germany) to adjust the MeOH-AMBIC solution temperature to −40 °C [99]. The pH value of the MeOH Ambic solution was adjusted to pH 7.4 by adding 5 M HCl. Then, 10 mL of this solution was transferred to 15 mL polypropylene tubes (17/120 mm, CELLSTAR, Greiner, Pleidelsheim, Germany) and cooled in a cryostat to −40 °C (approximately 10 min.). Next, 2 mL of the cell suspension samples were added to the MeOH-AMBIC solution and the tube was inverted twice and centrifuged for one minute at 3000× *g* in a precooled (−20 °C) centrifuge (Sigma 4–16KS, swing bucket rotor #11650, Sigma Laborzentrifugen, Harz, Germany). The supernatant was removed with a Pasteur pipette connected to a peristaltic pump, 600 µL of −20 °C methanol/chloroform solution [100] was added, vortexed for 5 s and snap frozen in liquid nitrogen. Samples were stored at −80 °C for up to five days until metabolite extraction.

Metabolite extraction was based on previous work for adherent cells [101] and suspension cells [56]. During extraction, all solutions were stored on ice for 5 min, vortexed and transferred to an extraction tube (Safe-Lock Tubes, 2 mL, Eppendorf) containing 500 µL of chloroform. To each sample vial, 800 µL of extraction solution (methanol 47.4% and tricine 2 mM) was added, vortexed and transferred to an extraction tube (E1). The resulting two phases (chloroform and MeOH-buffer) were mixed thoroughly (vortex, 20 s, max. speed), and centrifuged for 5 min at 16,000× *g* (Biofuge Primo R, swing bucket rotor # 7592, Heraeus, Thermo Scientific, Waltham, MA, USA), which was precooled to 0 °C. The upper hydrophilic layer (MeOH-buffer) was removed and transferred to another extraction tube (E2). For the second extraction, 800 µL of extraction buffer was transferred to an extraction tube (E1), followed by the same steps as for the first extraction. The hydrophilic layers of the first and second extraction were combined (E2), heated to 85 °C for 5 min and centrifuged for 10 min at 16,000× *g*. The resulting extracts were transferred to storage tubes (Safe-Lock Tubes, 2 mL, ambra, Eppendorf AG, Hamburg, Germany) and stored at −80 °C until drying. The extracts were dried for 8–10 h at RT under a nitrogen gas stream and stored as dry powder at −80 °C until quantification. All solutions used for the previous steps were prepared before media sampling and precooled to the according temperature (Table S4A). Before quantification, dry powders were solved in 300–800 µL of ultra-pure LC-MS grade water (Milli-Q Type 1 plus LC-Pak Polisher, Merck, Darmstadt, Germany), vortexed and incubated at 4 °C for 15 min. The final volume of the reconstituted sample volume was adjusted to match the viable cell volume of the 2 mL sample. Reconstituted samples were vortexed, centrifuged at 16,000× *g* for 10 min at 4 °C and transferred to HPLC glass vials. Intracellular metabolites were quantified by liquid chromatography-mass spectrometry (LC-MS) with an ICS-5000 MSQ-plus system (Dionex, Thermo Scientific, Waltham, MA, USA) similar to the protocol of Ritter et al. [100]. Accordingly, the run time was reduced by 10 min. Furthermore, a more robust separation was achieved by skipping declining gradients of potassium hydroxide (KOH), which was used as eluent. For each measurement, 15 µL volume of the reconstituted samples was injected and metabolites were separated with two analytical anion-exchange columns (Dionex IonPac AS11, 2 × 250 mm, 30 °C) connected serially after an inline filter (35/5/0.45 µm) and a guard column (Dionex IonPac AG11, 2 × 50 mm). A potassium hydroxide gradient (2–100 mM) was used as eluent with a constant flow of 0.35 mL/min (≅2300 psi) using an in-line eluent generator (Dionex ICS-5000 + EG). Post column continuous eluent suppression (Dionex AERS 500, 2 mm) permitted the detection of metabolites using a serial connected conductivity (Dionex ICS-5000+ CD), UV (Dionex ICS-Series VWD, single-channel, 260 nm) and MS detector (MSQ Plus Mass Spectrometer). The eluent flow through the columns was directed to the MSQ from 2 to 6 min and from 9 to 53 min, due to the elution of a high amount of chlorine ions between 6–9 min. Several metabolites were separated with this optimized gradient (Table S4B) and quantified using an external standard mix of all metabolites. Single ion monitoring was used to detect specific predetermined metabolite ions (Table S4C). The standard mix stock (Table S4D) stored at −80 °C was diluted with tricine buffer (10 mM tricine, 6 mM NaCl) and water to simulate the extraction matrix (Table S4E). All standards and samples were measured as analytical triplicates. Intracellular metabolite concentrations were calculated by the product of the amount of metabolites ([C], concentration of metabolite in mmol per L) in the reconstituted extract measured via LC-MS ($\left[C_{analyte}\right]$) and the reconstitution volume ($V_{rec}$), divided by the product of the viable cell volume ($V^{c}$) in the extracted sample and the volume of the sampled cell suspension ($V_{s}$, 2 mL), as in Equation (2). The experimental data set is provided in Supplementary File S4.

(2) $$\left[C\right]=\frac{\left[C_{analyte}\right]\cdot V_{rec}}{V^{c}\cdot V_{s}}$$

### 3.3. Model Definition

**Figure 9:**
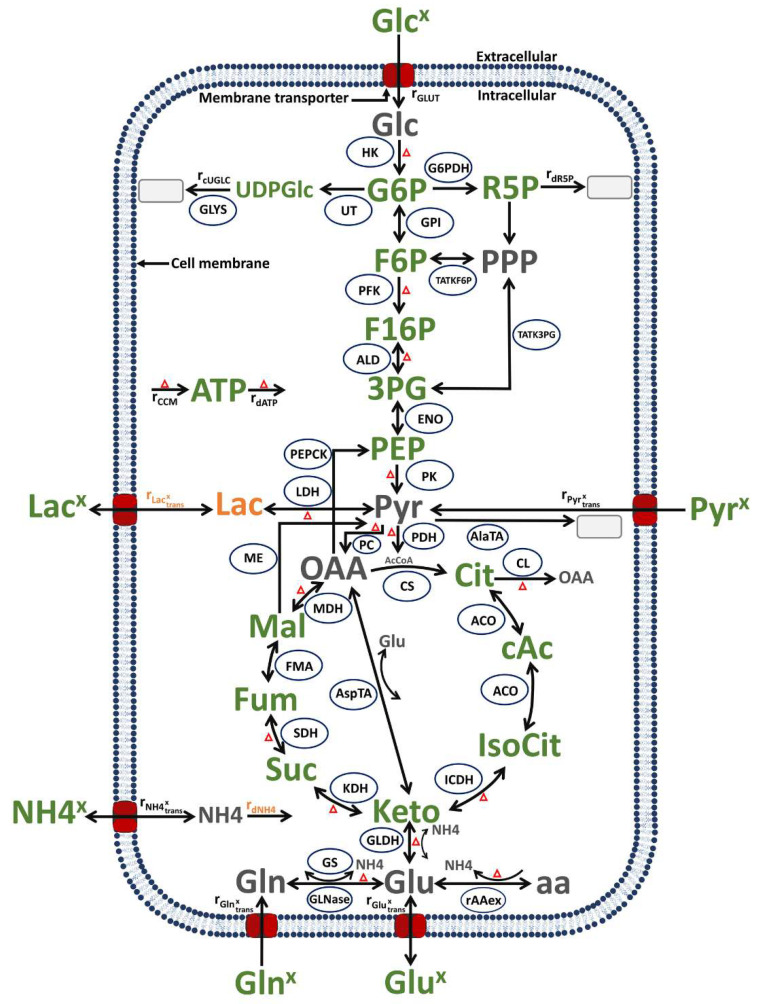
Simplified model of the central carbon and energy metabolism of MDCK cells, modified from [52] (changes to the previous model in orange). In green: metabolites and product measured experimentally; in grey: metabolites not measured. Ellipsoids: enzymes considered in the model. Arrows: reactions or transport, with the arrowhead indicating the reaction or transport direction (for simplification, reversible reactions have an arrow for both directions). Grey rectangles: sinks or metabolites not accounted for in the model. Red triangles: all the reactions included in the energy balance. Abbreviations of metabolites and product: 3PG: 3-phosphoglycerate, AcCoA: acetyl coenzyme A, ATP: adenosine tri-phosphate, cAc: cis-Aconitate, Cit: citrate, F16P: fructose 1,6-biphosphate, F6P: fructose-6-phosphate, Fum: fumarate, G6P: glucose-6-phosphate, Glc: glucose (intracellular), Glc^x^: glucose (extracellular), Gln: glutamine (intracellular), Gln^x^: glutamine (extracellular), Glu: glutamate (intracellular), Glu^x^: glutamate (extracellular), IsoCit: iso-citrate, Keto: alpha-ketoglutarate, Lac^x^: lactate (extracellular), Mal: malate, NH4: ammonium (intracellular), NH4^x^: ammonium (extracellular), OAA: oxaloacetate, PEP: phosphoenolpyruvate, Pyr: pyruvate (intracellular), Pyr^x^: pyruvate (extracellular), R5P: ribose-5-phosphate, SUC: succinate, UDPGlc: uridine diphosphate Glucose. Abbreviations of enzymes and transport rates: HK: hexokinase, G6PDH: glucose-6-phosphate dehydrogenase, UT: uridyl transferase, GLYS: glycogen synthetase, GPI: glucose-6-phosphate isomerase, TATKF6P: transaldolase and transketolase, TATK3PG: transaldolase and transketolase, PFK: phosphofructokinase, ALD: aldolase, ENO: Enolase, $r_{\mathrm{CCM}}$: reaction rate with overall ATP production, r_dATP_: reaction rate with overall ATP consumption, PK: pyruvate kinase, PEPCK: phosphoenolpyruvate-kinase, LDH: lactate dehydrogenase, PC: pyruvate carboxylase, PDH: pyruvate dehydrogenase, AlaTA: alanine transaminase, ME: malic enzyme, CS: citrate synthetase, CL: citrate lyase, ACO: aconitase, ICDH: isocitrate dehydrogenase, KDH: ketoglutarate dehydrogenase, AspTA: aspartate transaminase, SDH: succinate dehydrogenase, FMA: fumarase, MDH: malate dehydrogenase, GLDH: glutamate dehydrogenase, GS: glutamine synthetase, GLNase: glutaminase, $r_{\mathrm{AAex}}$: amino acids degradation, in the following reaction rates are listed as “reaction rate accounting for”: $r_{\mathrm{dR}5P}$: ribose-5-phosphate consumption, $r_{dNH4}$: ammonium consumption, $r_{\mathrm{uGLC}}$: other uridine diphosphate glucose consumption, $r_{\mathrm{GLUT}}$: extracellular glucose consumption, $P_{yr_{\mathrm{trans}}^{x}}$: extracellular pyruvate consumption, $r_{\mathrm{NH}4_{\mathrm{trans}}^{x}}$: extracellular ammonium production from intracellular rates, $r_{{\mathrm{Gln}}_{\mathrm{trans}}^{x}}$: extracellular glutamine consumption, $r_{Lac_{\mathrm{trans}}^{x}}$: extracellular lactate production/consumption from intracellular rates and $r_{{\mathrm{Glu}}_{\mathrm{trans}}^{x}}$: extracellular glutamate production from intracellular rates.

The model established for this study follows, in structure and basic assumptions, a model established previously for the human suspension cell line AGE.HN [52]. This model describes cell growth, virus production and metabolism by coupling model variables from a segregated growth model (for the macroscopic scale) with a structured model of the central carbon metabolism (for the microscopic scale). Compared to the previous approach [52], various modifications were made to cover the virus infection phase. This includes a description of virus production (the concentration of all virus particles in the supernatant) as well as changes in cell growth and the death of infected cells that are not related to the substrate availability. Further modifications, described in more detail below, refer to the introduction of new states in the structured part of model dealing with the central metabolism. This includes new state variables for the intracellular concentration of lactate and ammonium, a rate to describe the consumption of pyruvate, the transport of ammonium and lactate, and some reactions/transport kinetics modified in order to fit the experimental data sets obtained for shake flask cultivations (compared to stirred tank bioreactors described before [55]). Finally, equations related to alpha-1-antitrypsin production were removed. An overview of this model is shown in Figure 9 and a detailed model description and a list of symbols used are found in the Supplementary Files S1 and S2, respectively.

#### 3.3.1. Segregated Cell Growth and Infection Model

The segregated cell growth and infection model describes the dynamics of cells, substrates, metabolic by-products, and virus particles on the macroscopic scale.

The specific transition rate ($r_{trans}$, Equation (3)), between the cell classes is described by a Monod equation (μ) using the extracellular glucose concentration ($Glc^{x}$) multiplied with a constant (δ) that depends on the number of cell classes considered in the model (for a mathematical explanation, see [53]). The transition rate is not equal for infected and mock-infected cells; to use the same model variable for both, two step-functions are used. The first step function (ε) is 0 for mock-infected cells and 1 for infected cells.

(3) $$\begin{array}{l} r_{trans}=\mu \delta \left(1-\Phi_{1}\varepsilon \right)\\ with\left\{ \begin{array}{l} \mu =\mu_{\max}\frac{\left[Glc^{x}\right]}{k_{Glc^{x}}^{m}+[Glc^{x}]}\\ \delta =\frac{1}{2^{1/N^{c}}-1} \end{array}\right. \end{array}$$

The parameter *μ*_max_ is the maximum specific cell growth rate and $k_{Glc^{x}}^{m}$ is the Monod constant. A time dependent sigmoidal step function (Φ_1_ and Φ_2_) are used to take into account the decrease in µ and increase in the death rate (*k_d_*, Equation (6)), respectively, after virus infection. This function was previously used to describe the transition of viable cell to apoptosis for IAV infected cells [102], and the smoothness of the transition depends on a constant ($\rho_{1}$, manually adjusted) and the number of hours post infection (hpi).

(4) $$\Phi_{1}=\frac{1}{1+e^{\left(\rho_{1}-hpi\right)}}$$

The inhibition factor (*f*, Equation (5)) corresponds to a limitation in the number of cells that start cell division and is related to extracellular glucose concentration ($Glc^{x}$). It has a maximum value of 1 (corresponding to 100% of the first cell class starting the division process) and a minimum of 0 (0% of the cells of the first cell class). A scaling constant (α) is used to adjust the changes in the inhibition factor. Here, it was assumed that infected cells do not divide anymore by using the step function (ε) introduced above.

(5) $$f=\left(1-e^{-\alpha \frac{Glc^{x}}{X_{v}}}\right)\left(1-\varepsilon \right)$$

Overall, the inhibition factor (f, Equation (5)) is effectively zero after virus infection and the growth rate decreases with a smooth step such as function ($\Phi_{1}$) leading also to a smooth decrease in the transition rate ($r_{trans}$, Equation (3)). This implies that even after a synchronous infection, some cells of the classes ($X_{2}-X_{4}$, Equations (3)–(5) in Supplementary File S1) finish the division process leading to a small but noticeable increase in the cell concentration shortly after the infection step. More importantly, however, this also has an impact on the dynamics of the mean cell diameters and consequently the cell-specific volume and maximum enzyme activities. A more detailed discussion and data showing differences between the assumption of a null transition rate ($r_{trans}$) and the smooth decrease in the transition rate after infection and their impact on the model prediction of viable cell concentrations and mean cell diameters can be found in Section 3.2 of the Supplementary File S1.

During the cell growth phase, cell death after substrate depletion is described by the rate ($k_{d}$, Equation (6)) as introduced before by Ramos et al. [52]. It considers a basal cell death rate ($k_{d}^{\min}$) related to cell age, mechanical damage, etc. and an additional term ($k_{d}^{\max}$), which is inversely correlated with the effective cell growth rate. The parameter β is an adjustable constant for which effective growth rate and death rate are equal.

(6) $$k_{d}=\left(k_{d}^{\min}+k_{d}^{\max}{\left(\frac{\beta }{\beta +\mu f}\right)}^{2}\right)\left(1-\varepsilon \right)+\left(k_{d\inf}^{\min}+k_{d\inf}^{\max}\Phi_{2}\right)\varepsilon$$

Mechanisms of cell death are obviously not the same for infected and mock-infected cells. In particular, infected cells mainly die due to virus-induced apoptosis. Accordingly, death rate ($k_{d}$) in these two scenarios also makes use of the step function (ε) introduced previously, and a smooth step-like function ($\Phi_{2}$). The latter is similar to the previously introduced step function ($\Phi_{1}$, Equation (4)), where the constant parameter $\rho_{1}$ was replaced with a different value ($\rho_{2}$, manually adjusted). More specifically, for infected cells, it is assumed that a basal cell death rate ($k_{d\inf}^{\min}$) applies and, in addition, a term ($k_{d\inf}^{\max}$) is added, which is a time-related increase in the cell death rate caused by using the time step function ($\Phi_{2}$). The time-related increase in the cell death rate is closely related to the transition to an apoptotic state typically observed for infected cells, as described by Rüdiger et al. [102].

Finally, a new state variable was required to describe the virus dynamics. As the maximum number of virions produced per cell, the CSVY ($v_{p}=11,989$), corresponds to only 0.55% of the mean cell volume, no attempts were made to describe the virus particle increase by substrate- or precursor-based kinetics. Instead, the concentration of all virions ($V_{t}$) released from infected cells was considered using Equation (7), where $v_{p}$ is the virus production rate and $\Phi_{3}$ is a smooth step function to account for the time delay between virus infection and virus release (eclipse phase). This step function is similar to the previously introduced step function ($\Phi_{1}$, Equation (4)), where the constant parameter $\rho_{1}$ was replaced with a different value ($\rho_{3}$, manually adjusted).

(7) $$\frac{d\left[V_{t}\right]}{dt}=X_{v}\cdot v_{p}\Phi_{3}$$

#### 3.3.2. Structured Model of the Central Carbon Metabolism

The structured model for central carbon metabolism describes the microscopic scale (Figure 9), and comprises reactions from glycolysis, citric acid cycle, glutaminolysis, transamination, and the pentose phosphate pathway. The model was coupled with the structured cell growth model using growth-related variables such as the cell-specific volume, and uptake or release rates of the extracellular substrates and metabolic by-products, respectively. This link was accomplished by converting the model variables between the different scales using (Equation (1) in Supplementary File S1). This implies that substrate consumption rates were used as input and metabolic by-products accumulation rates were the outputs of the intracellular metabolic network, effectively allowing the description of dynamics of metabolites both at the extra- and the intracellular level. To describe the dynamics of metabolic product accumulation on the extracellular level, linked to the central carbon metabolism, the concentrations of intracellular lactate and ammonium were considered (Equations (25) and (32) in Supplementary File S1). In addition, to allow fitting of the dynamics of PEP, 3-phosphoglycerat, extracellular lactate (Lac^x^), extracellular glutamate (Glu^x^), extracellular ammonium (NH4^x^), Cit and Keto, the model described by Ramos et al. [52] was updated in a few cases. Whether this was because parts of the central carbon metabolism of suspension MDCK cells differ from metabolism of suspension AGE1.HN cells or that metabolism of suspension cells differs for cultivations performed in shaker flasks (MDCK cells) and stirred tank reactor (AGE1.HN cells) or both cannot be decided for now. The main changes concern few enzymes or transporter kinetics such as enolase (ENO), aldolase (ALD), pyruvate kinase (PK), pyruvate carboxylase (PC), glutaminase (Glnase), ammonium transporter ($NH4_{trans}^{x}$), citrate lyase (CL) and aspartate transaminase (AspTA) according to the literature [70]. The equations for the structured intracellular metabolism states (ODEs and kinetics) along with the symbols used are provided in the Supplementary Files S1 and S2, respectively. The MATLAB version of the model for simulation is provided in Supplementary File S3.

### 3.4. Parameter Fitting and Model Simulation

The model was implemented using the Systems Biology Toolbox 2 [103] in MATLAB (Version R2012b, the Mathworks, Inc., Natick, MA, USA). Model simulations were carried out using MATLAB executable (mex) of the model, which speeds up the execution time significantly. For the integration of ODEs, the CVODE from SUNDIALS was used [104]. For the parameter optimization, a covariance matrix adaptation evolution (CMA-ES) algorithm was used that enables stochastic and derivative free global optimization [105,106]. The CMA-ES was used as it performed better than methods used in a similar study [52]. For the implementation of the bootstrap method, in silico data were generated through Monte-Carlo sampling using the average of the experimental data and their corresponding standard deviation. In a next step, parameters were fitted using these newly generated data sets (provided in Supplementary File S5, Tables S1 and S2). Additionally, figures from model simulations using the over 2500 parameter sets obtained via the bootstrap method can be found in Figure S2.

In a first step, parameters related to cell growth and extracellular glucose dynamics (Equations (3)–(6) and (10) in Supplementary File S1) were fitted using the experimental data from the mock-infected culture. Next, parameters for the infected culture were fitted (Equations (6)–(7); step functions $\Phi_{1}$, $\Phi_{2}$, $\Phi_{3}$) to describe virus dynamics and the transition from exponential cell growth phase to cell death after virus infection, respectively. Finally, the parameters related to the dynamics of the remaining extracellular (Equations (11)–(15) in Supplementary File S1) and intracellular metabolites were fitted (Equations (16)–(38) in Supplementary File S1). During optimization, the initial values for the parameters were either taken from the literature [45,52,58] or estimated from experimental data (i.e., the specific cell growth rate). Note that, apart from the description of virus dynamics, cell growth and cell death, the same kinetics (transport kinetics, intracellular reactions kinetics) and the same set of parameters were used for both the infected and mock-infected cells. Overall, 143 parameters were fitted using 360 data points applying Equation (8) where p is the parameter set, $e=1,\ldots ,T^{e}$ the number of experiments, $n=1,\ldots ,T^{n}$ is number of states and $i=1,\ldots ,T^{i}$ the time, while η is the weighting to the maximum for state n in the experiment e. Due to the complexity of the developed model and the limited number of experimental data sets to test the model predictability, parameter overfitting (fitting of the noise in the data set) cannot be ruled out in this instance. Nevertheless, parameter fitting of over 2500 data sets generated in silico using Monte-Carlo sampling (as described above) resulted in reasonably low parameter ranges (Tables S1 and S2). Furthermore, model simulations using these parameters show similar dynamics (Figure S2).

(8) $$\underset{p}{\min}{\sum_{e=1}^{T^{e}}\sum_{n=1}^{T^{n}}\sum_{i=1}^{T^{i}}\left(\frac{prediction_{eni}-data_{eni}}{\eta }\right)}^{2}$$

Initial values for the state variables of the cell classes (Equations (3)–(5) in Supplementary File S1) and extracellular metabolite concentrations (Equations (10)–(15) in Supplementary File S1) were adjusted manually within the error range of the first experimental data point. The same principle was used for the state variable that describes the virus particle concentration (Equation (7)). Initial conditions for the concentration of intracellular metabolites were obtained via simulation of a pre-culture coming from late exponential growth phase (85 h) as described in the Materials and Methods section (shake flask cultivation). For the simulation of extracellular metabolites of the pre-culture, the known medium composition was used. For intracellular metabolites state variables, the concentration of the first sampling time point was used (in case these data were not available, a low concentration was assumed taking into account values from previous studies [45,52,53,54]). A list of all initial values is given in Table S3.

## 4. Conclusions

Overall, based on a very comprehensive data set, most parameters of the model could be estimated reliably (see data in Supplementary File S5, Tables S1 and S2). Furthermore, based on one set of parameters estimated using experimental data from a mock-infected cell culture, the model accurately simulated the dynamics of mock-infected cells and generally correctly predicted the dynamics of virus-infected cells for up to 60 hpi. This implies that mock-infected and infected cells do not differ much in their metabolism for the initial period of virus replication and virus release for high moi scenarios. Furthermore, results obtained clearly suggest that the majority of differences in metabolism observed after infection are directly related to cessation of cell growth and the subsequent transition to apoptosis and cell death. For the final stage of virus production, which is of minor relevance in IAV vaccine manufacturing, a straightforward interpretation of some of the results is difficult. This concerns, in particular, the relatively high accumulation of glutamate and ammonium in the supernatant. As a release from lysing cells (by far) cannot explain their absolute level, the conversion of extracellular amino acids by enzymes released from the cells has to be assumed. Nevertheless, differences in the dynamics of metabolism occur after virus infection. Often, they were shown to be host cell-dependent [22,23,24,25]. However, differences were also noticed for the replication of the same influenza A virus subtype [21]. Clearly, future studies should be performed to assess the impact of cessation of cell growth and transition into apoptosis on yield after infection with other influenza viruses relevant for vaccine production to establish a broader database for simulation studies and to contribute to a better understanding of the complex interactions of viruses with their host cells.

## Data Availability

The data presented in this study are available as Supplementary Material.

## Supplementary Materials

The following supporting information can be downloaded at: https://www.mdpi.com/article/10.3390/metabo12030239/s1, Figure S1: Box-and-whisker plot of intracellular rates from model simulations for mock-infected and infected suspension MDCK cells; Figure S2: Results of 2500 simulations using the parameters obtained via a bootstrap method for the model established for mock-infected and infected suspension MDCK cells; Figure S3: Simulated rates and standard deviations for Cultivation 1 and 2 (mock-infected ─, infected), calculated from 2500 simulations via a bootstrap method; Figure S4: Box plot with average and standard deviation of intracellular rates estimated from model simulations for mock-infected and infected suspension MDCK cells; Figure S5: Simulated rates for Cultivation 1 and 2 (mock-infected ─, infected); Table S1: Estimated global parameters of the structured intracellular model with confidence intervals between 0.025-quantile and 0.972-quantile (Q0.025–Q0.975), calculated via a bootstrap method with 2500 runs; Table S2: Estimated global parameters of the segregated cell growth model with confidence intervals between 0.025-quantile and 0.975-quantile (Q0.025–Q0.975), calculated via a bootstrap method with 2500 runs; Table S3: Initial conditions used for the simulation of the model established for mock-infected and infected suspension MDCK cells; Table S4: Experimental procedures; Supplementary File S1: Model definitions and equations; Supplementary File S2: List of symbols; Supplementary File S3: Demo version of the MATLAB model; Supplementary File S4: Experimental data; Supplementary File S5: Bootstrap method results; Supplementary File S6: Additional studies regarding the impact of different number of cell classes, the impact of usage of a growth-related time step function after virus infection and the impact of intracellular metabolite and enzyme leakage into the supernatant due to cell lysis.
