# Supplementary material for: Impact of Influenza A Virus Infection on Growth and Metabolism of Suspension MDCK Cells Using a Dynamic Model
Source: Metabolites. 2022 Mar 12;12(3):239. doi: 10.3390/metabo12030239 (PMC8950586; doi:10.3390/metabo12030239)
Supplement: Supplementary file 1 [file metabolites-12-00239-s001.zip › Figure S1.pdf]

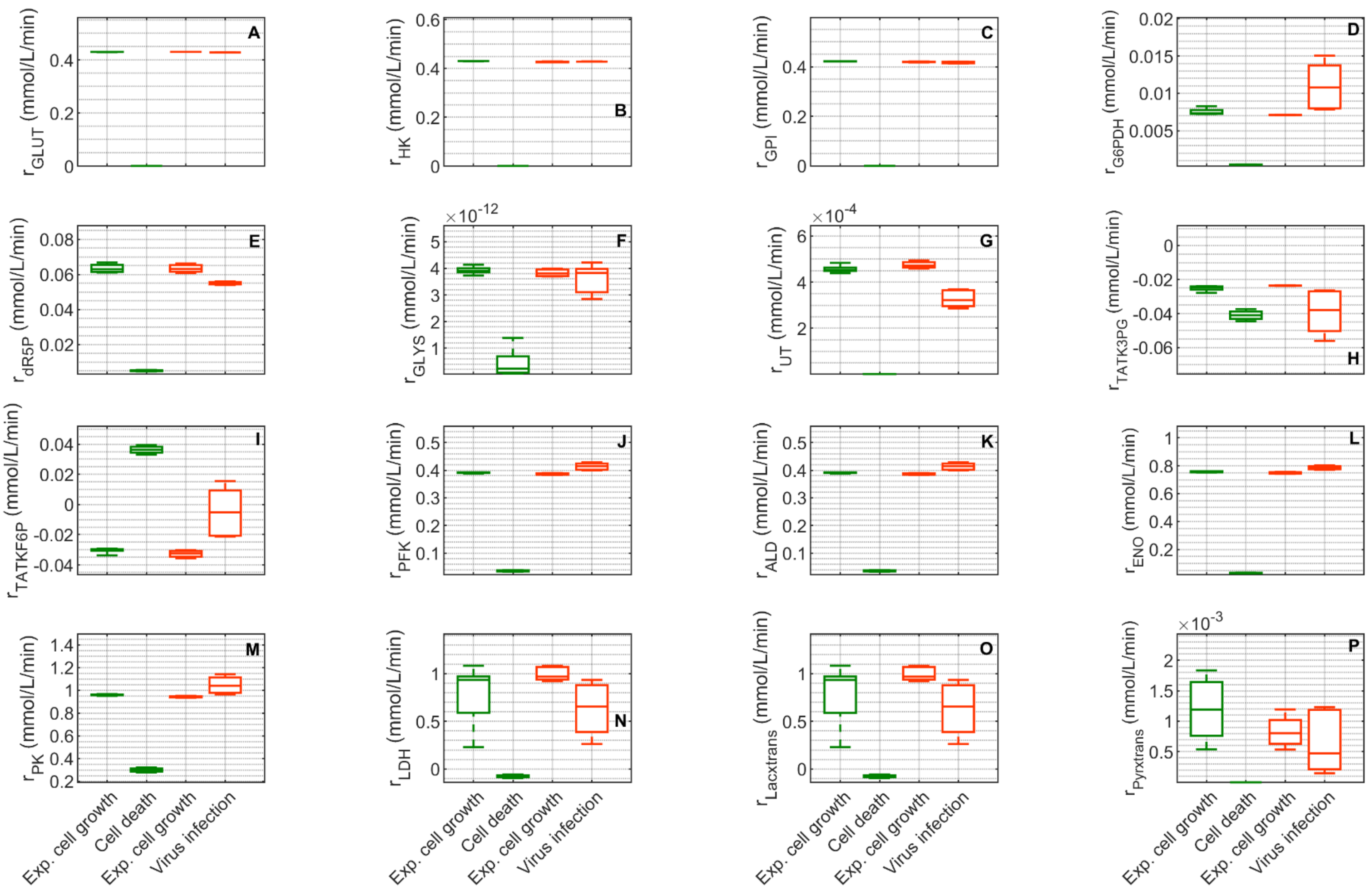

Figure S1A. Box-and-Whisker plot of intracellular rates from model simulations for mock-infected and infected suspension MDCK cells. (A) Glucose transporter rate, (B) hexokinase rate, (C) glucose-6-phosphate isomerase rate, (D) glucose-6-phosphate dehydrogenase rate, (E) ribose-5-phosphate consumption rate, (F) glycogen synthetase rate, (G) uridyl transferase rate, (H) transaldolase and transketolase rate, (I) transaldolase and transketolase rate, (J) phosphofructokinase rate, (K) aldolase rate, (L) enolase rate, (M) pyruvate kinase rate, (N) lactate dehydrogenase rate, (O) extracellular lactate production/ consumption rate and (P) extracellular pyruvate consumption rate. Calculated from the exponential growth phase of Cultivation 1 (□, 6-108h), death phase of Cultivation 1 (□, 146-169 h), exponential growth phase of Cultivation 2 (□, 6-48 h) and during virus replication Cultivation 2 (□, 49.9-107 h). The bar represents the median, the box is the first and third quartile, and the whisker the minimum and maximum of the rates from the model simulations of the corresponding cultivation phase.

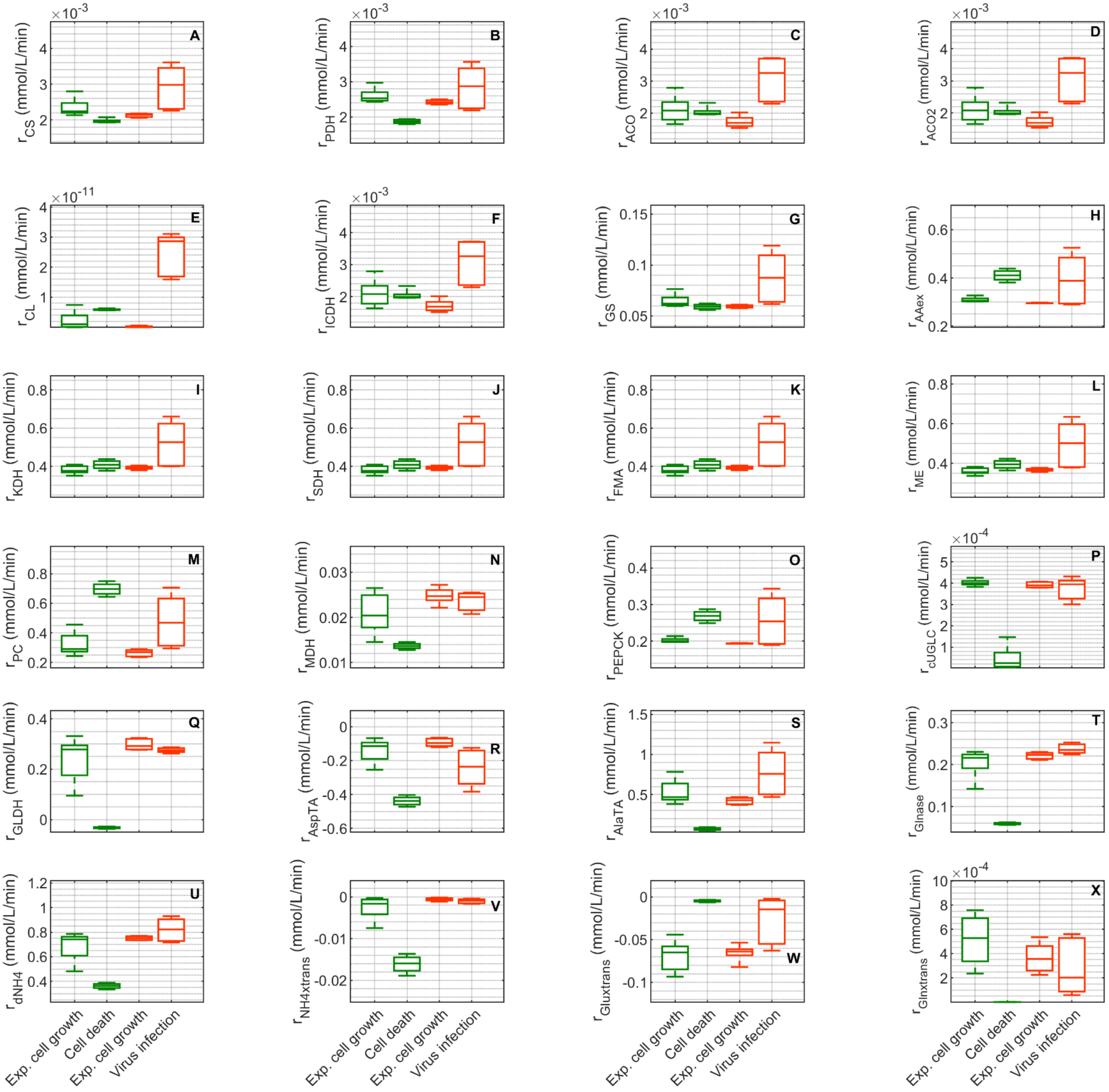

Figure S1B. Box-and-whisker plot of intracellular rates from model simulations for mock-infected and infected suspension MDCK cells. (A) Citrate synthetase rate, (B) pyruvate dehydrogenase rate, (C) aconitase rate, (D) aconitase rate, (E) citrate lyase rate, (F) isocitrate

dehydrogenase rate, (G) glutamine synthetase rate, (H) amino acids degradation rate, (I) ketoglutarate dehydrogenase rate, (J) succinate dehydrogenase rate, (K) fumarase rate, (L) malic enzyme rate, (M) pyruvate carboxylase rate, (N) malate dehydrogenase rate, (O) phosphoenolpyruvate-kinase rate, (P) uridine diphosphate glucose consumption rate, (Q) glutamate dehydrogenase rate, (R) aspartate transaminase rate, (S) alanine transaminase rate, (T) glutaminase rate, (U) ammonium consumption rate, (V) extracellular ammonium production rate, (W) extracellular glutamate production rate and (X) extracellular glutamine consumption rate. Calculated from the exponential growth phase of Cultivation 1 (□, 6-108h), death phase of Cultivation 1 (□, 146-169 h), exponential growth phase of Cultivation 2 (□, 6-48 h) and during virus replication Cultivation 2 (□, 49.9-107 h). The bar represents the median, the box is the first and third quartile, and the whisker the minimum and maximum of the rates from the model simulations of the corresponding cultivation phase.

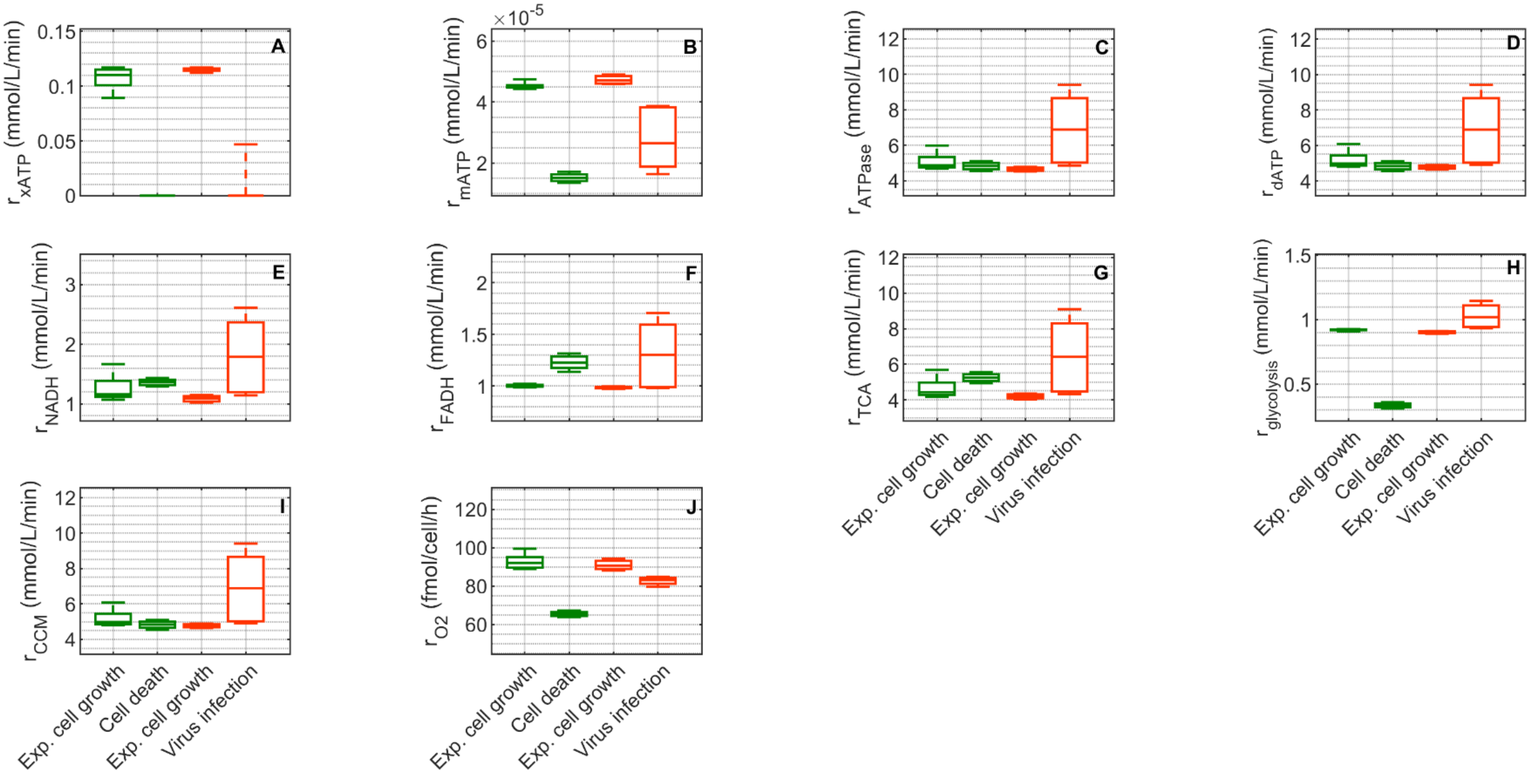

Figure S1C. Box-and-whisker plot of intracellular rates from model simulations for mock-infected and infected suspension MDCK cells. (A) ATP consumption for growth, (B) ATP consumption for maintenance, (C) ATPase rate, (D) net consumption of ATP, (E) NADH oxidative phosphorylation rate, (F) FADH oxidative phosphorylation rate, (G) TCA net ATP production rate, (H) glycolytic net ATP production rate, (I) net production of ATP and (J)

theoretical oxygen consumption rate. Calculated from the exponential growth phase of Cultivation 1 (□, 6-108h), death phase of Cultivation 1 (□, 146-169 h), exponential growth phase of Cultivation 2 (□, 6-48 h) and during virus replication Cultivation 2 (□, 49.9-107 h). The bar represents the median, the box is the first and third quartile, and the whisker the minimum and maximum of the rates from the model simulations of the corresponding cultivation phase.
