# Supplementary material for: Impact of Influenza A Virus Infection on Growth and Metabolism of Suspension MDCK Cells Using a Dynamic Model
Source: Metabolites. 2022 Mar 12;12(3):239. doi: 10.3390/metabo12030239 (PMC8950586; doi:10.3390/metabo12030239)
Supplement: Supplementary file 1 [file metabolites-12-00239-s001.zip › Figure S2.pdf]

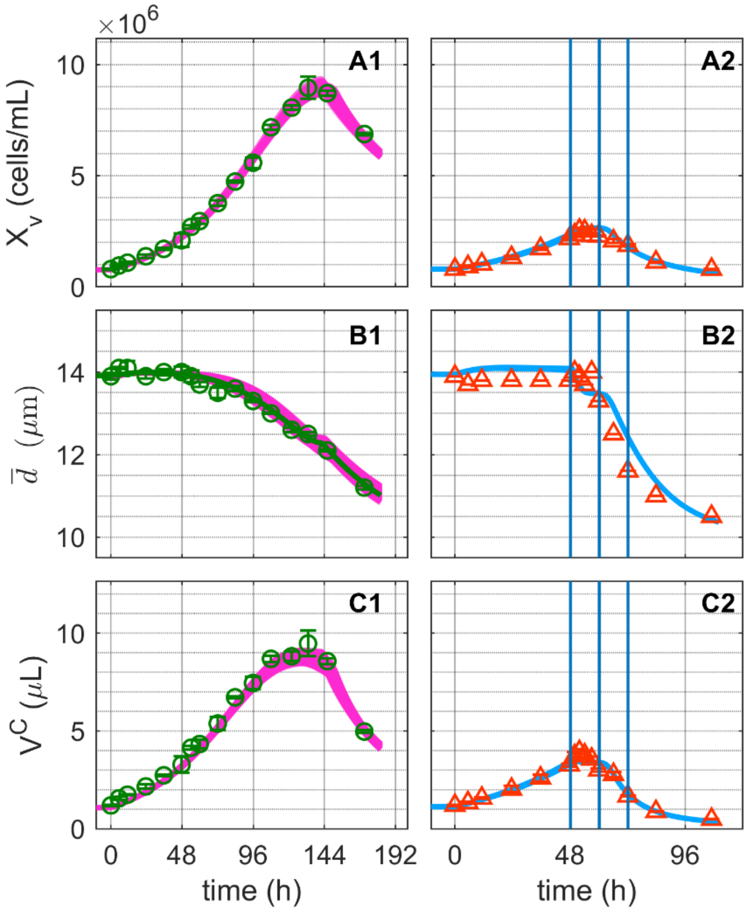


**Figure S2A. Results of 2500 simulations using the parameters obtained via a bootstrap method for the model established for mock-infected and infected suspension MDCK cells.** (A1–2) Viable cell concentration, (B1–2) mean cell diameter and (C1–2) viable cell volume. Data and error bars represent the mean and standard deviation of technical triplicates for two independent experiments (mock-infected Θ and infected Δ). Light Blue and pink patches: Lines corresponding to 2500 model simulations (thicker during towards end of cultivation due to higher sensibility parameters related to cell death). Vertical blue lines correspond to (0, 12 and 24 h post infection respectively). Experimental data used for parameter estimation (A1, B1, C1). The grey lines indicate the limit of quantification for each metabolite and the grey data points are under the limit of quantification.

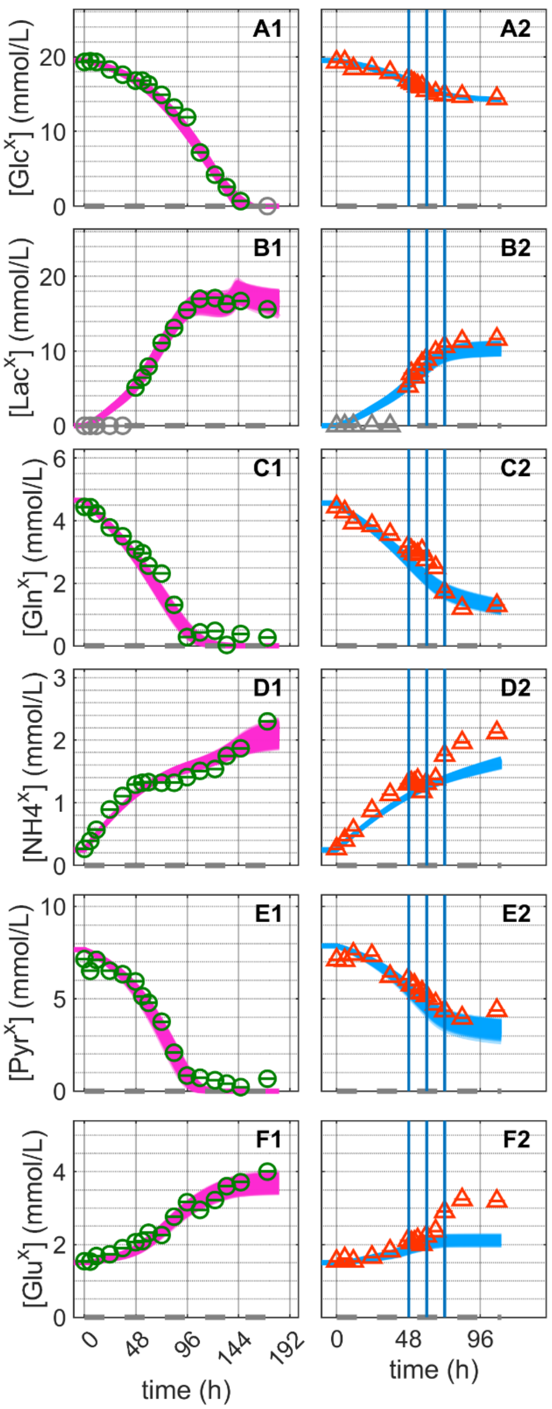


**Figure S2C. Results of 2500 simulations using the parameters obtained via a bootstrap method for the model established for mock-infected and infected suspension MDCK**

**cells.** (A1–2) Glucose, (B1–2) lactate, (C1–2) glutamine, (D1–2) extracellular ammonium, (E1–2) pyruvate and (F1–2) glutamate. Data and error bar represent mean and standard deviation of technical triplicates for two independent experiments (mock Θ and infected Δ). Light Blue and pink patches: Lines corresponding to 2500 model simulations (thicker during towards end of cultivation due to higher sensibility of parameters related to cell death). Vertical blue lines correspond to (0, 12 and 24 h post infection respectively). Experimental data used for parameter estimation (A1, B1, C1, D1, E1, F1). The grey lines indicate the limit of quantification for each metabolite and the grey data points are under the limit of quantification.

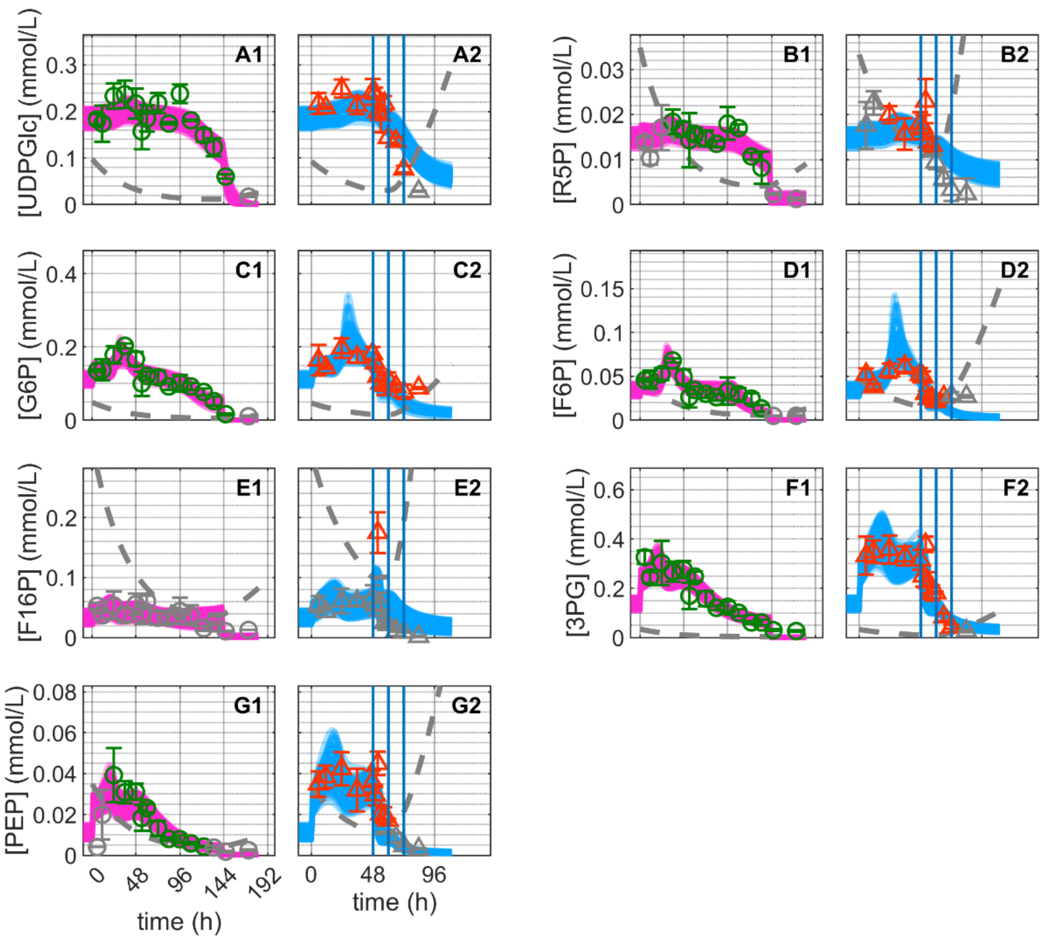


**Figure S2D. Results of 2500 simulations using the parameters obtained via a bootstrap method for the model established for mock-infected and infected suspension MDCK cells.** (A1–2) Uridine diphosphate glucose, (B1–2) ribose-5-phosphate, (C1–2) glucose-6-phosphate, (D1–2) fructose-6-phosphate, (E1–2) fructose-1,6-biphosphate, (F1–2) 3-phosphoglutarate and (G1–2) phosphoenolpyruvate. Data and error bar represent mean and standard deviation of technical triplicates for two independent experiments (mock Θ and infected Δ). Light Blue and pink patches: Lines corresponding to 2500 model simulations (thicker during towards end of cultivation due to higher sensibility of parameters related to cell death). Vertical blue lines correspond to (0, 12 and 24 h post infection respectively). Experimental data used for parameter estimation (A1, B1, C1, D1, E1, F1, G1). The grey

lines indicate the limit of quantification for each metabolite and the grey data points are under the limit of quantification.

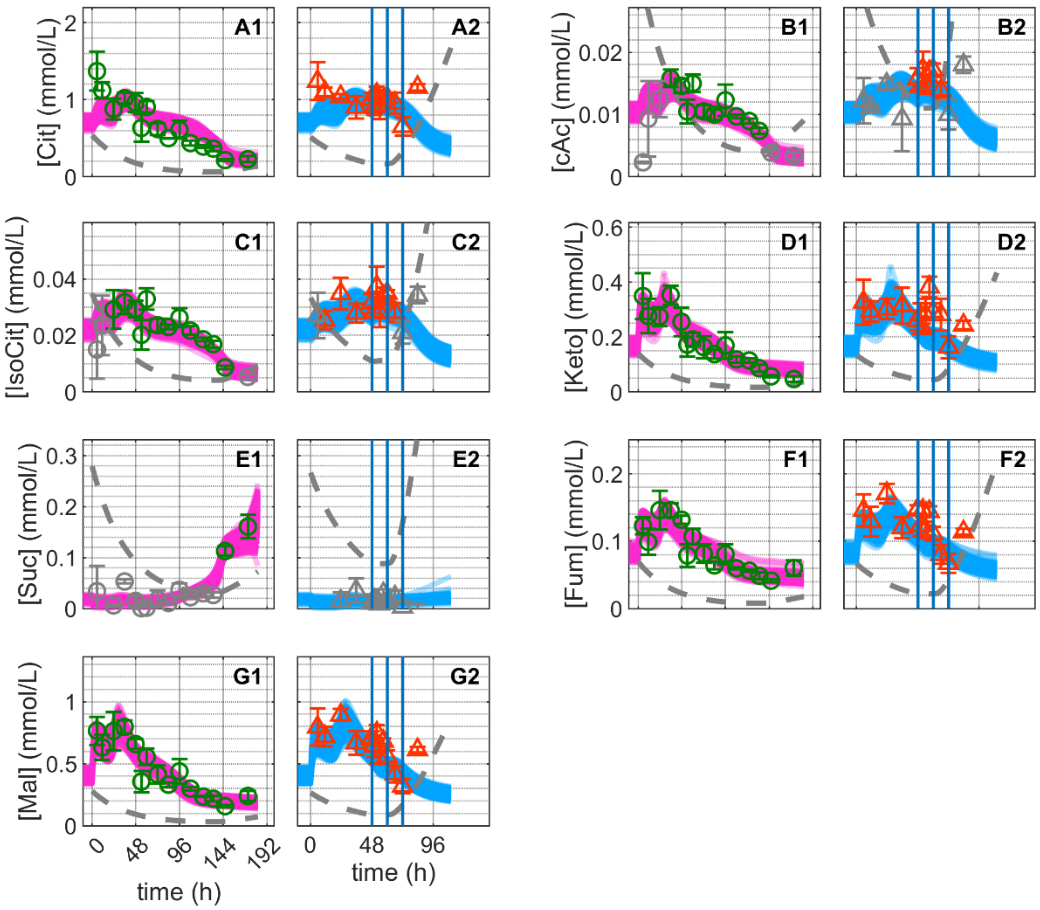


**Figure S2E. Results of 2500 simulations using the parameters obtained via a bootstrap method for the model established for mock-infected and infected suspension MDCK cells.** (A1–2) Citrate, (B1-2) cis-aconitate, (C1–2) iso-citrate, (D1–2) alpha-ketoglutarate, (E1–2) succinate, (F1–2) fumarate and (G1–2) malate. Data and error bar represent mean and standard deviation of technical triplicates for two independent experiments (mock Θ and infected Δ). Light Blue and pink patches: Lines corresponding to 2500 model simulations (thicker during towards end of cultivation due to higher sensibility of parameters related to cell death). Vertical blue lines correspond to (0, 12 and 24 h post infection respectively).

Experimental data used for parameter estimation (A1, B1, C1, D1, E1, F1, G1). The grey lines indicate the limit of quantification for each metabolite and the grey data points are under the limit of quantification.

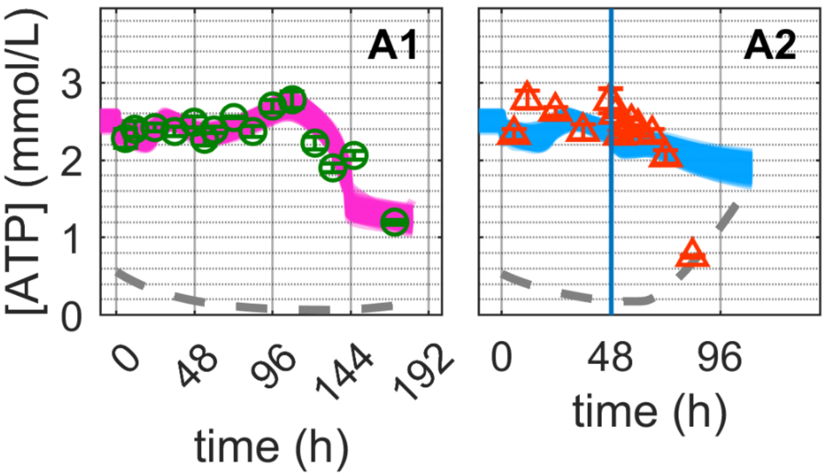


**Figure S2F. Results of 2500 simulations using the parameters obtained via a bootstrap method for the model established for mock-infected and infected suspension MDCK cells.** (A1–2) Adenosine tri-phosphate. Data and error bars represent the mean and standard deviation of technical triplicates for two independent experiments (mock-infected Θ and infected Δ). Light Blue and pink patches: Lines corresponding to 2500 model simulations (thicker during towards end of cultivation due to higher sensibility of parameters related to cell death). Vertical blue lines correspond to (0, 12 and 24 h post infection respectively). Experimental data used for parameter estimation (A1). The grey lines indicate the limit of quantification for each metabolite and the grey data points are under the limit of quantification.
