# Supplementary material for: Impact of Influenza A Virus Infection on Growth and Metabolism of Suspension MDCK Cells Using a Dynamic Model
Source: Metabolites. 2022 Mar 12;12(3):239. doi: 10.3390/metabo12030239 (PMC8950586; doi:10.3390/metabo12030239)
Supplement: Supplementary file 1 [file metabolites-12-00239-s001.zip › Figure S3.pdf]

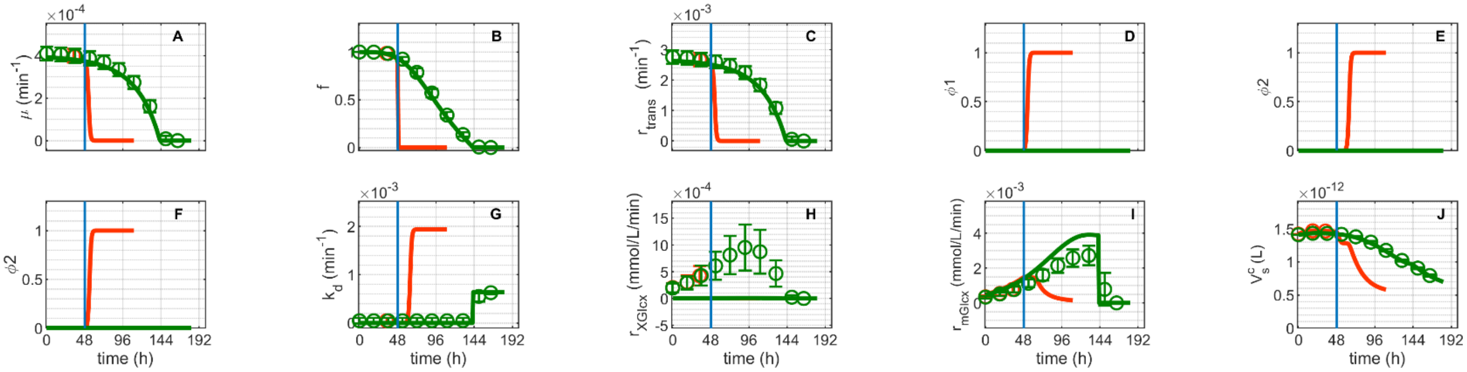

Figure S3A. Simulated rates and standard deviations for Cultivation 1 and 2 (mock-infected —, infected —) calculated from 2500 simulations via a bootstrap method. (A) Growth rate, (B) inhibition factor, (C) transition rate, (D, E, F) step functions Φ_{1-3} (G) cell death rate, (H) growth-related glucose consumption rate, (I) maintenance-related glucose consumption and (J) cell-specific volume. Data and error bars represent the mean and standard deviation of the rates at each time point based on 2500 model simulations. Vertical blue line: 0 h post infection for Cultivation 2. Mean and standard deviation was not calculated for growth-related parameters after infection since they were fixed (the continuous red line represents only one simulation). ○: mock-infected and ○: infected.

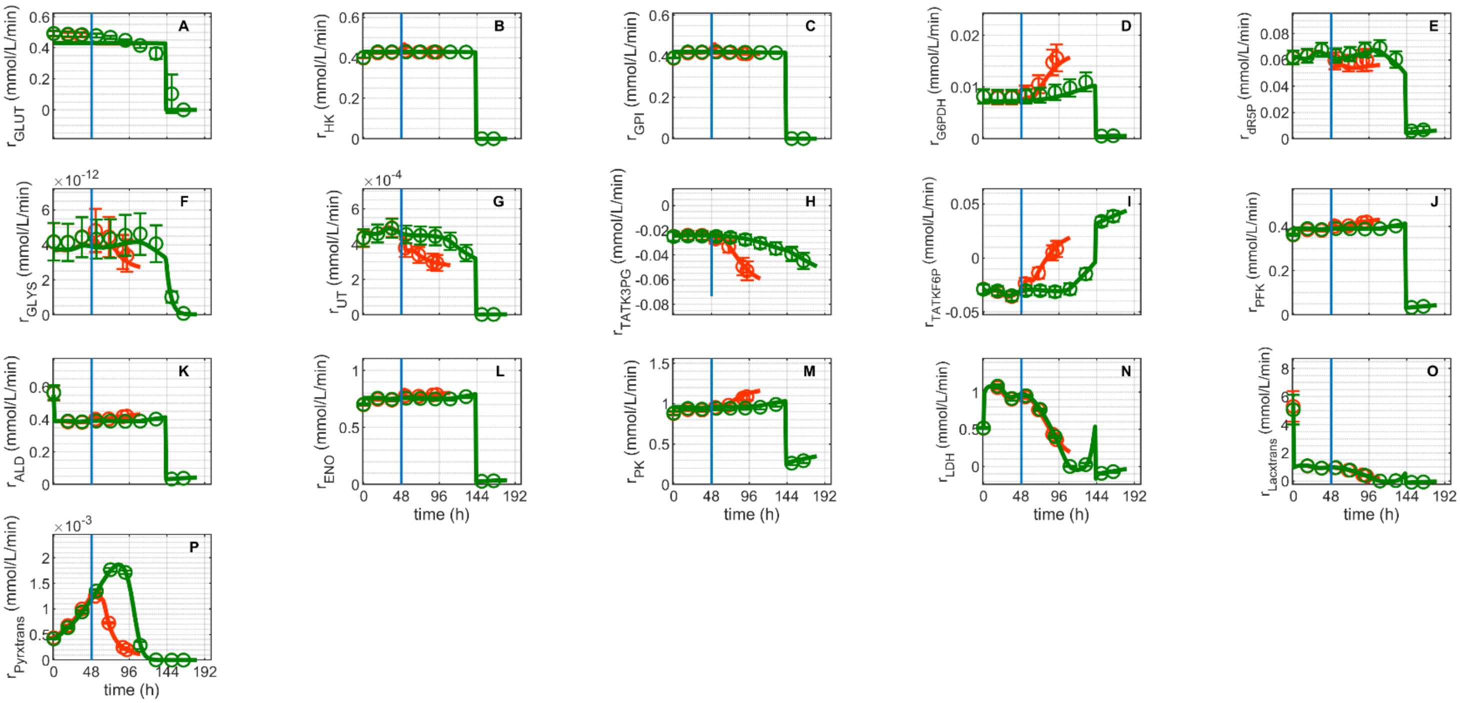

Figure S3B. Simulated rates and standard deviations for Cultivation 1 and 2 (mock-infected —, infected —) calculated from 2500 simulations via a bootstrap method. (A) Glucose transporter rate, (B) hexokinase rate, (C) glucose-6-phosphate isomerase rate, (D) glucose-6-phosphate dehydrogenase rate, (E) ribose-5-phosphate consumption rate, (F) glycogen synthetase rate, (G) uridyl transferase rate, (H) transaldolase and transketolase rate, (I) transaldolase and transketolase rate, (J) phosphofructokinase rate, (K) aldolase rate, (L) enolase rate, (M) pyruvate kinase rate, (N) lactate dehydrogenase rate, (O) extracellular lactate production/ consumption rate and (P) extracellular pyruvate consumption rate. Data and error bars represent the mean and standard deviation of the rates at each time point based on 2500 model simulations. Vertical blue line: 0 h post infection for Cultivation 2. ○: mock-infected and ○: infected.

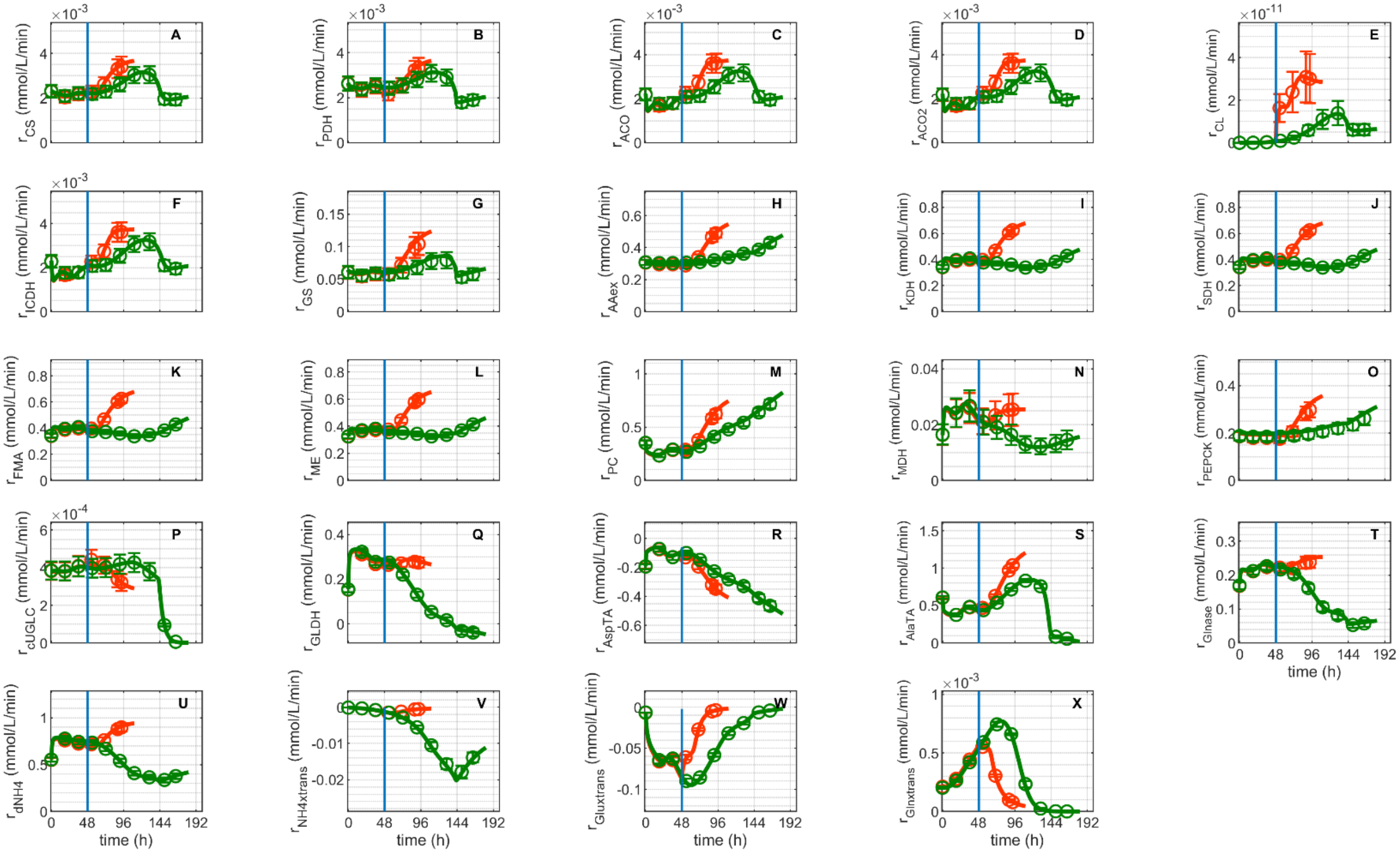

Figure S3C. Simulated rates and standard deviations for Cultivation 1 and 2 (mock-infected —, infected —) calculated from 2500 simulations via a bootstrap method. (A) Citrate synthetase rate, (B) pyruvate dehydrogenase rate, (C) aconitase rate, (D) aconitase rate, (E) citrate lyase rate, (F) isocitrate dehydrogenase rate, (G) glutamine synthetase rate, (H) amino acids degradation rate, (I) ketoglutarate dehydrogenase rate, (J) succinate dehydrogenase rate, (K) fumarase rate, (L) malic enzyme rate, (M) pyruvate carboxylase rate, (N) malate dehydrogenase rate, (O) phosphoenolpyruvate-kinase rate, (P) uridine diphosphate glucose consumption rate, (Q) glutamate dehydrogenase rate, (R) aspartate transaminase rate, (S) alanine transaminase rate, (T) glutaminase rate, (U) ammonium consumption rate, (V) extracellular ammonium production rate, (W) extracellular glutamate production rate and (X) extracellular glutamine consumption rate. Data and error bars represent the mean and standard deviation of the rates at each time point based on 2500 model simulations. Vertical blue line: 0 h post infection for Cultivation 2. ○: mock-infected and ○: infected.

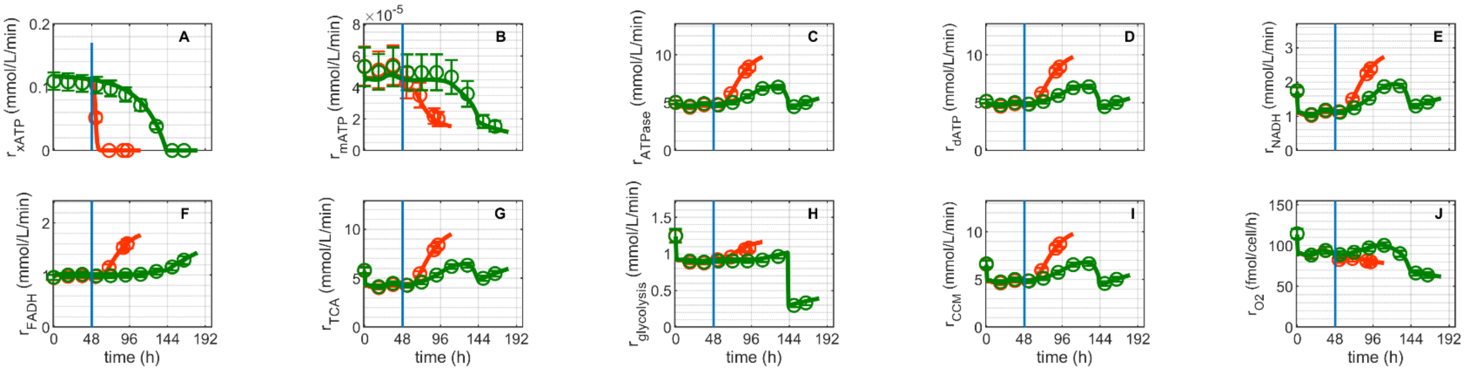

Figure S3D. Simulated rates and standard deviations for Cultivation 1 and 2 (mock-infected —, infected —) calculated from 2500 simulations via a bootstrap method. (A) ATP consumption for growth, (B) ATP consumption for maintenance, (C) ATPase rate, (D) net consumption of ATP, (E) NADH oxidative phosphorylation rate, (F) FADH oxidative phosphorylation rate, (G) TCA net ATP production rate, (H) glycolytic net ATP production rate, (I) net production of ATP and (J) theoretical oxygen consumption rate. Data and error bars represent the mean and standard deviation of the rates at each time point based on 2500 model simulations. Vertical blue line: 0 h post infection for Cultivation 2. ○: mock-infected and ○: infected.
