# Supplementary material for: Impact of Influenza A Virus Infection on Growth and Metabolism of Suspension MDCK Cells Using a Dynamic Model
Source: Metabolites. 2022 Mar 12;12(3):239. doi: 10.3390/metabo12030239 (PMC8950586; doi:10.3390/metabo12030239)
Supplement: Supplementary file 1 [file metabolites-12-00239-s001.zip › Figure S4.pdf]

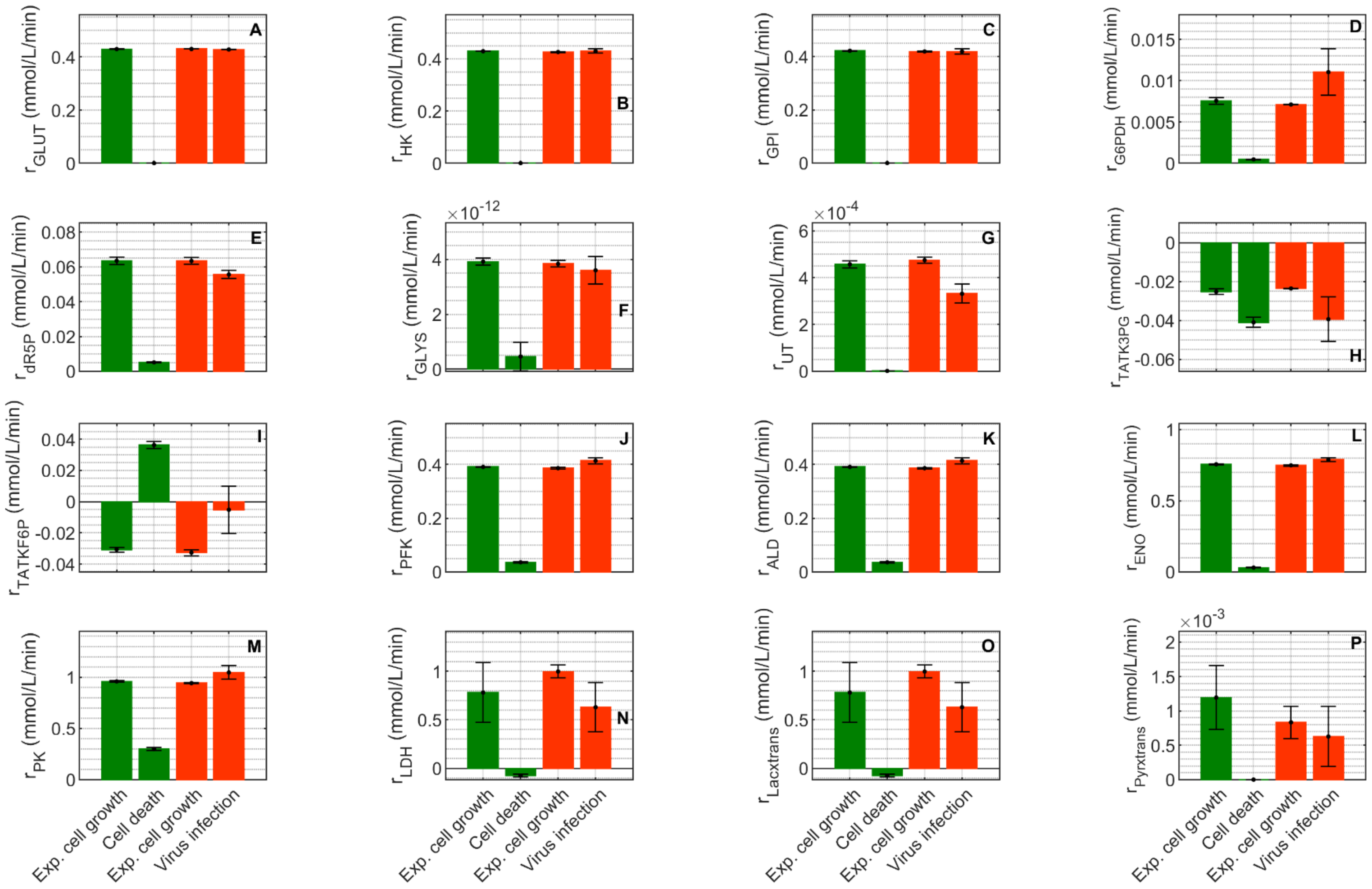

Figure S4A. Box plots with average and standard deviation of intracellular rates estimated from model simulations for mock-infected and infected suspension MDCK cells. (A) Glucose transporter rate, (B) hexokinase rate, (C) glucose-6-phosphate isomerase rate, (D) glucose-6-phosphate dehydrogenase rate, (E) ribose-5-phosphate consumption rate, (F) glycogen synthetase rate, (G) uridyl transferase rate, (H) transaldolase and transketolase rate, (I) transaldolase and transketolase rate, (J) phosphofructokinase rate, (K) aldolase rate, (L) enolase rate, (M) pyruvate kinase rate, (N) lactate dehydrogenase rate, (O) extracellular lactate production/ consumption rate and (P) extracellular pyruvate consumption rate. Calculated from the exponential growth phase of Cultivation 1 (□, 6–108 h), death phase of Cultivation 1 (□, 146–169 h), Exponential growth phase of Cultivation 2 (□, 6–48 h) and during virus replication of Cultivation 2 (□, 49.9–107 h). Error bars represent the mean and standard deviation of the rates for the time interval of the corresponding cultivation phase. The white columns represent the maximum rates for comparison. Note that some rates are negative since some reactions are reversible.

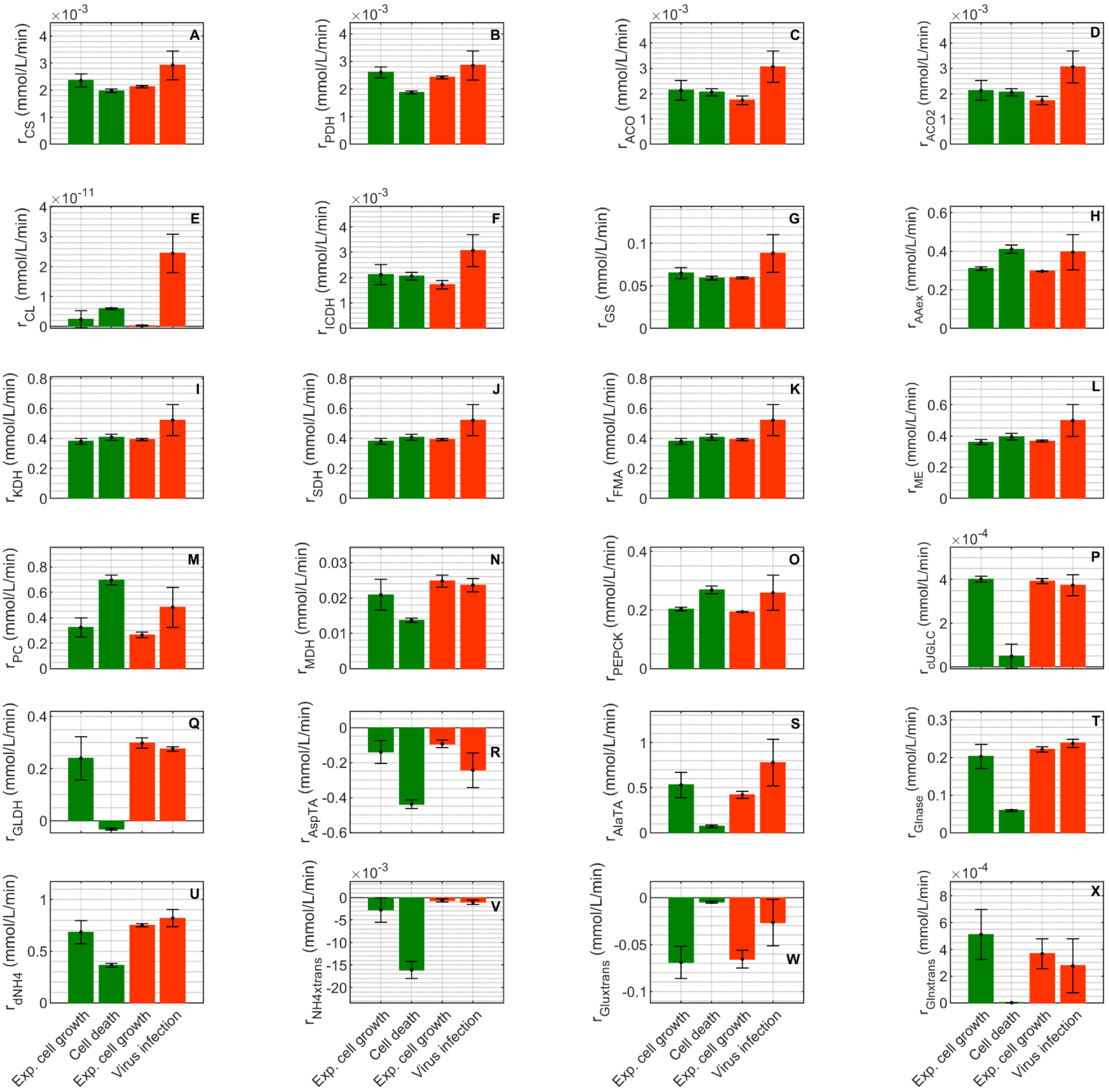

Figure S4B. Box plots with average and standard deviation of intracellular rates estimated from model simulations for mock-infected and infected suspension MDCK cells. (A) Citrate synthetase rate, (B) pyruvate dehydrogenase rate, (C) aconitase rate, (D) aconitase rate, (E) citrate lyase rate, (F) isocitrate dehydrogenase rate, (G) glutamine

synthetase rate, (H) amino acids degradation rate, (I) ketoglutarate dehydrogenase rate, (J) succinate dehydrogenase rate, (K) fumarase rate, (L) malic enzyme rate, (M) pyruvate carboxylase rate, (N) malate dehydrogenase rate, (O) phosphoenolpyruvate-kinase rate, (P) uridine diphosphate glucose consumption rate, (Q) glutamate dehydrogenase rate, (R) aspartate transaminase rate, (S) alanine transaminase rate, (T) glutaminase rate, (U) ammonium consumption rate, (V) extracellular ammonium production rate, (W) extracellular glutamate production rate and (X) extracellular glutamine consumption rate. Calculated from the exponential growth phase of Cultivation 1 (□, 6–108 h), death phase of Cultivation 1 (□, 146–169 h), exponential growth phase of Cultivation 2 (□, 6–48 h) and during virus replication of Cultivation 2 (□, 49.9–107 h). Error bars represent the mean and standard deviation of the rates for the time interval of the corresponding cultivation phase. The white columns represent the maximum rates for comparison. Note that some rates are negative since some reactions are reversible.

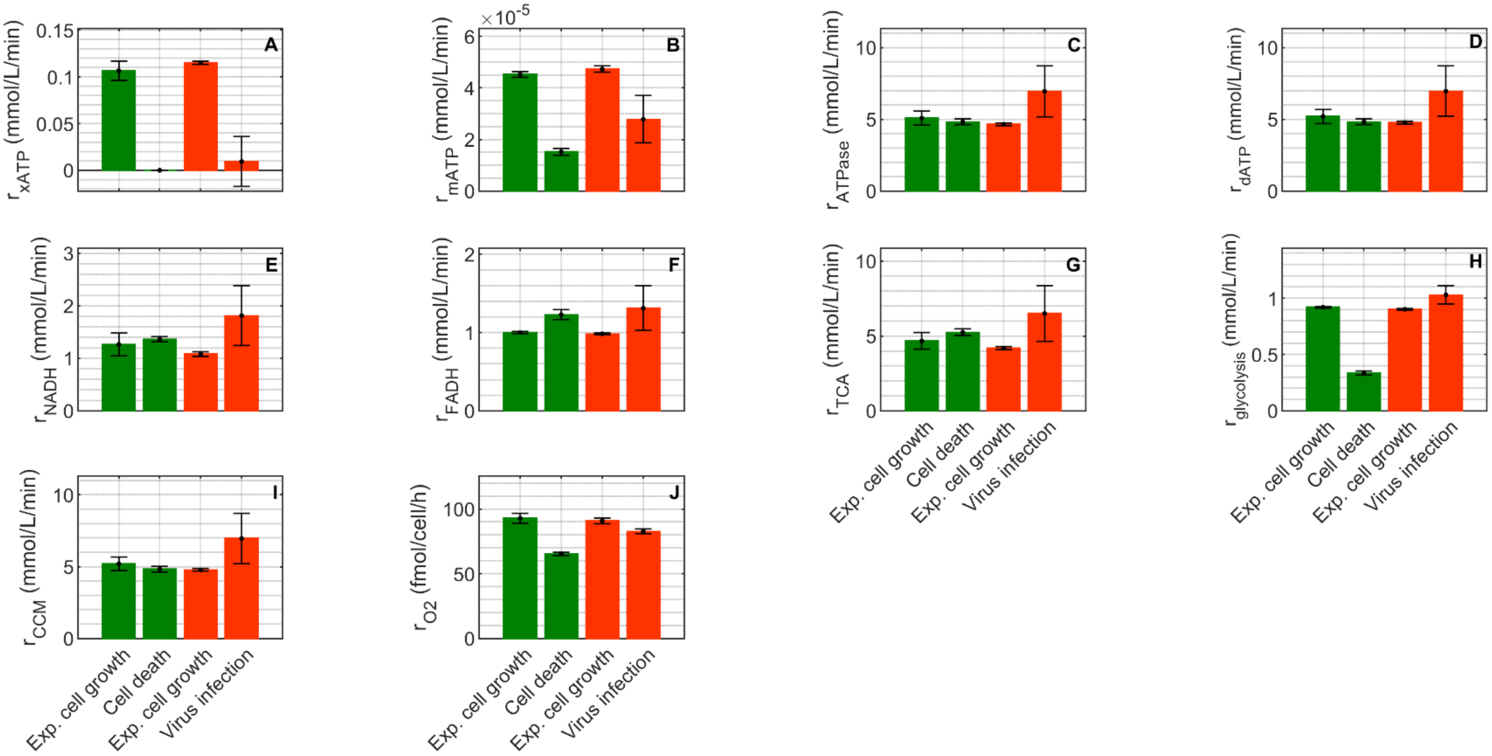

Figure S4C. Box plot with averages and standard deviation of intracellular rates estimated from model simulations for mock-infected and infected suspension MDCK cells. (A) ATP consumption for growth, (B) ATP consumption for maintenance, (C) ATPase rate, (D) net consumption of ATP, (E) NADH oxidative phosphorylation rate, (F) FADH oxidative phosphorylation rate, (G) TCA net ATP production rate, (H) glycolytic net ATP production rate, (I) net production of ATP and (J) theoretical oxygen consumption rate. Calculated from the exponential growth phase of Cultivation 1 (□, 6–108h), death phase of

Cultivation 1 (□, 146–169h), Exponential growth phase of cultivation 2 (□, 6–48h) and during virus replication cultivation 2 (□, 49.9–107h). Error bars represent the mean and standard deviation of the rates for the time interval of the corresponding cultivation phase. The white columns represent the maximum rates for comparison. Note that some rates are negative since some reactions are reversible.
