# Supplementary material for: Impact of Influenza A Virus Infection on Growth and Metabolism of Suspension MDCK Cells Using a Dynamic Model
Source: Metabolites. 2022 Mar 12;12(3):239. doi: 10.3390/metabo12030239 (PMC8950586; doi:10.3390/metabo12030239)
Supplement: Supplementary file 1 [file metabolites-12-00239-s001.zip › Figure S5.pdf]

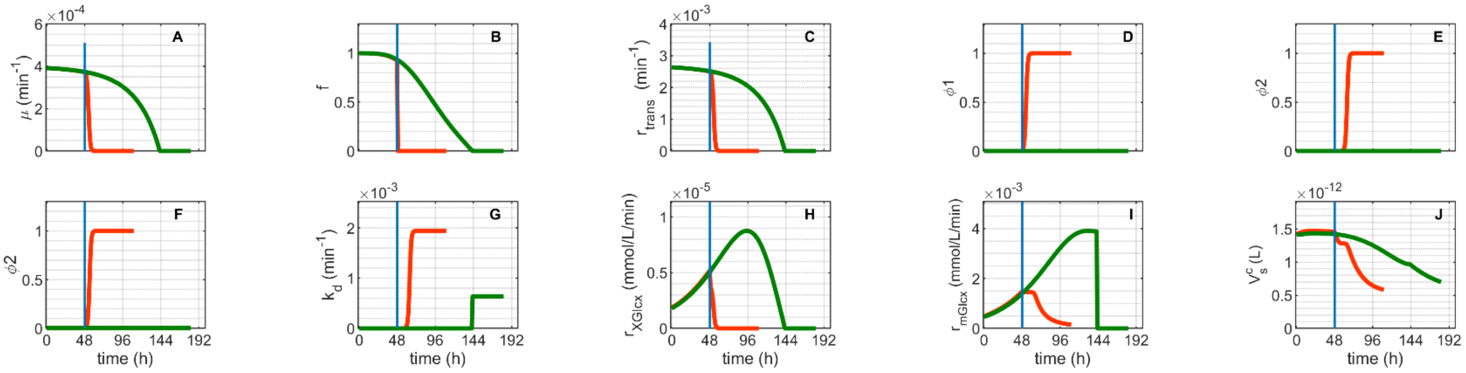

Figure S5A. Simulated rates for Cultivation 1 and 2 (mock-infected —, infected —). (A) Growth rate, (B) inhibition factor, (C) transition rate, (D, E, F) step function Φ_{1-3}, (G) cell death rate, (H) growth related glucose consumption rate, (I) maintenance-related glucose consumption and (J) cell-specific volume.

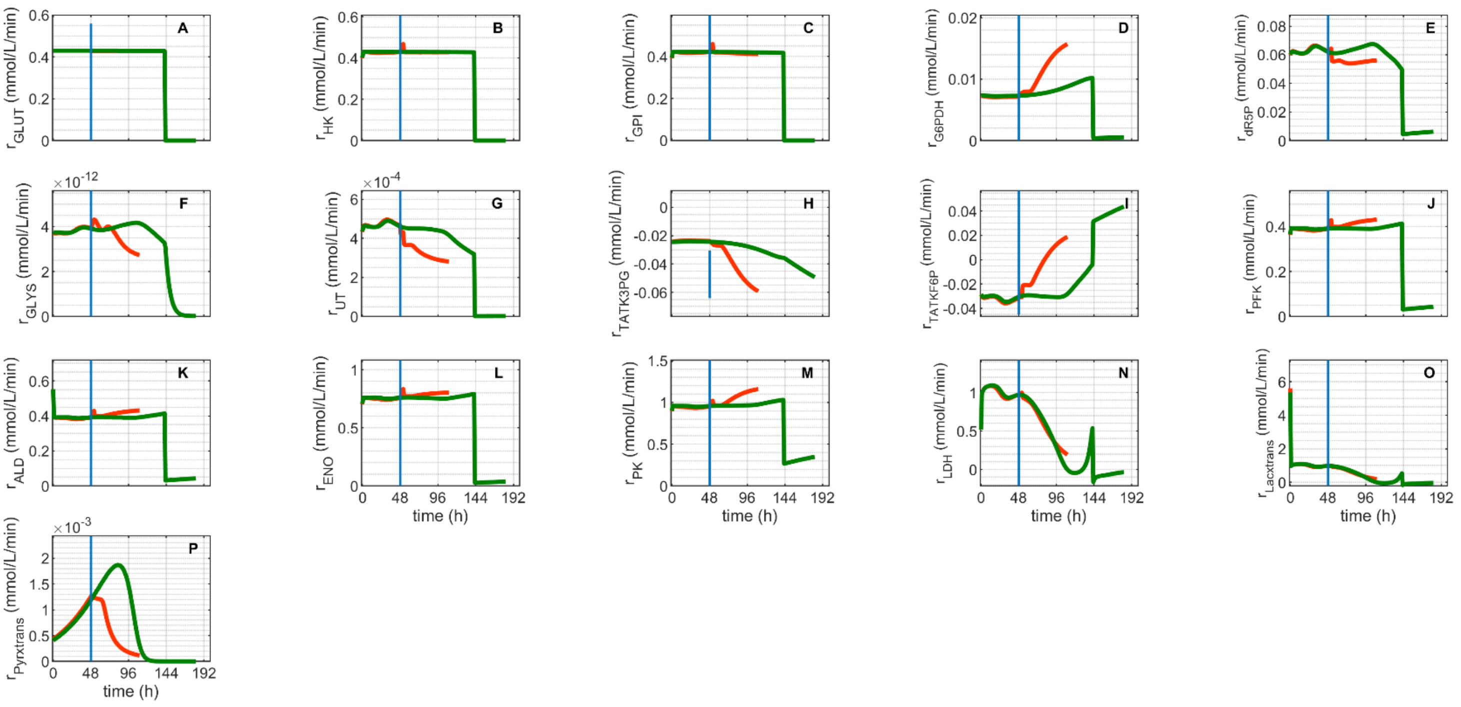

Figure S5B. Simulated rates for Cultivation 1 and 2 (mock-infected —, infected —). (A) Glucose transporter rate, (B) hexokinase rate, (C) glucose-6-phosphate isomerase rate, (D) glucose-6-phosphate dehydrogenase rate, (E) ribose-5-phosphate consumption rate, (F) glycogen synthetase rate, (G) uridyl transferase rate, (H) transaldolase and transketolase rate, (I) transaldolase and transketolase rate, (J) phosphofructokinase rate, (K) aldolase rate, (L) enolase rate, (M) pyruvate kinase rate, (N) lactate dehydrogenase rate, (O) extracellular

lactate production/ consumption rate and (P) extracellular pyruvate consumption rate. Vertical blue line represents 0 h post infection for Cultivation 2.

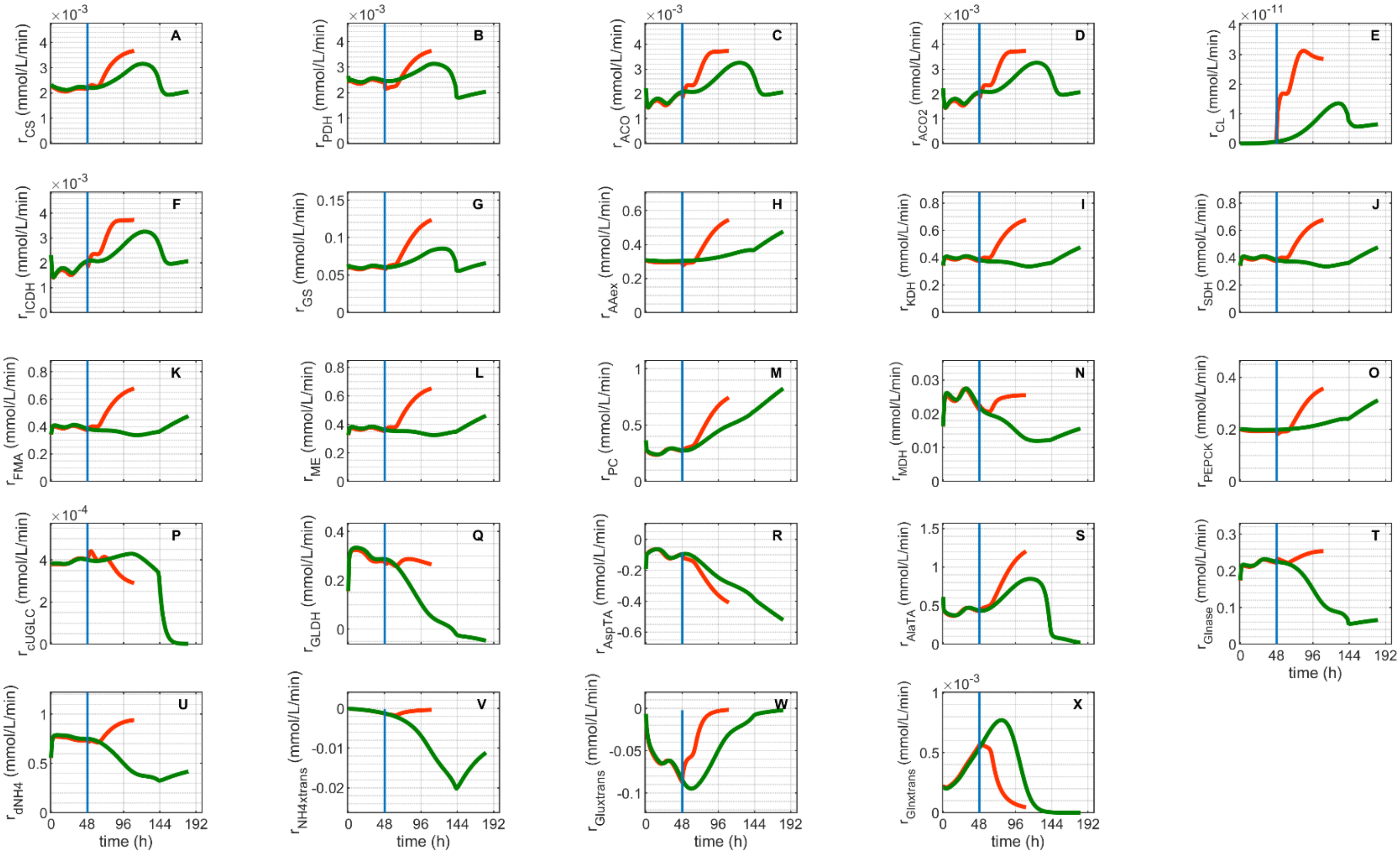

Figure S5C. Simulated rates for Cultivation 1 and 2 (mock-infected —, infected —). (A) Citrate synthetase rate, (B) : pyruvate dehydrogenase rate, (C) aconitase rate, (D) aconitase rate, (E) citrate lyase rate, (F) isocitrate dehydrogenase rate, (G) glutamine synthetase rate, (H) amino acids degradation rate, (I) ketoglutarate dehydrogenase rate, (J) succinate dehydrogenase rate, (K) fumarase rate, (L) malic enzyme rate, (M) pyruvate carboxylase rate, (N) malate dehydrogenase rate, (O) phosphoenolpyruvate-kinase rate, (P) uridine diphosphate glucose consumption rate, (Q) glutamate dehydrogenase rate, (R) aspartate transaminase rate, (S) alanine transaminase rate, (T) glutaminase rate, (U) ammonium consumption rate, (V) extracellular ammonium production rate, (W) extracellular glutamate production rate and (X) extracellular glutamine consumption rate. Vertical blue line represents 0 h post infection for Cultivation 2.

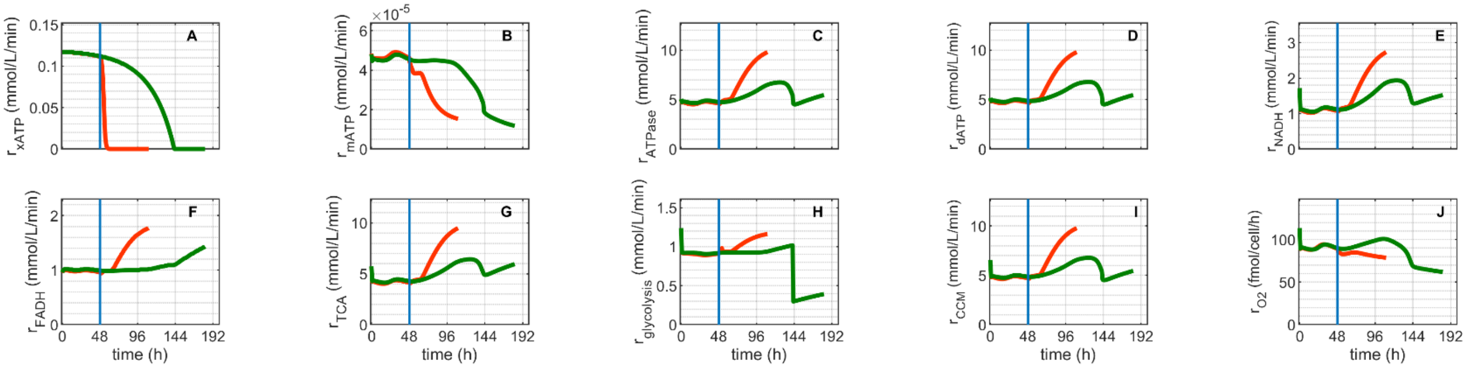

Figure S5D. Simulated rates for Cultivation 1 and 2 (mock-infected —, infected —). (A) ATP consumption for growth, (B) ATP consumption for maintenance, (C) ATPase rate, (D) net consumption of ATP, (E) NADH oxidative phosphorylation rate, (F) FADH oxidative phosphorylation rate, (G) TCA net ATP production rate, (H) glycolytic net ATP production rate, (I) net production of ATP and (J) theoretical oxygen consumption rate. Vertical blue line represents 0 h post infection for Cultivation 2.
