# Supplementary material for: Impact of Influenza A Virus Infection on Growth and Metabolism of Suspension MDCK Cells Using a Dynamic Model
Source: Metabolites. 2022 Mar 12;12(3):239. doi: 10.3390/metabo12030239 (PMC8950586; doi:10.3390/metabo12030239)
Supplement: Supplementary file 1 [file metabolites-12-00239-s001.zip › Supplementary File S1.pdf]

João R. C. Ramos, Thomas Bissinger, Yvonne Genzel, Udo Reichl, Impact of influenza A virus infection on growth and metabolism of suspension MDCK cells using a dynamic model

Model definition and equations supporting information

Contents

1 Model definition 2

1.1 Segregated cell growth and infection model 3

1.2 Structured model of the central carbon metabolism 5

1.2.1 Glycolysis 5

1.2.2 Citric acid cycle, glutaminolysis and transamination 7

1.2.3 Energy metabolism 8

2 Equations for the rates used in the segregated cell growth model and structured model of the intracellular metabolism 9

2.1 Rates of the segregated cell growth model 9

2.2 Rates of the structured model of the intracellular metabolism 10

2.2.1 Rates from glycolysis 10

2.2.2 Rates from glycolysis, TCA and other pathways. 12

2.2.3 Rates from TCA 12

2.2.3 Rates from energy production 14

3 Supplementary references 15

1 Model definition

Using the same model variables at the macro- and microscopic scale considered in this approach requires a conversion function, which provides a link between rates on the macroscopic scale (r_{macro}, mmol/min/L referring to the working volume of the shaker) and the corresponding rates at the microscopic scale r_i (mmol/min/L referring to the volume of cells). Where V_s^c is cell-specific volume (L/cell, Equation (9)) and X_v is the viable cell concentration (cells/mL, Equation (6)). In order to convert the viable cell volume per mL to the viable cell volume per L, a conversion factor (V_w, 10^{-3}) was used.

$$r_{macro} = r_i \frac{V_s^c X_v}{V_w} \tag{1}$$

The cell-specific enzyme activities were expressed per cell (v_e, mmol/cell/min), and because the average cell volume changes during cultivation, the maximum volumetric enzyme activities change as well (Equation (2)).

$$K_e^{\max} = \frac{v_e E_{level}}{V_c^s} \tag{2}$$

Where $K_e^{\max}$ denotes the maximum volumetric enzyme activities (mmol/L/min) and are dependent on their related cell-specific enzyme activity (v_e, mmol/cell/min), the enzyme level (E_{level}, constant) and the cell-specific volume (V_c^s, L/cell). The enzyme level (E_{level}) is a term that was previously proposed for MDCK cells [1] and corresponds to the experiment-specific relative enzyme level of the cell population, which indicates that total enzyme content varies between

experiments. Here, since the experiments were conducted using the same pre-culture, the enzyme level was set to one for both cultivations.

1.1 Segregated cell growth and infection model

The segregated cell growth model approaches allow the consideration of several classes of cells. Similar to previous approaches [2,3], five classes ($N^c = 5$) were used to describe changes in the mean cell diameter of MDCK cells. Because a segregated cell growth model enables the estimation of the mean cell diameter, it also allows the estimation of the total viable cell volume. Estimating the cell volume was critical for determining the cell-specific volume, which impacts the maximum volumetric enzyme activities (Equation (2)).

The model describes the cell transition between each cell class using a transition rate (r_{trans} , Equation (3), main manuscript), starting with the class (X_1) which contains the smallest cells and ending with the class (X_5) containing the largest cells. The latter divide and produce two cells of the first class (Equations (3)–(5)). Further analyses and discussions regarding the choice of cell classes can be found in section 1 in S6 File and additional aspects regarding the choice of classes have been also discussed elsewhere [1–4].

$$\frac{dX_1}{dt} = r_{trans}(2\mathrm{X}_5 - X_1 f) - k_d X_1, \tag{3}$$

$$\frac{dX_2}{dt} = r_{trans}(X_1 f - X_2) - k_d X_2, \tag{4}$$

$$\frac{dX_i}{dt} = r_{trans}(X_{i-1} - X_i) - k_d X_i, \text{ for } \mathrm{i} = 3, ..., \mathrm{N}^c. \tag{5}$$

Given the different cell classes introduced (Equations (3)–(5)), the viable cell concentration (X_v) was calculated as the sum of cells in each class (Equation (6)).

$$X_v = \sum_{i=1}^{N^c} X_i \qquad (6)$$

The mean cell diameter ($\overline{d}$) was estimated using Equation (7), in which the number of cells of each class (X_i) and their respective diameters is taken into account. Here, the diameter of cells in each class are equidistant ranging from a minimum value (d_m , for the smallest cells found in the first class X_1) to a critical diameter value (d_c , for the largest cells found in class X_{N^c}). Note that both d_c and d_m were manually adjusted for each experiment.

$$\overline{d} = \sum_{i=1}^{N^c} \left(d_m + \frac{d_c - d_m}{N^c - 1}(i-1) \right) \frac{X_i}{X_v} \qquad (7)$$

Using the mean diameter ($\overline{d}$), the viable cell volume (V^c , μL) and the cell-specific volume (V_s^c , L/cell) were calculated using Equations (8) and (9), respectively.

$$V^c = \pi \frac{\overline{d}^3}{6} X_v 10^{-9} \qquad (8)$$

$$V_s^c = \frac{V^c}{X_v} 10^{-6} \qquad (9)$$

Substrates and dynamics of released metabolic by-products are also part of the macroscopic model. These include extracellular glucose, glutamine, glutamate, lactate and ammonium. The glucose dynamics was described using cell growth-related and maintenance terms. The remaining extracellular metabolites were described using a Michaelis-Menten-like kinetic or more complex equations in accordance with previous studies describing their transport through the cell membrane

[2,5–10]. In particular, these metabolites were produced and/or consumed independently from the cell growth rate and cell growth occurs even after depletion in some cases. The spontaneous degradation of extracellular glutamine to ammonium was also taken into account, as reported in [11]. Equations (10)–(15) introduce the ODEs associated with the extracellular metabolites and the transport kinetics in these ODEs, chosen based on previous literature [1–4,12], were provided later in section 2 below.

$$\frac{d[Glc^x]}{dt} = -r_{X/Glc^x} - r_{m/Glc^x} \tag{10}$$

$$\frac{d[Gln^x]}{dt} = -r_{Gln^x{}_{trans}} - r_{dGln^x} \tag{11}$$

$$\frac{d[Glu^x]}{dt} = -r_{Glu_{trans}} \frac{V_s^c X_v}{V_w} \tag{12}$$

$$\frac{d[\mathrm{NH4}^x]}{dt} = r_{dGln^x} - r_{\mathrm{NH4}^x_{trans}} \frac{V_s^c X_v}{V_w} \tag{13}$$

$$\frac{d\left[Lac^x\right]}{dt} = r_{Lac^x_{trans}} \frac{V_s^c X_v}{V_w} \tag{14}$$

$$\frac{d[Pyr^x]}{dt} = -r_{Pyr^x{}_{Trans}} \tag{15}$$

1.2 Structured model of the central carbon metabolism

1.2.1 Glycolysis

Glycolysis and pentose phosphate pathway were described using the ODEs in Equations (16)–(25) in accordance with the structure and basic assumptions for modeling MDCK cells [1]

and other animal cell lines [2]. For each intracellular metabolite (C), the consumption or production by individual enzymes was considered. The enzyme and transport kinetics in these ODEs, chosen based on previous literature [1–4,12], were provided later in section 2 below. Additionally, the transport of glucose (r_{GLUT}, Equation (47)), pyruvate ($r_{Pyr^x_{Trans}}$, Equation (46)) and lactate ($r_{Lac_{trans}}$, Equation (45)) were considered, linking the extra- and intracellular forms of these metabolites. The dilution of each intracellular metabolite due to changes in the cell volume caused by cell growth was taken into account by using the term $\mu f[C]$.

$$\frac{d[Glc]}{dt} = r_{\mathrm{GLUT}} - r_{HK} - \mu f[Glc] \tag{16}$$

$$\frac{d[G6P]}{dt} = r_{HK} - r_{GPI} - r_{G6PDH} - r_{UT} - \mu f[\mathrm{G6P}] \tag{17}$$

$$\frac{d[\mathrm{F}6P]}{dt} = r_{GPI} - r_{PFK} + r_{TATKF6P} - \mu f[\mathrm{F6P}] \tag{18}$$

$$\frac{d[R5P]}{dt} = r_{G6PDH} - r_{TATKF6P} - r_{TATK3PG} - r_{dR5P} - \mu f[\mathrm{R5P}] \tag{19}$$

$$\frac{d[UDPGlc]}{dt} = r_{UT} - r_{cUGlC} - r_{GLYS} - \mu f[\mathrm{UDPGlc}] \tag{20}$$

$$\frac{d[F16P]}{dt} = r_{PFK} - r_{ALD} - \mu f[\mathrm{F16P}] \tag{21}$$

$$\frac{d[\mathrm{3PG}]}{dt} = 2r_{ALD} - r_{ENO} + r_{TATK3PG} - \mu f[3PG] \tag{22}$$

$$\frac{d[PEP]}{dt} = r_{ENO} - r_{PK} - r_{PEPCK} - \mu f[\mathrm{PEP}] \tag{23}$$

$$\frac{d[Pyr]}{dt} = r_{PK} - r_{LDH} - r_{PDH} + r_{ME} - r_{PC} + r_{Pyr^x_{Trans}} \frac{V_w}{\left(V_s^c X_v\right)} - r_{AlaTA} - \mu f[\mathrm{Pyr}] \tag{24}$$

$$\frac{[Lac]}{dt} = r_{LDH} - r_{Lac_{trans}} - \mu f[Lac] \tag{25}$$

1.2.2 Citric acid cycle, glutaminolysis and transamination

Following the structure and basic assumptions from previous work [2], Equations (26)–(37) were used to describe citric acid cycle (TCA), glutaminolysis and transamination. For each ODE, the individual enzyme and transport kinetics, chosen based on previous literature [1–4,12], were provided later in section 2 below. Additionally, the transport of glutamine ($r_{Gln^x_{trans}}$, Equation (41)), glutamate ($r_{GLU_{trans}}$, Equation (43)) and ammonium ($r_{\mathrm{NH4}^x_{trans}}$, Equation (44)) were considered, linking the extra- and intracellular forms of these metabolites. The dilution of each intracellular metabolite due to changes in the cell volume caused by cell growth was taken into account by using the term $\mu f[C]$.

$$\frac{d[AcCoA]}{dt} = r_{PDH} - r_{CS} - \mu f[AcCoA] \tag{26}$$

$$\frac{d[Cit]}{dt} = r_{CS} - r_{ACO} - r_{CL} - \mu f[Cit] \tag{27}$$

$$\frac{d[cAc]}{dt} = r_{ACO} - r_{ACO2} - \mu f[cAc] \tag{28}$$

$$\frac{d[Isocit]}{dt} = r_{ACO2} - r_{ICDH} - \mu f[Isocit] \tag{29}$$

$$\frac{d[Gln]}{dt} = r_{Gln^x_{trans}} \frac{V_w}{\left(V_s^c X_v\right)} - r_{Glnase} + r_{GS} - \mu f[Gln] \tag{30}$$

$$\frac{d[Glu]}{dt} = r_{GLU_{trans}} + r_{Glnase} + r_{AAex} - r_{GS} - r_{GLDH} + r_{AspTA} - \mu f[\mathrm{Glu}] \tag{31}$$

$$\frac{d[NH4]}{dt} = r_{\mathrm{NH4}^{x}_{trans}} + (1+\omega) r_{AAex} + r_{GLNase} + r_{GL\mathrm{DH}} - r_{d\mathrm{NH4}} - r_{GS} - \mu f [NH4] \tag{32}$$

$$\frac{d[\mathrm{Keto}]}{dt} = r_{ICDH} - r_{KDH} + r_{GLDH} - r_{AspTA} - \mu f [\mathrm{Keto}] \tag{33}$$

$$\frac{d[\mathrm{Suc}]}{dt} = r_{KDH} - r_{SDH} - \mu f [\mathrm{Suc}] \tag{34}$$

$$\frac{d[\mathrm{Fum}]}{dt} = r_{SDH} - r_{FMA} - \mu f [\mathrm{Fum}] \tag{35}$$

$$\frac{d[\mathrm{Mal}]}{dt} = r_{FMA} - r_{MDH} - r_{ME} - \mu f [\mathrm{Mal}] \tag{36}$$

$$\frac{d[\mathrm{OAA}]}{dt} = r_{MDH} + r_{AspTA} + r_{CL} + r_{PC} - r_{PEPCK} - r_{CS} - \mu f [\mathrm{OAA}] \tag{37}$$

1.2.3 Energy metabolism

Following the structure and basic assumptions from previous work [2], the ODE in Equation (38) was used to describe ATP dynamics. ATP production was lumped into one rate (r_{CCM}), where 2.5 ATP are produced from NADH and 1.5 ATP from $FADH_2$ as reported in [13,14]. ATP usage was lumped into a degradation term (r_{dATP}), which contains the terms for the estimated consumption for cell growth and maintenance. It was assumed that energy precursors do not accumulate but are used directly in the oxidative phosphorylation (electron transport) pathway. Furthermore, due to the lack of experimental data, the impact of cofactors on enzyme regulation and homeostasis was not taken into account. Further details for both reactions (r_{CCM} and r_{dATP}) were provided later in section 2.2.3 below.

$$\frac{d[\mathrm{ATP}]}{dt} = r_{CCM} - r_{dATP} - \mu f[\mathrm{ATP}] \tag{38}$$

2 Equations for the rates used in the segregated cell growth model and structured model of the intracellular metabolism

2.1 Rates of the segregated cell growth model

$$r_{x/\mathrm{Glc}^x} = \mu\left(X_1 f + \sum_{i=2}^{N^c} X_i\right) Y_{x/Glc^x} \tag{39}$$

$$r_{m/Glc^x} = m_{Glc^x} V^c \Theta\left[Glc^x\right] \tag{40}$$

$$r_{Gln^x_{trans}} = v_{Gln^x_{trans}} \frac{[Gln^x]}{1+\left(\frac{[Gln]}{k_{Gln^x}}\right)^3 + [Gln^x]} V^c \tag{41}$$

$$r_{dGln^x} = k_{dGln^x}[Gln^x] \tag{42}$$

$$r_{Glu^x_{trans}} = v_{Glu^x_{trans}} \frac{\left(\frac{[Glu^x]}{k_{Glu^x}}\right)^2 \left(1-\frac{[Glu]}{[Glu^x]k^{eq}_{Glu^x_{trans}}}\right)}{1+\left(\frac{[Glu^x]}{k_{Glu^x}}\right)^2 + \left(\frac{[Glu]}{k_{Glu}}\right)^2} V^c \tag{43}$$

$$r_{\mathrm{NH4}^x_{trans}} = v_{\mathrm{NH4}_{trans}} \frac{\left(\frac{[NH4^x]}{k_{\mathrm{NH4}^x}}\right)^2 \left(1-\frac{[NH4]}{[NH4^x]k^{eq}_{\mathrm{NH4}^x_{trans}}}\right)}{1+\left(\frac{[Glu^x]}{k_{\mathrm{NH4}^x}}\right)^2 + \left(\frac{[Glu]}{k_{\mathrm{NH4}}}\right)^2} V^c \tag{44}$$

$$r_{Lac^x_{trans}} = v_{Lac^x_{trans}} \frac{\left(1-\frac{\frac{\left[Lac^x\right]}{\left[Lac\right]}}{k^{eq}_{Lac^x_{trans}}}\right)\left(\frac{\left[Lac\right]}{k_{Lac}}+\frac{\left[Lacx\right]}{k_{Lac^x}}\right)^3}{1+\left(\frac{\left[Lac\right]}{k_{Lac}}+\frac{\left[Lacx\right]}{k_{Lac^x}}\right)^4} V^c \tag{45}$$

$$r_{Pyr^x{}_{trans}} = v_{Pyr^x{}_{trans}} \frac{[Pyr^x]}{1+[Pyr^x]+k^m_{Pyr^x{}_{trans}}} V^c \tag{46}$$

2.2 Rates of the structured model of the intracellular metabolism

2.2.1 Rates from glycolysis

$$r_{\mathrm{GLUT}} = \left(r_{X/Glc^x} + r_{m/Glc^x}\right)\frac{V_w}{V^c_s X_v} \tag{47}$$

$$r_{HK} = K^{\max}_{HK} \frac{\left[Glc\right][ATP]}{\left[Glc\right][ATP]+k^m_{HK}[\mathrm{Glc}]+\mathrm{k}^m_{ATP_{HK}}[Glc]+\mathrm{k}^m_{ATP_{HK}}\, k^m_{HK}} \tag{48}$$

$$r_{GPI} = K^{\max}_{GPI} \frac{\left(\left[G6P\right]-\frac{\left[F6P\right]}{k^{eq}_{GPI}}\right)}{k^m_{GPI}+\left[G6P\right]+\frac{\left[F6P\right]}{k^{eq}_{GPI}}} \tag{49}$$

$$r_{G6PDH} = K^{\max}_{G6PDH} \frac{\left[G6P\right]}{\left[G6P\right]+k^m_{G6PFH}} \tag{50}$$

$$r_{UT} = K^{\max}_{UT} \frac{\left[G6P\right]}{\left[G6P\right]+k^m_{UT}} b_{NAD} \tag{51}$$

$$r_{cUGLC} = K^{\max}_{cUGLC} \frac{\left[UDPGlc\right]}{\left[UDPGlc\right]+k^m_{cUGLC}} \tag{52}$$

$$r_{PFK} = K_{PFK}^{\max} \frac{[F6P]}{k_{PFK}^{m} + [F6P]} \left(\frac{k_{PFK}^{a}}{k_{PFK}^{a} + [ATP]} \right)^{4} \tag{53}$$

$$r_{TATKF6P} = K_{TATKF6P}^{\max} \frac{[R5P] - \frac{[F6P]}{k_{TATKF6P}^{eq}}}{\frac{[F6P]}{k_{TATKF6P}^{eq}} + k_{TATKF6P}^{m} + [R5P]} \tag{54}$$

$$r_{TATK3PG} = K_{TATK3PG}^{\max} \frac{[R5P] - \frac{[3PG]}{k_{TATK3PG}^{eq}}}{\frac{[3PG]}{k_{TATK3PG}^{eq}} + k_{TATK3PG}^{m} + [R5P]} \tag{55}$$

$$r_{dR5P} = K_{dR5P}^{\max} \frac{[R5P]}{[R5P] + k_{dR5P}^{m}} \tag{56}$$

$$r_{GLYS} = K_{GLYS}^{\max} \frac{[UDPGlc]}{[UDPGlc] + k_{GLYS}^{m}} \tag{57}$$

$$r_{ENO} = K_{ENO}^{\max} \frac{\frac{[3PG]}{k_{3PG}} \left(1 - \frac{[PEP]}{[3PG] k_{ENO}^{eq}} \right)}{1 + \frac{[3PG]}{k_{3PG}} + \frac{[PEP]}{k_{PEP}}} \tag{58}$$

$$r_{ALD} = K_{ALD}^{\max} \frac{\frac{[F16P]}{k_{F16P}} \left(1 - \frac{[3PG]}{[F16P] k_{ALD}^{eq}} \right)}{1 + \frac{[F16P]}{k_{F16P}} + \frac{[3PG]}{k_{3PG_{ALD}}} + \frac{[3PG][ATP]}{k_{3PG_{ALD}} k_{ATP_{ALD}}} + \frac{[ATP]}{k_{ATP_{ALD}}} + k_{\mu}^{i} (1 - f)} \tag{59}$$

$$r_{PK} = K_{PK}^{\max} \frac{\frac{[PEP]}{k_{PEP_{PK}}} - \frac{[Pyr]}{k_{Pyr_{PK}}}}{1 + \frac{[PEP]}{k_{PEP_{PK}}} + \frac{[Pyr]}{k_{Pyr_{PK}}}} \tag{60}$$

$$r_{LDH} = K_{LDH}^{\max} \frac{\frac{[Pyr]}{k_{Pyr}}\left(1-\frac{\frac{[Lac]}{[Pyr]}}{k_{LDH}^{eq}}\right)\left(\frac{[Pyr]}{k_{Pyr_{LDH}}}+\frac{[Lac]}{k_{Lac_{LDH}}}\right)^{3}}{\left(\frac{[Pyr]}{k_{Pyr_{LDH}}}+\frac{[Lac]}{k_{Lac_{LDH}}}\right)^{4}+\left(\frac{1+k_{Pyr_{LDH}}^{a}\left(\frac{[Pyr]}{k_{cPyr}}\right)^{4}}{1+[Pyr]^{4}}\right)\left(\frac{1+k_{Glu_{LDH}}^{i}\left(\frac{[Glu]}{k_{Glu_{LDH}}}\right)^{4}}{1+[Glu]^{4}}\right)} \quad (61)$$

2.2.2 Rates from glycolysis, TCA and other pathways.

$$r_{PEPCK} = K_{PEPCK}^{\max} \frac{[OAA]}{[OAA]+k_{PEPCK}^{m}} b_{NAD} \quad (62)$$

$$r_{PDH} = K_{PDH}^{\max} \frac{[Pyr][ATP]}{[Pyr][ATP]+k_{Pyr_{PDH}}^{m}[Pyr]+\mathrm{k}_{ATP_{PDH}}^{m}[Pyr]+\mathrm{k}_{ATP_{PDH}}^{m}\,k_{Pyr_{PDH}}^{m}} b_{NAD} \quad (63)$$

$$r_{PC} = K_{PC}^{\max} \frac{[Pyr][ATP]}{[Pyr][ATP]+k_{Pyr_{PC}}^{m}[Pyr]+\mathrm{k}_{ATP_{PC}}^{m}[Pyr]+\mathrm{k}_{ATP_{PC}}^{m}\,k_{Pyr_{PC}}^{m}} b_{NAD} \quad (64)$$

$$r_{ME} = K_{ME}^{\max} \frac{[Mal]}{k_{ME}^{m}\left(1+\frac{[ATP]}{k_{ATP_{ME}}^{i}}\right)+[Mal]} \quad (65)$$

$$r_{AlaTA} = K_{AlaTA}^{\max} \frac{[Pyr]}{k_{AlaTA}^{m}+[Pyr]}\left(\frac{[Glu]}{k_{Glu_{AlaTA}}^{a}+[Glu]}\right)^{4} \quad (66)$$

$$r_{d\mathrm{NH4}} = K_{d\mathrm{NH4}}^{\max} \frac{[\mathrm{NH4}]}{k_{d\mathrm{NH4}}^{m}+[\mathrm{NH4}]}\left(\frac{[ATP]}{k_{ATP_{d\mathrm{NH4}}}^{a}+[ATP]}\right)^{4} \quad (67)$$

2.2.3 Rates from TCA

$$r_{CS} = K_{CS}^{\max} \frac{[OAA][AcCoA]}{k_{AcCoA}^{m}[OAA] + k_{OAA}^{m}[AcCoA] + [AcCoA][OAA]} \tag{68}$$

$$r_{ACO} = K_{ACO}^{\max} \left([Cit] - \frac{[cAc]}{k_{ACO}^{eq}} \right) \tag{69}$$

$$r_{ACO2} = K_{ACO}^{\max} \left([cAc] - \frac{[Isocit]}{k_{ACO2}^{eq}} \right) \tag{70}$$

$$r_{CL} = K_{CL}^{\max} \frac{[Cit][ATP]}{[Cit][ATP] + k_{Cit}^{m}[Pyr] + \mathrm{k}_{ATP_{CL}}^{m}[Pyr] + \mathrm{k}_{ATP_{CL}}^{m} k_{Cit}^{m}} (1 - b_{NAD}) \tag{71}$$

$$r_{ICDH} = K_{ICDH}^{\max} \frac{[Isocit] - \frac{[Keto]}{k_{ICDH}^{eq}}}{k_{ICDH}^{m} + [Isocit] + \frac{[Keto]}{k_{ICDH}^{eq}}} b_{NAD} \tag{72}$$

$$r_{GS} = K_{GS}^{\max} \frac{[Glu][ATP]}{[Glu][ATP] + k_{Glu_{GS}}^{m}[\mathrm{Glu}] + \mathrm{k}_{ATP_{GS}}^{m}[Glu] + \mathrm{k}_{ATP_{GS}}^{m} k_{Glu_{GS}}^{m}} \tag{73}$$

$$r_{Glnase} = K_{Glnase}^{\max} \frac{[Gln]}{[Gln] + k_{\mathrm{Glnase}}^{m} + \frac{[Glu]}{k_{ATP_{Glnase}}^{i}}} \tag{74}$$

$$r_{GLDH} = K_{GLDH}^{\max} \frac{[Glu] - \frac{[Keto]}{k_{GLDH}^{eq}}}{k_{GLDH}^{m} + [Glu] + \frac{[Keto]}{k_{GLDH}^{eq}} + \frac{[Glu]}{k_{Glu_{GLDH}}^{i}}} \tag{75}$$

$$r_{AspTA} = K_{AspTA}^{\max} \frac{\frac{[Keto]}{k_{keto}} - \frac{[OAA][Glu]}{K_{AspTA_{OAA}}^{eq} K_{AspTA_{Glu}}^{eq}}}{k_{AspTA}^{m} + \frac{[OAA][Glu]}{K_{AspTA_{OAA}}^{eq} K_{AspTA_{Glu}}^{eq}} + \frac{[Keto]}{k_{keto}}} b_{NAD} \tag{76}$$

$$r_{AAex} = K_{AAex}^{\max} \left(1 - \frac{[Keto]}{k_{AAex}^{eq}} \right) b_{NAD} \tag{77}$$

$$r_{KDH} = K_{KDH}^{\max} \frac{[Keto]}{[Keto] + k_{KDH}^{m}} \tag{78}$$

$$r_{SDH} = K_{KDH}^{\max} \frac{[Suc]}{[Suc] + k_{SDH}^{m} + \frac{[OAA]}{k_{OAA_{SDH}}^{i}}} \tag{79}$$

$$r_{FMA} = K_{FMA}^{\max} \frac{\left([Fum] - \frac{Mal}{k_{FMA}^{eq}}\right)}{k_{FMA}^{m} + [Fum] + \frac{[Mal]}{k_{FMA}^{eq}}} \tag{80}$$

$$r_{MDH} = K_{MDH}^{\max} \frac{[Mal]}{[Mal] + k_{MDH}^{m}} \tag{81}$$

2.2.3 Rates from energy production

$$r_{Glycolysis} = r_{PK} + 2r_{ALD} - r_{HK} - r_{PFK} \tag{82}$$

$$r_{TCA} = 2.5r_{NADH} + 1.5r_{FADH} \tag{83}$$

$$r_{CCM} = r_{Glycolysis} + r_{TCA} - r_{GS} - r_{CL} - r_{PC} \tag{84}$$

$$r_{xATP} = k_{xATP}\mu[ATP] \tag{85}$$

$$r_{mATP} = V_{cs} k_{mATP}[ATP] \tag{86}$$

$$r_{ATPase} = k_{ATPase} \frac{[ATP]}{[ATP] + k_{ATPase}^{m}} \tag{87}$$

$$r_{dATP} = r_{xATP} + r_{mATP} + r_{ATPase} \tag{88}$$

$$r_{NADH} = 2r_{ALD} + r_{MDH} + r_{ICDH} + r_{KDH} + 2r_{AAex} + r_{GLDH} + r_{PDH} - r_{LDH} \tag{89}$$

$$r_{FADH} = r_{SDH} + 2r_{AAex} \tag{90}$$

$$r_{O_2} = 60\frac{r_{NADH} + r_{FADH}}{2} V_s^c 10^{12} \tag{91}$$

3 Supplementary references

1. Rehberg, M.; Ritter, J.B.; Reichl, U. Glycolysis Is Governed by Growth Regime and Simple Enzyme Regulation in Adherent MDCK Cells. *PLoS Comput. Biol.* **2014**, *10*, e1003885, doi:10.1371/journal.pcbi.1003885.
2. Ramos, J.R.C.; Rath, A.G.; Genzel, Y.; Sandig, V.; Reichl, U. A dynamic model linking cell growth to intracellular metabolism and extracellular by-product accumulation. *Biotechnol. Bioeng.* **2020**, *117*, 1533–1553, doi:10.1002/bit.27288.
3. Rehberg, M.; Ritter, J.B.; Genzel, Y.; Flockerzi, D.; Reichl, U. The relation between growth phases, cell volume changes and metabolism of adherent cells during cultivation. *J. Biotechnol.* **2013**, *164*, 489–499, doi:10.1016/j.jbiotec.2013.01.018.
4. Rehberg, M.; Wetzel, M.; Ritter, J.B.; Reichl, U. The regulation of glutaminolysis and citric acid cycle activity during mammalian cell cultivation. *12th IFAC Symp. Comput. Appl. Biotechnol. CAB 2013* **2013**, *12*, 48–53, doi:10.3182/20131216-3-IN-2044.00011.
5. Uldry, M.; Ibberson, M.; Hosokawa, M.; Thorens, B. GLUT2 is a high affinity glucosamine transporter. *FEBS Lett.* **2002**, *524*, 199–203, doi:10.1016/S0014-5793(02)03058-2.
6. Marín-Hernández, A.; Gallardo-Pérez, J.C.; Rodríguez-Enríquez, S.; Encalada, R.; Moreno-Sánchez, R.; Saavedra, E. Modeling cancer glycolysis. *Biochim. Biophys. Acta - Bioenerg.* **2011**, *1807*, 755–767, doi:10.1016/j.bbabio.2010.11.006.
7. Luni, C.; Marth, J.D.; Doyle, F.J. Computational Modeling of Glucose Transport in Pancreatic ??-Cells Identifies Metabolic Thresholds and Therapeutic Targets in Diabetes. *PLoS One* **2012**, *7*, 1–8, doi:10.1371/journal.pone.0053130.
8. de Tremblay, M.; Perrier, M.; Chavarie, C.; Archambault, J. Optimization of fed-batch culture of hybridoma cells using dynamic programming: single and multi feed cases. *Bioprocess Eng.* **1992**, *7*, 229–234, doi:10.1007/BF00369551.
9. Barford, J.; Phillips, P.; Harbour, C. Simulation of animal cell metabolism. *Math. Comput. Simul.* **1992**, *33*, 397–402, doi:10.1016/0378-4754(92)90128-4.
10. Rath, A.G.; Rehberg, M.; Janke, R.; Genzel, Y.; Scholz, S.; Noll, T.; Rose, T.; Sandig, V.; Reichl, U. The influence of cell growth and enzyme activity changes on intracellular metabolite dynamics in AGE1.HN.AAT cells. *J. Biotechnol.* **2014**, *178*, 43–53, doi:10.1016/j.jbiotec.2014.03.012.
11. Ozturk, S.S.; Palsson, B.O. Chemical decomposition of glutamine in cell culture media: effect of media type, pH, and serum concentration. *Biotechnol. Prog.* **1990**, *6*, 121–128, doi:10.1021/bp00002a005.
12. Sauro, H.M. *Enzyme Kinetics for Systems Biology*; Future Skill Software (Ambrosius Publishing), 2011; ISBN 9780982477311.
13. J.G.Salway Summary for Policymakers. *Clim. Chang. 2013 - Phys. Sci. Basis* **2004**, *16*, 1–30, doi:10.1017/CBO9781107415324.004.
14. Xie, L.; Wang, D.I.C. Energy metabolism and ATP balance in animal cell cultivation

using a stoichiometrically based reaction network. *Biotechnol. Bioeng.* **1996**, *52*, 591–601, doi:10.1002/(SICI)1097-0290(19961205)52:5<591::AID-BIT6>3.0.CO;2-E.
