# Supplementary material for: Impact of Influenza A Virus Infection on Growth and Metabolism of Suspension MDCK Cells Using a Dynamic Model
Source: Metabolites. 2022 Mar 12;12(3):239. doi: 10.3390/metabo12030239 (PMC8950586; doi:10.3390/metabo12030239)
Supplement: Supplementary file 1 [file metabolites-12-00239-s001.zip › Supplementary File S2.pdf]

João R. C. Ramos, Thomas Bissinger, Yvonne Genzel, Udo Reichl, Impact of influenza A virus infection on growth and metabolism of suspension MDCK cells using a dynamic model

List of symbols.

Symbol	Unit	Description
Θ	-	unit step function
ε	-	unit step function
$\Phi_{1,..,3}$	-	exponential step function
$\rho_{1,...,3}$	-	constant to adjust the exponential step function
ω	-	constant to adjust ammonium release from amino acid degradation
μ_{max}	1/min	maximum cell-specific growth rate
μ	1/min	cell-specific growth rate
α	-	constant adjusting inhibition factor
β	1/min	switch constant for cell death rate
$\overline{d}$	µm	mean cell diameter
b_{NAD}	-	adjustable parameter for influence of NAD/NADH
d_c	µm	critical cell diameter
d_m	µm	minimum cell diameter
f	-	growth inhibition factor
N^c	-	number of cell classes
v_e^{max}	L/cell/min	cell number-specific activity of enzyme e

List of symbols (continued).

Symbol	Unit	Description
$v_{Glu^x_{trans}}$	mmol/L/µL/min	cell volume-specific transport rate of Glu
v^{max}_{HK}	mmol/cell/min	cell number-specific maximum activity of HK
v^{max}_{GPI}	mmol/cell/min	cell number-specific maximum activity of GPI
v^{max}_{G6PDH}	mmol/cell/min	cell number-specific maximum activity of G6PDH
$v_{Lac^x_{trans}}$	mmol/L/µL/min	cell volume-specific transport rate of Lac
$v_{Gln^x_{trans}}$	mmol/L/µL/min	cell volume-specific transport rate of Gln
$v_{NH4^x_{trans}}$	mmol/L/µL/min	cell volume-specific transport rate of NH4
v^{max}_{dR5P}	mmol/cell/min	cell number-specific maximum activity of R5P
v^{max}_{UT}	mmol/cell/min	cell number-specific maximum activity of UT
v^{max}_{GLYS}	mmol/cell/min	cell number-specific maximum activity of GLYS
v^{max}_{PFK}	mmol/cell/min	cell number-specific maximum activity of PFK
$v^{max}_{TATKF6P}$	mmol/cell/min	cell number-specific maximum activity of TATKF6P
$v^{max}_{TATK3PG}$	mmol/cell/min	cell number-specific maximum activity of TATK3GP
v^{max}_{ALD}	mmol/cell/min	cell number-specific maximum activity of ALD
$v_{Pyr^x_{trans}}$	mmol/L/µL/min	cell volume-specific transport rate of Pyr

List of symbols (continued).

Symbol	Unit	Description
v_{ENO}^{max}	mmol/cell/min	cell number-specific maximum activity of ENO
v_{PK}^{max}	mmol/cell/min	cell number-specific maximum activity of PK
v_{LDH}^{max}	mmol/cell/min	cell number-specific maximum activity of LDH
v_{ACO}^{max}	mmol/cell/min	cell number-specific maximum activity of PDH
v_{CL}^{max}	mmol/cell/min	cell number-specific maximum activity of ACO
v_{ICDH}^{max}	mmol/cell/min	cell number-specific maximum activity of CL
v_{GS}^{max}	mmol/cell/min	cell number-specific maximum activity of ICDH
v_{KDH}^{max}	mmol/cell/min	cell number-specific maximum activity of GS
v_{SDH}^{max}	mmol/cell/min	cell number-specific maximum activity of SDH
v_{FMA}^{max}	mmol/cell/min	cell number-specific maximum activity of FMA
v_{MDH}^{max}	mmol/cell/min	cell number-specific maximum activity of MDH
v_{ATPase}^{max}	mmol/cell/min	cell number-specific maximum activity of ATPase
v_{AAex}^{max}	mmol/cell/min	cell number-specific maximum activity of AAex
v_{cUGLC}^{max}	mmol/cell/min	cell number-specific maximum activity of UGLC
v_{CS}^{max}	mmol/cell/min	cell number-specific maximum activity of CS
v_{ME}^{max}	mmol/cell/min	cell number-specific maximum activity of ME

List of symbols (continued).

Symbol	Unit	Description
v_{PEPCK}^{max}	mmol/cell/min	cell number-specific maximum activity of PPECK
v_{PC}^{max}	mmol/cell/min	cell number-specific maximum activity of PC
v_{AlaTA}^{max}	mmol/cell/min	cell number-specific maximum activity of AlaTA
v_{AspTA}^{max}	mmol/cell/min	cell number-specific maximum activity of AspTA
v_{GLDH}^{max}	mmol/cell/min	cell number-specific maximum activity of GLDH
v_{GLNase}^{max}	mmol/cell/min	cell number-specific maximum activity of GLNase
v_{dNH4}	mmol/cell/min	cell number-specific maximum activity of NH4 degradation
k_{HK}^{m}	mmol/L	affinity constant of HK
k_{GPI}^{m}	mmol/L	affinity constant of GPI
k_{GPI}^{eq}	-	equilibrium constant of GPI
k_{G6PDH}^{m}	mmol/L	affinity constant of G6PDH
k_{UT}^{m}	mmol/L	affinity constant of UT
$k_{TATKF6P}^{eq}$	-	equilibrium constant of TATKF6P
$k_{TATK3PG}^{eq}$	-	equilibrium constant of TATK3PG

List of symbols (continued).

Symbol	Unit	Description
k^{m}_{PFK}	mmol/L	affinity constant of PFK
k_{F16P}	mmol/L	affinity constant of ALD for F16P
k^{eq}_{ENO}	-	equilibrium constant of ENO
$k_{PEP_{PK}}$	mmol/L	affinity constant of PK for PEP
$k_{Pyr_{PK}}$	mmol/L	affinity constant of PK for Pyr
$k_{Pyr_{PDH}}$	mmol/L	affinity constant of PDH for Pyr
k^{eq}_{ACO}	-	equilibrium constant of ACO
k^{eq}_{ACO2}	-	equilibrium constant of ACO
k^{m}_{Cit}	mmol/L	affinity constant of CL for Cit
k^{eq}_{ICDH}	-	equilibrium constant of ICDH
k^{eq}_{AAex}	-	equilibrium constant of AAex
k^{eq}_{FMA}	-	equilibrium constant of FMA
k^{m}_{FMA}	mmol/L	affinity constant of FMA
k^{m}_{MDH}	mmol/L	affinity constant of MDH
k_{xATP}	cell/L/min	specific ATP consumption related to growth

List of symbols (continued).

Symbol	Unit	Description
k_{mATP}	cell/L/min	specific ATP consumption related to maintenance
NAD_{basal}	mmol/L	adjustable parameter for influence of NAD/NADH
k^{m}_{cUGLC}	mmol/L	affinity constant of a general enzyme to UDPGlc
k^{m}_{ME}	mmol/L	affinity constant of ME
$k^{i}_{ATP_{ME}}$	$mmol^2/L^2$	inhibition constant of ME from ATP
k^{i}_{μ}	mmol/L	growth related inhibition constant of ALD
$k^{m}_{Glu_{GS}}$	mmol/L	affinity constant of GS for Glu
k^{m}_{ICDH}	mmol/L	affinity constant of ICDH
k^{m}_{OAA}	mmol/L	affinity constant of CS for OAA
k^{m}_{AcCoA}	mmol/L	affinity constant of CS for AcCoA
k^{m}_{SDH}	mmol/L	affinity constant of SDH
k^{m}_{dR5P}	mmol/L	affinity constant of a general enzyme to R5P
k^{m}_{PEPCK}	mmol/L	affinity constant of PEPCK
$k^{m}_{Pyr_{PC}}$	mmol/L	affinity constant of PC for Pyr

List of symbols (continued).

Symbol	Unit	Description
k_{ME}^{eq}	-	equilibrium constant of ME
k_{KDH}^{m}	mmol/L	affinity constant of KDH
$k_{OAA_{AspTA}}$	mmol/L	affinity constant of AspTA for OAA
$k_{Glu_{AspTA}}$	mmol/L	affinity constant of AspTA for Glu
$k_{Keto_{AspTA}}$	mmol/L	affinity constant of AspTA for Keto
$k_{Glu_{GLDH}}$	mmol/L	affinity constant of LDH for Glu
k_{Glnase}^{m}	mmol/L	affinity constant of GLNase
k_{GLDH}^{eq}	-	equilibrium constant of GLDH
k_{ALD}^{eq}	-	equilibrium constant of ALD
k_{PFK}^{a}	mmol/L	activation constant of PFK
k_{ATPase}^{m}	mmol/L	affinity constant of ATPase
k_{3PG}	mmol/L	affinity constant of ENO for 3PG
$k_{PEP_{ENO}}$	mmol/L	affinity constant of ENO for PEP
$k_{TATK3PG}^{m}$	mmol/L	affinity constant of TATK3PG
$k_{TATKF6P}^{m}$	mmol/L	affinity constant of TATKF6P

List of symbols (continued).

Symbol	Unit	Description
k^{m}_{GLDH}	mmol/L	affinity constant of GLDH
$k^{m}_{ATP_{HK}}$	mmol/L	affinity constant of HK for ATP
$k^{m}_{ATP_{ALD}}$	mmol/L	affinity constant of ALD for ATP
$k^{m}_{3PG_{ALD}}$	mmol/L	affinity constant of ALD for 3PG
$k^{m}_{ATP_{PC}}$	mmol/L	affinity constant of PC for ATP
$k^{m}_{ATP_{PDH}}$	mmol/L	affinity constant of PDH for ATP
$k_{Glu^{x}}$	mmol/L	direct binding affinity constant for extracellular Glu
k_{Glu}	mmol/L	direct binding affinity constant for Glu transporter
$k^{eq}_{Glu^{x}_{trans}}$	-	direct biding equilibrium constant for Glu transporter
$k_{Gln^{x}}$	mmol/L	Direct binding (simplified) affinity constant for extracellular Gln
$k^{m}_{Pyr^{x}_{trans}}$	mmol/L	affinity constant of extracellular Pyr transporter
k^{m}_{AspTA}	mmol/L	affinity constant of AspTA
$k^{m}_{ATP_{GS}}$	mmol/L	affinity constant of GS for ATP
$k^{i}_{ATP_{Glnase}}$	$mmol^{2}/L^{2}$	inhibition constant of GLNase from ATP

List of symbols (continued).

Symbol	Unit	Description
$k^{i}_{OAA_{SDH}}$	$mmol^2/L^2$	inhibition constant of SDH from ATP
k^{m}_{GLYS}	mmol/L	affinity constant of GLYS
$k^{m}_{ATP_{CL}}$	mmol/L	affinity constant of CL for ATP
$k^{eq}_{Lac^{x}_{trans}}$	-	equilibrium constant of transporter for Lac
$k_{Lac_{trans}}$	mmol/L	affinity constant of transporter for Lac
$k_{Lac^{x}_{trans}}$	mmol/L	affinity constant of transporter for extracellular Lac
$k_{Pyr_{LDH}}$	mmol/L	affinity constant of LDH for Pyr
$k_{Lac_{LDH}}$	mmol/L	affinity constant of LDH for Lac
k_{cPyr}	$mmol^2/L^2$	activation constant of LDH from Pyr
$k^{a}_{Pyr_{LDH}}$	-	activation constant of LDH from Pyr
$k_{Glu_{LDH}}$	$mmol^2/L^2$	inhibition constant of LDH from Glu
$k^{i}_{Glu_{LDH}}$	-	inhibition constant of LDH from Glu
k^{eq}_{LDH}	-	equilibrium constant of LDH
$k^{m}_{d\mathrm{NH4}}$	mmol/L	affinity constant of general enzyme for NH4
$k^{a}_{ATP_{d\mathrm{NH4}}}$	mmol/L	activation constant of NH4 degradation from ATP

List of symbols (continued).

Symbol	Unit	Description
k_{NH4^x}	mmol/L	direct binding affinity constant for NH_4 transporter
$k^{eq}_{NH4^x_{trans}}$	-	direct biding equilibrium constant of NH_4 transporter
k_{NH4}	mmol/L	direct binding affinity constant for NH_4 transporter
k^m_{AlaTA}	mmol/L	affinity constant of AlaTA
$k^i_{Glu_{AlaTA}}$	$mmol^2/L^2$	inhibition constant of AlaTA from Glu
$k^m_{Glc^x}$	mmol/L	Monod constant for extracellular Glc
$k_d^{\min}$	1/min	basal cell death rate
$k_d^{\max}$	1/min	maximum cell death rate
$k_d^{\min\inf}$	1/min	basal cell death rate of infected cells
$k_d^{\max\inf}$	1/min	maximum cell death rate of infected cells
$k_{dG\ln^x}$	1/min	spontaneous glutamine degradation rate
m_{Glc^x}	mmol/L/µL/min	maintenance related glucose consumption rate
$K_e^{\max}$	mmol/L/min	cell volume-specific reaction rate of enzyme e
$K_{HK}^{\max}$	mmol/L/min	cell volume-specific reaction rate of enzyme HK
$K_{GPI}^{\max}$	mmol/L/min	cell volume-specific reaction rate of enzyme GPI

List of symbols (continued).

Symbol	Unit	Description
K_{G6PDH}^{max}	mmol/L/min	cell volume-specific reaction rate of enzyme G6PDH
K_{dR5P}^{max}	mmol/L/min	cell volume-specific reaction rate of general enzyme of R5P degradation
K_{UT}^{max}	mmol/L/min	cell volume-specific reaction rate of enzyme UT
K_{GLYS}^{max}	mmol/L/min	cell volume-specific reaction rate of enzyme GLYS
K_{PFK}^{max}	mmol/L/min	cell volume-specific reaction rate of enzyme PFK
$K_{TATKF6P}^{max}$	mmol/L/min	cell volume-specific reaction rate of transaldolase and transketolase for F6P
$K_{TATK3PG}^{max}$	mmol/L/min	cell volume-specific reaction rate of transaldolase and transketolase for 3PG
K_{ALD}^{max}	mmol/L/min	cell volume-specific reaction rate of enzyme ALD
K_{ENO}^{max}	mmol/L/min	cell volume-specific reaction rate of enzyme ENO
K_{PK}^{max}	mmol/L/min	cell volume-specific reaction rate of enzyme PK
K_{LDH}^{max}	mmol/L/min	cell volume-specific reaction rate of enzyme LDH
K_{PDH}^{max}	mmol/L/min	cell volume-specific reaction rate of enzyme PDH
K_{dNH4}^{max}	mmol/L/min	cell volume-specific reaction rate of general enzyme for NH4 degradation
K_{ACO}^{max}	mmol/L/min	cell volume-specific reaction rate of enzyme ACO
K_{CL}^{max}	mmol/L/min	cell volume-specific reaction rate of enzyme CL

List of symbols (continued).

Symbol	Unit	Description
$K_{ICDH}^{\max}$	mmol/L/min	cell volume-specific reaction rate of enzyme ICDH
$K_{GS}^{\max}$	mmol/L/min	cell volume-specific reaction rate of enzyme GS
$K_{KDH}^{\max}$	mmol/L/min	cell volume-specific reaction rate of enzyme KDH
$K_{SDH}^{\max}$	mmol/L/min	cell volume-specific reaction rate of enzyme SDH
$K_{FMA}^{\max}$	mmol/L/min	cell volume-specific reaction rate of enzyme FMA
$K_{MDH}^{\max}$	mmol/L/min	cell volume-specific reaction rate of enzyme MDH
$K_{ATPase}^{\max}$	mmol/L/min	cell volume-specific reaction rate of enzyme ATPase
$K_{AAex}^{\max}$	mmol/L/min	cell volume-specific reaction rate of enzyme AAex
$K_{cUGLC}^{\max}$	mmol/L/min	cell volume-specific reaction rate of enzyme UGLC
$K_{CS}^{\max}$	mmol/L/min	cell volume-specific reaction rate of enzyme CS
$K_{ME}^{\max}$	mmol/L/min	cell volume-specific reaction rate of enzyme ME
$K_{PEPCK}^{\max}$	mmol/L/min	cell volume-specific reaction rate of enzyme PEPCK
$K_{PC}^{\max}$	mmol/L/min	cell volume-specific reaction rate of enzyme PC
$K_{AlaTA}^{\max}$	mmol/L/min	cell volume-specific reaction rate of enzyme AlaTA
$K_{AspTA}^{\max}$	mmol/L/min	cell volume-specific reaction rate of enzyme AspTA

List of symbols (continued).

Symbol	Unit	Description
$K_{GLDH}^{\max}$	mmol/L/min	cell volume-specific reaction rate of enzyme GLDH
$K_{GLNase}^{\max}$	mmol/L/min	cell volume-specific reaction rate of enzyme GLNase
NAD_{basal}	-	adjustable parameter for influence of NAD/NADH
r_i	mmol/L/min	cell-number-specific activity of enzyme i
r_{macro}	-	formula for conversion of macroscopic scale rates to microscopic scale
r_{HK}	mmol/L/min	cell volume-specific reaction rate of HK
r_{GPI}	mmol/L/min	cell volume-specific reaction rate of GPI
r_{G6PDH}	mmol/L/min	cell volume-specific reaction rate of G6PDH
r_{dR5P}	mmol/L/min	cell volume-specific reaction rate of R5P degradation
r_{dNH4}	mmol/L/min	cell volume-specific reaction rate of NH4 degradation
r_{UT}	mmol/L/min	cell volume-specific reaction rate of UT
r_{GLYS}	mmol/L/min	cell volume-specific reaction rate of GLYS
r_{PFK}	mmol/L/min	cell volume-specific reaction rate of PFK
$r_{TATKF6P}$	mmol/L/min	cell volume-specific reaction rate of TATKF6P
$r_{TATK3PG}$	mmol/L/min	cell volume-specific reaction rate of TATK3PG

List of symbols (continued).

Symbol	Unit	Description
r_{ALD}	mmol/L/min	cell volume-specific reaction rate of ALD
r_{ENO}	mmol/L/min	cell volume-specific reaction rate of ENO
r_{PK}	mmol/L/min	cell volume-specific reaction rate of PK
r_{LDH}	mmol/L/min	cell volume-specific reaction rate of LDH
r_{PDH}	mmol/L/min	cell volume-specific reaction rate of PDH
r_{ACO}	mmol/L/min	cell volume-specific reaction rate of ACO
r_{CL}	mmol/L/min	cell volume-specific reaction rate of CL
r_{ICDH}	mmol/L/min	cell volume-specific reaction rate of ICDH
r_{GS}	mmol/L/min	cell volume-specific reaction rate of GS
r_{KDH}	mmol/L/min	cell volume-specific reaction rate of KDH
r_{SDH}	mmol/L/min	cell volume-specific reaction rate of SDH
r_{FMA}	mmol/L/min	cell volume-specific reaction rate of FMA
r_{MDH}	mmol/L/min	cell volume-specific reaction rate of MDH
r_{ATPase}	mmol/L/min	cell volume-specific reaction rate of ATPase
r_{AAex}	mmol/L/min	cell volume-specific reaction rate of AAex

List of symbols (continued).

Symbol	Unit	Description
r_{cUGLC}	mmol/L/min	cell volume-specific reaction rate of UGLC
r_{CS}	mmol/L/min	cell volume-specific reaction rate of CS
r_{ME}	mmol/L/min	cell volume-specific reaction rate of ME
r_{PEPCK}	mmol/L/min	cell volume-specific reaction rate of PEPCK
r_{PC}	mmol/L/min	cell volume-specific reaction rate of PC
r_{AlaTA}	mmol/L/min	cell volume-specific reaction rate of AlaTA
r_{AspTA}	mmol/L/min	cell volume-specific reaction rate of AspTA
r_{GLDH}	mmol/L/min	cell volume-specific reaction rate of GLDH
r_{GLNase}	mmol/L/min	cell volume-specific reaction rate of GLNase
$r_{Lac^x_{trans}}$	mmol/L/min	medium volume-specific transport rate of Lac
$r_{NH4^x_{trans}}$	mmol/L/min	medium volume-specific transport rate of NH4
$r_{Gln^x_{trans}}$	mmol/L/min	medium volume-specific transport rate of Gln
$r_{Glu^x_{trans}}$	mmol/L/min	medium volume-specific transport rate of Glu
$r_{Pyr^x_{trans}}$	mmol/L/min	medium volume-specific transport rate of Pyr
r_{trans}	1/min	specific transition rate

List of symbols (continued).

Symbol	Unit	Description
r_{m/Glc^x}	mmol/L/min	medium volume-specific uptake rate of Glc for maintenance
r_{x/Glc^x}	mmol/L/min	medium volume-specific uptake rate of Glc for growth
r_{GLUT}	mmol/L/min	cell volume-specific transport rate of Glc
r_{xATP}	mmol/L/min	cell volume-specific ATP consumption rate of cell growth
r_{mATP}	mmol/L/min	cell volume-specific ATP consumption rate of cell maintenance
r_{ATPase}	mmol/L/min	cell volume-specific rate of ATPase
r_{dATP}	mmol/L/min	net ATP consumption rate
r_{CCM}	mmol/L/min	net ATP production rate
r_{NADH}	mmol/L/min	net ATP production from NADH
r_{FADH}	mmol/L/min	net ATP production from FADH
r_{TCA}	mmol/L/min	net ATP production from TCA
$r_{glycolysis}$	mmol/L/min	net ATP production from glycolysis
r_{O2}	fmol/cell/min	theoretical O2 consumption rate
V^c	µL/mL	viable cell volume
V_s^c	L/cell	cell-specific volume

List of symbols (continued).

Symbol	Unit	Description
V_w	-	working volume
X_i	cells/mL	number of viable cells of class i
X_v	cells/mL	viable cell concentration
Y_{x/Glc^x}	mmol/L/cell	cell growth-specific yield coefficient of Glc
E_{level}	-	relative enzyme level
IAV	-	influenza A virus
