# Supplementary material for: Impact of Influenza A Virus Infection on Growth and Metabolism of Suspension MDCK Cells Using a Dynamic Model
Source: Metabolites. 2022 Mar 12;12(3):239. doi: 10.3390/metabo12030239 (PMC8950586; doi:10.3390/metabo12030239)
Supplement: Supplementary file 1 [file metabolites-12-00239-s001.zip › Supplementary File S6.pdf]

João R. C. Ramos, Thomas Bissinger, Yvonne Genzel, Udo Reichl, Impact of influenza A virus infection on growth and metabolism of suspension MDCK cells using a dynamic model

Model *in-silico* studies - supporting information

Contents

1 Impact of different cell classes 2

2 Impact of growth-related time step function after virus infection 3

2 Impact of cell lysis on model prediction 5

2.1 Impact of intracellular metabolites leaking into the supernatant 5

2.2 Impact of intracellular enzymes leaking into the supernatant 9

3 Supplementary references 12

1 Impact of different cell classes

Five cell classes were used in this study, analogous to the models used to describe cell growth of suspension AGE1.HN and adherent MDCK cells [1,2]. In the following, *in-silico* studies were performed to compare the effects varying the number of cell classes on the model fitness (Figure 1, Table 1).

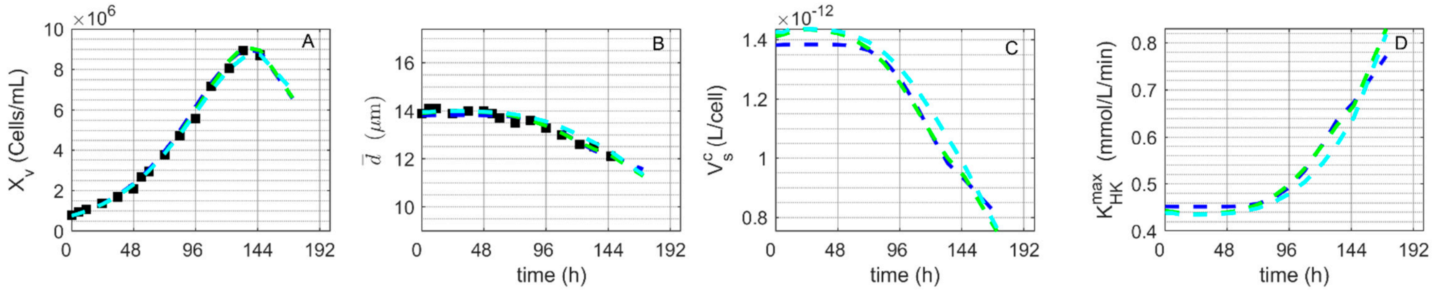

Figure 1. Model simulations considering a different number of cell classes. (A) Viable cell concentration, (B) mean cell diameter, (C) cell-specific volume and (D) cell-specific hexokinase activity. N^c: number of cell classes (Blue: N^C=2, Green: N^C=5 and Cyan: N^C= 7). Black squares: experimental data of Cultivation 1.

Table 1. Model fitness for the viable cell concentration and the mean cell diameter for different cell classes.

	**Model Fitness***		
**Number of cell Classes**	Viable cell concentration (X_v)	Mean cell diameter ($\bar{d}$)	Overall
N^c=2	0.0102	0.0026	0.0128
N^c=5	0.0067	0.0011	0.0078
N^c=7	0.0043	0.0034	0.0077

*Model fitness was calculated using Equation (8) (see main manuscript which this supplement belongs to).

These findings indicate that reducing the number of cell classes results in a worse fit of the viable cell concentration (X_v, Table 1) and the mean cell diameter ($\bar{d}$, Table 1). For example,

when two cell classes were used, the cell-specific volume ranges between 0.71–1.38e-12 L/cell, whereas when five classes were used, the range was 0.63–1.44e-12 L/cell (V_s^c, Figure 1C). Consequently, these differences in the cell-specific volume also had noticeable impact on cell-specific enzyme activities (e.g., for hexokinase, Figure 1D), which had significant impact on metabolism prediction over the course of 200h. The relationship between cell-specific volume and maximum volumetric enzyme activities was introduced in Equation (2) (section 1 in Supplementary File S1). On the other hand, increasing the number of cell classes, specifically to seven classes in this case, did not improve model fitness significantly. Given these results, the number of cell class of 5 was chosen, which is also similar to previous studies [1–4]. The models for simulation considering different cell classes are included in the Supplementary File S3.

2 Impact of growth-related time step function after virus infection

Cell growth is halted within a few hours of viral infection, virions are released and the cells die. Cell staining can be used to monitor the transition of a cell between its growth and apoptotic states, which is also indicated by a decline in viable cell concentration and mean cell diameter. In theory, after complete cell growth arrest, the majority of cells will remain in the first cell class (X_1) containing the smallest cells rather than transitioning to the last cell (X_5) that contains the largest cell cells. This implies that cells would only grow in size during cell growth phase to produce two daughter cells. In theory, this also implies that the mean cell diameter will tend to decrease to the smallest possible value as cell growth decreases over time. For this reason, a step function that accurately describes the transition from cell growth to cell death following viral infection is required in order to accurately describe viable cell, mean cell diameter and consequently the viable cell volume. In the following, an *in-silico* study compares the use of a smooth step-function and the absence of a step function (cell growth assumed to be zero immediately) after infection (results shown in Figure 2 and Table 2).

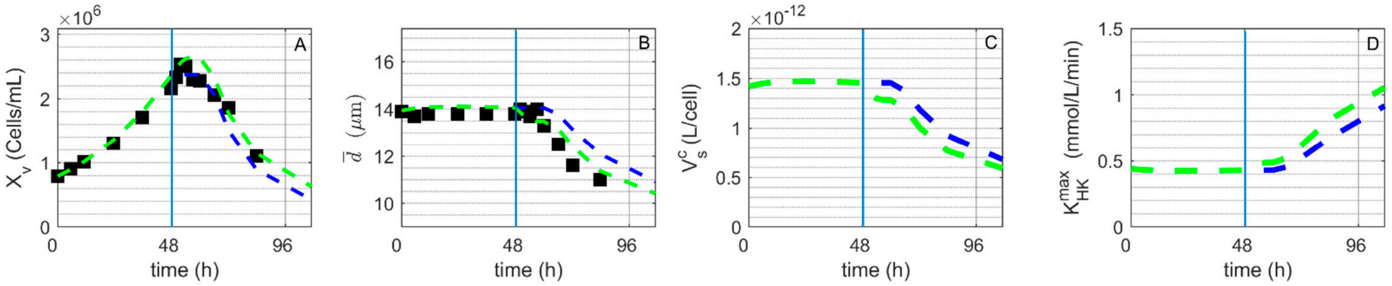

Figure 2. Model simulations considering a step function for the decrease in the cell transition rate after virus infection. (A) Viable cell concentration, (B) mean cell diameter, (C) cell-specific volume and (D) cell-specific hexokinase activity. Blue line: cell growth without a step function. Green: cell growth with a step function. Black squares: experimental data of Cultivation 2. Vertical blue line: time of infection (48 h).

Table 2. **Model fitness for viable cell concentration and mean cell diameter with and without step function for the cell growth rate.**

	Model Fitness*		
**Step function**	Viable cell concentration (X_v)	Mean cell diameter ($\bar{d}$)	Overall
without	0.0621	0.0333	0.0954
with	0.0670	0.0107	0.0776

*Model fitness was calculated using Equation (8) (see main manuscript which this supplement belongs to).

Overall better fit of the viable cell concentration (X_v , Figure 2A and Table 2) and the mean cell diameter ($\bar{d}$, Figure 2B and Table 2) is obtained using the smooth step function (Φ_1, introduced in Equation (5) on the manuscript which this supplement belongs to). This step function allows a better description of the decrease in the cell growth rate observed after virus infection. The decision to use a step function was also influenced by the accuracy of metabolic model predictions. For example, mean cell diameter affects the cell-specific volume (V_s^c , Figure 2C), which in turn has an impact on cell-specific enzyme activities (e.g., the cell-specific hexokinase

activity, Figure 2D). The correlation between the cell-specific volume and the maximum volumetric enzyme activities is described by Equation (2) (section 1 in Supplementary File S1). In this instance, without a step function, the cell-specific hexokinase activity ranges between 0.43–94 mmol/L/min, while with a step function, it ranges between 0.43–1.07 mmol/L/min. These small differences had significant effect on the model's prediction after virus infection, demonstrating the importance of using a step function. Models for simulations with and without the step function are included in the Supplementary File S3.

2 Impact of cell lysis on model prediction

2.1 Impact of intracellular metabolites leaking into the supernatant

The following section describes an *in-silico* study of the impact of intracellular metabolite release on the extracellular metabolite concentration following cell lysis. To investigate the impact of the intracellular metabolites (glucose, lactate, pyruvate, glutamate, glutamine and ammonia) release on their concentration in the supernatant after cell lysis, it was assumed that cell death occurs concomitantly with cell lysis. To begin, new model variables were introduced for each cell class's dead cells (X_{di} ,cells/ L, Equation (1)). The total number of dead cells per class is derived from multiplication of the cell death rate with the cell concentration of each cell class. The total number of dead cell (X_d , dead cells/ L, Equation (2)) is the sum of dead cells from all cell classes.

$$X_{di} = k_d X_i \quad i = 1, \ldots, N^c \tag{1}$$

$$X_d = \sum_{i=1}^{N^c} X_{di} \tag{2}$$

Similar to the average cell diameter of a viable cell, the average cell diameter of a dead cell ($\overline{d_d}$) was calculated using Equation (3).

$$\overline{d_d} = \sum_{i=1}^{N^c} \left(d_m + \frac{d_c - d_m}{N^c - 1}(i-1) \right) \frac{X_{id}}{X_d} \tag{3}$$

Like the viable cell volume, the dead cell volume (V^{dc}, Equation (4)) was calculated using the average diameter of dead cells and the total number of dead cells.

$$V^{dc} = \pi \frac{\overline{d_d}^3}{6} X_d 10^{-9} \tag{4}$$

Finally, the dead cell-specific volume (V_s^{dc}) was estimated using Equation (5).

$$V_s^{dc} = \frac{V_d^c}{X_d} 10^{-6} \tag{5}$$

Taking into account the conversion required to link the microscopic scale (cell volume) to the macroscopic scale (working volume) (introduced in Equation (1) in Supplementary File S1), the total number of moles of any intracellular metabolite (C) that can potentially be release into to the supernatant can be calculated by the multiplication of its intracellular concentration by the total volume of lysed cells ($V_s^{dc} X_d$). The corresponding increase of this metabolite on the supernatant or the macroscopic scale (C^x) is estimated taking into account conversion of the total number C into bioreactor level using the working volume (V_w), as shown in Equation (6). In Equation (6) the term φ refers to the other variables previously used to describe extracellular metabolites consumption/ secretion, as shown in Equations (10)-(15) in Supplementary File S1. Note certain substrates and metabolic by-products are present both in the supernatant and intracellularly, thus making it possible to estimate the impact of their release from intracellular to the bioreactor level. For example, intracellular glucose was designated Glc and the extracellular glucose as Glc^x.

$$\frac{d\left[C^x\right]}{dt} = \varphi + \frac{[C]V_s^{dc}X_d}{V_w} \tag{6}$$

The results of intracellular metabolites leaked into the bioreactor based on dead cells volume is presented in Table 3A and 3B.

Table 3A. Concentration of extracellular metabolites after virus infection with (+) and without (-) leakage of intracellular metabolites due to cell lysis. (Glc^x) extracellular glucose, (Lac^x) extracellular lactate and (Glu^x) extracellular glutamate; diff (%): percentage difference between both scenarios.

	**Glc^x**			**Lac^x**			**Glu^x**		
**Time (h)**	(-)	(+)	diff (%)	(-)	(+)	diff (%)	(-)	(+)	diff (%)
49.90	16.72	16.72	2.67E-06	6.36	6.36	4.46E-05	1.928	1.928	1.43E-05
51.90	16.54	16.54	4.27E-06	6.769	6.769	4.25E-05	1.956	1.956	4.14E-05
54.10	16.34	16.34	4.28E-06	7.195	7.195	3.85E-05	1.985	1.985	7.17E-05
57.00	16.09	16.09	4.39E-06	7.739	7.739	3.41E-05	2.021	2.021	0.00017
59.90	15.84	15.84	5.58E-06	8.271	8.271	9.33E-06	2.055	2.055	0.00133
66.10	15.33	15.33	1.77E-05	9.306	9.306	0.001687	2.117	2.119	0.1108
72.10	14.98	14.98	6.47E-06	9.939	9.94	0.004518	2.143	2.148	0.2672
83.80	14.6	14.6	3.42E-05	10.5	10.5	0.007366	2.155	2.163	0.37
107.00	14.26	14.26	**5.41E-05**	10.78	10.79	**0.009126**	2.158	2.167	**0.4062**

Table 3B. Concentration of extracellular metabolites after virus infection with (+) and without (-) leakage of intracellular metabolites due to cell lysis. (Pyr^x) extracellular pyruvate, (Gln^x) extracellular glutamine and ($NH4^x$) extracellular ammonium; diff (%): percentage difference between both scenarios.

	Pyr^x			Gln^x			$NH4^x$		
**Time (h)**	(-)	(+)	diff (%)	(-)	(+)	diff (%)	(-)	(+)	diff (%)
49.90	5.447	5.447	2.42E-06	2.611	2.611	2.55E-05	1.137	1.137	8.12E-05
51.90	5.297	5.297	2.30E-06	2.518	2.518	4.49E-05	1.163	1.163	1.68E-04
54.10	5.134	5.134	2.25E-06	2.417	2.417	6.72E-05	1.19	1.19	2.68E-04
57.00	4.922	4.922	2.63E-06	2.288	2.288	1.43E-04	1.224	1.224	0.0006158
59.90	4.711	4.711	8.89E-06	2.161	2.161	1.08E-03	1.256	1.256	0.004894
66.10	4.291	4.291	4.22E-04	1.913	1.915	0.0978	1.319	1.325	0.4282
72.10	4.011	4.011	1.13E-03	1.738	1.742	0.2403	1.373	1.388	1.049
83.80	3.708	3.708	1.80E-03	1.517	1.523	0.3384	1.466	1.487	1.47
107.00	3.441	3.441	**2.30E-03**	1.254	1.259	**0.3824**	1.621	1.646	**1.561**

Table 3A and B clearly show that the leakage of intracellular metabolites into the supernatant after cell lysis has only a minor impact on their corresponding extracellular concentrations. The smallest difference was found for glucose and pyruvate, followed by lactate (below 0.1 %, at 107 h). The highest difference between these two scenarios was found for glutamine, glutamate and ammonium (0.38–1.56 %, at 107 h). Overall, these results suggest that a leakage of intracellular metabolites into the extracellular environment can be neglected, especially as in this case only relatively low cell concentrations (2.1×10^6 cells/mL) was infected. Nevertheless, a high cell density cultivations [5–8] like 20×10^6 cells/mL could result in more than 15% of the accumulated

ammonium in the cultivation vessel being due to cell lysis. The model for simulation of increase in extracellular metabolites after cell lysis is provided in the Supplementary File S3.

2.2 Impact of intracellular enzymes leaking into the supernatant

The following section describes an *in-silico* study of the possibility of intracellular enzyme leaked into supernatant after cell lysis remaining active. More specifically the possibility of amino acid degradation or conversion to glutamate and production of ammonium occurring on the supernatant. This essentially means converting the enzyme's activity on the viable cell volume scale (microscale) to volume scale of the bioreactor (macroscale). The conversion between these scales was introduced in Equation (1) in Supplementary File S1. To begin evaluation of the impact of the activity of intracellular enzymes released into the supernatant on the extracellular concentration of glutamate and ammonium, it was assumed that cell death occurs concomitantly with cell lysis. New model variables were introduced to describe the concentration of dead cells for each cell class (X_{di}, dead cells/ mL) as described in Equation (1) and the total concentration of dead cells (X_d, cells/mL) using Equation (2). The average diameter of a dead cell ($\overline{d_d}$) was calculated using Equation (3) and the dead cell-specific volume (V_s^{dc}) was calculated using Equation (5), introduced in the preceding section.

Next, the volumetric enzyme activity related to the dead cell volume ($K_e^{\max dc}$, mmol/L/min) was estimated based on the dead cell-specific volume (V_s^{dc}).

$$K_e^{\max dc} = \frac{v_e E_{level}}{V_s^{dc}} \tag{7}$$

The corresponding volumetric enzyme activity after cell lysis on the macroscopic scale ($K_e^{\max macro}$) was estimated taking into account the working volume (V_w) and the total dead cell volume ($V_s^{dc} X_d$) as shown in Equation (8) (similar to the conversion of the micro- to the macroscale described in Equation (1) in Supplementary File S1)

$$K_e^{\max macro} = K_e^{\max dc} \frac{V_s^{dc} X_d}{V_w} \quad (8)$$

To evaluate the impact of the amino acid metabolism on the production of glutamate and ammonium, the estimated amino acid degradation rate (r_{AAex} , Equation (77) in Supplementary File S1) was modified as shown in Equation (9).

$$r_{AAex}^{x} = K_{AAex}^{\max macro} b_{NAD} \Theta \quad (9)$$

Here, r_{AAex}^{x} describes the amino acid degradation rate on the supernatant after cell lysis, $K_{AAex}^{\max macro}$ is the macroscopic volumetric amino acid degradation rate, b_{NAD} is the relative NADH level, Θ is a step function which is zero for non-infected cells and one for infected cells.

The model simulations are shown in Figure 3. The model with the updated states and state variables for simulation of enzyme leakage into supernatant is provided in the Supplementary File S3.

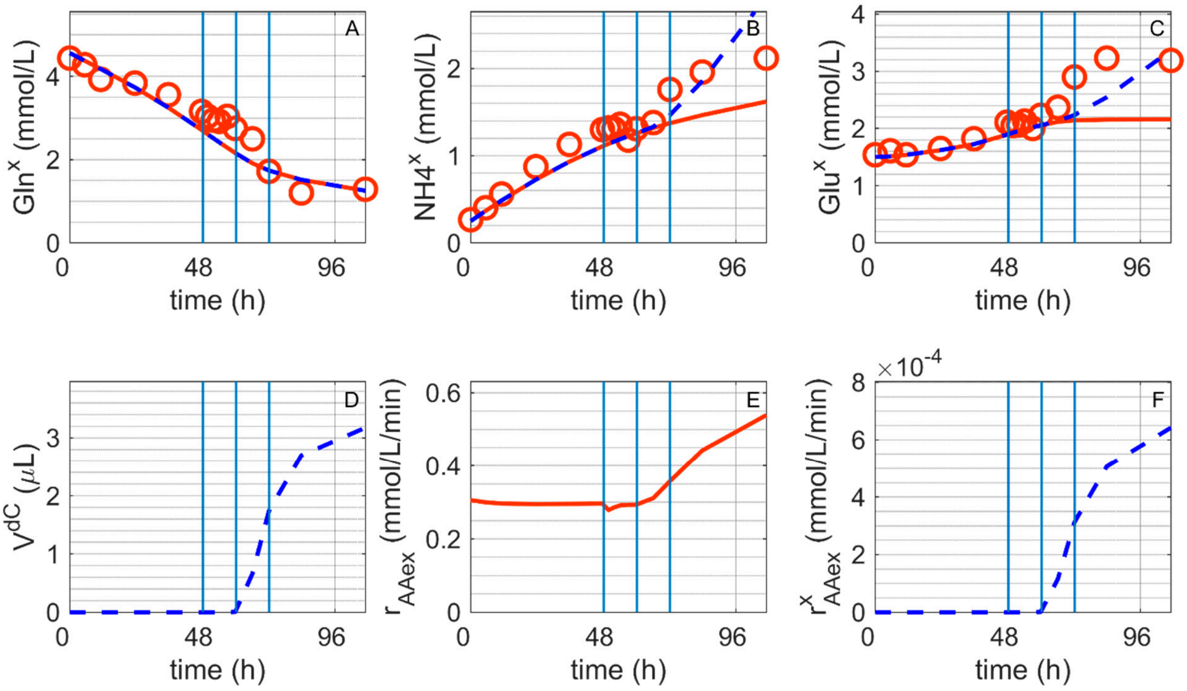

Figure 3. Concentration of extracellular metabolites before and after virus infection with (—) and without (—) leakage of enzymes related to amino acid degradation into the supernatant. (A) extracellular glutamine, (B) extracellular ammonium, (C) extracellular glutamate, (D) volume of dead cells, (E) intracellular amino acid degradation rate and (F) extracellular amino acid degradation rate. Blue vertical lines: 0, 12 and 24 hours post infection. Experimental data of Cultivation 2. Blue line: enzyme leakage (+). Red line: enzyme leakage (-). Red circles: experimental data of Cultivation 2.

These results demonstrate that accounting for extracellular amino acid degradation caused by the intracellular enzymes leakage (r^x_{AAex}, Figure 3F) has no impact on the concentration of glutamine in the supernatant (Figure 3A). On the other hand, this results in a significant increase in the concentration of ammonium and glutamate in the extracellular environment (Figure 3B, 3C). This is true even when the rate of extracellular amino acid degradation (Figure 3F) is significantly lower than the rate of intracellular amino acid degradation (r_{AAex}, Figure 3E). As can be seen, the rate of extracellular amino acid degradation is proportional to the increase in the volume of dead

cells volume (V^{dC}, Figure 3D) which would be proportional to the amount of intracellular enzyme leaked.

Overall, these results indicate that if the enzymes leaked into the supernatant after cell lysis remain active, they can have a significant impact on the concentrations of extracellular metabolite. Additional experiments should be performed to support this finding. The model for simulation with the updated states and a description of state variables that take into account cell lysis and enzyme leakage is provided in the Supplementary File S3.

3 Supplementary references

1. Rehberg, M.; Ritter, J.B.; Genzel, Y.; Flockerzi, D.; Reichl, U. The relation between growth phases, cell volume changes and metabolism of adherent cells during cultivation. *J. Biotechnol.* **2013**, *164*, 489–499, doi:10.1016/j.jbiotec.2013.01.018.
2. Rehberg, M.; Ritter, J.B.; Reichl, U. Glycolysis Is Governed by Growth Regime and Simple Enzyme Regulation in Adherent MDCK Cells. *PLoS Comput. Biol.* **2014**, *10*, e1003885, doi:10.1371/journal.pcbi.1003885.
3. Rehberg, M.; Wetzel, M.; Ritter, J.B.; Reichl, U. The regulation of glutaminolysis and citric acid cycle activity during mammalian cell cultivation. *12th IFAC Symp. Comput. Appl. Biotechnol. CAB 2013* **2013**, *12*, 48–53, doi:10.3182/20131216-3-IN-2044.00011.
4. Ramos, J.R.C.; Rath, A.G.; Genzel, Y.; Sandig, V.; Reichl, U. A dynamic model linking cell growth to intracellular metabolism and extracellular by-product accumulation. *Biotechnol. Bioeng.* **2020**, *117*, 1533–1553, doi:10.1002/bit.27288.
5. Genzel, Y.; Vogel, T.; Buck, J.; Behrendt, I.; Ramirez, D.V.; Schiedner, G.; Jordan, I.;

Reichl, U. High cell density cultivations by alternating tangential flow (ATF) perfusion for influenza A virus production using suspension cells. *Vaccine* **2014**, *32*, 2770–2781, doi:10.1016/j.vaccine.2014.02.016.

6. Gränicher, G.; Babakhani, M.; Göbel, S.; Jordan, I.; Marichal-Gallardo, P.; Genzel, Y.; Reichl, U. A high cell density perfusion process for Modified Vaccinia virus Ankara production: Process integration with inline DNA digestion and cost analysis. *Biotechnol. Bioeng.* **2021**, *118*, 4720–4734, doi:10.1002/bit.27937.
7. Nikolay, A.; Léon, A.; Schwamborn, K.; Genzel, Y.; Reichl, U. Process intensification of EB66® cell cultivations leads to high-yield yellow fever and Zika virus production. *Appl. Microbiol. Biotechnol.* **2018**, *102*, doi:10.1007/s00253-018-9275-z.
8. Bissinger, T.; Fritsch, J.; Mihut, A.; Wu, Y.; Liu, X.; Genzel, Y.; Tan, W.-S.; Reichl, U. Semi-perfusion cultures of suspension MDCK cells enable high cell concentrations and efficient influenza A virus production. *Vaccine* **2019**, *37*, 7003–7010, doi:10.1016/j.vaccine.2019.04.054.
