# Supplementary material for: Impact of Influenza A Virus Infection on Growth and Metabolism of Suspension MDCK Cells Using a Dynamic Model
Source: Metabolites. 2022 Mar 12;12(3):239. doi: 10.3390/metabo12030239 (PMC8950586; doi:10.3390/metabo12030239)
Supplement: Supplementary file 1 [file metabolites-12-00239-s001.zip › Table S1.pdf]

Table S1. Estimated global parameters of the structured intracellular model with confidence intervals between 0.025-quantile and 0.972-quantile ($Q_{0.025} – Q_{0.975}$) calculated via a bootstrap method with 2500 runs.

Parameter	Value	$Q_{0.025}$–$Q_{0.975}$	Unit	Parameter	Value	$Q_{0.025}$–$Q_{0.975}$	Unit
v_{HK}^{max}	6.25e-13	6.15e-13-1.21e-12	mmol/cell/min	v_{PK}^{max}	1.44e-12	1.25e-12-1.48e-12	mmol/cell/min
v_{GPI}^{max}	1.29e-12	8.43e-13-4.99e-12	mmol/cell/min	v_{LDH}^{max}	3.79e-09	1.73e-09-1.20e-08	mmol/cell/min
v_{G6PDH}^{max}	1.06e-14	1.03e-14-3.50e-14	mmol/cell/min	v_{PDH}^{max}	5.82e-13	1.30e-13-1.14e-12	mmol/cell/min
v_{UT}^{max}	9.86e-16	6.16e-16-1.70e-15	mmol/cell/min	v_{ACO}^{max}	3.10e-08	1.02e-08-4.99e-08	mmol/cell/min
v_{GLYS}^{max}	2.44e-17	6.32e-18-8.96e-17	mmol/cell/min	v_{CL}^{max}	1.79e-16	2.43e-17-9.12e-16	mmol/cell/min
v_{PFK}^{max}	7.88e-13	6.67e-13-9.96e-13	mmol/cell/min	v_{ICDH}^{max}	6.47e-15	2.66e-15-1.39e-14	mmol/cell/min
$v_{TATKF6P}^{max}$	3.45e-14	1.56e-14-6.17e-14	mmol/cell/min	v_{GS}^{max}	7.61e-13	1.05e-14-1.44e-12	mmol/cell/min
$v_{TATK3PG}^{max}$	7.50e-14	3.72e-14-1.18e-13	mmol/cell/min	v_{KDH}^{max}	7.90e-13	6.62e-13-9.68e-13	mmol/cell/min
v_{ALD}^{max}	3.34e-10	1.77e-10-8.27e-10	mmol/cell/min	v_{SDH}^{max}	1.48e-10	8.11e-11-3.35e-10	mmol/cell/min
v_{ENO}^{max}	2.17e-11	1.17e-11-4.00e-11	mmol/cell/min	v_{FMA}^{max}	8.45e-08	3.07e-08-1.00e-07	mmol/cell/min

Table S1. Estimated global parameters of the structured intracellular model with confidence intervals between 0.025-quantile and 0.972-quantile ($Q_{0.025}$ – $Q_{0.975}$) calculated via a bootstrap method with 2500 runs (continued).

Parameter	Value	$Q_{0.025}$–$Q_{0.975}$	Unit	Parameter	Value	$Q_{0.025}$–$Q_{0.975}$	Unit
v_{MDH}^{max}	1.97e-13	2.87e-14-3.93e-13	mmol/cell/min	v_{GLDH}^{max}	9.44e-09	5.96e-09-2.01e-08	mmol/cell/min
v_{dR5P}	2.90e-12	1.28e-12-4.85e-12	mmol/cell/min	v_{GLNase}^{max}	1.23e-11	4.99e-12-7.20e-11	mmol/cell/min
v_{ATPase}^{max}	4.52e-11	3.18e-11-7.64e-11	mmol/cell/min	v_{AlaTA}^{max}	4.69e-12	2.26e-12-1.12e-11	mmol/cell/min
v_{AAex}^{max}	4.34e-13	3.28e-13-5.80e-13	mmol/cell/min	$v_{d\mathrm{NH4}}$	8.58e-11	4.17e-11-2.24e-10	mmol/cell/min
v_{cUGLC}^{max}	1.19e-14	7.01e-15-2.29e-14	mmol/cell/min	$v_{Lac_{trans}^{x}}$	1.92e+03	4.05e+02-7.6e+03	mmol/L/µL/min
v_{CS}^{max}	4.32e-10	2.05e-10-9.39e-10	mmol/cell/min	$v_{\mathrm{Gln}_{trans}^{x}}$	2.35e-04	1.95e-04-2.72e-04	mmol/L/µL/min
v_{ME}^{max}	7.05e-13	5.53e-13-9.30e-13	mmol/cell/min	$v_{Pyr_{trans}^{x}}$	4.31e-04	3.90e-04-5.12e-04	mmol/L/µL/min
v_{PEPCK}^{max}	2.84e-13	1.12e-13-3.36e-13	mmol/cell/min	$v_{\mathrm{NH4}_{trans}^{x}}$	4.25e-04	1.09e-04-2.99e-03	mmol/L/µL/min
v_{PC}^{max}	1.55e-12	5.36e-13-2.40e-12	mmol/cell/min	$v_{Glu_{trans}^{x}}$	4.70e-05	2.15e-05-1.01e-04	mmol/L/µL/min
v_{AspTA}^{max}	9.54e-09	3.44e-09-3.11e-08	mmol/cell/min	k_{HK}^{m}	5.01e-04	2.00e-04-	mmol/L

Table S1. Estimated global parameters of the structured intracellular model with confidence intervals between 0.025-quantile and 0.972-quantile ($Q_{0.025} – Q_{0.975}$) calculated via a bootstrap method with 2500 runs (continued).

Parameter	Value	$Q_{0.025}$–$Q_{0.975}$	Unit	Parameter	Value	$Q_{0.025}$–$Q_{0.975}$	Unit
k_{GPI}^{m}	7.97e-02	1.37e-02-3.25e-01	mmol/L	$k_{Pyr_{PK}}$	3.21e+03	8.00e+02-8.29e+03	mmol/L
k_{GPI}^{eq}	1.55e+00	5.63e-01-6.12e+02	-	$k_{Pyr_{PDH}}$	4.97e-07	1.56e-07-1.31e-06	mmol/L
k_{G6PDH}^{m}	3.39e-03	9.91e-04-9.19e-03	mmol/L	k_{ACO}^{eq}	1.46e-02	1.28e-02-1.61e-02	-
k_{UT}^{m}	7.23e-02	1.33e-02-1.82e-01	mmol/L	k_{ACO2}^{eq}	2.14e+00	1.90e+00-2.38e+00	-
$k_{TATKF6P}^{eq}$	7.01e-01	2.03e-01-1.73e+00	-	k_{Cit}^{m}	7.83e+01	1.32e+01-2.75e+02	mmol/L
$k_{TATK3PG}^{eq}$	6.63e-06	1.09e-06-4.82e-05	-	k_{ICDH}^{eq}	2.33e+01	1.21e+01-3.30e+01	-
k_{PFK}^{m}	5.67e-03	3.12e-03-1.13e-02	mmol/L	k_{AAex}^{eq}	6.72e+05	1.64e+05-9.00e+05	-
k_{F16P}	1.55e-02	4.99e-03-3.12e-02	mmol/L	k_{FMA}^{eq}	7.71e+00	5.63e+00-3.66e+03	-
k_{ENO}^{eq}	4.17e+02	1.20e+02-3.66e+03	-	k_{FMA}^{m}	5.54e+03	1.49e+03-1.72e+04	mmol/L
$k_{PEP_{PK}}$	1.64e-03	8.58e-04-2.36e-03	mmol/L	k_{MDH}^{m}	3.25e+00	6.91e-01-	mmol/L

Table S1. Estimated global parameters of the structured intracellular model with confidence intervals between 0.025-quantile and 0.972-quantile ($Q_{0.025} – Q_{0.975}$) calculated via bootstrap method with 2500 runs (continued).

Parameter	Value	$Q_{0.025}$–$Q_{0.975}$	Unit	Parameter	Value	$Q_{0.025}$–$Q_{0.975}$	Unit
k_{xATP}	3.00e+02	2.00e+02-4.79e+02	cell/L/min	k^{m}_{OAA}	2.31e-04	1.92e-05-6.18e-04	mmol/L
k_{mATP}	1.33e+07	1.00e+07-8.81e+07	cell/L/min	k^{m}_{AcCoA}	1.10e+05	4.29e+04-3.05e+05	mmol/L
NAD_{basal}	2.33e+00	1.26e+00-3.55e+00	mmol/L	k^{m}_{SDH}	4.07e+00	2.78e-03-6.39e+00	mmol/L
k^{m}_{cUGLC}	4.07e+00	1.97e+00-6.39e+00	mmol/L	k^{m}_{dR5P}	5.25e-01	1.96e-01-9.22e-01	mmol/L
k^{m}_{ME}	2.27e-01	1.53e-01-4.49e-01	mmol/L	k^{m}_{PEPCK}	3.93e-09	8.42e-10-	mmol/L
$k^{i}_{ATP_{ME}}$	6.89e+04	1.90e+04-1.99e+05	$mmol^2/L^2$	$k^{m}_{Pyr_{PC}}$	7.62e-02	9.36e-03-1.24e-01	mmol/L
k^{i}_{μ}	1.31e+03	3.87e+02-2.75e+03	mmol/L	k^{eq}_{ME}	1.84e+04	4.28e+02-6.93e+04	-
$k^{i}_{Glu_{AlaTA}}$	1.89e-01	9.75e-02-3.61e-01	$mmol^2/L^2$	k^{m}_{KDH}	1.14e-01	7.42e-02-2.06e-01	mmol/L
$k^{m}_{Glu_{GS}}$	2.33e-03	8.16e-04-2.51e-02	mmol/L	$k_{OAA_{AspTA}}$	1.15e+03	2.89e+02-3.05e+03	mmol/L
k^{m}_{ICDH}	1.52e-07	5.07e-08-5.63e-07	mmol/L	$k_{Glu_{AspTA}}$	2.70e-03	7.99e-04-	mmol/L

Table S1. Estimated global parameters of the structured intracellular model with confidence intervals between 0.025-quantile and 0.972-quantile ($Q_{0.025}$ – $Q_{0.975}$) calculated via a bootstrap method with 2500 runs (continued).

Parameter	Value	$Q_{0.025}$–$Q_{0.975}$	Unit	Parameter	Value	$Q_{0.025}$–$Q_{0.975}$	Unit
$k_{Keto_{AspTA}}$	1.93e+00	4.91e-01-4.57e+05	mmol/L	$k^{m}_{TATKF6P}$	4.41e-07	1.10e-07-1.60e-06	mmol/L
$k_{Glu_{GLDH}}$	6.27e-05	2.57e-05-9.71e-05	mmol/L	k^{m}_{GLDH}	3.32e+04	1.44e+04-6.37e+04	mmol/L
k^{m}_{Glnase}	1.69e-04	2.99e-05-2.59e-02	mmol/L	$k^{m}_{ATP_{HK}}$	2.36e-06	7.20e-07-7.32e-06	mmol/L
k^{eq}_{GLDH}	4.51e-01	1.25e-01-1.71e+00	-	$k^{m}_{ATP_{ALD}}$	7.40e+04	1.93e+04-2.31e+05	mmol/L
k^{eq}_{ALD}	8.95e+03	2.59e+03-4.02e+04	-	$k^{m}_{3PG_{ALD}}$	1.47e-04	4.57e-05-2.99e-04	mmol/L
k^{a}_{PFK}	3.39e+00	2.88e+00-4.28e+00	mmol/L	$k^{m}_{ATP_{PC}}$	4.06e-09	4.39e-10-1.16e-08	mmol/L
k^{m}_{ATPase}	1.37e+01	8.79e+00-2.24e+01	mmol/L	$k^{m}_{ATP_{PDH}}$	3.94e+02	2.16e+02-7.31e+02	mmol/L
k_{3PG}	1.35e+00	6.39e-01-2.94e+00	mmol/L	$k_{Glu^{x}}$	2.47e+01	1.46e+01-4.57e+01	mmol/L
$k_{PEP_{ENO}}$	1.01e-02	3.53e-03-1.90e-02	mmol/L	k_{Glu}	2.00e+03	4.71e+02-1.03e+04	mmol/L
$k^{m}_{TATK3PG}$	6.59e-09	5.14e-10-2.77e-08	mmol/L	$k^{eq}_{Glu^{x}_{trans}}$	3.56e-05	4.05e-06-9.25e-05	-

Table S1. Estimated global parameters of the structured intracellular model with confidence intervals between 0.025-quantile and 0.972-quantile ($Q_{0.025} – Q_{0.975}$) calculated via a bootstrap method with 2500 runs (continued).

Parameter	Value	$Q_{0.025}–Q_{0.975}$	Unit	Parameter	Value	$Q_{0.025}–Q_{0.975}$	Unit
$k_{G\ln^x}$	1.05e+01	4.63e+00-4.86e+01	mmol/L	$k_{Lac_{trans}}$	2.73e+00	1.46e+00-7.52e+00	mmol/L
$k^m_{Pyr^x_{trans}}$	2.55e-04	3.24e-05-1.07e-03	mmol/L	$k_{Lac^x_{trans}}$	7.97e+01	2.68e+01-1.42e+02	mmol/L
ω	8.57e-06	2.66e-06-2.20e-05	-	$k_{Pyr_{LDH}}$	6.37e-02	3.21e-02-1.24e-01	mmol/L
k^m_{AspTA}	4.25e+03	2.81e+02-1.14e+04	mmol/L	$k_{Lac_{LDH}}$	4.39e-02	2.71e-02-1.01e-01	mmol/L
$k^m_{ATP_{GS}}$	1.88e+01	1.05e+01-1.17e+02	mmol/L	k_{cPyr}	9.10e-06	1.48e-06-5.27e-05	$mmol^2/L^2$
$k^i_{ATP_{G\ln ase}}$	2.81e-02	1.16e-02-7.50e-02	$mmol^2/L^2$	$k^a_{Pyr_{LDH}}$	3.07e-05	1.07e-05-9.84e-05	-
$k^i_{OAA_{SDH}}$	1.56e-01	6.11e-02-2.85e-01	$mmol^2/L^2$	$k_{Glu_{LDH}}$	2.08e+01	8.97e+00-6.08e+01	$mmol^2/L^2$
k^m_{GLYS}	8.96e+05	1.95e+05-9.00e+05	mmol/L	$k^i_{Glu_{LDH}}$	1.73e+01	6.41e+00-9.50e+01	-
$k^m_{ATP_{CL}}$	3.08e+04	3.47e+03-9.44e+04	mmol/L	k^{eq}_{LDH}	1.01e+01	5.26e+00-1.84e+01	-
$k^{eq}_{Lac^x_{trans}}$	3.73e+01	1.88e+01-5.88e+01	-	$k^m_{dN\mathrm{H4}}$	8.12e+02	2.48e+02-1.65e+03	mmol/L

Table S1. Estimated global parameters of the structured intracellular model with confidence intervals between 0.025-quantile and 0.972-quantile ($Q_{0.025} – Q_{0.975}$) calculated via a bootstrap method with 2500 runs (continued).

Parameter	Value	$Q_{0.025}$–$Q_{0.975}$	Unit	Parameter	Value	$Q_{0.025}$–$Q_{0.975}$	Unit
$k^{a}_{ATP_{d\mathrm{NH4}}}$	5.27e-03	1.35e-03-3.66e-02	mmol/L	k_{NH4}	1.92e-02	7.14e-03-3.36e-02	mmol/L
$k_{\mathrm{NH4}^{x}}$	2.45e+00	1.22e+00-7.33e+00	mmol/L	k^{m}_{AlaTA}	1.68e-01	7.59e-02-4.91e-01	mmol/L
$k^{eq}_{\mathrm{NH4}^{x}_{trans}}$	7.46e-06	3.51e-06-2.28e-05	-				
