# Supplementary material for: Impact of Influenza A Virus Infection on Growth and Metabolism of Suspension MDCK Cells Using a Dynamic Model
Source: Metabolites. 2022 Mar 12;12(3):239. doi: 10.3390/metabo12030239 (PMC8950586; doi:10.3390/metabo12030239)
Supplement: Supplementary file 1 [file metabolites-12-00239-s001.zip › Table S2.pdf]

Table S2. Estimated global parameters of the segregated cell growth model with confidence intervals between 0.025-quantile and 0.975-quantile ($Q_{0.025} – Q_{0.975}$) calculated via a bootstrap method with 2500 runs.

Parameter	Value	$Q_{0.025}$–$Q_{0.975}$	Unit	Parameter	Value	$Q_{0.025}$–$Q_{0.975}$	Unit
$k^{m}_{Glc^x}$	9.78e+00	4.52e+00-1.25e+01	mmol/L	α	3.66e+05	3.08e+05-5.93e+05	-
$\mu_{\max}$	4.20e-04	3.46e-04-4.73e-04	1/min	$k_d^{\min}$	1.93e-12	1.93e-14-3.27e-05	1/min
Y_{x/Glc^x}	6.02e-09	4.96e-09-1.32e-06	mmol/L/cell	$k_d^{\max}$	9.48e-05	6.66e-05-1.02e-04	1/min
m_{Glc^x}	4.28e-04	1.70e-04-4.49e-04	mmol/L/µL/min	β	1.46e-19	1.46e-21-1.02e-05	1/min
