# Supplementary material for: Impact of Influenza A Virus Infection on Growth and Metabolism of Suspension MDCK Cells Using a Dynamic Model
Source: Metabolites. 2022 Mar 12;12(3):239. doi: 10.3390/metabo12030239 (PMC8950586; doi:10.3390/metabo12030239)
Supplement: Supplementary file 1 [file metabolites-12-00239-s001.zip › Table S3.pdf]

Table S3A. Initial conditions of substrates and metabolic by-products with local parameters used for the simulation of Cultivation 1 (Cult1) and Cultivation 2 (Cult2).

	Cult1	Cult2	Unit		Cult1	Cult2	Unit
$\left[Glc^x\right]\ (t=0)$	19.50	19.50	mmol/L	E_{Level}	1.00	1.00	-
$\left[G\ln^x\right]\ (t=0)$	4.65	4.56	mmol/L	V_w	1e-3	1e-3	-
$\left[Glu^x\right]\ (t=0)$	1.50	1.50	mmol/L	$X_1(t=0)$	2.19e+05	2.35e+05	cells/mL
$\left[Lac^x\right]\ (t=0)$	0.00	0.00	mmol/L	$X_2(t=0)$	1.49+05	1.57+05	cells/mL
$\left[NH4^x\right]\ (t=0)$	0.25	0.25	mmol/L	$X_3(t=0)$	1.50+05	1.58+05	cells/mL
$\left[Pyr^x\right]\ (t=0)$	7.66	7.86	mmol/L	$X_4(t=0)$	1.30+05	1.30+05	cells/mL
d_c	19.58	19.58	µm	$X_5(t=0)$	1.12+05	1.12+05	cells/mL
d_m	9.76	9.96	µm	$[V_t]\ (t=48.1)$	-	1.71e08	Virions/mL
k_{dGln^x}	8.8e-05	8.8e-05	1/min	$\rho 1$	-	4.85	-
$\rho 2$	-	15.61	-	$\rho 3$	-	6.00	-
k_d^{mininf}	-	4.9e-08	1/min	k_d^{maxinf}	-	2.9e-04	1/min
v_p	-	6.45e+00	virions/cell/min				

Table S3B. Initial conditions used for simulation of the pre-culture of Cultivation 1 (Cult1) and Cultivation 2 (Cult2).

	**Pre-culture**	**Unit**		**Pre-culture**	**Unit**
$X_1(t=0)$	1.49+05	cells/mL	$[G6P]\ (t=0)$	0.12	mmol/L
$X_2(t=0)$	1.50+05	cells/mL	$[F6P]\ (t=0)$	0.037	mmol/L
$X_3(t=0)$	1.30+05	cells/mL	$[R5P]\ (t=0)$	0.016	mmol/L
$X_4(t=0)$	1.12+05	cells/mL	$[UDPGlc]\ (t=0)$	0.19	mmol/L
$X_5(t=0)$	1.49+05	cells/mL	$[F16P]\ (t=0)$	0.035	mmol/L
$[Glc^x]\ (t=0)$	19.50	mmol/L	$[3PG]\ (t=0)$	0.15	mmol/L
$[G\ln^x]\ (t=0)$	4.65	mmol/L	$[PEP]\ (t=0)$	0.013	mmol/L
$[Glu^x]\ (t=0)$	1.50	mmol/L	$[Pyr]\ (t=0)$	0.036	mmol/L
$[Lac^x]\ (t=0)$	0.00	mmol/L	$[Lac]\ (t=0)$	0.39	mmol/L
$[NH4^x]\ (t=0)$	0.25	mmol/L	$[AcCoA]\ (t=0)$	0.99	mmol/L
$[Pyr^x]\ (t=0)$	7.66	mmol/L	$[IsoCit]\ (t=0)$	0.023	mmol/L
$[Glc]\ (t=0)$	0.019	mmol/L	$[Gln]\ (t=0)$	1.49	mmol/L

Table S3B. Initial conditions used for simulation of the pre-culture of Cultivation 1 (Cult1) and Cultivation 2 (Cult2) (Continued).

	**Pre-culture**	**Unit**		**Pre-culture**	**Unit**
$[Glu]\ (t=0)$	1.89	mmol/L	$[Keto]\ (t=0)$	0.17	mmol/L
$[Suc]\ (t=0)$	0.019	mmol/L	$[Fum]\ (t=0)$	0.086	mmol/L
$[Mal]\ (t=0)$	0.42	mmol/L	$[OAA]\ (t=0)$	0.24	mmol/L
$[NH4]\ (t=0)$	5.98	mmol/L	$[ATP]\ (t=0)$	2.53	mmol/L
