# Supplementary material for: Impact of Influenza A Virus Infection on Growth and Metabolism of Suspension MDCK Cells Using a Dynamic Model
Source: Metabolites. 2022 Mar 12;12(3):239. doi: 10.3390/metabo12030239 (PMC8950586; doi:10.3390/metabo12030239)
Supplement: Supplementary file 1 [file metabolites-12-00239-s001.zip › Table S4.pdf]

Table S4A. Experimental procedures. Solutions used for washing, quenching and metabolite extraction.

Name	Solvent (v/v)	Additives (f.c.)	Temperature
**Wash solution**	MilliQ-water (100 %)	NaCl (9.0 g/L)	4 °C
**Quench solution**	Methanol (66.7 %)<br>Chloroform (33.3 %)	-	-20 °C
**Extraction solution**	Methanol (47.4 %)<br>MilliQ-water (52.6 %)	Tricine (2 mM)	4 °C
**MeOH-AMBIC**	Methanol (60.0 %)<br>MilliQ-water (40.0 %)	AMBIC (8.5 g/L)<br>HCl (pH adjust)	4/-40 °C
**Chloroform**	Chloroform (100 %)	-	-20 °C

Table S4B. Experimental procedures. KOH-gradient for the separation of intracellular metabolites by AE-HPLC.

Time [min]	KOH [mM]	Curve [1]		Time [min]	KOH [mM]	Curve [1]
**0 [2]**	2	5		**33**	50	5
**5**	6	5		**41**	86	5
**10**	10	3		**41**	100	5
**15**	14	3		**46**	100	5
**18**	22	1		**46**	2	5
**27**	30	7		**53**	2	5

[1] Curves 1 through 4 are convex, curve 5 is linear and curves 6 through 9 are concave; [2] time of injection.

Table S4C. Experimental procedures. Dilutions used for the external standards for quantitative LC-MS measurements.

STD ID	Tricine-buffer	Metabolite-MM [1]	Water	e.g. [ATP] [2]
**155**	845 µL	155 µL	–	310 µM
**130**	845 µL	130 µL	25 µL	260 µM
**105**	845 µL	105 µL	50 µL	210 µM
**080**	845 µL	80 µL	75 µL	160 µM
**055**	845 µL	55 µL	100 µL	110 µM
**030**	845 µL	60 µL (1:2)	95 µL	60 µM
**020 [3]**	845 µL	40 µL (1:2)	115 µL	40 µM
**010 [3]**	845 µL	100 µL (1:10)	55 µL	20 µM
**005**	845 µL	50 µL (1:10)	105 µL	10 µM

[1] Composition of the metabolite master mix can be found in Table 1E in S4 Table; [2] Exemplary concentration of ATP for respective standard; [3] standards not used for method validation.

Table S4D. Experimental procedures. Overview of single ion monitoring channels for metabolite quantification by MS.

Name	Mass	Span	Time range (min)	Dwell time	Polarity	Cone (V)
**Sim 2: Fum**	115.0	1.0	16.0-18.5	0.1	-ve	30
**Sim 3: Suc**	117.0	1.0	12.0-15.0	0.1	-ve	30
**Sim 4: Aconitate**	129.2	1.0	28.5-33.0	0.1	-ve	50
**Sim 5 Mal**	133.0	1.0	12.5-15.0	0.1	-ve	35
**Sim 6: α-KG**	145.1	1.0	15.0-17.5	0.1	-ve	30
**Sim 7: PEP**	166.8	1.0	28.0-31.0	0.1	-ve	35
**Sim 8: 3-PG**	185.2	1.0	24.0-26.5	0.1	-ve	45
**Sim 9: Cit**	191.3	1.0	25.5-29.0	0.1	-ve	35
**Sim 10: R5P**	229.2	1.0	18.0-20.5	0.1	-ve	45
**Sim 15: F16BP**	339.1	1.0	35.0-37.5	0.1	-ve	30
**Sim 19: UDP-Glc**	502.0	1.0	30.0-30.0	0.1	-ve	30

[1] …

Table S4E. Experimental procedures. Metabolite standard mix used for LC-MS analysis.

Category	Substance Name	Supplier	Product Number	CAS-Number	Lot Purity [%]	Conc. Master-Mix [µM]	Conc. Std 155 [µM]	Conc. Std 005 [µM]
Additives	Sodium acetate	Sigma-Aldrich	S8750-250G	127-09-3	99.6	100	15.5	0.5
Additives	Sodium chloride	Roth	P029.3	7647-14-5	100	100	15.5	0.5
Additives	Sodium formate	Sigma-Aldrich	17841-50G	141-53-7	100	100	15.5	0.5
Additives	Sodium nitrate	Merck	1065371000	7631-99-4	99.5	100	15.5	0.5
Additives	Sodium sulfate	Roth	8631.2	7757-82-6	99	800	124	4
Nucleo-sugars	Disodium uridine-diphospho-N-acetylgalactosamine	Sigma-Aldrich	U5252-5MG	108320-87-2	100	60	9.3	0.3
Nucleo-sugars	Disodium uridine-diphospho-N-acetylglucosamine	Sigma-Aldrich	U4375-100MG	91183-98-1	99	200	31	1
Nucleo-sugars	Disodium uridine-diphosphoglucose hydrate	Sigma-Aldrich	U4625-100MG	28053-08-9	100	100	15.5	0.5
Nucleotides	Sodium adenosine-diphosphate	Sigma-Aldrich	A2754-100MG	20398-34-9	99	150	23.25	0.75
Nucleotides	Sodium adenosine-monophosphate	Sigma-Aldrich	01930-5G	4578-31-8	99.7	25	3.875	0.125
Nucleotides	Disodium adenosine-triphosphate hydrate	Sigma-Aldrich	A2383-1G	34369-07-8	99	2000	310	10
Nucleotides	Sodium cytidine-diphosphate	Sigma-Aldrich	C9755-25MG	34393-59-4	95	12.5	1.9375	0.0625
Nucleotides	Disodium cytidine-monophosphate	Sigma-Aldrich	C1006-500MG	6757-06-8	100	10	1.55	0.05
Nucleotides	Disodium cytidine-triphosphate	Sigma-Aldrich	C1506-100MG	36051-68-0	95.3	100	15.5	0.5
Nucleotides	Sodium guanosine-diphosphate	Sigma-Aldrich	G7127-100MG	43139-22-6	98	40	6.2	0.2
Nucleotides	Disodium guanosine-monophosphate hydrate	Sigma-Aldrich	G8377-500MG	5550-12-9	99.9	12.5	1.9375	0.0625
Nucleotides	Sodium guanosine-triphosphate hydrate	Sigma-Aldrich	G8877-25MG	36051-31-7	96	240	37.2	1.2
Nucleotides	Disodium uridine-diphosphate hydrate	Sigma-Aldrich	94330-100MG	27821-45-0	98.8	25	3.875	0.125
Nucleotides	Disodium uridine-monophosphate	Sigma-Aldrich	U6375-1G	3387-36-8	100	40	6.2	0.2
Nucleotides	Trisodium uridine-triphosphate hydrate	Sigma-Aldrich	U6625-100MG	19817-92-6	96	500	77.5	2.5
Organic acids	D-(−)-3-Phosphoglyceric acid disodium salt	Sigma-Aldrich	P8877-10MG	80731-10-8	93	50	7.75	0.25
Organic acids	cis-Aconitic acid	Sigma-Aldrich	A3412-1G	585-84-2	99	25	3.875	0.125
Organic acids	Citric acid monohydrate	Sigma-Aldrich	C1909-25G	5949-29-1	100	200	31	1
Organic acids	Fumaric acid	Sigma-Aldrich	F19353-25G	110-17-8	99.7	25	3.875	0.125
Organic acids	Trisodium isocitric acid hydrate	Sigma-Aldrich	I1252-1G	1637-73-6	99	20	3.1	0.1
Organic acids	Sodium L-lactate	Sigma-Aldrich	L7022-5G	867-56-1	99.8	160	24.8	0.8
Organic acids	DL-Malic acid	Sigma-Aldrich	M0875-100G	6915-15-7	99	250	38.75	1.25
Organic acids	Potasium phophoenolpyruvate	Applichem	A2271,0250	4265-07-0	98	25	3.875	0.125
Organic acids	Sodium pyruvate	Sigma-Aldrich	P8574-100G	113-24-6	100	200	31	1
Organic acids	Disodium succinate	Sigma-Aldrich	224731-5G	150-90-3	99.9	100	15.5	0.5
Organic acids	α-Ketoglutaric acid	Sigma-Aldrich	K1750-100G	328-50-7	100	100	15.5	0.5
Phospo-sugars	Trisodium D-fructose-1,6-bisphospohate hydrate	Sigma-Aldrich	F6803-10MG	38099-82-0	100	150	23.25	0.75
Phospo-sugars	Fructose-1 Phosphate Sodium salt	Sigma-Aldrich	sc-214802	53823-70-4	97	35	5.425	0.175
Phospo-sugars	Disodium fructose-6-phosphate hydrate	Sigma-Aldrich	F3627-100MG	26177-86-6	100	25	3.875	0.125
Phospo-sugars	Disodium α-D-glucose-1-phosphate hydrate	Sigma-Aldrich	G7000-1G	56401-20-8	100	35	5.425	0.175
Phospo-sugars	Sodium α-D-glucose-6-phosphate	Sigma-Aldrich	G7879-500MG	54010-71-8	100	35	5.425	0.175
Phospo-sugars	Disodium ribose-5-Phosphate dihydrate	Sigma-Aldrich	83875-250MG	207671-46-3	100	25	3.875	0.125
