# Supplementary material for: Impact of Influenza A Virus Infection on Growth and Metabolism of Suspension MDCK Cells Using a Dynamic Model
Source: Metabolites. 2022 Mar 12;12(3):239. doi: 10.3390/metabo12030239 (PMC8950586; doi:10.3390/metabo12030239)
Supplement: Supplementary file 1 [file metabolites-12-00239-s001.zip › Supplementary File S3.zip!/S3_file/Demo_MDCK/SBPOP_PACKAGE_Rev_1176_64bit/SBPD/_ACADEMIC/tasks/gui/SBPDgui_dir/documentation/about.html]

# About SBPD

The SBTOOLBOX2 and the SBPD addon package have been developed
by Dr. Henning Schmidt,
currently working at the University of Rostock, Germany.

In case of questions, feedback, employment offers, lottery-win notifications,
etc. please contact me via henning@sbtoolbox2.org.

# License Information SBPD

SBPD is licensed under a  
Creative Commons Attribution-Non-Commercial-No Derivative Works 2.0 Germany License.
