# Supplementary material for: Impact of Influenza A Virus Infection on Growth and Metabolism of Suspension MDCK Cells Using a Dynamic Model
Source: Metabolites. 2022 Mar 12;12(3):239. doi: 10.3390/metabo12030239 (PMC8950586; doi:10.3390/metabo12030239)
Supplement: Supplementary file 1 [file metabolites-12-00239-s001.zip › Supplementary File S3.zip!/S3_file/Demo_MDCK/SBPOP_PACKAGE_Rev_1176_64bit/SBPD/_ACADEMIC/tasks/gui/SBPDgui_dir/documentation/documentation.html]

# Documentation SBPDgui

**The SBPGgui is a graphical user-interface aiming at giving users,
which are unfamiliar with the MATLAB command line interface, the possibility of using
the SBPD package for parameter estimation tasks.**

Its use is hopefully (almost) self-explaining. Essentially, the main steps are the following:

1. *Load* (or *Import*) a project using the *File* menu.- Choose the parameters/initial conditions you want to estimate. You need to specify their names and
     lower and upper bounds.
     - Global parameters are model parameters that are the same across all experiments.- Local parameters are experiment dependent.- Initial conditions are always estimated experiment dependent.
     The *Initialize* and *Write out* buttons help with this task. Try them out and you will see
     what they do. Copy and paste and change as you need it.- Select desired estimation settings.- Run the parameter estimation.- Following the parameter estimation you can perform a fit analysis. A good idea is to select the
           *simplexSB* or *SSmSB* (if availabe) optimization methods for that, using smaller settings
           for the maximum number of iterations.- Export or save the project using again the *File* menu.

At all times other tasks can be run from the buttons to the left. Just try them out.

Please also have a look at the full SBPD documentation that
you can find here: SBPD Documentation.
