# Supplementary material for: Impact of Influenza A Virus Infection on Growth and Metabolism of Suspension MDCK Cells Using a Dynamic Model
Source: Metabolites. 2022 Mar 12;12(3):239. doi: 10.3390/metabo12030239 (PMC8950586; doi:10.3390/metabo12030239)
Supplement: Supplementary file 1 [file metabolites-12-00239-s001.zip › Supplementary File S3.zip!/S3_file/Demo_MDCK/SBPOP_PACKAGE_Rev_1176_64bit/SBTOOLBOX2/_ACADEMIC/tasks/analysis/globalparametersensitivity/auxiliary/sampling/LHS.pdf]

Sampling methods for uncertainty analysis

Simple random sampling

Simple random sampling involves repeatedly forming random vectors of parameters from prescribed probability distributions. A normally-distributed random variable x with mean μ and standard deviation σ can be generated by:

$$x^* = \sigma \mathrm{r_n} + \mu$$

where $\mathrm{r_n}$ are normally distributed random numbers with mean 0 and variance 1.

A multivariate normal distribution with variance-covariance matrix **V** can be sampled utilising the lower and upper triangular matrix (LU) decomposition method (Davis, 1987). The variance-covariance matrix **V** is first decomposed by Cholesky factorization:

$$\mathbf{V} = \mathbf{L}\,\mathbf{L}^{\mathrm{T}}$$

where **L** is the lower triangular matrix. To generate the random variables vector **x**, matrix **L** is multiplied by vector, $\mathbf{r_n}$, of independent normal random numbers with mean 0 and variance 1:

$$\mathbf{x} = \mathbf{L}\,\mathbf{r_n} + \boldsymbol{\mu}\ .$$

The procedure is repeated for sample size *ns*, resulting in a set of variables with expected mean vector $\boldsymbol{\mu}$ and expected variance-covariance matrix: $\mathbf{L}\ \mathrm{cov}(\mathbf{r_n})\ \mathbf{L}^{\mathrm{T}}$. Since the random numbers are independent, the covariance matrix $\mathrm{cov}(\mathbf{r_n})$ should equal **I** (the identity matrix),

$$\mathbf{L}\ \mathrm{cov}(\mathbf{r_n})\ \mathbf{L}^{\mathrm{T}} = \mathbf{L}\,\mathbf{I}\,\mathbf{L}^{\mathrm{T}} = \mathbf{L}\,\mathbf{L}^{\mathrm{T}} = \mathbf{V}\ .$$

Latin hypercube sampling

Latin hypercube sampling (LHS), a stratified-random procedure, provides an efficient way of sampling variables from their distributions (Iman and Conover, 1980). The LHS involves sampling *ns* values from the prescribed distribution of each of k variables x_1, x_2, … x_k. The cumulative distribution for each variable is divided into N equiprobable intervals. A value is selected randomly from each interval (Figure 1). The N values obtained for each variable are paired randomly with the other variables. Unlike simple random sampling, this method ensures a full coverage of the range of each variable by maximally stratifying each marginal distribution.

The LHS can be summarized as:

- divide the cumulative distribution of each variable into N equiprobable invervals;
- from each interval select a value randomly, for the ith interval, the sampled cumulative probability can be written as (Wyss and Jorgensen, 1998):

  $$\mathrm{Prob}_i = (1/N)\ \mathrm{r_u} + (i-1)/N$$

  where $\mathrm{r_u}$ is uniformly distributed random number ranging from 0 to 1;

- transform the probability values sampled into the value *x* using the inverse of the distribution function F^{-1}:

  $x = F^{-1}(\text{Prob})$;
- the *N* values obtained for each variable *x* are paired randomly (equally likely combinations) with the *ns* values of the other variables.

The method is based on the assumption that the variables are independent of each other, but in reality most of the input variables are correlated to some extent. Random pairing of correlated variables could result in impossible combinations, furthermore independent variables tend to bias the uncertainty.

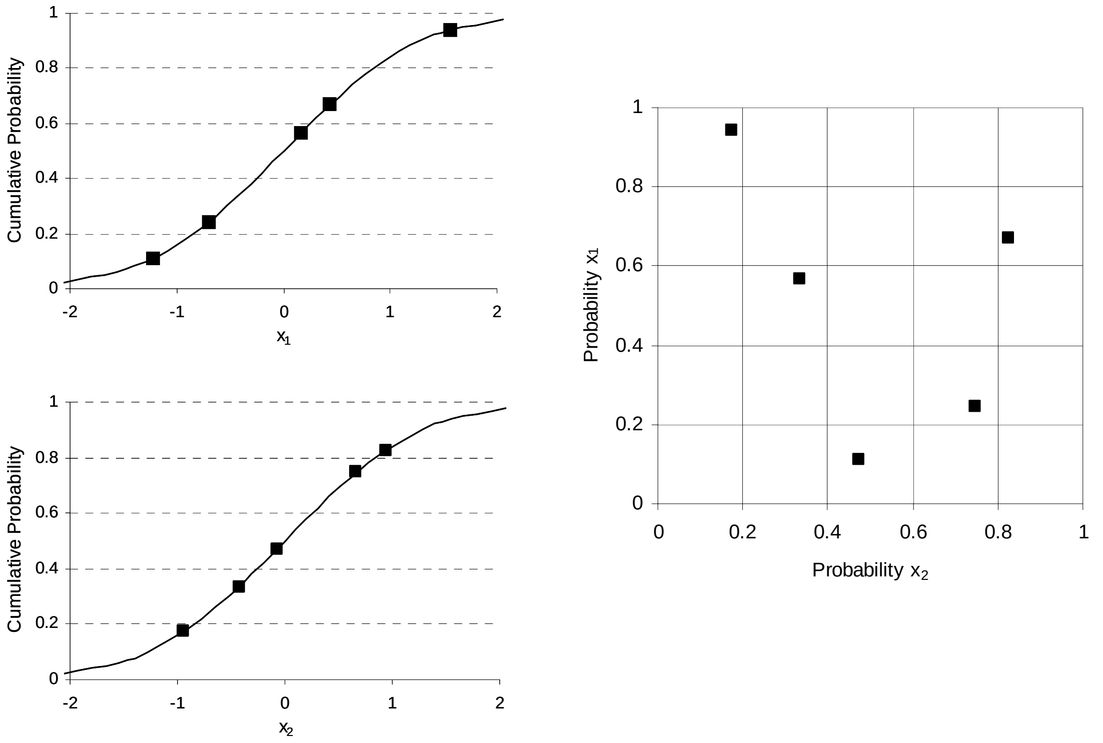

Fig. 1. Example of LHS: Random stratified sampling of variables x_1 and x_2 at 5 intervals (Left) and random pairing of sampled x_1 and x_2 forming a Latin hypercube (Right).

Inducing correlation in Latin hypercube sampling

Iman and Conover (1982) proposed a method for inducing correlation among the variables by restricting the way the variables are paired based on the rank correlation of some target values. The method is based on the Cholesky decomposition of the correlation matrix. Suppose matrix **X** is composed of independent random variables with correlation matrix **I** and **C** is the desired correlation matrix. Matrix **C** can be written as **C** = **P P**' where **P** is the lower triangular matrix. Similar to the simple random sampling, multiplying vector **x P'** yield random variables with correlation matrix **C**. Therefore the objective is to rearrange the input variables close to the target correlation matrix. The method is summarised as follows:

- generate matrix **R** using Latin hypercube sampling of *k* variables at sample size *ns*;
- calculate **T**, the correlation matrix of **R**;
- calculate the **P** lower triangular matrix of the target correlation matrix **C** using Cholesky factorization **C** = **P P**' and also **Q** the lower triangular matrix of **T**

  T = **Q Q**' ;
- solve to obtain matrix **S** such that **STS**' = **C**, which is calculated from **S** = **P** $\mathbf{Q}^{-1}$;
- calculate target correlation matrix **R*** = **R S**', which has a correlation matrix equal to **C**;
- rearrange the values of each variable in **R** so they have the same rank (order) as the target matrix **R***.

Stein (1987) also proposed a method for sampling dependent variables based on the rank of a target multivariate distribution.

- obtain matrix **R** of *k* variables at sample size *N* using simple random sampling;
- define **U** the matrix with *k* columns and *N* rows containing the order or rank corresponding to the target correlation matrix;
- obtain the Latin hypercube sample x_{ij} (i = 1, ..., N; j = 1, ..., k) by

$$x_{ij} = F_j^{-1}\left(\frac{u_{ij} - \mathrm{r_u}}{N}\right)$$

With this shifting (transformation), the sampled values yield an approximately joint distribution. See Pebesma and Heuvelink (1999) and Zhang and Pinder (2004) for more detail description.

References

Cochran, W.G., 1977. *Sampling Techniques*, 3rd edn. John Wiley and Sons, New York.

Davis, M.W., 1987. Production of conditional simulations via the LU triangular decomposition of the covariance matrix. *Mathematical Geology* 19, 91-98.

Iman, R.L., Conover, W.J., 1982. A distribution-free approach to inducing rank correlation among input variables. *Communications in Statistics* B11, 311-334.

Iman, R.L., 1992. Uncertainty and sensitivity analysis for computer modeling applications. In: *Reliability Technology*, *The American Society of Mechanical Engineers 1992*, (ed T.A. Cruse), pp. 153-168. The American Society of Mechanical Engineers, New York.

McKay, M.D., Beckman, R.J., Conover, W.J., 1979. A comparison of three methods for selecting values of input variables in the analysis of output from a computer code. *Technometrics* 21, 239-245.

Pebesma, E.J., Heuvelink, G.B.M., 1999. Latin hypercube sampling of Gaussian random fields. *Technometrics* 41, 303-312.

Stein, M.L., 1987. Large sample properties of simulations using Latin hypercube sampling. *Technometrics*, 29, 143-151.

Wyss, G.D., Jorgensen, K.H., 1998, *A User's Guide to LHS: Sandia's Latin Hypercube Sampling Software*. Technical Report SAND98-0210. Sandia National Laboratories, Albuquerque, NM.

Zhang, Y., Pinder, G.F., 2004. Latin-Hypercube Sample-Selection Strategies for Correlated Random Hydraulic-Conductivity Fields. *Water Resources Research* 39(8) SBH 11
