# Supplementary material for: Impact of Influenza A Virus Infection on Growth and Metabolism of Suspension MDCK Cells Using a Dynamic Model
Source: Metabolites. 2022 Mar 12;12(3):239. doi: 10.3390/metabo12030239 (PMC8950586; doi:10.3390/metabo12030239)
Supplement: Supplementary file 1 [file metabolites-12-00239-s001.zip › Supplementary File S3.zip!/S3_file/Demo_MDCK/SBPOP_PACKAGE_Rev_1176_64bit/SBTOOLBOX2/_ACADEMIC/tools/THIRD_PARTY_SOFTWARE/xppaut/unix_mac/help/keys.html]

Keywords

Contents


---

|  |  |
| --- | --- |
| **Item** | **Command** |
|  |  |
| Poincare maps | **nUmerics Poincare** |
| Curve fitting | **nUmerics stocHastic fIt\_curve** |
| Fourier transform | **nUmerics stocHastic Fourier** |
| Histograms | **nUmerics stocHastic Histogram** |
| Autocorrelation | **nUmerics stocHastic Stat Correlation** |
| Liapunov expontents | **nUmerics stocHastic Liapunov** |
| Tables, changing properties of | **nUmerics looKup** |
| Averaging oscillators and adjoints | **nUmerics Adjoint** |
| Boundary value problem, tolerances for | **nUmerics bdryVal** |
| Fixed points, tolerances for | **nUmerics sIngpt\_ctl** |
| Nullcline, set mesh for | **nUmerics nCline\_ctl** |
| Integration parameters | **nUmerics Total** |
| Integration method | **nUmerics Method** |
| Delay parameters | **nUmerics dElay** |
| Color coding trajectories | **nUmerics Color\_code** |
| Printing | **Graphics Postscript** |
| Saving multiple plots | **Graphics Freeze Freeze** |
| Bifurcation curves, importing | **Graphics Freeze Bif.diag** |
| Color map | **Graphics Colormap** |
| Axes, changing values | **Graphics aXes** |
| Linetypes and curve colors | **Graphics Editcrv** |
| Storing data from single curve | **Graphics expOrt** |
| Quitting | **File Quit** |
| Bell, turning off | **File Bell** |
| Read/write current XPP information | **File Read\_set/Write\_set** |
| Editing right-hand sides, loading DLLs | **File Edit** |
| Transpose data | **File Transpose** |
| Fixed points | **SingPts Go** |
| Changing from 2D to three-D view | **Viewaxes** |
| "Space-time" plots | **Viewaxes Array** |
