# Supplementary material for: Impact of Influenza A Virus Infection on Growth and Metabolism of Suspension MDCK Cells Using a Dynamic Model
Source: Metabolites. 2022 Mar 12;12(3):239. doi: 10.3390/metabo12030239 (PMC8950586; doi:10.3390/metabo12030239)
Supplement: Supplementary file 1 [file metabolites-12-00239-s001.zip › Supplementary File S3.zip!/S3_file/Demo_MDCK/SBPOP_PACKAGE_Rev_1176_64bit/SBTOOLBOX2/_ACADEMIC/tools/THIRD_PARTY_SOFTWARE/xppaut/unix_mac/help/xpp3d.html]

XPP - 3D PARAMETERS

Back | Next | Contents


---

# 3d params

Brings up a dialog box allowing you to rotate the axes in three-dimensional plots and make animations. The items are:   

- **theta**, the angle about the  z-axis  to use this.- **phi**, the angle about the  y-axis - **Movie** set this to 1 if you want to create a little movie in which the box rotates about one of the two principle axes.- Choose which angle you want to rotate about: theta or phi- Choose the starting angle- Choose the increment- Choose the number of frames

If you select  **Movie**  then a series of frames will be drawn. You can use the  Kinescope  command to play these back or save them in an animated gif file.
