# Supplementary material for: Impact of Influenza A Virus Infection on Growth and Metabolism of Suspension MDCK Cells Using a Dynamic Model
Source: Metabolites. 2022 Mar 12;12(3):239. doi: 10.3390/metabo12030239 (PMC8950586; doi:10.3390/metabo12030239)
Supplement: Supplementary file 1 [file metabolites-12-00239-s001.zip › Supplementary File S3.zip!/S3_file/Demo_MDCK/SBPOP_PACKAGE_Rev_1176_64bit/SBTOOLBOX2/_ACADEMIC/tools/THIRD_PARTY_SOFTWARE/xppaut/unix_mac/help/xppanim.html]

ANIMATION

Contents


---

# Toons

To create an animation, just do the following:

- Run the numerical simulation for however long you need it or run it once the toon is loaded.

  - Using a text editor create a description of the animation using
    the little scripting language described  below .

    - Click on the (V)iew axes (T)oon menu item from the
      main XPP window to bring up the  animation window.

      - Click on the File item in the animation window that pops
        up and give it the name of your script file.

        - If the file is OK, click on Go item and the animation will
          begin.

          - Click on the little box in the upper right for animation on the fly -- the animation runs along with the simulation!

## The animation window

A resizeable window will pop up when you choose the Toon item
form the Viewaxes submenu. This has 10 buttons on it. They are

- **File** Click on this to load a new animation
  file. Hereafter, I will call these **ani** files. The usual
  extension will be **filename.ani**.

  - **Go** Click on here after you have loaded an ani file and it
    will run.

    - **Reset** This moves the animation back to the beginning

      - **Fast** speeds up the animation

        - **Slow** slows it down

          - **Pause** pauses the animation. **Go** restarts it from
            where it started. This is very sluggish and it takes a long time to
            respond to it. I hope to fix this someday.

            - **Skip** sets the numbers of frames to skip

              - **»»»»** Move forward one frame

                - **««««** Move back one frame

                  - **Mpeg** Store a series of ppm files for use with the
                    mpeg\_encode program. This takes a ton of disk space. Better yet, use
                    the **anigif** option which creates an animated gif file. 600
                    frames can be as little as 500K so it wont eat up disk space.

Some computer systems cannot produce the required amount of graphics
memory for a pixmap and if this happens, you cannot currently run the
animation. You will be told this and the animation window will never
appear.

---

## DASL: Dynamical Animation Scripting Language

In order to use the animation components of XPP, you must first
describe the animation that you want to do. This is done by creating a
script with a text editor that describes the animation. You describe
the coordinates and colors of a number of simple geometric
objects. The coordinates and colors of these objects can depend on the
values of the variables (both regular and fixed, but not auxiliary) at
a given time. The animation then runs through the output of the
numerical solution and draws the objects based on that output. I will
first list all the commands and then give some examples.

Basically,
there are two different types of objects: (i) transient and (ii)
permanent. Transient objects have coordinates that are recomputed at
every time as they are changing with the output of the
simulation. Permanent objects are computed once and are fixed through
the duration of the simulation. The objects themselves are simple
geometric figures and text that can be put together to form the
animation.

Each line in the script file consists of a command or object followed
by a list of coordinates that must be separated by semicolons. For
some objects, there are other descriptors which can be optional.
Since color is important in visualization, there are two ways a color
can be described. Either as a formula which is computed to yield a
number between 0 and 1 or as an actual color name started with the
dollar sign symbol, $. The ani file consists of a lines of
commands and objects which are loaded into XPP and played back on the
animation window. Here are the commands:

:   **dimension** xlo;ylo;xhi;yho
:   **speed** delay
:   **transient**
:   **permanent**
:   **line** x1;y1;x2;y2;color;thickness
:   **rline** x1;y1;color;thickness
:   **rect** x1;y1;x2;y2;color;thickness
:   **frect** x1;y1;x2;y2;color
:   **circ** x1;y1;rad;color;thickness
:   **fcirc** x1;y1;rad;color
:   **ellip** x1;y1;rx;ry;color;thickness
:   **fellip** x1;y1;rx;ry;color
:   **comet** x1;y1;type;n;color
:   **text** x1;y1;s
:   **vtext** x1;y1;s;z
:   **settext** size;font;color
:   **end**

All commands can be abbreviated to their first three letters and case
is ignored. At startup the dimension of the animation window in user
coordinates is (0,0) at the bottom left and (1,1) at the top
right. Thus the point (0.5,0.5) is the center no matter what the
actual size of the window on the screen. **Color** is described by either
a floating point number between 0 and 1 with 0 corresponding to red
and 1 to violet. When described as a floating point number, it can be
a formula that depends on the variables. In all the commands, the
color is optional *except* **settext.** The other way of
describing color is to use names which all start with the $
symbol. The names are:
**$WHITE, $RED, $REDORANGE, $ORANGE, $YELLOWORANGE,
$YELLOW, $YELLOWGREEN, $GREEN, $BLUEGREEN,
$BLUE,$PURPLE, $BLACK**.

The **transient** and **permanent** declarations tell the animator
whether the coordinates have to be evaluated at every time or if they
are fixed for all time. The default when the file is loaded is **transient.** Thus, these are just toggles between the two different
types of objects.

The number following the **speed** declaration must be a nonnegative
integer. It tells tha animator how many milliseconds to wait between
pictures.

The **dimension** command requires 4 numbers following it. They are
the coordinates of the lower left corner and the upper right. The
defaults are (0,0) and (1,1).

The **settext** command tells the animator what size and color to
make the next text output. The size must be an integer, **{
0,1,2,3,4 }**  with 0 the smallest and 4 the biggest. The font is
either **roman** or **symbol.** The color must be a named color
and not one that is evaluated.

The remaining ten commands all put something on the screen.

- **line x1;y1;x2;y2;color;thickness**  draws a line from **(x1,y1)**
  to **(x2,y2)** in user coordinates. These four numbers can be any
  expression that involves variables and fixed variables from your
  simulation. They are evaluated at each time step (unless the line is
  **permanent**) and this is scaled to be drawn in the window. The
  **color** is optional and can either be a named color or an
  expression that is to be evaluated. The **thickness** is also
  optional but if you want to include this, you must include the **color** as well. **thickness** is any nonnegative integer and will
  result in a thicker line.
- **rline x1;y1;color;thickness**  is similar to the **line**
  command, but a line is drawn from the endpoints of the last line drawn
  to **(xold+x1,yold+y1)** which becomes then new last point. All other options
  are the same. This is thus a ``relative'' line.
- **rect x1;y1;x2;y2;color;thickness**  draws a rectangle with
  lower corner **(x1,y1)** to upper corner **(x2,y2)** with optional
  color and thickness.
- **frect x1;y1;x2;y2;color**  draws a filled rectangle with
  lower corner **(x1,y1)** to upper corner **(x2,y2)** with optional
  color.
- **circ x1;y1;rad;color;thick**  draws a circle with radius **rad**
  centered at **(x1,y1)** with optional
  color and thickness.
- **fcirc x1;y1;rad;color**  draws a filled circle with radius **rad**
  centered at **(x1,y1)** with optional color.
- **ellip x1;y1;rx;ry;color**  draws an ellipse with radii **rx,ry**
  centered at **(x1,y1)** with optional
  color and thickness.
- **fellip x1;y1;rx;ry;color**  draws a filled ellipse with
  radii **rx,ry** centered at **(x1,y1)** with optional
  color.
- **comet x1;y1;type;n;color** keeps a history of the last n points drawn and renders them in the optional color. If type is non-negative, then the last n points are drawn as a line with
  thickness in pixels of the magnitude of type. If type is
  negative, filled circles are drawn with a radius of -thick in
  pixels.
- **text x1;y1;s** draws a string **s** at position **(x1,y1)** with the current color and text properties. Only the
  coordinates can depend on the current values.
- **vtext x1;y1;s;z** draws a string **s** followed by
  the floating point value **z** at position **(x1,y1)** with the current color and text properties. Thus, you can
  print out the current time or value of a variable at any given time.

*REMARK.* As with lines in ODE files, it is possible to create
arrays of commands using the cobination of the **[i1..i2]** 
construction. Some examples are shown below.
