# Supplementary material for: Impact of Influenza A Virus Infection on Growth and Metabolism of Suspension MDCK Cells Using a Dynamic Model
Source: Metabolites. 2022 Mar 12;12(3):239. doi: 10.3390/metabo12030239 (PMC8950586; doi:10.3390/metabo12030239)
Supplement: Supplementary file 1 [file metabolites-12-00239-s001.zip › Supplementary File S3.zip!/S3_file/Demo_MDCK/SBPOP_PACKAGE_Rev_1176_64bit/SBTOOLBOX2/_ACADEMIC/tools/THIRD_PARTY_SOFTWARE/xppaut/unix_mac/help/xpparray.html]

ARRAY PLOTS

Contents


---

# Array plots

 **(A)rray plot**  introduces a new window that lets the user plot many
variables at once as a function of time with color coded values. The
user can plot for example an array of voltages,  V1, V2,
..., VN  across the horizontal as time varies in the vertical
dimension. For example, suppose you have discretized some PDE and want
to see the evolution in space and time of the variables. Then use
this plotting option. A dialog box will appear asking for

- the first column name
  of the  **array** ,- the number of columns,- the first row,- the numberof rows,- the number of rows to skip,- the max and min of the plotted variable- the column skip.

For example, if the
discretized PDE variables are {\tt u0,u1, ... ,u50}, then type in {\tt
u0} for the first element and {\tt 51} for the number of columns.
If you want rows 200
through 800 only every 4th time unit, you would put 200 for the first
row, 201 as the number of rows, and 4 as the row skip value.
You can also set the column skip as well. This is useful if you have
defined a series of variables with the array blocks. If the array
block is for a two-component system, then set the column skip to
2, to plot every other variable.

The  **Print** 
button asks you for a file name and top and bottom labels and a
render style. The render styles are:

- **-1:**  Grey scale- **0:**  Blue-red- **1:**  Red-Yellow-Green-Blue-Violet- **2:**  Like  **1** but periodic

The  **Style**  button does nothing yet. The  **Edit**  button lets
you change ranges and arrays to plot. The  **Redraw**  button is obvious.

## Shortcut

In the  **Initial conditions window**  each variable has a little
box next to it. If you click the box next to the first element in
the array and the box next to the last element in the array and then
click the  **Arry**  button at the bottom, you will get a nice
scaled array plot!
