# Supplementary material for: Impact of Influenza A Virus Infection on Growth and Metabolism of Suspension MDCK Cells Using a Dynamic Model
Source: Metabolites. 2022 Mar 12;12(3):239. doi: 10.3390/metabo12030239 (PMC8950586; doi:10.3390/metabo12030239)
Supplement: Supplementary file 1 [file metabolites-12-00239-s001.zip › Supplementary File S3.zip!/S3_file/Demo_MDCK/SBPOP_PACKAGE_Rev_1176_64bit/SBTOOLBOX2/_ACADEMIC/tools/THIRD_PARTY_SOFTWARE/xppaut/unix_mac/help/xppauto.html]

 XPP AUTO 

Main Help


# Bifurcation Calculations with AUTO

## Running AUTO

### Preparation Choosing the "hot" parameters Choosing the plotting axes Setting up the numerical parameters User defined points Running and continuing from a point Specially labeled points Traversing the diagram and selecting points Stopping a calculation Saving, printing, resetting diagrams

---

## Preparation

Before you can use AUTO, you must prepare your system for it. You must
start your bifurcation analysis from either a fixed point of your
model, a periodic orbit, or a boundary value.
AUTO seems to work best when you start
from a steady state, but I have had success starting at periodic
orbits.

- If you want to start at a steady state, find one and
  integrate so that the system is at rest. Use the   **Init.conds. Last**   repeatedly.- If you want to start at a
    periodic orbit, find one by integration and make sure you have
    converged to it. The set the  total integration 
    time is the ``period'' of your orbit. This is what the AUTO interface
    uses as an approximate starting period.- If you want to use AUTO to solve a boundary value problem, you
      must do several things.
      1. First rescale everything so that the total
         length of the interval is 1.- If the system is non-autonomous, define a new variable to make it
           autonomous.- The number of ODEs must be the same as the number of boundary
             conditions.- Compute a solution using XPP's  boundary
               value solver  or guessing a solution. (AUTO is pretty forgiving -- moreso than the XPP BVP solver.)
      - Setting up for homoclinics is similar to BVPs. Make sure that you
        include the  **hom\_bcs**  boundary conditions.

## Choosing parameters

The first thing you should do is tell AUTO which parameters you might
use in the bifurcation analysis. Up to 5 are allowed. Click on
``Parameter'' and a list of 5 parameters will appear. Type in the
names of the parameters you want to use. The default is the first 5
or fewer parameters
in your file. If you have fewer than 5 parameters, only the
available ones will appear.  *You can have as many parameters in
your ODE file as you want. However, the AUTO interface allows only 5
 **hot**  parameters.* 

## Diagram axes

Use this to set up the plotting of the diagram as well as to tell AUTO
which parameters should be varied. This is also where you set up for
two-parameter continuation. There are 8 choices

- **(H)i**  This plots the maximum of the chosen variable.- **(N)orm**  This plots the  *L\_2*  norm of the solution.- **h(I)-lo**  This plots both the max and min of the chosen variable
      (convenient for periodic orbits.)- **(P)eriod**  Plot the period versus a parameter- **(T)wo par**  Plot the second parameter versus the primary
          parameter for two-parameter continuations.- **(Z)oom**  Use the mouse to zoom in on a region.- **last (1) par**  Use the plot parameters from the last 1-parameter
              plot.- **last (2) par**  Use plot parameters from last 2-parameter
                plot.- **(F)requency**  Plot frequency versus a parameter- **(A)verage**  Plot the average over a period of a quantity

After clicking, a new window pops up with the following items:

- **Y-axis**  This is the variable for the y-axis of the plot. For
  two-parameter and period plots, its contents is ignored.- **Main Parm**  This is the principal bifurcation parameter. It must
    be one of those you specified in the parameter window. The default is
    the first parameter in the parameter list.- **2nd Parm**  This is the other parameter for two-parameter
      continuations.- **Xmin ... Ymax**  The plotting dimensions of the diagram.

Once you press (OK) the axes will be redrawn and labeled.

## Numerical parameters

You will often have to change these. In particular, you set the range
of the parameters as well as the direction of bifurcation in this
dialog.

- **Ntst**  This is the number of mesh intervals for discretization
  of periodic orbits.
  If you are getting bad results or not converging, it helps
  to increase this. For following period doubling bifurcations, this is
  automatically doubled so you should reset it later.- **Nmax**  The maximum number of steps taken along any branch. If
    you max out, make this bigger.- **Npr**  Give complete info every Npr steps. Set this to a
      big number if you want to speed things along.- **Ds**  This is the initial step size for the bifurcation
        calculation.  *The sign of Ds tells AUTO the direction to
        change the parameter.*  Since stepsize is adaptive, (Ds) is just a
        ``suggestion.''- **Dsmin**  The minimum stepsize (positive).- **Dsmax**  The maximum step size. If this is too big, AUTO will
            sometimes miss important points so if it seems to miss a stability
            transition, or if the diagram is jagged, decrease this.- **Par Min**  This is the left-hand limit of the diagram for the
              principle parameter. The calculation will stop if the parameter is
              less than this.- **Par Max**  This is the right-hand limit of the diagram for the
                principle parameter. The calculation will stop if the parameter is
                greater than this.- **Norm Min**  The lower bound for the *L_2_* norm of the solution. If it
                  is less than this the calculation will stop.- **Norm Max**  The upper bound for the *L_2_* norm of the solution. If it
                    is greater than this the calculation will stop.- **Ncol**  number of collocation points used. I never have changed this in 10 years of running AUTO.- **EPSU,EPSS,EPSL**  some sort of tolerances for AUTO. Keep 'em positive but make 'em smaller for better accuracy. 1e-7 seems to be a popular choice!

## User functions

Suppose you want to get plots at specific values of parameters or at
fixed periods of a limit cycle. Then you can click on ``User''
which produces a menu 0-9 asking you how many points you want to keep.
Click on 0 for none or another number less than 10. A new window will appear
with slots for 9 items. You can type in anything of the form:
parameter=value
or
T=value
AUTO will mark and save complete information for any point that
satisfies either of these criteria. The second is used to indicate
that you want to keep a point with a particular period, e.g.,
T=25 will save the any periodic orbit with period 25.

## Running

Click on ``Run'' to run the bifurcation. Depending on the situation,
a number of menus can come up. For initial exploration, there are four
choices:

- starting at a new steady state- starting at a periodic orbit- starting at a boundary-value solution,- starting at a homoclinic/heteroclinic orbit.

You must be sure that you are starting from a good initial solution or the continuation will fail. If successful,
a nice diagram will show up and a bunch of points will move
around in the stability circle. These indicate stability: for fixed
points, they represent exponentials of the eigenvalues; for periodics,
the Floquet multipliers. Thus those in
the circle are stable and those out of the circle are unstable.
Bifurcations occur on the circle. The outer eigenvalues are ``clipped'' so
that they will always lie in the square, thus you can keep count of
them.

### Special Points

The diagram,itself, has two different lines and two different circles.
Stable fixed points are thick lines, stable periodics are solid
circles, unstable fixed points are thin lines, and unstable periodics
are open circles. Additionally, there are crosses occasionally
dispersed with numbers associated with them. These represent
``special'' points that AUTO wants to keep. There are several of
them:

- **EP**  Endpoint of a branch- **LP**  Limit point or turning point of a branch- **TR**  Torus bifurcation from a periodic- **PD**  Period doubling bifurcation- **UZ**  User defined function- **MX**  Failure to converge- **BP**  Bifurcation or branch point- **HB** Hopf bifurcation point- Output every  *N^th^*  point.

## Grabbing

You can use these special points to continue calculations with AUTO.
The ``Grab'' item lets you peruse the diagram at a leisurely pace and
to grab special points or regular points for importing into XPP or
continuing a bifurcation calculation. Click on ``Grab'' and info
appears in the info window and a cross appears on the diagram. Use
the left and right arrow keys to cruise through the diagram. The right
key goes forward and the left backward. At the bottom, information
about the branch, the point number, the type of point, the AUTO label,
the parameters, and the period are given. The points marked by crosses
have lables and types associated with them. The type is one of the
above. The label corresponds to the number on the diagram. If point
is positive, it is an unstable solution and if it is negative it is
stable. As you traverse the diagram, stability is shown in the circle.

You can traverse the diagram very quickly by tapping the (Tab) key
which takes you the special points only. Type (Esc) to exit with
no action or type (Enter) to grab the point. If it is a regular
point (i.e., not special) then the parameters and the variables will
be set to the values for that point within XPP. You can then
integrate the equations or look at nullclines, etc. If you grab a
special point, then you can use this as a restart point for more AUTO
calculations, such as fixed period, two-parameter studies, and
continuations. Then, you can run AUTO again. Bifurcation diagrams are
cumulative unless you reset them in the ``File'' menu. That is, new
stuff is continually appended to the old. The only limit is machine
memory.

If you grab a special point and click on ``Run'' several possibilities
arise depending on the point:

- **Regular Point**  Reset the diagram and begin anew. You will be
  asked first if you want to reset the diagram first.- **Hopf Point** 
    - **Periodic**  Compute the branch of periodics emanating from
      the Hopf point- **Extend**  Continue the branch of steady states through
        this point.- **New Point**  Restart whole calculation using this as a
          starting point- **Two Param**  Compute a two parameter diagram of Hopf
            points.
    - **Period doubling** 
      - **Doubling**  Compute the branch of period 2 solutions.- **Two-param**  Compute two-parameter curve of period
          doubling points.
      - **Limit point**  Compute two parameter family of limit points (fixed
        points or periodic.)- **Periodic point**  The point is periodic so
          - **Extend**  Extend the branch- **Fixed Period**  Two parameter branch of fixed period
              points.
          - **Branch point of periodic**  Branch points of steady states
            are automatically followed by AUTO. If you click on a branch point
            for a periodic, the program traces out the new branch. Changing the
            sign of  **Ds**  will trace a different branch.- **Torus point**  Compute two-parameter family of torus
              bifurcations or extend the branch or compute two-parameter
              fixed period.

*Before running, after a point is grabbed, be sure to set up the
correct axes and ranges for the parameters.* 

## Aborting

Any calculation can be gracefully stopped by clicking on the ``Abort''
key. This produces a new end point from which you can continue. Note
that if there are many branches, you may have to press "Abort"
several times.

**Clear**  clears the diagram and  **reDraw** redraws it.

## Saving diagrams

**File**  allows you to do several things:

- **Import orbit**  If the grabbed point is a special one and is a
  periodic orbit or BVP solution,
  this loads the orbit into XPP for plotting. This is
  useful for unstable orbits that can't be computed by integrating
  initial data.- **Save diagram**  Writes a file for the complete diagram which you
    can use later.- **Load Diagram**  Loads a previously saved one.- **Postscript**  This makes a hard copy of the bifurcation diagram- **Reset diagram**  This clears the whole thing.- **Clear Grab**  This clears the grab point.- **Write Points**  This makes an ascii file of the  **(x,y)**  coordinates of the diagram. Use this to make nicer figures with
              by  importing  the diagram into XPP- **All info**  writes all the fixed points, eigenvalues, etc for
                each point on the diagram.
