# Supplementary material for: Impact of Influenza A Virus Infection on Growth and Metabolism of Suspension MDCK Cells Using a Dynamic Model
Source: Metabolites. 2022 Mar 12;12(3):239. doi: 10.3390/metabo12030239 (PMC8950586; doi:10.3390/metabo12030239)
Supplement: Supplementary file 1 [file metabolites-12-00239-s001.zip › Supplementary File S3.zip!/S3_file/Demo_MDCK/SBPOP_PACKAGE_Rev_1176_64bit/SBTOOLBOX2/_ACADEMIC/tools/THIRD_PARTY_SOFTWARE/xppaut/unix_mac/help/xppbdry.html]

XPP - BVP SOLVER

Back | Contents


---

# Boundry Value Problem Solver

This invokes the boundary value solver which uses numerical shooting. There are 4 choices.

- **(S)how** This shows the successive results of the shooting, erases the screen at the end and redraws the last solution. XPP uses the currently selected numerical integation method, the current starting point,  **T0**  as the left end time and  **T0+TEND**  as the right end. These are set in the  Numerics  menu. Thus, if the interval of interest is (2.5,6)then set  **T0=2.5**  and  **TEND=3.5**  in the numerics menu.

  - **(N)o show**   
     This is as above but will not show intermediate solutions.

    - **(R)ange**   
       This allows you to range over a parameter keeping starting or ending values of each of the variables. A dialog box appears asking you for   
      - the parameter to vary,- the starting value,- the end value,- the number of steps,- You will be asked if you want to cycle color which means that the results of each successful solution to the BVP will appear in different colors,- **side** tells the program whether to save the initial **(0)** or final**(1)** values of the solution.
      As the program progresses, you will see the current parameter in the info window under the main screen. You can abort the current step by pressing (Esc) and the whole process by pressing (/).

      - **(P)eriodic**   
         Periodic boundary conditions can be solved thru the usual methods, but one then must write an addition equation for the frequency parameter. This option eliminates that need so that a 2-D
        autonomous system need not be suspended into a 3D one. A dialog box appears and you will be asked for
        - the name of the adjustable parameter for frequency,- the section variable and section. This is an additional condition that must be satisfied, namely, *x(0)=x\_0*  where *x* is the section variable and  *x\_0*  is the section.- Choose  **yes** if you want
              the progress shown.
        - **(H)omoclinic**

          This lets you tell XPP which variables carry the values of the fixed
          points as well as the dimensions of the stable and unstable manifolds. Left and right fixed points are requested (for homoclinics they are the same.) You also must tell the number of unstable eigenvalues on the left and stable on the right.
