# Supplementary material for: Impact of Influenza A Virus Infection on Growth and Metabolism of Suspension MDCK Cells Using a Dynamic Model
Source: Metabolites. 2022 Mar 12;12(3):239. doi: 10.3390/metabo12030239 (PMC8950586; doi:10.3390/metabo12030239)
Supplement: Supplementary file 1 [file metabolites-12-00239-s001.zip › Supplementary File S3.zip!/S3_file/Demo_MDCK/SBPOP_PACKAGE_Rev_1176_64bit/SBTOOLBOX2/_ACADEMIC/tools/THIRD_PARTY_SOFTWARE/xppaut/unix_mac/help/xppbrowse.html]

 XPP BROWSER 


# Examining the data

## Click open the Data Browser in order to use it. Typing (Esc) makes the main XPP window the "hot" window.

### The DATA BROWSER lets you examine and minipulate the numbers generated by your simulation. It also lets you import files of numbers for plotting . It acts like a *very primitive* spreadsheet.

Once
you have computed a trajectory to an equation, you can use the Data
Browser (here after, DB) to look at the data. There are some known
``bugs.'' You must sometimes grab it with the mouse and shake it to
get the data to show up (no kidding!) Also, sometimes it will not
resize properly; in this case, you must continue the integration to
the current starting point (in other words, do nothing but press (C) and
then (Enter).) To activate the DB, bring the window to the top and
put the pointer inside the box.

Across the top is a menu of commands
and then there follows a list of titles for the variables, time and
the auxiliary functions. Using the arrow keys, the page keys, home and
end keye or
clicking on the appropriate commands allows you to scroll through the
data. By resizing the DB you can get more or less data. Clicking on
(Left) shifts the data window to the left and (Right) moves it to the
right. The time column always stays fixed. If you do parametric or
range calculations, the range variable is kept in the time column.
(Home) takes you to the top of the data and (End) to the last row. The
remaining commands will be described separately. The keyboard
shortcuts to invoke them are in parentheses.

- **(F)ind**  This pops up a window and asks for a variable and a
  value. It then looks through the data until it comes to the closest
  value to the specified that the variable takes and brings it to
  the top of the browser. It only moves down
  the file so that you can find successive values by starting at the
  top. ( *Hint: To find the largest or smallest value in a column,
  just set the value really high or really low; XPP will find the
  closest value.*)- **(G)et** This makes the top row of data the initial coonditions
    for a new integration.- **Re(p)lace** This pops up a window asking you for a column to
      replace. Then it prompts you for a formula. Suppose you want to
      replace AUX1 with x+y-t where x,y are two variables.
      Then type AUX1 when asked which column to replace and then type in the
      formula and (OK). Each row in the requested column
      will be replaced by the values of your formula. Any valid XPP
      function or user-defined function can be used. Two special symbols can also
      be used when applied to single variables:

      **Numerical integration and differentiation.** 

      - **@VARIABLE** replaces the column with the numerical derivative of
        the variable. You cannot use this within a formula, but once the
        column is replaced, it can be treated as any other column.- **\&VARIABLE** replaces the column with the numerical integral.
          Note that successive applications of the derivative and then the
          integral will result in the original plus a constant.
      - **(U)nreplace** This undoes the most recent replacement.- **Fir(s)t** This marks the top row in the DB (nothing is shown)as
          the start or first row for saving or restoring.- **Last (e)** This marks the top row as the last or end row for
            saving or restoring. Using First and Last lets you mark and plot
            selected parts of your data.- **(T)able** This lets you save data in a tabulated format that can
              then be used by XPP as a function or inputs. You must use the (First)
              and (Last) keys to mark the desired data you want to save.
              You are then prompted for the column name, the minimum and maximum you
              want your independent variable to range and the file name. The result
              is a table file of the format used by XPP.- **(R)estore** replots the data marked by First and Last. The
                default is the entire data set

                **Reading and writing numbers to files**  

                - **(W)rite** prompts you for a filename and writes the marked data
                  to a file. The files are of the form:

                  t0 x1(t0) ... xn(t0)

                  t1 x1(t0) ... xn(t0)

                  .

                  .

                  .

                  tf x1(tf) ... xn(tf)

                  and reflect the current contents of the DB. They are ASCII
                  readable. Thus you can use  *superior*  graphics programs to plot
                  the results of your simulations.- **(L)oad** Will load in as much of a similarly formated data file
                    as possible for graphing.

                    **Adding and deleting columns of data**  

                    - **(A)ddcol** This allows you to add an additional column to the
                      data browser. You are prompted for the name you want to give the
                      column and for the formula. It is thus, like an auxiliary variable
                      with a name. Thereafter, it will be computed along with any other
                      quantities that you have defined. It is as though you had included
                      another auxilliary variable in your original file.- **(D)elcol** This is buggy so it is disabled.
                        If the purpose of deleting
                        them is to change the formula, use the right-hand-side editor,
                        (File-Edit) instead.
                        <\ul>
