# Supplementary material for: Impact of Influenza A Virus Infection on Growth and Metabolism of Suspension MDCK Cells Using a Dynamic Model
Source: Metabolites. 2022 Mar 12;12(3):239. doi: 10.3390/metabo12030239 (PMC8950586; doi:10.3390/metabo12030239)
Supplement: Supplementary file 1 [file metabolites-12-00239-s001.zip › Supplementary File S3.zip!/S3_file/Demo_MDCK/SBPOP_PACKAGE_Rev_1176_64bit/SBTOOLBOX2/_ACADEMIC/tools/THIRD_PARTY_SOFTWARE/xppaut/unix_mac/help/xppdfield.html]

XPP - DIRECTION FIELD/FLOW

Back | Next | Contents


---

# **Direction Field/Flow**

This option is best used for two-dimensional systems however, it can be applied to any system. The current graphics view must be a two-d plot in which both variables are different and neithet is the time variable, T. There are five items.

- **(D)irection fields**    
   Choosing the direction field option will prompt you for a grid size. The two dimensional plane is broken into a grid of the size specified and lines are drwan at each point specifying the direction of
  the flow at that point. If the system is more than two-dimensional, the other variables will be held at the values in the initial conditions window.

  - **(F)low**    
     Choosing the flow, you will be prompted for a grid size
    and trajectories started at each point on the grid will be integrated according to the numerical parameters.

    Any given trajectory can be aborted by pressing
    **Esc** and the whole process stopped by pressing **/**.

    The remaining
    variables if in more than two-dimensions are initialized with the values in the IC window.

    - **(N)o Dir Field**    
       turns off redrawing of direction fields when you click on (Redraw). Erasing the screen automatically turns this off

      - **(C)olorize**    
         This draws a grid of filled rectangles on the screen whose color is coded by the velocity or some other quantity. (See the Numerics|Color code menu item).

        - **(S)caled Dir. Fld**    
           is the same as (D)irection field but the
          lengths are all scaled to 1 so only directional information is given.
