# Supplementary material for: Impact of Influenza A Virus Infection on Growth and Metabolism of Suspension MDCK Cells Using a Dynamic Model
Source: Metabolites. 2022 Mar 12;12(3):239. doi: 10.3390/metabo12030239 (PMC8950586; doi:10.3390/metabo12030239)
Supplement: Supplementary file 1 [file metabolites-12-00239-s001.zip › Supplementary File S3.zip!/S3_file/Demo_MDCK/SBPOP_PACKAGE_Rev_1176_64bit/SBTOOLBOX2/_ACADEMIC/tools/THIRD_PARTY_SOFTWARE/xppaut/unix_mac/help/xppdll.html]

XPP - DLL

Contents


---

# Using DLLs

For compilers which allow dynamic linking, it is possible to use
external C programs to speed up the right-hand sides etc. This also
lets you create complicated right-hand sides that would be difficult
to do with the XPP parser. Here are the steps you need to do this.

1. Write some C-code for the right-hand sides. Here is the form of
   the code:

   ```
   #include 
   f1(double *in,double *out,int nin,int nout,double *var,double *con)
   {


   }
   f2(double *in,double *out,int nin,int nout,double *var,double *con)
   {

   }

   ...
   ```

   You can define as many of these functions as you want within one
   file. The parameters  **in**  are passed by XPP to the
   function. The parameters  **out**  are what the function computes
   and passes back to XPP. The integers  **nin,nout**  are junt the
   number of such passed parameters. I never use them.
   The array  **con**  contains all your parameters in the order you
   defined them in the ODE file
   starting with  **con[2]**  and the array  **var**  contains the time
   variable, followed by the state variables, and then the fixed
   variables. Thus, you could directly communicate with all the
   variables or parameters.- Next compile to code into a shared library. Here is what I type

     ```
     gcc -shared -fpic -o pp.so pp.c
     ```

     where  **pp.c**  is what I called the C file.- Next write an ODE file that can take advantage of this using
       the  `export {} {}`  statement to send out and read in what
       is needed.- Run XPP and use the  **File Edit Load-DLL**  command to load
         in the library and tell XPP which function to call. For the latter,
         typing  **f1**  would implement the first bit of code while typing
         **f2** uses the second. You can change both the function and the
         library while XPP is running.- If all goes well, then XPP will run with the hard-coded RHS.

---

### Example 1

I will implement a predator-prey model:

*x' = x((x+c)(1-x)-y)

y' = y(x-a)* 
I will pass in the value of the parameters and the variables and get
back the values of the right-hand sides.

Here is the ODE file:

```
# pp.ode with dlls
#
# xp,yp are the right-hand sides
x'=xp
y'=yp
# a,b are parameters
par a=.4,c=0
init x=.1,y=.2
# dummies to allocate storage
xp=0
yp=0
# here is the main communication
export {a,c,x,y} {xp,yp}
@ total=50
done
```

The parameters and current values of the variables are passed in via
the first  **{}**  in the export statement. The second  **{}** 
passes back values for the fixed variables,  **xp,yp**  which are
the right-hand sides. You have to define these in XPP so I usually set
them to zero.

Next I write the C code for the right-hand sides:

```
/* pp.c for DLL
 */
#include 

/* some defines for readability */

#define a in[0]
#define c in[1]
#define x in[2]
#define y in[3]

#define xp out[0]
#define yp out[1]

pp(double * in,double *out, int nin,int nout, double *var,double *con)
{
  xp=x*((x+c)*(1-x)-y);
  yp=y*(x-a);
}
```

I have added a few defines to make it easier to read and write.

I compile this as

```
gcc -shared -fpic -o pp.so pp.c
```

This should give me a file called  **pp.so**  which is a shared
labrary.

I run the ODE file with XPP. I click on  **File Edit Load-DLL** 
and choose the file  **pp.so**  and  **pp**  for the function
name (since that is what I called it in the C file).

Then let it rip to see the solution.

---

### Example 2 - an array

This next example reads the variables and passes the right-hand side
directly to the array  **var**  since the  **export**  directive
cannot pass arrays in any simple manner. The equation is the
discretization of the bistable front:

*u_0_' = f(u_0_)+d(u_1_ - u_0_)

u_80_' = f(u_80_)+d(u_79_ - u_80_)

u_j_' = f(u_j_)+d(u_j-1_ - 2u_j_
+u_j+1_ )*  for  *j=1,...,79* 

Here is the ODE file

```
# wave front in bistable RD model
u[0..80]'=up[j]
up[0..80]=0
par a=.1,d=.25,n=80
init u[0..4]=1
export {a,d}
@ total=400,nout=2
done
```

Note that only the parameters are exported and nothing is sent back
throught the  **export**  statement. I have set the total to 400
and plot every other point.

Next, I write the C code:

```
#include 

double f(double x,double a)
{
  return  x*(1-x)*(x-a);
}

front(double *in,double *out, int nin, int nout, double *var, double *con)
{
  int i;
  double a=in[0];
  double d=in[1];
  double *x=var+1;
  double *xp=x+81;
  xp[0]=f(x[0],a)+d*(x[1]-x[0]);
  xp[80]=f(x[80],a)+d*(x[79]-x[80]);
  for(i=1;i<80;i++)
    xp[i]=f(x[i],a)+d*(x[i+1]-2*x[i]+x[i-1]);
}
```

Note how I pass the inputs to the pointer **x** and the outputs to
the pointer **xp** which respectively point to the elements
**var[1]** and **var[82]**

Compile this

```
gcc -shared -fpic -o front.so front.c
```

Now run  **front.ode**, click on  **File Edit Load DLL** 
and load  **front.so**  choosing the function  **front**. Let it
rip.

I compared this to a normal ODE file

```
# wave front in bistable RD model
f(u)=u*(1-u)*(u-a)
u[0..80]'=up[j]
up0=f(u0)+d*(u1-u0)
up[1..79]=f(u[j])+d*(u[j-1]+u[j+1]-2*u[j])
up80=f(u80)+d*(u79-u80)
par a=.1,d=.25,n=81
init u[0..4]=1
@ total=400,nout=2
done
```

and get about a two-fold increase in speed.
