# Supplementary material for: Impact of Influenza A Virus Infection on Growth and Metabolism of Suspension MDCK Cells Using a Dynamic Model
Source: Metabolites. 2022 Mar 12;12(3):239. doi: 10.3390/metabo12030239 (PMC8950586; doi:10.3390/metabo12030239)
Supplement: Supplementary file 1 [file metabolites-12-00239-s001.zip › Supplementary File S3.zip!/S3_file/Demo_MDCK/SBPOP_PACKAGE_Rev_1176_64bit/SBTOOLBOX2/_ACADEMIC/tools/THIRD_PARTY_SOFTWARE/xppaut/unix_mac/help/xppexample.html]

XPP - EXAMPLES

Contents


---


# Examples

#### Systems of ODEs

#### Higher order ODEs

#### Maps and difference equations

#### Delay equations

#### Differential-algebraic equations

#### Volterra equations

#### Markov models

#### Delta functions

#### Boundary value problems

#### Discretized PDEs

#### Spatial networks

---

- ### Differential equation

  Here is the Wilson-Cowan equation:

  *u' = -u+f(a_ee_u-a_ie_v-z_e_ + I_e_(t) )
    
  v' = (-v+f(a_ei_u-a_ii_v-z_i_ + I_i_(t) ))/tau*  
  and here is the corresponding ODE file:

  ```
  # wilson-cowan
  f(u)=1/(1+exp(-u))
  par aee=10,aie=8,aei=12,aii=3,ze=.2,zi=4
  par tau=1
  i_e(t)=ie0+ie1*sin(w*t)
  i_i(t)=ii0+ii1*sin(w*t)
  par ie0=0,ie1=0,w=.25
  par ii0=0,ii1=0
  init u=.1,v=.05
  u'=-u+f(aee*u-aie*v-ze+i_e(t))
  v'=(-v+f(aei*u-aii*v-zi+i_i(t)))/tau
  @ total=40
  @ xp=u,yp=v,xlo=-.1,xhi=1,ylo=-.1,yhi=1
  done
  ```

  I have added definitions for the inputs and the function **f** as
  well as default initial data and parameters. I also set it up so that
  the initial window is the phaseplane.

  ---

  - ### Higher order differential equations

    XPP cannot solve higher order equations. You must first write them as
    a system of first order equations. For example, the third order
    equation:

    *x''' + a x'' + b x' + c x = x^2^*

    should be rewritten as

    *x' = y   
    y' = z   
    z' = x^2^ - a z -b y -c x*

    with the corresponding ODE file

    ```
    # third.ode
    x'=y
    y'=z
    z'=x^2-a*z-b*y-c*x
    par a=1,b=2,c=3.7
    init x=1,y=0,z=0
    aux en=x^2+y^2+z^2
    @ total=200
    @ xp=x,yp=y,xlo=-2,xhi=5,ylo=-4,yhi=5
    done
    ```

    I have set the plotting parameters and integration time. I have also
    added another quantity  **en**  which is stored and can be plotted
    as well.

    ---

    - ### Difference equation or maps

      The example is the delayed logistic map

      *x_n+1_ = a x_n-1_(1-x_n_)* 

      This is a second order map and XPP can only solve first order
      equations. We write it as a pair of first order equations:

      *x_n+1_ = y_n_   
      y_n+1_ = a y_n_(1-x_n_)* 

      and get the corresponding ODE file:

      ```
      # delayed logistic map
      x'=y
      y'=a*y*(1-x)
      par a=2.2
      init x=.2,y=.2
      # set method to discrete
      @ meth=discrete
      @ total=100
      @ ylo=-.1,yhi=1,xhi=100
      done
      ```

      Here the most important thing is that I tell XPP that this is a
      discrete dynamical system.

      ---

      - ### Delay-differential equation

        XPP can solve delay equations with one or more delays. Here is a delay
        equation due to Mike Mackey and Leon Glass:

        *x'(t) = -g x(t) + b f(x(t-d))   
        f(x) = x/(1+x^n^)*  
        Here is the ODE file:

        ```
        # mackey-glass
        f(x)=x/(1+x^n)
        x'=-g*x+b*f(delay(x,d))
        init x=.2
        par g=.1,b=.2,n=10,d=6
        @ delay=20
        @ total=200
        @ xhi=200,yhi=2
        done
        ```

        I have told XPP that the maximal delay to expect is 20.

        Delay equations with delay  **d**  require initial data on an
        interval  **-d < t < 0.**  You can set these in the ODE file
        by adding the line:

        ```
        x(0)=exp(t)*0.2
        ```

         which sets  **x(t)=0.2e^t^**  for  **-d
        < t < 0.** 

        **NOTE**  this does  *not*   set the initial condition for  **x** at **t=0.** You can do this by adding one more line after the delayed condition:

        ```
        x(0)=exp(t)*0.2
        init x=.2
        ```

        #### Setting delay initial data within XPP

        There is a window called  **Delay ICs**  which shows up in XPP and
        you can use this to type in delay initial data.  Before integrating delay equations always click on the
         OK  button in the  **Delay ICs**  window! Otherwise, the delay initial data will not take effect.

        ---

        - ### Differential-algebraic equations

          Differential algebraic equations of the form:

          *x' = F(x,y)   
          0 = G(x,y)*

          can be solved in XPP. The DAE

          *x' = z  
          z' = y   
          0 = x+z+y+y^3*

          has an ODE file:

          ```
          # dae.ode
          x'=z
          z'=y
          0=x+z+y+y^3
          solve y=-1
          init x=2
          aux ys=y
          @ yhi=2.5
          done
          ```

          You have to tell it which variables to solve for. In this case, we
          solve for  **y**  as a function of  **x,z** . You can give XPP a
          guess about what the value of  **y**  is at the initial conditions
          for  **x,z.** I have added an additional quantity  **ys**  which
          is the solution to the algebraic condition.

          ---

          - ### Volterra integral equations

            XPP solves both continuous
            and singular integral equations. Here is a regular equation:

            *u(t)=sin(t) + 0.5 cos(t)-0.5 e^-t^(t + 1) + int[u(t') (t-t')
            e^t'-t^,t'=0..t]*

            and here is the ODE file

            ```
            # volterra example 1
            u(t)=sin(t)+.5*cos(t)-.5*t*exp(-t)-.5*exp(-t)+int{(t-t')*exp(t'-t)*u} 
            aux utrue=sin(t)
            done
            ```

            I have included the true solution as well. This is actually a
            convolution equation so that you and improve the speed by exploiting
            that:

            ```
            #  volt2.ode - uses convolution to speed up soln
            u(t)=sin(t)+.5*cos(t)-.5*t*exp(-t)-.5*exp(-t)+int{t*exp(-t)#u}
            aux utrue=sin(t)
            done
            ```

            Integro-differential equations are also possible:

            *u'(t)=-2u+e^-2t^ + int[u^2(t')e^t'-t^,t'=0..t]*

            with  *u(0)=1*  has a solution  *u(t)=e^-t^* as can
            be confirmed with the ODE file

            ```
            # nonlinear integrodifferential volterra equation
            u'=-2*u+exp(-2*t)+int{exp(-t)#u^2}
            init u=1
            aux utrue=exp(-t)
            done
            ```

            Finally, integral equations with singularities are easily solved. For
            example

            *u(t)=exp(-t)-int[e^-(t-s)^(t-s)^-¼^
            u(s),s=0..t]*

            This has a removeable singularity in that the power of **t** is
            less than 1. The ODE file has the form:

            ```
            # singular integral equation
            u(t)=exp(-t)-int[.25]{exp(-t)#sin(u)}
            done
            ```

            WARNINGS 
            1. The expression inside the **[ ]** must be a number and not a
               parameter.- Volterra integral equations cannot be edited inside XPP.

            ---

            - ### Markov models

              XPP simulates random processes. Here is a stochastic ratchet:

              *dx=z f(x) dt + s dW*

              The variable  **z**  randomly switches back and forth between 0 and
              1 at rates  **r_0_,r_1_**  respectively.  **dW**  is a Wiener process. The function **f(x)** is an asymmetric
              periodic potential with zero mean. Here is the ODE file

              ```
              # a simple thermal ratchet
              wiener w
              par a=.8,s=.05,r0=.1,r1=.1
              f(x)=if(x<a)then(-1)else(a/(1-a))
              x'=z*f(mod(x,1))+s*w
              # z is two states
              markov z 2
              {0} {r0}
              {r1} {0}
              @ meth=euler,dt=.1,total=2000,nout=10
              @ xhi=2000,yhi=8,ylo=-8
              done
              ```

              The rates in a Markov process need not be constant but can depend on
              any of the variables or parameters.  Always use
              Euler's method  for stochastic equations.

              ---

              - ### Delta functions and all that

                XPP has a way of dealing with delta-function discontinuities. XPP uses
                 **global**  flags which check for events and act on variables
                accordingly. Here is an equation due to John Tyson for the cell cycle:

                *u' = k_4_ (v-u)(a + u^2^) - k_6_   
                v' = k_1_ -k_6_ u   
                m' = b m*

                with the condition that when **u** falls below **0.2** the mass
                is halved. We tell XPP to look for crossings of **u** and to cut
                the mass in half. Here is the ODE file

                ```
                # tyson.ode
                init u=.0075,v=.48,m=1
                p k1=.015,k4=200,k6=2,a=.0001,b=.005
                u'=  k4*(v-u)*(a+u^2) - k6*u
                v'= k1*m - k6*u
                m'= b*m
                global 1 {0.2-u} {m=.5*m}
                @ total=1000,nout=10
                @ yp=m,xhi=1000,ylo=0,yhi=2 
                done
                ```

                ---

                - ### Boundary value problems

                  Boundary value problems require that you satisfy conditions at both
                  ends of the integration interval. Consider the boundary value
                  problem:

                  *u'' = sin(t u)   
                  u(0)= 1   
                  u(2) = 0*

                  We rewrite this as a first order system:

                  *u' = v   
                  v' = sin(t u)   
                  u(0)= 1   
                  u(2) = 0*

                  Here is the ODE file for this:

                  ```
                  # nonlinear boundary value problem
                  u'=v
                  v'=sin(t*u)
                  bdry u-1
                  bdry u'
                  init u=1,v=-1
                  @ total=2,bell=0,xhi=2
                  done
                  ```

                  Boundary conditions take the form:

                  ```
                  bdry F(u,u')
                  ```

                  where **u'** represents the value of the variable at the end of the
                  interval.  *It is not the derivative in this
                  context!!*   
                  XPP tries to make the functions defined by each boundary condition
                  vanish. The length of the interval is the total amount of time
                  integrated.
                  Note that in this example, I have turned off the stinking
                  bell.

                  You solve boundary value problems in XPP by using the  **Bdryval** 
                  menu item instead of the **Initialconds** item.

                  XPP can also solve systems with homoclinic orbits. See the manual for
                  an example.

                  ---

                  - ### Discretized PDEs

                    XPP can make fake arrays of variables and save you from typing every
                    one. The Schnakenberg system is a classic model:

                    *u_t_ = -u+vu^2^+d_u_ u_xx_   
                    v_t_ = a - vu^2^+d_v_ v_xx_*

                    which we can discretize yielding:

                    *u'_j_ = -u_j_+v_j_u^2^_j_+(d_u_/h^2^) (u_j+1_-2 u_j_+u_j-1_)   
                    v'_j_ = a - v_j_u^2^_j_+(d_v_/h^2^) (v_j+1_-2 v_j_+v_j-1_)*

                    with appropriate end conditions. We will make this into an ODE file:

                    ```
                    # schnakenberg PDE 100 points
                    f(u,v)=-u+v*u^2
                    g(u,v)=a-v*u^2
                    u0'=f(u0,v0)+duh*(u1-u0)
                    u[1..99]'=f(u[j],v[j])+duh*(u[j+1]-2*u[j]+u[j-1])
                    u100'=f(u100,v100)+duh*(u99-u100)
                    v0'=g(u0,v0)+dvh*(v1-v0)
                    v[1..99]'=g(u[j],v[j])+dvh*(v[j+1]-2*v[j]+v[j-1])
                    v100'=g(u100,v100)+dvh*(v99-v100)
                    par a=1.05,du=.2,dv=3,h=.2
                    !duh=du/(h*h)
                    !dvh=dv/(h*h)
                    init u0=1.5,u1=1.3,u2=1.1,v0=1.05,v1=1.05,v2=1.05
                    init u[3..100]=1.05,v[j]=1.05
                    @ dt=.1,nout=50,total=50,meth=cvode,tol=1e-5,atol=1e-5
                    done
                    ```

                    Note that I have defined the kinetics as a pair of functions. The
                    statements starting with the  **!**  are derived parameters --
                    parameters which depend on other parameters. I use a stiff integrator
                     **CVODE** . Plot this using  array plots
                     or look at the spatial profile with the  transpose  operation.

                    Take advantage of the banded quality of the system and speed up the
                    stiff integrators my an order of magnitude. You have to rearrange the
                    equations a bit. Here is the ODE file:

                    ```
                    # schnakenberg PDE 100 points
                    # interlaced
                    f(u,v)=-u+v*u^2
                    g(u,v)=a-v*u^2
                    u0'=f(u0,v0)+duh*(u1-u0)
                    v0'=g(u0,v0)+dvh*(v1-v0)
                    %[1..99]
                    u[j]'=f(u[j],v[j])+duh*(u[j+1]-2*u[j]+u[j-1])
                    v[j]'=g(u[j],v[j])+dvh*(v[j+1]-2*v[j]+v[j-1])
                    %
                    u100'=f(u100,v100)+duh*(u99-u100)
                    v100'=g(u100,v100)+dvh*(v99-v100)
                    par a=1.05,du=5,dv=75,h=.2
                    !duh=du/(h*h)
                    !dvh=dv/(h*h)
                    init u0=1.5,u1=1.3,u2=1.1,v0=1.05,v1=1.05,v2=1.05
                    init u[3..100]=1.05,v[j]=1.05
                    @ dt=.1,nout=50,total=50,meth=cvode,tol=1e-5,atol=1e-5
                    @ bandlo=2,bandup=2
                    done
                    ```

                    I tell XPP the system is banded and interlace the two variables.
                    This runs significantly faster than
                    the previous ODE!

                    ---

                    - ### Network problems and tables

                      The last example is a solution to the Wilson-Cowan equations
                      distributed in space. We will solve:

                      *u'_j_ = -u_j_ + F(a_ee_K_e,j_
                      -a_ie_ K_i,j_)   
                      v'_j_ = (-v_j_ + F(a_ei_K_e,j_
                      -a_ii_ K_i,j_))/tau*

                      where

                      *K_e,j_ = SUM(W_e_(k)u_j-k_,k=-m..m)  
                      K_i,j_ = SUM(W_i_(k)u_j-k_,k=-m..m)*

                      with some sort of "boundary" conditions at the edges. Here is an XPP file for a network of 201 cells and an exponential weight function:

                      ```
                      # Wilson-Cowan network
                      table we % 51 -25 25 .5*exp(-abs(t)/se)/se
                      table wi % 51 -25 25 .5*exp(-abs(t)/si)/si
                      special ke=conv(even,201,25,we,u0)
                      special ki=conv(even,201,25,wi,v0)
                      par se=4,si=2.5
                      f(u)=1/(1+exp(-u))
                      par aee=16,aie=10,aei=25,aii=3,ze=4,zi=10
                      par tau=4
                      init u[0..4]=1
                      u[0..200]'=-u[j]+f(aee*ke([j])-aie*ki([j])-ze)
                      v[0..200]'=(-v[j]+f(aei*ke([j])-aii*ki([j])-zi))/tau
                      @ total=60,meth=qualrk,dt=.25
                      @ yp=u100,xhi=60,ylo=0
                      @ yp2=u150,nplot=2
                      done
                      ```

                      The weights are defined as tables which give the number of points in
                      the table, the x-range, and the functional form of the table. You can
                      also read a file to fill a table. Consult the documentation for
                      more. The  **special**  declaration convolves the values in the
                      tables with the variables. The  **even**  means that the ends are
                      reflected. Note that you must write  **ke([j])**, that is the  **[
                      ]**  must be included. I have also told XPP to plot two things,  **u100** and  **u150.**
