# Supplementary material for: Impact of Influenza A Virus Infection on Growth and Metabolism of Suspension MDCK Cells Using a Dynamic Model
Source: Metabolites. 2022 Mar 12;12(3):239. doi: 10.3390/metabo12030239 (PMC8950586; doi:10.3390/metabo12030239)
Supplement: Supplementary file 1 [file metabolites-12-00239-s001.zip › Supplementary File S3.zip!/S3_file/Demo_MDCK/SBPOP_PACKAGE_Rev_1176_64bit/SBTOOLBOX2/_ACADEMIC/tools/THIRD_PARTY_SOFTWARE/xppaut/unix_mac/help/xppfile.html]

XPP - FILE

Back | Next | Contents


---

# File

- **(P)rt info**   
   This creates a new window which contains the complete source of the ODE file. If you click on the  **Action**  and have defined some  action  associated with it, then this is how to execute it. Click on  **Source**  to get back the original ODE file.- **(W)rite set**   
     This creates a file with all of the info about the current numerics, etc as well as all of the currently highlighted graphics window. It is easily readable by the user and essentially saves the current state of XPP to be read in later.

    - **(S)ave info**   
       This is like (P)rt info but saves the info to a file. It is human readable but not nearly as comprehensive as the files saved by **Write set**.

      - **(A)uto**   
         This brings up the AUTO window if you have installed it.

        - **(C)alculator**   
           This pops up a little answer window. Type formulae in the command line involving your variables and the results are displayed in the popup.

          To set an initial condition or parameter type x:exp(-1), for example, to set the parameter or variable, x to exp(-1) .

          To exit the calculator, click on **Quit** in the answer window or type **Esc**.

          - **(R)ead set**    
             This reads a set that you have previously written with the **Write set** command. The files is very tightly connected to the ODE file that wrote it, so you should not try to load a saved file from one equation for a different problem or even the same one if you have altered the ODE file.

            - **(Q)uit**   
               This exits XPP first asking if you are sure.

              - **(E)dit**   
                 You can edit the equations from within XPP. ***Note:** XPP is capable of understanding right-hand sides of up to 1000 characters. However, the RHS editor will not accept anything longer than about 72 characters.*  This menu item presents a list of four options:

                - **(R)HS**   
                   Edit the right-hand sides of the ODEs IDEs, and auxiliary variables. If you are happy with the editing, then type  **TAB**  or click on  **OK.**  The program will parse the new equations and if they are syntactically correct, alter the corresponding equation. If there is an error, the you will be told of the offending right-hand-side and that will not be changed.

                  - **(F)unctions**   
                     This lets you alter any user-defined functions. It is otherwise the same as the above.

                    - **(S)ave as**   
                      This creates an ``ODE'' file based on the current parameter values, functions, and right-hand sides. You will be asked for a filename.

                      - **(L)oad DLL**    
                        On systems which allow dynamically linked libraries, you can load in a compiled version of right-hand sides for your ODE.
                        See  linking to C for examples.
                - **(B)ell on/off**    
                   This toggles the stupid noisy bell on and off.

                  - **(T)ranspose**     
                     This allows you to transpose data in the data browser. You may want to do this is you are solving a discretization of a PDE and want to see solutions at a fixed
                    value of time plotted as a function of index. A dialog box comes up asking for
                      
                    - the first column   
                      - the total number of columns   
                        - the skip between columns (eg every 3 cols)   
                          - the first time slice as a row number   
                            - the number of rows   
                              - skip between rows (e.g. every 10th row)

                    Suppose you have data of the form  *[u1(t),...,u20(t)]* for*t=0,0.05, ..., 20.00*  in the data browser. You thus have 401 rows of data. Then if you choose **u1** as the first column, **20** as the number of columns, **1** for the column skip, **100** as the first row, **10** as the number of rows, and **20** as the row skip, the data in the "time" column of the browser is replaced by the numbers 1 to 20
                    representing the index; the column for "u1" is replaced by

                    *[u1(t1),u2(t1),...,u20(t1)]*

                     where  *t1=100\*0.05* . The tenth column,"u10", is replaced by

                    *[u1(t10),u2(t10),...,u20(t10)]*

                     where  *t10=100\*0.05 + 10\*20\*0.05*  since you chose to skip
                    every 20 rows.
