# Supplementary material for: Impact of Influenza A Virus Infection on Growth and Metabolism of Suspension MDCK Cells Using a Dynamic Model
Source: Metabolites. 2022 Mar 12;12(3):239. doi: 10.3390/metabo12030239 (PMC8950586; doi:10.3390/metabo12030239)
Supplement: Supplementary file 1 [file metabolites-12-00239-s001.zip › Supplementary File S3.zip!/S3_file/Demo_MDCK/SBPOP_PACKAGE_Rev_1176_64bit/SBTOOLBOX2/_ACADEMIC/tools/THIRD_PARTY_SOFTWARE/xppaut/unix_mac/help/xppgraph.html]

XPP - GRAPHICS

Back | Next | Contents


---

# Graphics

This menu lets you alter things like linetypes, add multiple curves, freeze simulations, import diagrams, change axes, colormaps, and print.

- To change the view, axes labels, start the animator, or plot arrays,
  click  here 

  - To change the three-d settings click  here 

    - To make a new window  click here 

      - To quickly plot a variable against time  click here

|  |  |
| --- | --- |
| **(A)dd curve  \|** |
| **(D)elete last  \|** |
| **(R)emove all  \|** |
| **(E)dit curve  \|** |
| **(P)ostscript  \|** |
| **(F)reeze  \|** |
| **a(X)es opts  \|** |
| **exp(O)rt  \|** |
| **(C)olormap  \|** |

---

- **(A)dd curve** This lets you add another curve to the picture. A dialog box will appear asking you for   
  - the variables on each axis,- a color and- a line type (1 - solid and 0 - point, negative integers draw circles of various radii)

  All subsequent integrations and restorations will include the new graph. Up to
  10 per window are allowed.

  - **(D)elete last**   
     Will remove most recent curve that was added.

    - **(R)emove all**   
       Deletes all curves but first which can never be deleted.

      - **(E)dit curve**   
         You will be asked for the curve to edit.
        The first is 0, the second 1, etc. You will get a dialog box like the (A)dd curve
        option.

        - **(P)ostscript**  
          This generates a postscript file for the XPP window including nullclines, text, and all graphs. A new dialog box will come up asking for   
          - whether you want black and white or color   
            - whether you want landscape or portrait   
              - the fontsize of the axes text and numbers

          You are then asked for a file name for the postscript file.

          - **(F)reeze**
             
          This will create a permanent curve in the window. Usually,
          when you reintegrate the equations or load in some new data, the current curve
          will be replace by the new data. Freeze prevents this. Up to 26 curves can be
          frozen per window.

          There are several options:

          - **(F)reeze**    
             This freezes the current curve 0 for the current plotting window. It will not be plotted in other windows. If you change the axes from 2 to 3 dimensions and it was frozen as a 2D curve (and  *vice versa*  ) then it will also not be plotted. It is better to create another window to work in 3 dimensions so this is avoided.

            A parameter box pops up that asks you for the color (linetype) as well as the key name and the curve name. The curve name is for easy reference and should be a few characters. The key name is what will be printed on the graph if a key is present.

            - **(D)elete**   
               This gives you a choice of available curves to delete.

              - **(E)dit**   
                 This lets you edit a named curve; the key, name, and linetype can be altered.

                - **(R)emove all**   
                   This gets rid of all of the frozen curves in the current window.

                  - **(K)ey**   
                     This turns the key on or off. If you turn it on, then you can position it with the mouse on the graph. The key consists of a line followed by some text describing the line. Only about 15 characters are permitted.

                    - **(B)if.diag**
           
         will prompt you for a filename and then using the
        current view, draw the diagram.

        The file must be of the same format as
        is produced by the  **Write pts**  option in the AUTO menus.

        The diagram is colored according to whether the points are stable/unstable fixed points or periodics. The diagram is ``frozen'' and there can only be one diagram at a time.

        - **(C)lr. BD**   
           clears out the current bifurcation diagram.

          - **(O)n freeze**    
             Toggles a flag that automatically freezes the
            curves as you integrate them. Use this to permanantly freeze everything
            that you draw

- **a(X)es opts**  
  This puts up a window which allows you to tell XPP where you want the axes to be drawn, whether you want them, and what fontsize to make the PostScript axes labels.

  - **exp(O)rt**  
     This lets you save the points that are currently plotted on the screen in XY format. Thus if you have a phaseplane on the screen, only the X and Y values are saved. This makes it
    compatible with programs like XMGR which assume  X Y1 Y2 ...  data. If you have several traces on the screen at once, it saves the X values of the first trace and the Y values of the first and all subsequent
    traces. Frozen curves are not exported.

    - **(C)olormap**  
       This lets you choose a different color map from the
      default. There are a bunch of them; try them all and pick your
      favorite.
