# Supplementary material for: Impact of Influenza A Virus Infection on Growth and Metabolism of Suspension MDCK Cells Using a Dynamic Model
Source: Metabolites. 2022 Mar 12;12(3):239. doi: 10.3390/metabo12030239 (PMC8950586; doi:10.3390/metabo12030239)
Supplement: Supplementary file 1 [file metabolites-12-00239-s001.zip › Supplementary File S3.zip!/S3_file/Demo_MDCK/SBPOP_PACKAGE_Rev_1176_64bit/SBTOOLBOX2/_ACADEMIC/tools/THIRD_PARTY_SOFTWARE/xppaut/unix_mac/help/xpphalf.html]

XPP - MAKE WINDOWS

Back | Next | Contents


---

# Make window

The option allows you to create and destroy graphics windows. There are several choices.

- **(C)reate**   
   makes a copy of the currently active window and makes itself active. You can change the graphs in this window without affecting the other windows.

  - **(K)ill all**   
     Removes all but the main graphics window.

    - **(D)estroy**   
      This destroys the currently active window. The main window cannot be destroyed.

      - **(B)ottom**   
         puts the active window on the bottom.

        - **(A)uto**   
           turns on a flag so that the window will automatically be redrawn when needed.

          - **(M)anual**   
             turns off the flag and the user must restore the picture manually.

            - **(S)imPlot on/off**    
               lets you plot the solution in all active windows while the simulation is running. This slows you down quite a bit.

After a window is created, you can use the mouse to find the
coordinates by pressing and moving in the window. The coordinates are
given near the top of the window.
