# Supplementary material for: Impact of Influenza A Virus Infection on Growth and Metabolism of Suspension MDCK Cells Using a Dynamic Model
Source: Metabolites. 2022 Mar 12;12(3):239. doi: 10.3390/metabo12030239 (PMC8950586; doi:10.3390/metabo12030239)
Supplement: Supplementary file 1 [file metabolites-12-00239-s001.zip › Supplementary File S3.zip!/S3_file/Demo_MDCK/SBPOP_PACKAGE_Rev_1176_64bit/SBTOOLBOX2/_ACADEMIC/tools/THIRD_PARTY_SOFTWARE/xppaut/unix_mac/help/xpphelp.html]

 XPP - MAIN 


# XPP Help

## Writing ODE files

## Reserved words and functions

## Example ODE files

## Using the browser

## Using the animator

## AUTO

## Common commands --- Menu items | | | | --- | --- | | (I)nitialconds Initial conditions/Integration \| | | (C)ontinue Continue an integration longer \| | | (N)ullcline Draw Nullclines \| | | (D)ir.field/flow Direction fields and flows \| | | (W)indow/zoom Window and Zoom \| | | ph(A)sespace Setting up toroidal phase-spaces \| | | (K)inescope Making movies \| | | (G)raphic stuff Graphics settings, keeping curves \| | | n(U)merics Numerical settings/Post processing data, Poincare maps, etc \| | | (F)ile Editing, saving, calculator,quitting, etc \| | | (P)arameters Set problem parameters \| | | (E)rase Erasing the screen \| | | (M)akewindow Multiple windows \| | | (T)ext,etc Drawing text and other objects in a window \| | | (S)ing pts Computing fixed points and stability \| | | (V)iewaxes Setting 2D and 3D views \| | | (X)i vs t Plot a variable versus time \| | | (R)estore Redrawing the window \| | | (3)d-params Three-d parameters \| | | (B)ndryval Running the boundary-value solver \| | ---
