# Supplementary material for: Impact of Influenza A Virus Infection on Growth and Metabolism of Suspension MDCK Cells Using a Dynamic Model
Source: Metabolites. 2022 Mar 12;12(3):239. doi: 10.3390/metabo12030239 (PMC8950586; doi:10.3390/metabo12030239)
Supplement: Supplementary file 1 [file metabolites-12-00239-s001.zip › Supplementary File S3.zip!/S3_file/Demo_MDCK/SBPOP_PACKAGE_Rev_1176_64bit/SBTOOLBOX2/_ACADEMIC/tools/THIRD_PARTY_SOFTWARE/xppaut/unix_mac/help/xppics.html]

XPP - INITAL CONDITIONS

Back | Next | Contents


---

# Initial Conditions

This lets you set initial data and then integrates the equations. Ypou can also set initial data by typing into the initial condition window.

To solve boundary-value problems using shooting, click  here 

---

|  |  |
| --- | --- |
| **(R)ange  \|** |
| **(2)par range  \|** |
| **(L)ast  \|** |
| **(O)ld  \|** |
| **(G)o  \|** |
| **(M)ouse  \|** |
| **(S)hift  \|** |
| **(N)ew  \|** |
| **s(H)oot  \|** |
| **(F)ile  \|** |
| **form(U)la  \|** |
| **m(I)ce  \|** |
| **(D)AE guess  \|** |
| **(B)ackward  \|** |

---

- **(R)ange**
   
This lets you
integrate multiple times with the results shown
in the graphics window. Pressing this option produces a new window with
several boxes to fill in.

First choose the quantity you want to range
over. It can be a parameter or a variable. The integrator will be called
and this quantity will be changed at the beginning of each integration.

Then choose the starting and ending value and the number of steps.

The
option **Reset storage** only stores the last integration. If you choose not
to reset, each integration is appended to storage. Most likely, storage
will be exceeded and the integration will overwrite or stop.

The option
to use last initial conditions will automatically use the final result of
the previous integration as initial data for the next integration. Otherwise,
the current ICs will be used at each step (except of course for the variable
through wich you are ranging.)

If you choose  **Yes**  in the  **Movie**  item,
then after each integration, XPP will take a snapshot of the picture.
You can then replay this series of snapshots back using the Kinescope.

When you are happy with the parameters, simply
press the  **OK** button. Otherwise, press the  **Cancel**  button to abort.

Assuming
that you have accepted, the program will compute the trajectories and plot
them storing none of them or all of them. If you press  **Esc** it will abort the
current trajectory and move on to the next.

Pressing the  **/**  key will abort
the whole process.

- **(2)par range**   
   Similar to range integration, but allows you to
  range over two items.

  The  Crv(1) Array(2)  item determines how
  the range is done. If you choose  Crv  then the two paramaters are
  varied in concert, *[a(i),b(i)]* for *i=0,...,N*.

  The more useful
   Array varies them independently as *[a(i),b(j)]* for
  *i=0,...,N* and  *j=0,...,M.*

  - **(L)ast**    
     This uses the end result of the most recent integration as the starting point of the current integration.

    - **(O)ld**    
       This uses the most recent initial data as the current initial data. It is essentially the same as Go.

      - **(G)o**    
         Uses the initial data in the IC window and the current
        numerics parameters to solve the equation.

        The output is drawn in the
        current selected graphics window and the data are saved for later use.

        The
        solution continues until either the user aborts by pressing  **Esc** , the
        integration is complete, or storage runs out.

        - **(M)ouse**    
           allows you to specify the values with the mouse. Click at the
          desired spot in a phase-plane picture. You must have a two-dimensional
          view and only variables along the axes.

          - **(S)hift**    
             This is like  **Last**  except that the stating time is shifted to the current integration time. This is irrelevant for autonomous systems but is useful for nonautonomous ODEs.

            - **(N)ew**   
               This prompts you at the command line for each
              initial condition.

              Press  **Enter**  to accept the value presented. Press **Esc**  to quit entering and start the integration.

              - **s(H)oot**  
                 allows you to use initial data that was produced when
                you last searched for an equilibrium.

                When a rest state has a single
                positive or negative eigenvalue, then XPP will ask if you want to
                approximate the invariant manifold. If you choose  yes  to this,
                then the initial data that were used to compute the trajectories are
                remembered. Thus, when you choose this option, you will be asked for
                a number 1-4. This number is the order in which the invariant
                trajectories were computed.

                1 and 2 are always unstable manifolds and 3 and 4 are stable manifolds.

                Note if the invariant set is a stable
                manifold, then you should integrate backwards in time.

                - **(F)ile**   
                   Prompts you for a file name which has the initial data
                  as a sequence of numerical values.

                  - **form(U)la**   
                     Allows you to set all the initial data as a
                    formula. This is good for systems that represent chains of many ODEs.

                    When prompted for the variable, type in  u[2..10]  for example to
                    set the variables  u2,u3, ..., u10  and then put in a formula
                    using the index  [j] .

                    Note you must use  [j] and not  j  by itself.

                    For example  sin([j]\*2\*pi/10). Repeat this for
                    different variables hitting enter twice to begin the integration.

                    - **m(I)ce**   
                       allows you to choose multiple points with the
                      mouse. Click  **Esc**  when done.

                      - **(D)AE guess**   
                         lets you choose a guess for the algebraic variables of the DAE.

                        - **(B)ackward   
                           is the same as  Go  but the integration is run backwards in time.**
