# Supplementary material for: Impact of Influenza A Virus Infection on Growth and Metabolism of Suspension MDCK Cells Using a Dynamic Model
Source: Metabolites. 2022 Mar 12;12(3):239. doi: 10.3390/metabo12030239 (PMC8950586; doi:10.3390/metabo12030239)
Supplement: Supplementary file 1 [file metabolites-12-00239-s001.zip › Supplementary File S3.zip!/S3_file/Demo_MDCK/SBPOP_PACKAGE_Rev_1176_64bit/SBTOOLBOX2/_ACADEMIC/tools/THIRD_PARTY_SOFTWARE/xppaut/unix_mac/help/xppkine.html]

XPP - KINESCOPE

Back | Next | Contents


---

# Kinescope

This allows you to capture the bitmap of the active window and play it back.

- **(C)apture**  
   takes a snapshot of the currently active window

  - **(R)eset**  
     deletes all the snapshots

    - **(P)layback**  
       cycles thru the pictures each time you click the left mouse button and stops if you click the middle or tap  **Esc** . It will beep when done.

      - **(A)utoplay**  
         continuously plays back snapshots. You tell it how many cycles and how much time between frames in milliseconds.

        - **(S)ave**   
           saves the frames in either ppm or gif format

          - **(M)ake anigif**   
             Create an animated gif from the frames. The file is always called **anim.gif** and can be played with Netscape, Explorer, xanim, etc.
