# Supplementary material for: Impact of Influenza A Virus Infection on Growth and Metabolism of Suspension MDCK Cells Using a Dynamic Model
Source: Metabolites. 2022 Mar 12;12(3):239. doi: 10.3390/metabo12030239 (PMC8950586; doi:10.3390/metabo12030239)
Supplement: Supplementary file 1 [file metabolites-12-00239-s001.zip › Supplementary File S3.zip!/S3_file/Demo_MDCK/SBPOP_PACKAGE_Rev_1176_64bit/SBTOOLBOX2/_ACADEMIC/tools/THIRD_PARTY_SOFTWARE/xppaut/unix_mac/help/xppncline.html]

XPP - NULLCLINES

Back | Next | Contents


---

# **Nullclines**

This option allows you to draw the
nullclines of systems. Nullclines are curves where the right-hand
sides vanish. Their intersections show fixed points.
They are most useful for two-dimensional
models, but XPP lets you draw them for any model. The constraints are
the same as in the direction fields option.
The menu has 6 items:

- **(N)ew**  draws a new set of nullclines.

  - **(R)estore**  restores the most recently computed set.

    - **(A)uto**  turns on a flag that makes XPP redraw them every
      time it is necessary because some other window obscured them.

      - **(M)anual**  turns this flag off so that you must restore them manually. The X-axis nullcline is red and the Y-axis nullcline is green.

        - **(F)reeze**  allows you to freeze and play back multiple nullclines

          - **(F)reeze**  freezes the current nullclines

            - **(D)elete all**  deletes all frozen nullclines

              - **(R)ange**  lets you vary a parameter through some range,
                computes the nullclines and stores them

                - **(A)nimate**  redraws all the frozen nullclines erasing the
                  screen between each one. The user specifies a delay between
                  drawing.
          - **(S)ave** saves the nullclines into a file. They can then be
            plotted using other software.

            **NOTE:**  The way that XPP computes
            nullclines (by computing zero contours of a two-variable function)
            means that the nullclines are composed of a series of small line
            segments. This means that if you try to plot them as a continuous
            curve, your plotting program will produce garbage. Thus, you should
            plot them as points and not draw line segments between them. The data
            file produced has 4 columns. The first two are the **x**  nullcline and
            the second two are the **y** nullcline.

            Data is as follows:

            ```
            xnx1 xny1 ynx1 yny1
            xnx2 xny2 ynx2 yny2
            ...
            ```

            There are an even number of entries.

            The **x**  nullcline consists of
            segments  (xnx1,xny1),(xnx2,xny2)  between pairs of
            points. Similarly for the **y** nullcline.
