# Supplementary material for: Impact of Influenza A Virus Infection on Growth and Metabolism of Suspension MDCK Cells Using a Dynamic Model
Source: Metabolites. 2022 Mar 12;12(3):239. doi: 10.3390/metabo12030239 (PMC8950586; doi:10.3390/metabo12030239)
Supplement: Supplementary file 1 [file metabolites-12-00239-s001.zip › Supplementary File S3.zip!/S3_file/Demo_MDCK/SBPOP_PACKAGE_Rev_1176_64bit/SBTOOLBOX2/_ACADEMIC/tools/THIRD_PARTY_SOFTWARE/xppaut/unix_mac/help/xppnumerics.html]

XPP - NUMERICS

Back | Next | Contents


---

# Numerics

This menu controls the numerical aspects of the program.

### Press nUmerics from main menu

|  |  |  |  |  |  |  |  |  |  |  |  |  |  |  |  |  |
| --- | --- | --- | --- | --- | --- | --- | --- | --- | --- | --- | --- | --- | --- | --- | --- | --- |
| (T)otal  -- Change total time\| (S)tart time  -- Change start time of simulation\| t(R)ansient  -- Only store after this much time\| (D)t -- Integration timestep\| n(C)line ctrl -- nullcline grid\| s(I)ng pt ctl -- fixed point accuracy\| n(O)ut -- frequency of output\| (B)ounds -- global bounds for all variables\| (M)ethod -- integration method\| d(E)lay -- delay equations params\| (C)olor code -- color code trajectories\| (P)oincare map -- c'mon you should know!\| r(U)elle plot -- make delayed embedding plots\| stoc(H)astic -- all sorts of statistics, curve fitting, miscellany!\| loo(K)up -- change tables\| bndry(V)al -- params for BVP solver\| (A)djoint -- weakly coupled oscillator stuff. \| \| \| \| \| \| \| \| \| \| \| \| \| \| \| \| |

---

- **(T)otal**    
  This is the amount of time
  to integrate. If it is negative then it will be made positive and no
  data will be stored. Thus you can integrate for very long periods of
  time without being told that the storage is full.

  - **(S)tart time**   
    This is the initial time
    **T0**. For autonomous systems it is usually irrelevant.

    - **t(R)ansient**   
      The program will
      integrate silently for this amount of time before plotting output. It
      is used to get rid of transients.

      - **(D)t**   
        This is the step size used by the
        fixed step integrators and the output step for the adaptivve
        algorithms. It is positive or negative depending on the direction of
        integration. That is, to integrate backwards, make it negative.

        - **n(C)line ctrl**   
           will prompt you for the
          grid size for computing nullclines.

          - **s(I)ng pt ctrl**   
             This prompts you for errors and epsilons for eigenvalues and equilibria calculations as well as the maximum iterates.

            - **n(O)utput**   
              This sets the number of integration steps to perform between output to storage for fixed step integrators.
              Thus, if you output every 10 steps with a Dt of .05, XPP will yield output at times that are 10\*.05=.5 timesteps apart. The advantage of this is that lengthier records of data can be made without losing accuracy of the integrator.

              - **(B)ounds**  
                 This sets a global bound on the integrator. If any variable exceeds this value in magnitude, a message appears and the integration stops.

                - **(M)ethod**   
                   allows you to choose the
                  methods of integration. There are many integrators available for use:
                  - **(D)iscrete**  Use this for discrete dynamical systems or
                    maps.- **(E)uler**  is the usual Euler one-step method.- **(M)od. Euler**  is the two-step Euler or Heun method.- **(R)unge-Kutta**  is the fourth order Runge-Kutta method
                          described in every text book- **(A)dams**  is an Adams-Bashforth fourth order
                            predictor-corrector method. It requires fewer right-hand side
                            eva,uations than RK and achieves the same accurace. However, it is
                            notoriously unstable.- **(G)ear**  is a stiff integrator based on the algorithm in
                              Gear's classic text. The step size is adaptive. You need to specify a
                              tolerance (the smaller, the more accurate), a minimum step size, and a
                              maximum step size.- **(V)olterra**  is a backward integrator used for solve
                                Volterra integral equations. It is first order accurate. XPP prompts for
                                a tolerance, maximum iterates, and a ``memory size.'' The memory size
                                sets the storage used by the solver. If the kernel decays rapidly,
                                you can make this small. Tolerance and maximum iterates are used to
                                advance to the next step. You will also
                                be asked if you want the convolution kernels to be re-evaluated after
                                any parameter is changed in either the range integration or through a
                                manual change in parameters. If this flag is 1 then the kernels will
                                be re-computed. The default is to not recompute them.- **(B)ackEul**  is a simple backward Euler
                                  integrator. Smaller tolerances imply tighter requirements for the
                                  Newton's method and maximum iterates specifies how many Newton
                                  attempts will be made.- **(Q)ualst.RK4**  is an adaptive stepsize Runge-Kutta
                                    integrator recommended for nonstiff systems. Choose small tolerances
                                    for increased accuracy and a minimum/maximum allowable step size.- **(S)tiff**  is another adaptive stiff integrator similar to
                                      Gear. You are prompted for error tolerance and maximum/minimum
                                      allowable steps.- **(C)Vode**  a C implementation of LSODE based on Gear. It is
                                        the recommended stiff integrator. Relative and absolute tolerances are
                                        requested. You will be asked if you want to use a banded solver. For
                                        systems like discretized PDEs, using banded solvers can increase the
                                        speed by factors of 10 to 100. The upper and lower bands will be requested.- **DoPri(5)**  Dormand-Prince adaptive step size integrator
                                          for nonstiff systems. Absolute and relative tolerances are requested.- **DoPri(8)3**  is a higher order DP integrator. Absolute and
                                            relative tolerances are requested.- **Rosen(2)3**  is a Rosenbrock adaptive integrator for stiff
                                              systems. Tolerances and bandedness are requested,- **s(Y)mplect**  is a symplectic integrator for conservative
                                                systems of the form:

                                                y_1_'' = f_1_(y_1_,...,y_n_)

                                                y_2_'' = f_2_(y_1_,...,y_n_)

                                                ...

                                                y_n_'' = f_n_(y_1_,...,y_n_)

                                                - **d(E)lay**  
                                                   This sets the upper bound for
                                                  the maximum delay as well as some other parameters used in computing
                                                  the stability of fixed points for delay equations. The first pair are
                                                  guesses for the eigenvalue with maximal real part and the  **grid**  is for the integration around a contour to determine how many
                                                  eigenvalues there are with positive real part using the argument
                                                  principle. If in your delay
                                                  equations, the delay exceeds the  **maximal delay**  , a message will appear and the
                                                  integration will stop. Each time the  **maximal delay** 
                                                  is changed all previous delay
                                                  data is destroyed and you must begin your integration anew. Thus, it
                                                  should be the first thing you set when solving a delay equation.
                                                  Since the storage depends on the size of Dt when you change this, then
                                                  the delay storage will also be destroyed. Delay equations require
                                                  data for  *t <t0*  so that you should edit the Delay ICs to
                                                  achieve this. Use caution when integrating with adaptive step-size
                                                  integrators.

                                                  - **(C)olor code**  
                                                     If you have a color system,
                                                    XPP can code the output according to the magnitude of the velocity or
                                                    the magnitude of a different quantity. A choice pops up for these two
                                                    options or for turning off the color. This overides any color on any
                                                    other curves in your picture. Once you choose to color code, you are
                                                    asked to either choose max and min values or have XPP do it for you
                                                    via optimize. The latter will compute the max and min and set the
                                                    scales accordingly.

                                                    - **(P)oincare map**   
                                                      This sets up
                                                      parameters for Poincare sections. A choice of four items will appear:
                                                      the **Max/Min**  option, the **Poincare section** option,  **period**  option, and
                                                      the option to turn  **off** all maps. Only sections orthogonal to
                                                      the coordinates are allowed, however, by defing the appropriate
                                                      auxiliary variables, you can make sections for any quantity.
                                                      The  **Max/Min**  finds local extrema of variable and the  **Period**  option records the time between events in the  **Time**  column as well as the local section info.
                                                      A window will pop up and you should
                                                      type in the parameters. They are the variable to check and the
                                                      section and the direction. That is, a point will be recorded if the
                                                      variable crosses the section such that it is either positive going to
                                                      negative or vice versa according to the direction parameter. If the
                                                      direction is set to zero, the, the point will be recorded from either
                                                      direction. The flag **Stop on Section** instructs XPP to halt when
                                                      the section is crossed. Note that automatic interpolation is done.
                                                      If the section variable is  **Time, T**  and the section is say
                                                       *T1*,then each time *T=0 modulo T1* the point is recorded.
                                                      This is useful for periodically driven systems. If you have opted for
                                                      the  **Max/Min**  option, then the section is irrelevant and the
                                                      point will be recorded when a local maximum (if the direction is 1)
                                                      minimum (direction=-1) or both (direction=0) of the variable is
                                                      encountered.

                                                      - **stoc(H)astic**  
                                                        This brings up a series of items that allow you to compute many trajectories and find their mean and variance. It is most useful when used with systems that are either Markovian or have noise added to the right-hand sides. The items are:

                                                        - **New seed**   
                                                          Use this to reseed the random number generator. If you use the same seed then the results will not change from run to run.

                                                          - **Compute**   
                                                            This will put up the same dialog box as the ``Integrate'' ``Range'' choice. Two new data sets will be created that will compute the mean and the variance of the point by point values of the trajectories over the number of trial runs you choose. If the system is completely deterministic and the parameters and initial conditions are identical for each run, then this is superfluous. Otherwise, the mean and variance are computed. You can then access these new arrays as described below. If you fire up the sample Markov problem, choose the ``Compute'' option, and set keep the initial data constant over say 20 runs, then you can look at the mean trajectory and its variance for each of your variables.

                                                            - **Data**   
                                                              This puts the results of the most recent run into the data browser and enables plotting of them.

                                                              - **Mean**   
                                                                This puts the results of the mean value of the most recently computed set of trials.

                                                                - **Variance**   
                                                                  This does the same for the variance.

                                                                  - **Histogram**  
                                                                     This computes a histogram for a chosen variable and additional conditions and replaces the ``t'' column and
                                                                    the first variable column with the bin values and the number per bin respectively. You will be prompted for the number of bins, a maximum and minimum value and the variable on which to perform the histogram. Finally, you will be asked for additional conditions that involve the other stored variables (not the fixed ones though.) For example, suppose you have run an ODE/Markov system and you want the distribution of a continuous variable when the Markov variable is in state 1. Then the additional condition would be z==1 where z is the Markov variable. Multiple conditions are made by using the & and | expressions. Note that == is the logical equal and is not the same as the algebraic one.

                                                                    - **Old Hist**   
                                                                      brings back the most recently computed histogram.

                                                                      - **Fourier**   
                                                                         This prompts you for
                                                                        a data column and the number of modes you want. It then
                                                                        computes a Fourier transform (not an FFT, since I don't want
                                                                        to worry about zero padding and other matters) for the number
                                                                        of modes you have chosen. The results are in the Browser.
                                                                        The first column (labeled ``T'') is the mode. The second, the
                                                                        cosine component and the third, the sine component.

                                                                        - Power    
                                                                           This is just like  **Fourier** 
                                                                          except the magnitude and phase of the FFT are kept.
                                                                          - **f(I)t curve**   
                                                                             This is a routine based on
                                                                            Marquardt-Levenberg algorithm for nonlinear least squares fitting. A description of the method can be found in  *Numerical Recipes in C.* In this implementation, one can choose parameters and initial data to vary in an attempt to minimize the least-squares difference between solutions to a dynamical system and data. The data must be in a file in which the first column contains the independent values in increasing order. The remaining columns contain data which are to be fitted to solutions to a DE. Not all the columns need be used. When you choose this option, a window pops up with 10 entries describing the fit parameters. The items are:
                                                                            - **File** This is the name of the data file. The first column must contain the times at which the data was taken.- **NCols** This contains the total number of columns in the
                                                                                data file. This includes all columns in the file, even those that you will not use.- **Fitvar** This is a list separated by commas or spaces of variables that you want to fit to the data. These must not be Markov variables or Auxiliary variables. They are restricted to the items that you define as ``Variables'' in the ODE file. Due to laziness, you can only have as many variables as you can type in 25 or fewer characters.- **To Col** This should contain a list of column numbers in the data file associated with each of the variables you want to fit. Thus, for example, if you want to fit ``x'' to column 5 and ``y'' to column 2, you would type ``x y'' in the ``Fitvar'' entry and ``5 2'' in the ``To Col'' entry. The number of columns in this must equal the number of variables to be fit.- **Params** These items (there are 2 of them in case you have lots
                                                                                      of parameters you need to vary) contain the names of parameters and variables. If the name is a variable, then the initial data for that variable will be adjusted. If it is a parameter, then the parameter will be adjusted. On the initial call, the current initial data and parameter values are used. The lists of parameters and initial data can be separated by spaces or commas.- **Tolerance** This is just a small number that tells the algorithm when the least square error is not changing enough to be significant. That is if either the difference is less than ``TOL'' or the ratio of the difference with the least square is less than ``TOL'' then the program will halt sucessfully. One should not make this too small as such differences are insignificant and a waste of CPU time. The default of .001 seems to work well.- **Npts** This is the number of points in the data file that you
                                                                                          want to fit to.- **Epsilon** This is used for numerical differentiation. 1e-5 is
                                                                                            a good value since we really don't need precise derivatives.- **Max iter** This is the maximum number of iterates you should use
                                                                                              before giving up.
                                                                            On return, the program will put the best set of parameters that it has
                                                                            found. It currently is quite verbose and prints a lot of stuff to the
                                                                            console. This is mainly info about the current values of the
                                                                            parameters and the least square.- **Stat**   brings up two choices:  **Mean/Dev**  which
                                                                              computes the mean and standard deviation of a given column of numbers;
                                                                              and  **autoCor**  which computes the autocorrelation within a
                                                                              column of data. You should give a number for the bins and for the max
                                                                              and min. It is a symmetric function.- **Liapunov**   computes the maximal Liapunov exponent for the
                                                                                current simulation. If you choose to compute it over a range of
                                                                                parameters, the parameter and the maximal exponent are stored in the
                                                                                first two columns of the browser.

                                                        - **R(U)elle plot**   
                                                           This allows you to retard any of the axes by an integral number of steps. This is useful for chaotic orbits and delayed systems. Choosing a number for any of the axes will result in the variable associated with that axis being delayed by the number of steps inputted. Thus if you plot X vs X then of course you will get a diagonal line, but if you make the Y-axis delayed by say 50 and the output is every .1 timesteps, then the plot will be X(t-5) vs X(t). This does not appear during integration of the equations and is available only after a computation. You set it up and then click  Restore  from the main menu.

                                                          - **loo(K)up**   
                                                             This allows you to change the definitions of tabulated functions by reading in a different file. Thus, if you have many experimental sets of data, you can read them in one by one and integrate the equations. You are prompted for the name of a tabulated function. Then you give the filename to read in. You will continue to be prompted and can type a few carriage returns to get out. If the table was defined as a function instead of a file, then you will be prompted for the number of points, the limits of the range (Xhi,Xlo) ad finally, the formula of for the function defining the table. Note that it must be a function of t. Note that if the
                                                            function contains parameters and these are changed, it will be automatically recomputed.

                                                            - **bndry(V)al**   
                                                              prompts you for the maximum iterates, the error tolerance, and the deviation for the numerical Jacobian for the shooting method for solving BVPs.

                                                              - **(A)djoint**  
                                                                 This allows you to compute
                                                                the adjoint and do averaging for weakly coupled oscillators. To use
                                                                this option, you must successfully compute a periodic orbit and set
                                                                the total integration time to one full orbit. If you are only
                                                                interested in the adjoint of a single component of an oscillation, the
                                                                algorithm works best if you start at a maximum of that component.
                                                                The menu that pops up is:
                                                                - **(N)ew adj** This makes the adjoint from the
                                                                  computed periodic data. Success or failure will be noted. Separate
                                                                  storage is maintained for the adjoint. The program automatically
                                                                  puts the data from the adjoint into the browser so it can be
                                                                  viewed and plotted or saved. If  **Out of bounds**  increase
                                                                  the  bounds. - **(A)djoint** This will place the adjoint data in the browser,- **(O)rbit** This places the periodic orbit in the browser.- **(M)ake H** This will prompt you for the coupling function
                                                                        and the result will be averaged and placed in 2 columns of
                                                                        storage, the first is the time, then the H function. If there are
                                                                        enough columns, the odd and even
                                                                        parts of the averaged function will be retained. As with the adjoint,
                                                                        this list is automatically placed in the browser. The user will be
                                                                        prompted for the coupling functions and should type in the
                                                                        formulas. The coupling is between two identical units. Say you
                                                                        want to couple two oscillators via a variable called V Then, you
                                                                        V refers to the oscillator getting the input
                                                                        and V' refers to the oscillator providing it. So, e.g, for diffusive
                                                                        coupling you would type in  V'-V - **(H) function** This places the computed H function in the browser.
                                                                Anytime you integrate, etc, the data will be placed back into the storage area.
