# Supplementary material for: Impact of Influenza A Virus Infection on Growth and Metabolism of Suspension MDCK Cells Using a Dynamic Model
Source: Metabolites. 2022 Mar 12;12(3):239. doi: 10.3390/metabo12030239 (PMC8950586; doi:10.3390/metabo12030239)
Supplement: Supplementary file 1 [file metabolites-12-00239-s001.zip › Supplementary File S3.zip!/S3_file/Demo_MDCK/SBPOP_PACKAGE_Rev_1176_64bit/SBTOOLBOX2/_ACADEMIC/tools/THIRD_PARTY_SOFTWARE/xppaut/unix_mac/help/xppopt.html]

OPTIONS


# Options

**XPP** enables the user to incorporate
a large number of options within an ODE file. These have the form:

```
@ opt1=value,opt2=value2,
```

where opt1 is the name of an option and value is the
assigned value. Almost every option can also be set within **XPP** from
the menus. Similarly, most items that you want to set in **XPP** can be
set in the ODE file.

The following options *can
only be set outside the program.* They are:

- MAXSTOR=integer sets the total number of time steps that
  will be kept in memory. The default is 5000. If you want to perform
  very long integrations change this to some large number.
- BACK= {Black,White} sets the background to black or white.
- SMALL=fontname where fontname is some font available
  to your X-server. This sets the ``small'' font which is used in the
  Data Browser and in some other windows.
- BIG=fontname sets the font for all the menus and popups.
- SMC={0,...,10} sets the stable manifold color
- UMC={0,...,10} sets the unstable manifold color
- XNC={0,...,10} sets the X-nullcline color
- YNC={0,...,10} sets the Y-nullcline color
- OUTPUT=filename sets the filename to which you want to write for
  ``silent'' integration. The default is ``output.dat''.
- BUT=name:kbs defines button on the top of the main window. You
  can define up to 20 such buttons. They will be colored yellow and when
  you press them, they will execute an XPP keyboard shortcut. For
  example,  **BUT=Fit:wf** will create a button labeled **Fit**
  and when you proess it, it will be as if you had clicked  **Window/zoom Fit.** Most of the menu items are available.

The remaining options can be set from within the program. They are

:   Plotting options.

    - LT=int sets the linetype. It should be less than 2 and
      greater than -6.
    - XP=name sets the name of the variable to plot on the x-axis.
      The default is T, the time-variable.
    - YP=name sets the name of the variable on the y-axis.
    - ZP=name sets the name of the variable on the z-axis (if the plot
      is 3D.)
    - NPLOT=int tells XPP how many plots will be in the opening
      screen.
    - XP2=name,YP2=name,ZP2=name tells XPP the variables on the axes
      of the second curve; XP8 etc are for the 8th plot. Up to 8 total plots
      can be specified on opening. They will be given different colors.
    - AXES={2,3} determine whether a 2D or 3D plot will be
      displayed.
    - PHI=value,THETA=value set the angles for the three-dimensional
      plots.
    - XLO=value,YLO=value,XHI=value,YHI=value set the limits for
      two-dimensional plots (defaults are 0,-2,20,2 respectively.) Note that
      for three-dimensional plots, the plot is scaled to a cube with
      vertices that are **± 1** and this cube is rotated and projected onto
      the plane so setting these to **± 2** works well for 3D plots.
    - XMAX=value, XMIN=value, YMAX=value, YMIN=value, ZMAX=value, ZMIN=value set
      the scaling for three-d plots.
:   Numerical options

    - SEED=int sets the random number generator seed.
    - TOTAL=value sets the total amount of time to integrate the
      equations (default is 20).
    - DT=value sets the time step for the integrator (default is 0.05).
    - NJMP=integer *or* NOUT=integer
      tells **XPP** how frequently to output the
      solution to the ODE. The default is 1, which means at each
      integration step. This is also used to specify a the period for maps in
      the continuation package AUTO.
    - T0=value sets the starting time (default is 0).
    - TRANS=value tells XPP to integrate until T=TRANS and then
      start plotting solutions (default is 0.)
    - NMESH=integer sets the mesh size for computing nullclines
      (default is 40).
    - {BANDUP=int, BANDLO=int} sets the upper and lower limits for
      banded systems which use the banded version of the CVODE integrator.
    - METH={
      discrete,euler,modeuler,rungekutta,adams,gear,volterra, backeul,
      qualrk,stiff,cvode,5dp,83dp,2rb,ymp}
      sets the integration method (see below; default is Runge-Kutta.) The
      latter four are the Dormand-Prince integrators and a Rosenbrock (2,3)
      integrator and a symplectic integrator.
    - DTMIN=value sets the minimum allowable timestep for the Gear
      integrator.
    - DTMAX=value sets the maximum allowable timestep for the Gear
      integrator
    - VMAXPTS=value sets the number of points maintained in for the
      Volterra integral solver. The default is 4000.
    - { JAC\_EPS=value, NEWT\_TOL=value, NEWT\_ITER=value} set
      parameters for the root finders.
    - ATOLER=value sets the absolute tolerance for several of the
      integrators.
    - TOLER=value sets the error tolerance for the Gear, adaptive RK,
      and stiff integrators. It is the relative tolerance for CVODE and the
      Dormand-Prince integrators.
    - BOUND=value sets the maximum bound any plotted variable can
      reach in magnitude. If any plottable quantity exceeds this, the
      integrator will halt with a warning. The program will not stop
      however (default is 100.)
    - DELAY=value sets the maximum delay allowed in the integration
      (default is 0.)
    - AUTOEVAL={0,1} tells XPP whether or not to automatically
      re-evaluate tables everytime a parameter is changed. The default is
      to do this. However for random tables, you may want this off. Each
      table can be flagged individually within XPP.
:   Poincare map

    - POIMAP={ section,maxmin,period}  sets up a Poincare map for
      either sections of a variable, the extrema, or period between events.
    - POIVAR=name sets the variable name whose section you are
      interested in finding.
    - POIPLN=value is the value of the section; it is a floating
      point.
    - POISGN={ 1, -1, 0 } determines the direction of the
      section.
    - POISTOP=1 means to stop the integration when the section is
      reached.
:   Range integration

    - RANGE=1 means that you want to run a range integration (in batch
      mode).
    - RANGEOVER=name, RANGESTEP, RANGELOW, RANGEHIGH, RANGERESET=Yes,No, RANGEOLDIC=Yes,No all correspond to the entries in the
      range integration option.
:   Phasespace

    - TOR\_PER=value, defined the period for a toroidal phasespace and
      tellx XPP that there will be some variables on the circle.
    - FOLD=name, tells XPP that the variable <name> is to be
      considered modulo the period. You can repeat this for many variables.
:   AUTO options. The following AUTO-specific variables can also be
    set: NTST, NMAX, NPR, DSMIN, DSMAX, DS, EPSS,EPSL,EPSU, PARMIN,PARMAX, NORMMIN,
    NORMMAX, AUTOXMIN, AUTOXMAX, AUTOYMIN, AUTOYMAX, AUTOVAR. The last
    is the variable to plot on the y-axis. The x-axis variable is always
    the first parameter in the ODE file unless you change it within AUTO.
:   Miscellaneous

    - AUTOEVAL={1,0} tells **XPP** whether or not to recompute tables whenever parameters are changed. If you have a table of random connectivuty, you want this turned off (0).
    - BELL=0 turns of the bell for **XPP**.
    - COLORMAP={0,1,2,3,4,5} switches the color map:

      :   0: standard
      :   1: periodic
      :   2: ``hot''
      :   3: ``cool''
      :   4: red-blue
      :   5: grey
