# Supplementary material for: Impact of Influenza A Virus Infection on Growth and Metabolism of Suspension MDCK Cells Using a Dynamic Model
Source: Metabolites. 2022 Mar 12;12(3):239. doi: 10.3390/metabo12030239 (PMC8950586; doi:10.3390/metabo12030239)
Supplement: Supplementary file 1 [file metabolites-12-00239-s001.zip › Supplementary File S3.zip!/S3_file/Demo_MDCK/SBPOP_PACKAGE_Rev_1176_64bit/SBTOOLBOX2/_ACADEMIC/tools/THIRD_PARTY_SOFTWARE/xppaut/unix_mac/help/xpppara.html]

XPP - PARAMETER EDIT

Back | Next | Contents


---

# Parameters

Type the name of a parameter to change. Repeat for more
parameters. Hit **Enter** a few times to exit. Type **default** to get back the values when you started XPP.  Use the parameter window or the **sliders** -- its easier!
