# Supplementary material for: Impact of Influenza A Virus Infection on Growth and Metabolism of Suspension MDCK Cells Using a Dynamic Model
Source: Metabolites. 2022 Mar 12;12(3):239. doi: 10.3390/metabo12030239 (PMC8950586; doi:10.3390/metabo12030239)
Supplement: Supplementary file 1 [file metabolites-12-00239-s001.zip › Supplementary File S3.zip!/S3_file/Demo_MDCK/SBPOP_PACKAGE_Rev_1176_64bit/SBTOOLBOX2/_ACADEMIC/tools/THIRD_PARTY_SOFTWARE/xppaut/unix_mac/help/xppphase.html]

XPP - PHASE SPACE

Back | Next | Contents


---

# Phase Space

XPP allows for periodic domains so that you can solve equations on a torus or cylinder.

You will be prompted to make

- (A)ll variables periodic,- (N)o variables periodic or- (C)hoose the ones you want.

You will be asked for the period which is the same for all periodic variables (if they must be different, rescale them)

Choose them by name or by clicking the
appropriate names from the list presented to you. An  **X**  will
appear next to the selected ones. Clicking toggles the  **X** .

Type  **Esc** when done or CANCEL or DONE.

XPP mods your variables by this period and is smart
enough when plotting to not join the two ends.
