# Supplementary material for: Impact of Influenza A Virus Infection on Growth and Metabolism of Suspension MDCK Cells Using a Dynamic Model
Source: Metabolites. 2022 Mar 12;12(3):239. doi: 10.3390/metabo12030239 (PMC8950586; doi:10.3390/metabo12030239)
Supplement: Supplementary file 1 [file metabolites-12-00239-s001.zip › Supplementary File S3.zip!/S3_file/Demo_MDCK/SBPOP_PACKAGE_Rev_1176_64bit/SBTOOLBOX2/_ACADEMIC/tools/THIRD_PARTY_SOFTWARE/xppaut/unix_mac/help/xppsing.html]

XPP - EQILIBRIUM POINTS

Back | Next | Contents


---

# Equilibrium Points

This allows you to calculate equilibria for a system. There
are three options.

- **(G)o**   
   Begins the calculation using the values in the initial data box as a first guess. Newton's method is applied. If a value is found XPP tries to find the eigenvalues and asks you if you want them printed out. If so, they are written to the console. Then if there is a single real positive or real negative eigenvalue, the program asks you if you want the unstable or stable manifolds to be plotted. Answer yes if so and they will be approximated. The calculation will continue until either a variable goes out of bounds or you press **Esc**. If **Esc** is pressed, the other branch is computed. (Unstable manifolds are yellow and computed first followed by the stable manifolds in color turquoise.) The program continues to find any other invariant sets until it has gotten them all. These are stored and can be accessed by clicking  Initalconds|Shoot. Once an equilibrium is computed a window appears with info on the value of the point and its stability. The top of the window tells you the number of complex eigenvalues with positive, **(c+)** , negative  **(c-)**, zero  **(im)**  real parts and the number of real positive  **(r+)**  and real negative  **(r-)** eigenvalues. If the equation is a difference equation, then the symbols correspond the numbers of real or complex eigenvalues outside  **(+)**  the unit circle or inside  **(-)** . This window remains and can be iconified.

  - **(M)ouse**   
     This is as above but you can specify the initial guess by clicking the mouse. Only the two variables in the two-D window will reflect the mouse values. This is most useful for 2D systems.

    - **monte(C)ar**   
       This lets you randomly select guesses for fixed points. You are asked whether you want to append the new list to an older list, the number of guesses, and ranges for the variables.

      - **(R)ange**   
         This allows you to find a set of equilibria over a range of parameters. A parameter box will prompt you for
        - the parameter,- starting and ending values,- number of steps;- Column for stability will record the stability of the equilibrium point in the specified column.- Shoot at each; if you choose this
                  the invariant manifolds will be drawn for each equilibrium computed.

        The stability can be read as a decimal number of the form **u.s** where  **s**  is the number of stable and **u** the number of unstable eigenvalues. So  **2.03**  means 3 eigenvalues with negative real parts (or in the unit circle) and 2 with positive real parts (outside the unit circle.)

        For delay equations, if a root is found, its real part is included in this column rather than the stability summary since there are infinitely many possible eigenvalues.

        The result of a range calculation is saved in the data array and replaces what ever was there. The value of the parameter is in the time column, the equilibria in the remaining columns and the
        stability info in whatever column you have specified.

        **Esc** aborts one step and **/** aborts the whole procedure.
