# Supplementary material for: Impact of Influenza A Virus Infection on Growth and Metabolism of Suspension MDCK Cells Using a Dynamic Model
Source: Metabolites. 2022 Mar 12;12(3):239. doi: 10.3390/metabo12030239 (PMC8950586; doi:10.3390/metabo12030239)
Supplement: Supplementary file 1 [file metabolites-12-00239-s001.zip › Supplementary File S3.zip!/S3_file/Demo_MDCK/SBPOP_PACKAGE_Rev_1176_64bit/SBTOOLBOX2/_ACADEMIC/tools/THIRD_PARTY_SOFTWARE/xppaut/unix_mac/help/xpptext.html]

XPP - TEXT

Back | Next | Contents


---

# Text, etc

Allows you to write text to the display in a variety
of sizes and in two different fonts. You can also add other symbols
to your graph.

- **(T)ext**   
   This prompts you for the text you want to add.
  You are asked for the size; there are five choices (0-5): 0-8pt,
  1-12pt, 2-14pt, 3-18pt, 4-24pt. (Note that not all X servers will have these
  fonts, but the postscript file will still draw them.) Finally, place
  the text with the mouse. There are a number of escape sequences that
  allow you to change fonts and add super- and subscripts. The escape
  sequences are:
  - **\0**  Use the normal Times-Roman font- **\1** Use the Symbol font- **\s** Subscript- **\S** Superscript- **\n** Normal - no sub- or superscripts

  - **(A)rrow**   
     This lets you draw an arrow-head to indicate a direction on a trajectory. You will be prompted for the size, which should be some positive number, usually less than 1. Then you must move the the mouse and select a direction and starting point. Click on the starting point and holding the mouse buttton down, drag the mouse to indicate the direction of the arrow-head. Then release the mouse-button and the arrow will be drawn.

    - **(P)ointer**   
       This is like an arrow, but draws the stem as well as the arrow head. It can be used to point to important features of your graph. The prompts are like those for **Arrow**.

      - **(E)dit**   
         This lets you edit the text,arrows, and pointers in one of three ways:

        - **(M)ove**   
           lets you move the object to another location without changing any of its properties. Choose the object with the mouse by clicking near it. You will then be prompted as to whether you want to move the item that XPP selected. If you answer yes use the mouse to reposition it.

          - **(C)hange**   
             lets you change the properties: for text, the text itself, size, and font can be change; for arrows and pointers, only the size of the arrow head can be changed. As above, select the object with the mouse and then edit the properties.

            - **(D)elete**   
               deletes the object that you select with the mouse.
        - **(D)elete All**  
          Deletes all the objects in the current window.

          - **marker(S)**  
            This is similar to the Marker command, but allows you to automatically mark a number of points along a computed trajectory. Use the data browser to move the desired starting point of the list to the top line of the browser. Then click on this command and choose a size and color. Then tell XPP how many markers and how many browser lines to jump between markers. (Thus, 10 would put a marker at every 10th data point).
