# Supplementary material for: Impact of Influenza A Virus Infection on Growth and Metabolism of Suspension MDCK Cells Using a Dynamic Model
Source: Metabolites. 2022 Mar 12;12(3):239. doi: 10.3390/metabo12030239 (PMC8950586; doi:10.3390/metabo12030239)
Supplement: Supplementary file 1 [file metabolites-12-00239-s001.zip › Supplementary File S3.zip!/S3_file/Demo_MDCK/SBPOP_PACKAGE_Rev_1176_64bit/SBTOOLBOX2/_ACADEMIC/tools/THIRD_PARTY_SOFTWARE/xppaut/unix_mac/help/xppview.html]

XPP - VIEW AXES

Back | Next | Contents


---

# View axes

This selects one of 2 different types of graphs: 2D or 3D or brings up a new animation window or a space-time array window

If you select the 2D curve, a dialog box will appear asking you for  

- the variables to place on the axes;- upper and lower limits of the two axes as in the  window  command; and- the labels for the axes

If you select 3D, a more complicated dialog box appears asking for:

- the variables to place on the axes;- their max and min values;- 4 more numbers, **XLO**, etc. XPP first scales the data to fit into a cube with corners (-1,-1,-1) and (1,1,1). Rotation of this cube is performed and then projected into the two-D window. **XLO**, etc define
      the scales of this projection and are thus unrelated to the values of your data. Use the Window|Fit option to have XPP set these for you.- Axes labels
