# Supplementary material for: Impact of Influenza A Virus Infection on Growth and Metabolism of Suspension MDCK Cells Using a Dynamic Model
Source: Metabolites. 2022 Mar 12;12(3):239. doi: 10.3390/metabo12030239 (PMC8950586; doi:10.3390/metabo12030239)
Supplement: Supplementary file 1 [file metabolites-12-00239-s001.zip › Supplementary File S3.zip!/S3_file/Demo_MDCK/SBPOP_PACKAGE_Rev_1176_64bit/SBTOOLBOX2/_ACADEMIC/tools/THIRD_PARTY_SOFTWARE/xppaut/unix_mac/help/xppwind.html]

XPP - WINDOW

Back | Next | Contents


---

# Window

Allows you to rewindow the current graph. Pressing this presents another menu with the choices:

- **(W)indow**   
   A window pops up prompting you for the values for the max and min of the two axes.
  Press OK or CANCEL when done.

  - **(Z)oom in**   
     Use the mouse to expand a region by clicking, dragging and releasing. The view in the rectangle will be expanded to the whole window.

    - **Zoom (O)ut**  
       As above but the whole window will be shrunk into the rectangle.

      - **(F)it**  
         The most common command will automatically fit the window so the entire curve is contained within it. For three-D stuff the window data will be scaled to fit into a cube and the cube scaled to fit in the window.

        **USE THIS OFTEN!**

( NOTE: On some displays, no rubber boxes are drawn for the zooming operations. If this occurs, run with the  **-xorflag**  option on the command line.)
