# Supplementary material for: Impact of Influenza A Virus Infection on Growth and Metabolism of Suspension MDCK Cells Using a Dynamic Model
Source: Metabolites. 2022 Mar 12;12(3):239. doi: 10.3390/metabo12030239 (PMC8950586; doi:10.3390/metabo12030239)
Supplement: Supplementary file 1 [file metabolites-12-00239-s001.zip › Supplementary File S3.zip!/S3_file/Demo_MDCK/SBPOP_PACKAGE_Rev_1176_64bit/SBTOOLBOX2/_ACADEMIC/tools/THIRD_PARTY_SOFTWARE/xppaut/unix_mac/install.pdf]

INSTALLATION

Installation of *XPPAUT* is done either by downloading the source code and compiling it or downloading one of the binary versions. I will give sample installations for UNIX, Windows, and MacOS X. If you are totally clueless at compiling source code, it is best to either have your system administrator install it for you or download a precompiled binary for your computer. There are compiled versions available for Linux, SUN, HP, Windows, and Mac OSX.

1 Installation on UNIX.

1.1 Installation from the source code.

Create a directory called `xppaut` and change to this directory by typing:

```
mkdir xppaut
cd xppaut
```

Step 1. Download the compressed tarred source code `xppaut_latest.tar.gz` into this directory from one of the two URLs:

- http://www.math.pitt.edu/*asymp*bard/xpp/xpp.html
- http://www.cnbc.cmu.edu/≍bard/files.html

Step 2. Uncompress and untar the archive:

```
gunzip xppaut_latest.tar.gz
tar xf xppaut_latest.tar
```

This will create a series of files and subdirectories.

Step 3. Type

```
make
```

and lots of things will scroll by including occasional warnings (that you can safely ignore). If you get no errors, then you probably have succeeded in the compilation. If the compilation stops very quickly, then you probably you will have to edit the Makefile according to the architecture of your computer. Look at the README file and the Makefile which has suggestions for many platforms.

Step 4. If you successfully have compiled the program, then you should have a file `xppaut` in your directory. To see, type

```
ls xppaut
```

If you see something like `xppaut*` listed then you have succeeded. If you don't see this, then the compilation was unsuccessful. Consult the README file for a variety of possible fixes. Also, there are many comments in the Makefiles that are included with the package. I have not yet found a computer on which I cannot compile the program. Common problems are the wrong path to the X Windows libraries, nonexistence of `ranlib` among others.

Step 5. Once you have compiled it, just move the executable to someplace in your path. (The usual is `/usr/local/bin` but you must have root privileges to do this.) *XPPAUT* needs no environment information.

1.2 Installation from binaries.

Some binaries are available at one or both of the above URLs. You should download these as well as the source code above. The source code has many examples and the *XPPAUT* reference manual. Download a binary, e.g., `xppaut4.6_hpux.gz` and uncompress it with the command `gunzip xppaut4.6_hpux.gz` and copy it to the desired directory. The binaries are missing things like the example files and the documentation. Download the source code to get these.

1.3 Additional UNIX setup.

In many systems, the zooming and cursor movement does not always work properly. In these systems, you want to call *XPPAUT* with an additional command line argument, e.g.,

```
xppaut -xorfix file.ode
```

This will usually fix these problems. By default, *XPPAUT* comes up with all the windows available. You may want to have *XPPAUT* come up with all but the main window iconified. To do this, add the command line argument, `-iconify`. On Linux, the standard window manager, `fvwm` does not properly iconify the windows so you should not use this option. You can use the `alias` command in your shell to call *XPPAUT* with these command line arguments. Alternatively, create a text file called, e.g., `myxpp` with the following line in it:

```
\usr\local\bin\xppaut %1 %2 -iconify -xorfix
```

Save the file and make it executable by typing `chmod +x myxpp`. Now if you call `myxpp`, it will have the two command-line options enabled.

2 Native MS Windows NT/95/98/2000

Just download the program `winpp.zip` into a folder, say **wpp** and then use Winzip or a similar program to unzip the file. Create a shortcut to `winpp`. This version does not have all the features of the full version. Furthermore, the

interface is quite different. Most of the equation files will work for this version and most of the standard features are extant. There is a binary X version for Windows which is identical to the full UNIX version and I recommend that you use that instead as this book describes the X version. (See the next section.)

3 X-windows version on Windows.

This is the recommended way to run the program in the Windows environment. It is only slightly more difficult to install. It does not use the Windows API, but works identically to the UNIX version. **NOTE.** If you have only used an X-windows emulator to log into another machine, this may be a bit of a surprise. You can run local programs which are properly compiled X-windows programs right on your PC with the X-emulator running. *You do not have to be on a network to run this program on your Windows PC.*

Before you download *XPPAUT* on a Windows machine, you should have X-windows emulator. There are a number of them available at a cost or as demos. There are at least three that are very inexpensive:

X-WinPro: The demo version runs for 30 minutes at a time and the full version is $90. URL: http://www.labf.com/index.html

X-Win32: The demo version runs for 120 minutes at a time. I use this product at home. Prices range from $50 for students to $200 for corporations. URL: http://www.starnet.com/productinfo/

MI/X: This is the smallest and has the fewest features. The demo lasts for 15 days. The cost is $25. URL:http://www.microimages.com/

They are all pretty simple to install and take up very little disk space. Many universities have site licenses for X-servers such as Exceed (see their site: http://www.hummingbird.com/products/nc/exceed).

Here are the steps to install *XPPAUT* in Windows:

Step 1. Create a folder `tmp` that will be a temporary directory. Create another folder called `xpp`.

Step 2. If you have an X-windows emulator already, then skip this step. Otherwise, you should install one of the above Xservers from the `tmp` directory or the desktop. I have an old version of the MI/X server available to download. If you want to try it, here is how:

- Download the two files into the `tmp` directory: `runme1st.exe` and `file000.bin`.
- Run the program `runme1st.exe` to install an X windows server onto your computer. This server only needs a few megabytes of disk space so it is pretty small. Test the installation by clicking on the START menu and running the program found under TNT Lite.

Step 3. Download the file `xpp4win.zip` into the folder `xpp`. Unzip this with the Winzip utility. There will be a number of files including `xppaut.exe`. Note that there are two dynamic link libraries (DLL's) in the zipped file, so, if you want to move `xppaut.exe` to a different directory, you should move the DLLs there as well. To make it available everywher, you can copy `xppaut.exe`, `cygwin1.dll`, and `libX11.dll` into your Programs directory or any other directory in your path.

Step 4. Test your download.

Step A. Start your X-server.

Step B. Open a MS-DOS prompt from the START menu. Change to the `xpp` directory. (In Windows 2000, this is called Command Prompt. It is available off the Start/Applications menu. If you cannot find it, click on `Run` and type in `command.com`.)

Step C Now you have to tell X where to send the display.

If you are on a network. Type `set DISPLAY=mypc:0.0` where `mypc` is the name of your PC on the network.

If you are not on a network. Type `set DISPLAY=127.0.0.1:0.0`

Note that even on a network, the second command usually works.

Step D You are now ready to run. Type `xppaut lecar.ode` and *XPPAUT* should fire up in the X-window. If not, then check that you have started the X server and set the DISPLAY correctly. Note that, if you get an error `Can't open display` then you should try to find out the name of your PC as that is probably the problem. Another possibility is that your X server won't let your PC host the display. Look for something that allows you to set HOSTS in your X-server and set the host to your display name.

Step E. If successful, exit *XPPAUT* by clicking on the `File` and then the `Quit` entry and answer Yes.

NOTE. My home computer is not on a network, so I have just created a batch file `xpp.bat` and included in it a line that sets the DISPLAY for me:

```
set DISPLAY=127.0.0.1:0.0
C:\xpp\xppaut %1 %2
```

4 Installation on MacOSX

Installation on Macintosh computers running OSX is possible by downloading the source code for *XPPAUT* and then compiling it using the software development tools provided for the new OS. In addition, you will need to download the X development libraries to compile it. The following steps were helpfully

provided to me by Chris Fall and James Sneyd. I have managed to test this on one laptop and everything seems to work. A Mac OSX binary can be found on the web site if you don't want to compile it yourself.

1. Make sure you get and install the full Developer Kit for Mac OSX. This is how you get the cc compiler.

2. Install XFree86 on OSX. Download from

   `ftp://ftp.xfree86.org/pub/XFree86/4.1.0/binaries/Darwin-ppc/`

   Make sure (no matter what the Install file says) that you also get the `Xprog.tgz` bundle. You need it.

3. Get the *xpp* source code and put it in a directory of your choice. I'll assume you've called it xpp. Untar the archive.

4. Make the following changes in the MAC system directories. (I think this is a bug in their header files.)

   - copy `/usr/include/dirent.h` to your xpp directory. I'll assume you've called it `dirent.h` locally.
   - copy `/usr/include/sys/dirent.h` to your xpp directory (giving it a new name obviously. I called it `sysdirent.h`).
   - In the file `read_dir.c` change the `#include <dirent.h>` statement to call your local copy of `dirent.h`, not the one in `/usr/include`.
   - In your local copy of `dirent.h`, change the `#include <sys/dirent.h>` statement to call your local copy of `sysdirent.h`.
   - In your local copy of sysdirent.h, change the lines:

     ```
     u_int32_t d_fileno;              /* file number of entry */
     u_int16_t d_reclen;              /* length of this record */
     u_int8_t  d_type;                /* file type, see below */
     u_int8_t  d_namlen;              /* length of string in d_name */
     ```

     to the new lines:

     ```
     unsigned long d_fileno;              /* file number of entry */
     unsigned short d_reclen;              /* length of this record */
     unsigned char d_type;                /* file type, see below */
     insigned char d_namlen;              /* length of string in d_name */
     ```

     (These occur in the `struct dirent` declaration) and save the file.

5. In the Makefile use the following options

   ```
   CC= cc
   CFLAGS=  -O -DAUTO -DCVODE_YES  -I/usr/X11R6/include
   LDFLAGS= -L/usr/X11R6/lib
   ```

```
AUTLIBS= -lf2c -lX11 -lm
LIBS= -lX11 -lm
OTHERLIBS= libcvode.a libf2cm.a
```

Note that in the subdirectories, `cvodesrc` and `libI77` make sure that `CC=cc`.

Then in the main directory, type `make` and everything should go fine. The rest of the story is like the UNIX installation.
