# Supplementary material for: Impact of Influenza A Virus Infection on Growth and Metabolism of Suspension MDCK Cells Using a Dynamic Model
Source: Metabolites. 2022 Mar 12;12(3):239. doi: 10.3390/metabo12030239 (PMC8950586; doi:10.3390/metabo12030239)
Supplement: Supplementary file 1 [file metabolites-12-00239-s001.zip › Supplementary File S3.zip!/S3_file/Demo_MDCK/SBPOP_PACKAGE_Rev_1176_64bit/SBTOOLBOX2/_ACADEMIC/tools/THIRD_PARTY_SOFTWARE/xppaut/unix_mac/xpp_sum.pdf]

XPP Commands

Bard Ermentrout — Jan 2000

ODE File Format

```
# comment line - name of file, etc
#include filenam
d<name>/dt=<formula>
<name>'=<formula>
<name>(t)=<formula>
volt <name>=<formula>
<name>(t+1)=<formula>
x[n1..n2]' = ...[j] [j-1] ... <--  Arrays
%[i1..i2]
u[j]'=...
v[j]'=...
%
markov <name> <nstates>
  {t01} {t02} ... {t0k-1}
  {t10} ...
  ...
  {tk-1,0} ... {tk-1 k-1}

aux <name>=<formula>
!<name>=<formula> <--  parameters defined as formulae
<name>=<formula>
parameter <name1>=<value1>,<name2>=<value2>, ...
wiener <name1>, <name2>, ...
number <name1>=<value1>,<name2>=<value2>, ...
<name>(<x1>,<x2>,...,<xn>)=<formula>
table <name> <filename>
table <name> % <npts> <xlo> <xhi> <function(t)>
global sign {condition} {name1=form1;...}
init <name>=<value>,...
<name>(0)=<value> or <expr> <--  delay initial conditions
bdry <expression>
0= <expression>    <---  For DAEs
solv <name>=<expression> <------ For DAEs
special <name>=conv(type,npts,ncon,wgt,rootname)
       fconv(type,npts,ncon,wgt,rootname,root2,function)
       sparse(npts,ncon,wgt,index,rootname)
       fsparse(npts,ncon,wgt,index,rootname,root2,function)
# comments
@ <name>=<value>, ...
set <name> {x1=z1,x2=z2,...}
only <name1>,<name2>,...
options <filename>
" {z=3,b=3,...} Some nice text   <---  Active comments
done
```

INTEGRAL EQUATIONS

The general integral equation

$$u(t) = f(t) + \int_0^t K(t,s,u(s))ds$$

becomes

```
u = f(t) + int{K(t,t',u)}
```

The convolution equation:

$$v(t) = \exp(-t) + \int_0^t e^{-(t-s)^2} v(s) ds$$

would be written as:

```
v(t) = exp(-t) + int{exp(-t^2)#v}
```

If one wants to solve, say,

$$u(t) = exp(-t) + \int_0^t (t-t')^{-mu} K(t,t',u(t'))dt'$$

the form is:

```
u(t)= exp(-t) + int[mu]{K(t,t',u}
```

and for convolutions, use the form:

```
u(t)= exp(-t) + int[mu]{w(t)#u}
```

NETWORKS

```
special zip=conv(type,npts,ncon,wgt,root)
```

where `root` is the name of a variable and `wgt` is a table, produces an array `zip` with `npts`:

$$\text{zip}[i] = \sum_{j=-\text{ncon}}^{\text{ncon}} \text{wgt}[j + ncon]\text{root}[i + j]$$

The `sparse` network has the syntax:

```
special zip=sparse(npts,ncon,wgt,index,root)
```

where `wgt` and `index` are tables with at least `npts * ncon` entries. The array `index` returns the indices of the offsets to with which to connect and the array `wgt` is the coupling strength. The return is

```
zip[i] = sum(j=0;j<ncon) w[i*ncon+j]*root[k]
k = index[i*ncon+j]
```

The other two types of networks allow more complicated interactions:

```
special zip=fconv(type,npts,ncon,wgt,root1,root2,f)
```

evaluates as

```
zip[i]=sum(j=-ncon;j=ncon) wgt[ncon+j]*f(root1[i+j],root2[i])
```

and

```
special zip=fsparse(npts,ncon,wgt,index,root1,root2,f)
```

evaluates as

```
zip[i]=sum(j=0;j<ncon) wgt[ncon*i+j]*f(root1[k],root2[i])
k = index[i*ncon+j]
```

Matrix multiplication is also possible:

```
special k=mmult(n,m,w,u)
```

returns a vector `k` of length `m` defined as

```
k(j)=sum(i=0;i<n)w(i+nj)u(i)
```

while

```
special k=fmmult(n,m,w,u,v,f)
```

returns

```
k(j)=sum(i=0;i<n)w(i+nj)f(u(i),v(j))
```

OPTIONS The format for changing the options is:

```
@ name1=value1, name2=value2, ...
```

where `name` is one of the following and `value` is either an integer, floating point, or string. (All names can be upper or lower case). The first four options *can only be set outside the program.* They are:

- MAXSTOR=`integer` sets the total number of time steps that will be kept in memory. The default is 5000. If you want to perform very long integrations change this to some large number.
- BACK= {`Black,White`} sets the background to black or white.
- SMALL=`fontname` where `fontname` is some font available to your X-server. This sets the "small" font which is used in the Data Browser and in some other windows.
- BIG=`fontname` sets the font for all the menus and popups.
- SMC={0,...,10} sets the stable manifold color
- UMC={0,...,10} sets the unstable manifold color
- XNC={0,...,10} sets the X-nullcline color
- YNC={0,...,10} sets the Y-nullcline color

The remaining options can be set from within the program. They are

- LT=`int` sets the linetype. It should be less than 2 and greater than -6.
- SEED=`int` sets the random number generator seed.
- XP=name sets the name of the variable to plot on the x-axis. The default is `T`, the time-variable.
- YP=name sets the name of the variable on the y-axis.
- ZP=name sets the name of the variable on the z-axis (if the plot is 3D.)
- COLORMAP=`0,1,2,3,4,5` sets the colormap
- NPLOT=`int` tells XPP how many plots will be in the opening screen.
- XP2=name,YP2=name,ZP2=name tells XPP the variables on the axes of the second curve; XP8 etc are for the 8th plot. Up to 8 total plots can be specified on opening. They will be given different colors.
- AXES={`2,3`} determine whether a 2D or 3D plot will be displayed.
- TOTAL=value sets the total amount of time to integrate the equations (default is 20).
- DT=value sets the time step for the integrator (default is 0.05).
- NJMP=`integer` tells XPP how frequently to output the solution to the ODE. The default is 1, which means at each integration step.
- T0=value sets the starting time (default is 0).
- TRANS=value tells XPP to integrate until `T=TRANS` and then start plotting solutions (default is 0.)
- NMESH=`integer` sets the mesh size for computing nullclines (default is 40).
- METH={ `discrete,euler,modeuler,rungekutta,adams,gear,volterra, backeul, qualrk,stiff,cvode,5dp,8` sets the integration method (default is Runge-Kutta.)
- DTMIN=value sets the minimum allowable timestep for the Gear integrator.
- DTMAX=value sets the maximum allowable timestep for the Gear integrator

- VMAXPTS=value sets the number of points maintained in for the Volterra integral solver. The default is 4000.
- { JAC_EPS=value, NEWT_TOL=value, NEWT_ITER=value} set parameters for the root finders.
- ATOLER=value sets the absolute tolerance for CVODE.
- TOLER=value sets the error tolerance for the Gear, adaptive RK, and stiff integrators. It is the relative tolerance for CVODE.
- BOUND=value sets the maximum bound any plotted variable can reach in magnitude. If any plottable quantity exceeds this, the integrator will halt with a warning. The program will not stop however (default is 100.)
- DELAY=value sets the maximum delay allowed in the integration (default is 0.)
- BANDUP=`int`, BANDLO=`int` bandwidths for the Jacobian computation.
- PHI=value,THETA=value set the angles for the three-dimensional plots.
- XLO=value,YLO=value,XHI=value,YHI=value set the limits for two-dimensional plots (defaults are 0,-2,20,2 respectively.) Note that for three-dimensional plots, the plot is scaled to a cube with vertices that are ±1 and this cube is rotated and projected onto the plane so setting these to ±2 works well for 3D plots.
- XMAX=value, XMIN=value, YMAX=value, YMIN=value, ZMAX=value, ZMIN=value set the scaling for three-d plots.
- OUTPUT=filename sets the filename to which you want to write for "silent" integration. The default is "output.dat".
- POIMAP={ `section,maxmin, period`} sets up a Poincare map for either sections of a variable, the extrema, or period.
- POIVAR=name sets the variable name whose section you are interested in finding.
- POIPLN=value is the value of the section; it is a floating point.
- POISGN={ `1, -1, 0` } determines the direction of the section.
- POISTOP=1 means to stop the integration when the section is reached.
- RANGE=1 means that you want to run a range integration (in batch mode).
- RANGEOVER=name, RANGESTEP=number, RANGELOW=number, RANGEHIGH=number, RANGERESET=`Yes,No`, RANGEOLDIC=`Yes,No` all correspond to the entries in the range integration option.
- TOR_PER=value, defined the period for a toroidal phasespace and tellx XPP that there will be some variables on the circle.
- FOLD=name, tells XPP that the variable ¡name¿ is to be considered modulo the period. You can repeat this for many variables.
- AUTO-stuff. The following AUTO-specific variables can also be set: `NTST, NMAX, NPR, DSMIN, DSMAX, DS, PARMIN, PARMAX, NORMMIN, NORMMAX, AUTOXMIN, AUTOXMAX, AUTOYMIN, AUTOYMAX, AUTOVAR.` The last is the variable to plot on the y-axis. The x-axis variable is always the first parameter in the ODE file unless you change it within AUTO.

COLOR MEANING 0-Black/White; 1-Red; 2-Red Orange; 3-Orange; 4-Yellow Orange; 5-Yellow; 6-Yellow Green; 7-Green; 8-Blue Green; 9-Blue; 10-Purple.

KEYWORDS You should be aware of the following keywords that should not be used in your ODE files for anything other than their meaning here.

```
sin cos tan atan atan2 sinh cosh tanh
exp delay ln log log10 t pi if then else
asin acos heav sign ceil flr ran abs del\_shft
max min normal besselj bessely erf erfc poisson
arg1 ... arg9  @ $ + - / * ^ ** shift
| > < == >= <= != not \# int sum of i'
```

These are mainly self-explanatory. The nonobvious ones are:

- `heav(arg1)` the step function, zero if `arg1<0` and 1 otherwise.

- `sign(arg)` which is the sign of the argument (zero has sign 0)
- `ran(arg)` produces a uniformly distributed random number between 0 and `arg.`
- `besselj, bessely` take two arguments, n, x and return respectively, $J_n(x)$ and $Y_n(x)$, the Bessel functions.
- `erf(x), erfc(x)` are the error function and the complementary function.
- `normal(arg1,arg2)` produces a normally distributed random number with mean `arg1` and variance `arg2`.
- `poisson(arg)` produces the number of events from a Poisson process with parameter `arg`. It is a random number.
- `max(arg1,arg2)` produces the maximum of the two arguments and `min` is the minimum of them.
- `if(<exp1>)then(<exp2>)else(<exp3>)` evaluates `<exp1>` If it is nonzero it evaluates to `<exp2>` otherwise it is `<exp3>`. E.g. `if(x>1)then(ln(x))else(x-1)` will lead to `ln(2)` if `x=2` and `-1 if x=0.`
- `delay(<var>,<exp>)` returns variable `<var>` delayed by the result of evaluating `<exp>`. In order to use the delay you must inform the program of the maximal possible delay so it can allocate storage.
- `del_shft(<var>,<shft>,<delay>).` This operator combines the `delay` and the `shift` operators and returns the value of the variable `<var>` shifted by `<shft>` at the delayed time given by `<delay>`.
- `ceil(arg),flr(arg)` are the integer parts of`<arg>` returning the smallest integer greater than and the largest integer less than `<arg>`.
- `t` is the current time in the integration of the differential equation.
- `pi` is π.
- `arg1, ..., arg9` are the formal arguments for functions
- `int, #` concern Volterra equations.
- `shift(<var>,<exp>)` This operator evaluates the expression `<exp>` converts it to an integer and then uses this to indirectly address a variable whose address is that of `<var>` plus the integer value of the expression. This is a way to imitate arrays in XPP. For example if you defined the sequence of 5 variables, `u0,u1,u2,u3,u4` one right after another, then `shift(u0,2)` would return the value of `u2.`
- `sum(<ex1>,<ex2>)of(<ex3>)` is a way of summing up things. The expressions `<ex1>,<ex1>` are evaluated and their integer parts are used as the lower and upper limits of the sum. The index of the sum is `i'` so that you cannot have double sums since there is only one index. `<ex3>` is the expression to be summed and will generally involve `i'.` For example `sum(1,10)of(i')` will be evaluated to 55. Another example combines the sum with the shift operator. `sum(0,4)of(shift(u0,i'))` will sum up `u0` and the next four variables that were defined after it.
