# Supplementary material for: Impact of Influenza A Virus Infection on Growth and Metabolism of Suspension MDCK Cells Using a Dynamic Model
Source: Metabolites. 2022 Mar 12;12(3):239. doi: 10.3390/metabo12030239 (PMC8950586; doi:10.3390/metabo12030239)
Supplement: Supplementary file 1 [file metabolites-12-00239-s001.zip › Supplementary File S3.zip!/S3_file/Demo_MDCK/SBPOP_PACKAGE_Rev_1176_64bit/SBTOOLBOX2/_ACADEMIC/tools/THIRD_PARTY_SOFTWARE/xppaut/windows/help/xppodes.html]

xppodes


# Structure of ODE files

All ODE files are just text files which consist of a series of
definitions and directives to the **XPP** parser. The order in which the
definitions are given is usually unimportant with one **major
exception**. So-called fixed or temporary variables are evaluated in
the order in which they are defined. Thus, you should **never** use
a named fix variable before it is defined as this will lead to some
rather bizarre results when you attempt to solve an ODE. ODE files are
all line oriented with a limit of 1024 characters per line. You can
use the standard line continuation symbol  **\** . Here are all
the possible ODE file directives:

:   **#**: comment line. This is ignored by **XPP**

    ---

    :   **''**: A comment that is extracted and can be displayed in a
        separate window.

        ---

        :   **'' {a=1,b=2,...}**: A comment with some ``action''
            associated with it. When these comments are displayed in the comment
            window, an **\***  appears next to them. When clicked by the user,
            various initial conditions, parameters, and XPP internal options can
            be set.

            ---

            :   **!**name=formula This defines a *derived*
                parameter, name whose values could depend on other
                parameters. These do not appear in the parameter list since they are
                presumably tied to the other parameters. Ecah time you change a
                parameter, they also are updated.

                ---

                :   **options** filename: Insert a file name with common
                    options. This is include only for backward compatibility
                    and is generally obsolete.

                    ---

                    :   **name(t+1)=**formula or

                        ---

                        :   **dname/dt=formula** or

                            ---

                            :   **name'=**formula: Define a differential/difference equation
                                dependent variable, **name** and the formula defining its
                                right-handside. For example

                                ```
                                x'=-x+sin(t)
                                z(t+1)=z*(4-z)
                                dw/dt=1-cos(w)+(1+cos(w))*a
                                ```

                                ---

                                :   **name(t)=**formula or

                                    ---

                                    :   **volterra** name = formula: Define a Volterra integral
                                        equation for the variable **name**. For example

                                        ```
                                        y(t)=int{exp(-t)#x}
                                        y'=-y + a int{exp(-(t-t'))*max(y-1/(1+(t-t')^2),0)}
                                        y(t)=int[.5]{1#y}
                                        ```

                                        The  **#**  symbol means a convolution and **[a]** multiplies the integrand by

                                        ### (t-t')^-a^

                                        ---

                                        :   **markov** name nstates

                                            |  |  |  |  |
                                            | --- | --- | --- | --- |
                                            | {0} | {P_0,1_} | **...** | {P_0,N-1_} |
                                            | {P_1,0_} | {0} | **...** | {P_1,N-1_} |
                                            | **...** | **...** | **...** | **...** |
                                            | {P_N-1,0_} | {P_N-1,1_} | **...** | {P_N-1,N-1_} |

                                            This defines a Markov variable, **name** which has nstates
                                            states. Then following is the transition table as a series of formulas
                                            that are delimited by the curly brackets  **{ }** . The diagonal
                                            entries are ignored but should still contain a number or formula. For
                                            example:

                                            ```
                                            markov z 2
                                            {0} {alpha(v)}
                                            {beta(v)} {0}
                                            ```

                                            defines a two-state Markov process with transition from state 0 to 1 determined by the rate  alpha(v)  and the transition from 1 to 0 determoined by  beta(v) 

                                            ---

                                            :   **aux** name=formula defines a named quantity, **name**
                                                which appears in the data browser and is available for plotting. Note
                                                that **auxiliary** variables are not known internally to **XPP** so
                                                that you can't use them in formulas

                                                ---

                                                :   **name=** formula defines an internal or quantity which
                                                    can be used in other formulas. This fixed variable serves the
                                                    same function as temporary quantities in C code.

                                                    ---

                                                    :   **par**  name1=value1, name2=value2, ... defines
                                                        named parameters with default values. These can later be altered and
                                                        varied in **XPP**. **Never put spaces between the name, value, and
                                                        equal sign**. For example:

                                                        ```
                                                        par a=1,b=2,c=2,big_g=20
                                                        ```

                                                    ---

                                                    :   **number**  name1=value1, name2=value2, ... defines
                                                        named parameters with default values. These are fixed and cannot be
                                                        changed in **XPP**. For example:

                                                        ```
                                                        number faraday=96485,rgas=8.3147,tabs0=273.15
                                                        ```

                                                        ---

                                                        :   **name(x,y,...)**=f(x,y,...) defines a function of up
                                                            to 9 variables. This function can be used in any right-hand side or
                                                            other functions. For example:

                                                            ```
                                                            f(x)=if(x>1)then(1/ln(x/(x-1)))else(0)
                                                            g(v,w)=2v^2(1-w)-w/10
                                                            ```

                                                            ---

                                                            :   **table**  name filename defines a lookup table called
                                                                **name** defined the values found in the file, **filename**. This
                                                                behaves as a one variable function which uses linear interpolation on
                                                                the values of the tabulated file. The file has the structure:

                                                                ```
                                                                npts
                                                                xlo
                                                                xhi
                                                                y(xlo)
                                                                ...
                                                                y(xhi)
                                                                ```

                                                                The domain of the function is  **[x_lo_,x_hi_]**

                                                                ---

                                                                :   **table** % name npts xlo xhi f(t) defines a
                                                                    precomputed table called **name** using **npts** and evaluating
                                                                    **f(t)** at  **t=x_lo_,...,x_hi_** 
                                                                    For example:

                                                                    ```
                                                                    table h % 101 0 6.283 sin(t)+.6+.4*cos(t)+.2*sin(2*t)
                                                                    ```

                                                                    Tables are automatically
                                                                    re-evaluated whenever you change parameters unless you turn off the
                                                                    AUTOEVALUATE flag from within  **XPP**  or in the ODE file with

                                                                    ```
                                                                    @ autoeval=0
                                                                    ```

                                                                    ---

                                                                    :   **wiener** name1, name2, ... defines a series of
                                                                        normally distributed random variables with mean 0 and standard
                                                                        deviation  **sqrt(dt)**  where  **dt** 
                                                                        is the internal time step. The
                                                                        advantage of using **wiener** is that when you change the time-step,
                                                                        the magnitude of these variables is automatically adjusted.

                                                                        ---

                                                                        :   **global** sign condition {name1=form1;...} defines a
                                                                            global flag allowing **XPP** to implement delta functions. If **sign=1** and **condition** changes from negative to positive or
                                                                            if **sign=-1** and **condition** changes from positive to
                                                                            negative, or if **sign=0** and **condition** vanishes identically,
                                                                            then each of the variables, **name** is immediately changed to the
                                                                            value of **formula**. For example:

                                                                            ```
                                                                            global 1 x-1 {x=0;y=y+a*sin(y)}
                                                                            ```

                                                                        ---

                                                                        :   **init** name1=val1,name2=val2,... sets the initial
                                                                            data of **name** to the number **val**.

                                                                        ---

                                                                        :   **name(0)**= expression sets the initial value of **name** to the expression. If **name** is a delay-differential
                                                                            equation variable, then if **expression** is a function of time,
                                                                            **t**, then **name(t)** is set to the function for  **-M < t < 0**  where  **M** 
                                                                            is the maximum delay. For example:

                                                                            ```
                                                                            x'=-delay(x,2)
                                                                            x(0)=sin(t)
                                                                            ```

                                                                        ---

                                                                        :   **bdry** expression defines a boundary condition at the
                                                                            start or the end of an interval. If the variable name is primed, then
                                                                            it refers to the end of the interval, otherwise to the start of the
                                                                            interval. For example:

                                                                            ```
                                                                            bdry u'-v-1
                                                                            ```

                                                                            forces the boundary condition **u(L)-v(0)-1=0**
                                                                            where  **[0,L]**  is the
                                                                            interval for the ODE.

                                                                        ---

                                                                        :   **0=** expression defines an algebraic condition for
                                                                            differential-algebric equations.

                                                                        ---

                                                                        :   **solve** name=expression tells **XPP** to solve the
                                                                            algebraic conditions for the variable **name** with initial guess
                                                                            **expression**. For example:

                                                                            ```
                                                                            x'=-y
                                                                            init x=0
                                                                            0=y+exp(y)-x
                                                                            solv y=-.56715
                                                                            ```

                                                                            Solves the DAE **x'=-y**
                                                                            where  **y+e^y=x** 
                                                                            Note that there is no closed
                                                                            form inverse of  **y+e^y.**

                                                                        ---

                                                                        :   **special** name=FUN(...) defines a one-dimensional
                                                                            array **name** that represents a special summation involving an
                                                                            array of variables and a table. **FUN** can be one of the
                                                                            following:

                                                                            - **conv**(type,n,m,w,u):
                                                                                

                                                                              **name(j)= sum(w(m+i)shift(u,j+i),i=-m..m), j=0,...,n-1**  

                                                                              where  **w** 
                                                                              is a  table  of length  **2m+1** 
                                                                              and **type={even,0,periodic}** determines how  **u(j+i)** 
                                                                              is defined for  **j+i <0**  and  **j+1 > n-1** 

                                                                              - **fconv**(type,n,m,w,u,v,f):
                                                                                  

                                                                                **name(j)= sum(w(m+i)f[shift(u,j+i),shift(v,j)],i=-m..m), j=0,...,n-1**  

                                                                                - **sparse**(n,m,w,l,u) evaluates to
                                                                                    

                                                                                  **name(j)= sum(w[j m + i]shift(u,c[j m + i]),i=0..m-1), j=0,...,n-1**  

                                                                                  where  **w** 
                                                                                  is a table of values of length  **m n** 
                                                                                  and  **c** 
                                                                                  is a table of indices of
                                                                                  length  **m n** 

                                                                                  - **fsparse**(n,m,w,l,u,v,f) evaluates to

                                                                                    **name(j)= sum(w[j m + i]f(shift(u,c[j m + i]),shift(v,j)),i=0..m-1), j=0,...,n-1**  - **mmult**(m,n,w,u) evaluates to:
                                                                                        

                                                                                      **name(j)=sum(w(i + m j)\*u(i),i=0..m-1), j=0,...,n-1** 

                                                                                      In other words, it returns the product of an  **n × m**  matrix with a  **m**  dimensional vector.- **fmmult**(m,n,w,u,v,f) evaluates to:
                                                                                          

                                                                                        **name(j)=sum(w(i + m j)\*f(u(i),v(j)),i=0..m-1), j=0,...,n-1**

                                                                            These can then be used in right-hand sides of ODEs. For example

                                                                            ```
                                                                            table w % 21 -10 10 exp(-abs(t))
                                                                            special k=conv(even,101,21,w,u0)
                                                                            u[0..100]'=-u[j]+f(k([j]))
                                                                            ```

                                                                            Here k evaluates as the discrete convolution of  **exp(-|x|)** 
                                                                            with the array  **u.** 

                                                                            ---

                                                                            :   **set** name {x1=z1,x2=z2,...,} defines a named set of
                                                                                declarations including parameter values, initial data, and options,
                                                                                which can be invoked while running **XPP** with the File Get
                                                                                par set command. For example:

                                                                                ```
                                                                                set hopf {a=.3,x=1.2,b=9}
                                                                                ```

                                                                                ---

                                                                                :   **@** opt1=val1,opt2=val2,... sets various internal
                                                                                    **XPP** options. For example:

                                                                                    ```
                                                                                    @ maxstor=100000,total=1000,dt=.02
                                                                                    ```

                                                                                    See  OPTIONS  for a complete list of options.

                                                                                    ---

                                                                                    :   **anything[j1..j2]** expr[j] is expanded by **XPP** into  **j_2_-j_1_+1** 
                                                                                        statements e.g.

                                                                                        ```
                                                                                        x[1..4]'=-x[j]+[j]
                                                                                        ```

                                                                                        is expanded into:

                                                                                        ```
                                                                                        x1'=-x1+1
                                                                                        x2'=-x2+2
                                                                                        x3'=-x3+3
                                                                                        x4'=-x4+4
                                                                                        ```

                                                                                    ---

                                                                                    :   **%[j1..j2]** expands all following statements until
                                                                                        another **%** is encountered as above. For example:

                                                                                        ```
                                                                                        %[1..4]
                                                                                        x[j]'=-y[j]-x[j-1]
                                                                                        y[j]'=x[j]
                                                                                        %
                                                                                        ```

                                                                                        is expanded into:

                                                                                        ```
                                                                                        x1'=-y1-x0
                                                                                        y1'=x1
                                                                                        x2'=-y2-x1
                                                                                        y2'=x2
                                                                                        x3'=-y3-x2
                                                                                        y3'=x3
                                                                                        x4'=-y4-x3
                                                                                        y4'=x4
                                                                                        ```

                                                                                    ---

                                                                                    :   **export**  `{x1,x2,...,xn} {y1,...,yn}`  sends and receives variables and parameters to and from dynamically loaded libraries.
                                                                                         Click here for details.


                                                                                    ---

                                                                                    :   **done** tells **XPP** the file is complete.

---


---


# Reserved words

You should be aware of the following
keywords that should not be used in your ODE files for anything other
than their meaning here.

```
sin cos tan atan atan2 sinh cosh tanh
exp delay ln log log10 t pi if then else
asin acos heav sign ceil flr ran abs del_shft
max min normal besselj bessely erf erfc hom_bcs
arg1 ... arg9  @ $ + - / * ^ ** shift
| > < == >= <= != not \# int sum of i'
```

These are mainly self-explanatory. The nonobvious ones are:

- heav(arg1) the step function, zero if arg1<0 and 1 otherwise.
- sign(arg) which is the sign of the argument (zero has sign 0)
- ran(arg) produces a uniformly distributed random number
  between 0 and arg.
- besselj, bessely  take two arguments,  **(n,x)** 
  and return  **J_n_(x)**  and  **Y_n_(x)** 
  respectively, the Bessel functions.
- erf(x), erfc(x) are the error function and the
  complementary function.
- normal(arg1,arg2) produces a normally distributed random number
  with mean arg1 and variance arg2.
- max(arg1,arg2) produces the maximum of the two arguments
  and min is
  the minimum of them.
- if(<exp1>)then(<exp2>)else(<exp3>) evaluates <exp1> 
  If it is nonzero
  it evaluates to <exp2> otherwise it is <exp3>. E.g. if(x>1)then(ln(x))else(x-1)
  will lead to ln(2) if x=2 and -1 if x=0.
- delay(<var>,<exp>) returns variable <var> delayed by the result of
  evaluating <exp>. In order to use the delay you must inform
  the program of the maximal possible delay so it can allocate storage.
   (see the numerics menu)
- ceil(arg),flr(arg) are the integer parts of<arg> returning the
  smallest integer greater than and the largest integer less than <arg>.
- t  is the current time in the integration of the differential equation.
- pi is **3.1415926...**
- arg1, ..., arg9 are the formal arguments for functions
- int, # concern Volterra equations.
- shift(<var>,<exp>) This operator evaluates the expression
  <exp> converts it to an integer and then uses this to indirectly
  address a variable whose address is that of <var> plus the
  integer value of the expression. This is a way to imitate arrays in
  XPP. For example if you defined the sequence of 5 variables, u0,u1,u2,u3,u4 one right after another, then shift(u0,2) would
  return the value of u2.
- del\_shft(<var>,<shft>,<delay>). This operator combines the
  delay and the shift operators and returns the value of the
  variable <var> shifted by <shft> at the delayed time given
  by <delay>. It is of limited utility as far as I know, but I
  needed it for a problem, so here it is.
- sum(<ex1>,<ex2>)of(<ex3>) is a way of summing up things.
  The expressions <ex1>,<ex1> are evaluated and their integer
  parts are used as the lower and upper limits of the sum. The index of
  the sum is i' so that you cannot have double sums since there is
  only one index. <ex3> is the expression to be summed and will
  generally involve i'. For example sum(1,10)of(i') will
  be evaluated to 55. Another example combines the sum with the shift
  operator. sum(0,4)of(shift(u0,i')) will sum up u0 and the
  next four variables that were defined after it.
- hom\_bcs(<int>)  is used only to tell  **XPP**  about
  heteroclinic and homoclinic connections at boundary points.
