# Supplementary material for: Impact of Influenza A Virus Infection on Growth and Metabolism of Suspension MDCK Cells Using a Dynamic Model
Source: Metabolites. 2022 Mar 12;12(3):239. doi: 10.3390/metabo12030239 (PMC8950586; doi:10.3390/metabo12030239)
Supplement: Supplementary file 1 [file metabolites-12-00239-s001.zip › Supplementary File S3.zip!/S3_file/Demo_MDCK/SBPOP_PACKAGE_Rev_1176_64bit/SBTOOLBOX2/_ACADEMIC/tools/THIRD_PARTY_SOFTWARE/xppaut/windows/xpp_doc.pdf]

XPPAUT5.41 – the differential equations tool

Bard Ermentrout

February 17, 2003

Contents

1 Introduction **5**

Interface 6

Disclaimer 6

Acknowledgments 6

World Wide Web Tutorial 7

1.1 Environment variables. 7

2 ODE Files **8**

Example 8

Quick exploration. 9

ODE File format 10

Options files 12

Include files 12

Defining Continuous Variables 13

Pseudo-arrays 14

Initial data statements 15

Markov Variables 15

Auxiliary quantities 16

Hidden and fixed quantities 17

Differential-Algebraic Equations 18

Parameters, Wiener parameters, Numbers, derived parameters . 19

User-defined functions 20

Tables 20

Global Flags 21

Initial and Boundary conditions 22

Only command 23

Setting internal options 23

Named parameter sets 26

Network functions 27

Active comments 28

Finishing up 29

Important shortcut 29

Reserved words . . . 29
Compatibility . . . 31

3 Command line arguments **31**

4 Examples **32**
Morris-Lecar . . . 32
Cable model . . . 34
Delayed feedback . . . 35
Random mutation . . . 35
Flags and discontinuities . . . 36
Large coupled systems . . . 37

5 Using xpp **38**
Windows . . . 38
Main Window . . . 38
Initial data,delays,parameters . . . 39
Equation window . . . 39
Browse window . . . 39
Parameter sliders . . . 40

6 The main commands **41**
ICs . . . 41
Continue . . . 43
Nullclines . . . 43
Direction Field/Flow . . . 44
Window . . . 44
Phase Space . . . 45
Kinescope . . . 45
Graphics . . . 45
Freezing Curves . . . 46
File . . . 47
Edit right-hand sides . . . 47
Parameters . . . 49
Erase . . . 49
Multiple windows . . . 49
Adding text etc . . . 49
Equilibria . . . 50
Axes . . . 52
Space-time plots . . . 52
Time plots . . . 52
Redrawing . . . 52
3D Parameters . . . 53
3D movies! . . . 53
Boundary values . . . 53

7 Numerical parameters **54**
Time control 54
Iteration control 54
Numerical method 54
Color coding 56
Poincare Section 56
Ruelle embedding plots 56
Stochastic stuff 57
Histogram 57
Fourier analysis 57
Curve Fitting 58
Liapunov exponent 59
Table lookup 59
Averaging 59

8 The Data Browser **60**
Numeric integration/differentiation 61
Writing output 61

9 Functional equations **62**
Automatic evaluation 63
Example 64

10 Auto interface **65**
The AUTO Window 65
10.1 Preparation 65
10.2 Choosing parameters 66
10.3 Diagram axes 66
10.4 Numerical parameters 67
10.5 User functions 68
10.6 Running 68
10.7 Grabbing 69
10.8 Aborting 70
10.9 Saving diagrams 70
10.10Homoclinics and heteroclinics 71

11 Creating Animations **76**
11.1 The animation window 77
11.2 DASL: Dynamical Animation Scripting Language 78
11.3 Examples 81
11.4 MPEG 86

12 Creating C-files for faster simulations **88**
12.1 Dynamically linked libraries 88
12.2 Completely defining the right-hand sides in C 89

13 Some comments on the numerical methods **95**

14 Colors **96**

15 The options file **96**

1 Introduction

XPP (XPPAUT is another name; I will use the two interchangeably) is a tool for solving differential equations, difference equations, delay equations, functional equations, boundary value problems, and stochastic equations. It evolved from a chapter written by John Rinzel and myself on the qualitative theory of nerve membranes and eventually became a commercial product for MSDOS computers called PHASEPLANE. It is now available as a program running under X11 and UNIX.

The code brings together a number of useful algorithms and is extremely portable. All the graphics and interface are written completely in Xlib which explains the somewhat idiosyncratic and primitive widgets interface.

XPP contains the code for the popular bifurcation program, AUTO. Thus, you can switch back and forth between XPP and AUTO, using the values of one program in the other and vice-versa. I have put a "friendly" face on AUTO as well. You do not need to know much about it to play around with it.

XPP has the capabilities for handling up to 590 differential equations. There are solvers for delay and stiff differential equations a well as some code for boundary value problems. Difference equations are also handled. Up to 10 graphics windows can be visible at once and a variety of color combinations is supported. PostScript output is supported. Post processing is easy and includes the ability to make histograms, FFTs and applying functions to columns of your data. Equilibria and linear stability as well as one-dimensional invariant sets can be computed. Nullclines and flow fields aid in the qualitative understanding of two-dimensional models. Poincare maps and equations on cylinders and tori are also supported. Some useful averaging theory tricks and various methods for dealing with coupled oscillators are included primarily because that is what I do for a living. Equations with Dirac delta functions are allowable.

With Version 3.0, I have added an animation package that allows you to create animated versions of your simulations, such as a little pendulum moving back and forth or lamprey swimming. The new window for animations is produced by invoking the `(V)iew axes (T)oon` menu item. See section 11 for complete info.

I will assume that you are well versed in the theory of ordinary differential equations although you need not be to use the program. There are a number of useful features designed for people who use dynamical systems to *model* their experiments. There is a curve-fitter based on the Marquardt-Levenberg algorithm which lets you fit data points to the solutions to dynamical systems. Gnuplot-like graphics and support for some graphics objects such as text, arrows, and pointers are part of the package. You can also import bifurcation curves as part of your graphs. It is possible to automatically generate "movies" of three-dimensional views of attractors or parametric changes in the attractor as some parameters vary. I have also included a small preprocessing utility that allows one to create files for large systems of coupled equations.

There are a number of other such programs available, but they all seem to require that your problems be compiled before using them. XPP does not;

I have devised a simple and fairly fast formula compiler that is based on the idea of the inner interpretor used in the language FORTH (which remains my first love as far as language is concerned) Fear not, the differential equations and boundary conditions and other formulae are written in usual algebraic notation. However, in order to run big problems very quickly, I have written the code so that it is possible to create a library that can be linked to your problem and thus create a binary with the right-hand sides compiled. This can run much faster than the parsed code. See the notes on this at the end of the document.

XPP has been successfully compiled on a SPARC II under OpenLook, a SPARC 1.5 running generic X, a NeXT running X11R4, a DEC 5000, a PC using Linux, and SGI and an HP 730. It also runs under Win95/NT/98 if you have an X-Server. I cannot vouch for other platforms but it has been compiled on the IBM RS6000. Building XPP requires only the standard C compiler, and Xlib. Look at the any README files that come with the distribution for solutions to common compilation problems.

The basic unit for XPP is a single ASCII file (hereafter called an ODE file) that has the equations, parameters, variables, boundary conditions, and functions for your model. You can also include numerical parameters such as time step size and method of integration although these can also be changed within the program. The graphics and postprocessing are all done within the program using the mouse and various menus and buttons. The impatient user should look at some sample `*.ode` files instead of actually reading the documentation. In addition, XPP uses a file called `default.opt` that describes initialization and memory options for the program. This is not necessary as all of the information contained in this file can now be included in ODE file.

Notes on the Interface

This interface is crude by most standards and it is rather ugly as well. I had originally thought to do the whole thing using the OpenLook Widget Set but found that many of the systems to which I wanted to port XPP did not have the set. The present design is completely portable. Someday I will redo it in something like Motif.

The text editing is also somewhat restricted. There is as yet no cut and paste. However, one can use the `Home,End` keys to move the text cursor to the beginning and end of the line. The left and right arrow keys let you move back and forth. You can insert or delete text at any point. **NOTE:** Be careful with the BackSpace and Delete keys as on some systems they are mapped differently. One of them causes the whole line to be erased and the other just erases a single character. Almost every command has a keyboard shortcut. These are given below.

Disclaimer

XPP is distributed as is. The author makes no claims as to the performance of the program. Anyone is allowed to modify and distribute XPP as long as the original code is also made available.

Acknowledgements

The porting of XPP to UNIX and X has been partially supported by NSF in the sense that they gave me the SPARC 2. MSRI in Berkeley gave me use of a lovely office facing the bay and a workstation on which the majority of the port was made in the Summer of 1992. The original version of PhasePlane benefitted by countless colleagues whose endless requests for features and whose constant use led to the version that you see now. Of these I would like to specifically cite John Rinzel and Artie Sherman at the NIH for being the main guinea pigs for the earliest versions.

Please let me know of any bugs or other stuff that you'd like to see incorporated into XPP. I will usually fix them quickly.

My EMAIL address is bard@pitt.edu.

NOTE

The easiest way to get a thorough understanding of the program as well as a short tutorial in dynamical systems is to use the World Wide Web tutorial which can be accessed from my home page at http://www.pitt.edu/≡phase. This tutorial is geared toward computational neuroscientists (in the choice of problems) but provides a fairly detailed introduction to the program.

1.1 Environment variables.

XPP uses some environment variables which you should set in order to call up the online help. You also can make a file in your root directory called `.xpprc` which contains a list of commonly used options. Here are the lines I add to my `.bashrc` file:

```
export XPPHELP=/home/bard/xppnew/help/xpphelp.html
export BROWSER=/usr/bin/netscape
```

When I run XPP on windows, I run the following batch file which serves to set various environment variables:

```
set BROWSER=c:\Program Files\Netscape\Communicator\Program\netscape.exe
set XPPHELP=c:\xppall\help\xpphelp.html
set DISPLAY=127.0.0.1:0.0
set HOME=c:\xppall
xppaut %1 %2 %3
```

Here is my `.xpprc` file:

```
# xpprc file
@ but=quit:fq
@ maxstor=50000,bell=0
@ meth=qualrk,tol=1e-6,atol=1e-6
# thats it
```

2 ODE Files

NOTE. *The latest version of XPP has a greatly improved parser. Any versions 1.6 and above will have this parser built into them. The new parser is compatible with the old format for ODE files so that it will read any old style files. Thus, for users of previous versions, you can still use all of the old files. However, the old style parser will no longer be described in the documentation.* A command line option lets you convert old-style to new style format.

ODE files are ASCII readable files that the XPP parser reads to create machine usable code. Lines can be continued with the standard backslash character, however, the total length of any line cannot exceed 1000 characters.

Example. I will start with a very simple example to get you up and running. The model is the periodically driven Fitzhugh-Nagumo equation:

$$dv/dt = f(v) - w + s(t) + I_0 \quad (1)$$

$$dw/dt = \epsilon(v - \gamma w) \quad (2)$$

$$f(v) = v(1 - v)(v - a) \quad (3)$$

$$s(t) = \alpha \sin \omega t \quad (4)$$

Here is the ODE file:

```
# Forced Fitzhugh-Nagumo fhn.ode
dv/dt = f(v)-w+s(t)+I_0
dw/dt = eps*(v-gamma*w)
f(v)=v*(1-v)*(v-a)
s(t)=al*sin(omega*t)
param a=.25,eps=.05,gamma=1,I_0=.25
param al=0,omega=2
@ total=100,dt=.2,xhi=100
done
```

The file is pretty self-explanatory. The first line cannot contain a number as its first character (this makes the parser think that the format of the ODE file is the old style.) The last line should be the word "done." The names of all parameters must be declared with optional values (the default sets them to 0.) There can be as many as you can fit on each line (up to 200 parameters) but they must be separated by commas or spaces and the "=" sign must have *no* spaces on either side of it.

You could optionally include initial data by adding either of the following sets of lines to the file:

```
init v=.25,w=.3
```

or

```
v(0)=.25
w(0)=.3
```

As you have probably guessed, comments have the form:

```
# This is a comment
```

The "@" sign tells XPP that you want to preset some of the internal parameters for numerical integration and graphing. In this case, we have told XPP to integrate the equations until t=100 with a timestep of 0.25 and to set the high value for the x-axis to 100. You can, of course, change all these internal options from within XPP; this provides an easy way "set" up the problem for "one-button" operation.

Quick exploration

Once you have written an ODE file, you can run XPP by typing

```
xpp ffhn.ode
```

where `ffhn.ode` is the filename you created. You may have to type

```
xppaut ffhn.ode
```

depending on what you named the program. (The distributed source produces a binary called `xppaut` , but I usually create a shell script called `xpp` that contains the following line

```
xppaut $1 -xorfix
```

thus alleviating problems my display has with the exclusive or drawing required for "zooming" in and other things.)

When you type one of the above lines, then the program fires up. It reads the ODE file and if there are any errors, it reports them and returns to the command line. Assuming you haven't written the ODE file wrong (which you shouldn't have since it is included in the source) you should get the main window. Depending on your X windows system, the other windows may or may not be iconified. Almost all command have keyboard shortcuts which are either the first letter of the command or the letter in parentheses or capitalized. Thus, you can oeither use the mouse to click on the command or you can use the keyboard to choose the command. To solve the differential equation with the current parameters, click on `Initialconds` and then `Go`. You will see the variable $V(t)$ plotted across the screen as a function of time. (Instead of using the mouse, you could type `I G` as a keyboard shortcut.) Click on `Xivst`. When the prompt comes up backspace over `V`, and type in `w` and `Enter.` The variable w will be plotted versus time. Note that the vertical axis of the window is automatically adjusted. Click on `Viewaxes` to choose the view and when the new window pops up fill it in as follows:

- `X-axis: V`
- `Y-axis: W`
- `Xmin: -.5`

- `Ymin: 0`
- `Xmax: 1.5`
- `Ymax: 1`
- `Xlabel: V`
- `Ylabel: w`

and then click on `Ok.` The phase-plane will be drawn showing a limit cycle. Click `Nullclines` and then `New` to draw the nullclines. Click `Text,etc` then `Text` and type in "V-nullcline" followed by `Enter` at the prompt. Accept the defaults for text size and font by typing `Enter` twice. Move the mouse pointer to the cubic-like curve and click the button. The text should appear on the screen. Repeat this but type in "w-nullcline" for the text. Click `Graphic stuff` and then `Postscript`. Accept the defaults and a hardcopy postscript file will be produced which you can view or printout on an appropriate printer. Click `File` and then `Quit` and answer `Yes` to exit XPP.

A much more extensive tutorial is available on the World Wide Web (see above). This document is mainly a reference to all the features (bugs :) of XPP.

ODE File format

ODE files consist of ascii readable text which XPP uses to describe the program it wants to solve. The line length is limited to 256 characters total. Individual lines can be continued with the UNIX backslash character, \. ODE files have any combination of the following lines. The order is not too important but can matter (see below).

```
# comment line - name of file, etc
...
#include <filename>
...
options <filename>
...
d<name>/dt=<formula>
<name>'=<formula>
...
<name>(t)=<formula>
...
volt <name>=<formula>
...
<name>(t+1)=<formula>
...
markov <name> <nstates>
{t01} {t02} ... {t0k-1}
{t10} ...
```

```
...
{tk-1,0} ...
...
aux <name>=<formula>
...
<name>=<formula>
...
parameter <name1>=<value1>,<name2>=<value2>, ...
...
!<name>=<formula>
...
wiener <name1>, <name2>, ...
...
number <name1>=<value1>,<name2>=<value2>, ...
...
<name>(<x1>,<x2>,...,<xn>)=<formula>
...
table <name> <filename>
...
table <name> % <npts> <xlo> <xhi> <function(t)>
...
global sign {condition} {name1=form1;...}
...
init <name>=<value>,...
...
<name>(0)=<value> or <expr>
...
bdry <expression>
...
%[i1 .. i2]
...
%
command[i1..i2] ...
...
name[i1..i2] ...
...
0= <expression>
...
solv <name>=<expression>
...
special <name>=conv(type,npts,ncon,wgt,rootname)
       fconv(type,npts,ncon,wgt,rootname,root2,function)
       sparse(npts,ncon,wgt,index,rootname)
               fsparse(npts,ncon,wgt,index,rootname,root2,function)
               mmult(n,m,w,root)
               fmmult(n,m,w,root1,root2,function)
```

```
...
only <name2>,<name2>,...
...
# comments
...
@ <name>=<value>, ...
...
set <name> {x1=z1,x2=z2,...,}
..
" More comments
"  {name=val,...,name=val} active comments
done
```

The typical ODE file contains some or all of the above types of lines. Continuous variables, auxiliary quantities, and Markov variables are all plottable quantities in XPP. That is, once you have solved your equation, you can plot or view any of the continuous and Markov variables or the auxiliary quantities.

Options files. XPP uses a bunch of defaults when it is started up by looking for a file called "default.opt." If it cannot find it, it uses internal options. Alternatively, you can tell XPP the name of the options file you want to use. The description of these files is below. The format for such a statement is:

```
option <filename>
```

which loads the options file specified in `<filename>`. You will probably not want to use this very much as you can now specify all of the parameters in the options file within your ODE file by using the "@" symbol.

Include files. XPP lets you include files in the ODE file so that for example, you can create a library of functions which your XPP ode file can call. Here is an example of two files, the first is called `test.ode` and the second is called `test.inc`:

```
# test.ode
#include test.inc
x'=f(x)
par a=.25
done

# test.inc
f(x)=x*(1-x)*(x-a)
#done
```

The contents of `test.inc` will be included into the ODE file as if you had written

```
# test.ode
f(x)=x*(1-x)*(x-a)
```

```
x'=f(x)
par a=.25
done
```

NOTES: (1) At the end of every include file you have to have the statement `#done` (2) include files can include other files.

Defining Continuous Variables. Variables are the quantities you wish to integrate in time. There are two types of variables: (i) continuous and (ii) Markov. I will first describe continuous variables. Variable names (as can all names in XPP) can have up to 9 letters each. XPP is case insensitive. Any combination of letters and numbers is valid as is the underscore, "_". There are 5 ways that you can tell XPP the names of the continuous variables and their right-hand sides. The following three are equivalent:

```
d<name>/dt=<formula>
<name>'=<formula>
<name>(t)=<formula>
```

Here `<name>` is the name of the variable. The last version is for notational convenience only sincd the `dx/dt` notation makes no sense for discrete dynamical systems. The `<formula>` is exactly that, the formula for the right-hand sides of the equations. These equations can appear anywhere in your file and in any order. However, the order in which they are written determines the order in which they appear in the Data Browser (see below.)

The fourth way of defining a continuous variable is:

```
<name>(t) = <formula>
```

which tells XPP that this defines a Volterra integral equation. (It is distinguished from a definition of some function of the dummy variable `t` by the presence of an integral operator (`int{` or `int[`) in the right-hand side. (see below). For example, the convolution equation:

$$v(t) = \exp(-t) + \int_0^t e^{-(t-s)^2} v(s) ds$$

would be written as:

```
v(t) = exp(-t) + int{exp(-t^2)#v}
```

Integro-differential equations use the `dv/dt` etc notation so that

$$\frac{dv(t)}{dt} = -v(t) + \int_0^t e^{-(t-s)^2} v(s) ds$$

becomes

```
dv/dt= -v+int{exp(-t^2)#v}
```

In the event that the right-hand side does not contain any integral operator (as would be the case, for example, if there was a fixed or hidden variable definition) then you can force the parser into making the equation a Volterra integral equation by typing

```
volt v= exp(-t)+int{exp(-t^2)#v}
```

NOTE. In this format you do not write `v(t)=...` but just `v=...`

Pseudo-arrays. XPP gives you the option of defining many variables at once using an array-like declaration which XPP expands into a set of declarations. For example, you could declare 10 equations with the following command:

```
x[1..10]'=-x[j]
```

and XPP would internally expand this is

```
dX1/dt=-X1
dX2/dt=-X2
dX3/dt=-X3
dX4/dt=-X4
dX5/dt=-X5
dX6/dt=-X6
dX7/dt=-X7
dX8/dt=-X8
dX9/dt=-X9
dX10/dt=-X10
```

Thus, you can make networks and discretizations of PDE's compactly. Note the appearance of the expression `[j]` . This is expanded by XPP to the value of the index. Similarly, the following are allowable indices:

```
[j+n]
[j-n]
[j*n]
```

where `n` is any integer. The `[1..10]` notation tells XPP to start `j` at 1 and go to 10. You can start with any nonnegative integer and end with any. The first number can be less than or greater than the second. XPP does not treat arrays in any efficient manner, it is as if you defined 10 or whatever variables. The names of the variables are the root name with the index appended.

Related to pseudo arrays are array blocks that have (for example) the form

```
%[1..3]
x[j]'=-y[j]
y[j]'=x[j]
init x[j]=1
%
```

This will be expanded as follows:

```
x1'=-y1
y1'=x1
init x1=1
x2'=-y2
y2'=x2
init x2=1
x3'=-y3
y3'=x3
init x3=1
```

which groups the "arrays" along their index rather than along the variable name. This has many disadvantages particularly if you want to put in initial data within the program. However, if you are attempting to solve a discretized version of a partial differential equation that is very stiff, then this has the advantage that the Jacobi matrix that arises from the linearization (required for stiff systems) is banded rather than dense. If you choose CVODE as the integration method (recommended for stiff systems) then there is an option to use the banded version of CVODE. For large systems (say 200 spatial points) this can result in a speed up of the order of 500- to 1000-fold! That is, a problem that would take an hour to integrate, instead takes 4 or 5 seconds. Note that Markov variables cannot be defined in one of these blocks. It will screwup!

Initial data statements. There are two ways to define initial data in the ODE file. Either use the method of typing `init x=1.23,y=423.6 ...` or `x(0)=1.23`. In the latter case, you can also initialize variables that involve delayed arguments. For example, `x(0)=sin(t)` will initialize `x` to be `sin(t)` for `-DELAY < t < 0` where `DELAY` is the maximum delay. WARNING: this has a few bugs in it; the formula `x(0)=t+1` will initialize `x(0)=0` but `x(0)=1+t` will initialze `x(0)=1`. The values for $t < 0$ will be properly evaluated but $t = 0$ will not be.

Markov Variables. Markov variables are finite state quantities that randomly flip from one integer state to another according to the transition probability table that is given in the ODE file. They are treated like variables in that they have initial conditions and are accessible to the user. Markov variables are declared as

```
markov <name> <nstates>
{t01} {t02} ... {t0k-1}
{t10} ...
...
{tk-1,0} ...
...
```

Each Markov variable has its own line which must begin with the letter "m". (All other letters are ignored, but for readability, it is best to write it out.) The name of the variable, `<name>`, and the number of states, `<nstates>`, are included on the first line. The possible values are 0,1, and so on up to $k - 1$ where k is the number of states.

A finite state Markov variable with k states must have associated with it a $k \times k$ matrix, T_{ij} which contains the probability of going from state i to state j per unit of time. Thus the effective transition probability is `DeltaT` times T_{ij}; the larger is `DeltaT` the higher the probability. Since the probabilities must add to 1 in any row, the diagonal terms are automatic and ignored by XPP. If there are k states to the variable, then there must be k rows following the declaration of the transition matrix. Each row contains k entries delimited by curly brackets and separated by spaces. For example, suppose z is a two-state variable with transition probabilities, P_{ij} then it would be defined by:

```
markov z 2
{0} {P01}
{P10} {0}
```

where `P01, P10` are any *algebraic expression or number involving the parameters and variables* of the XPP file. Note that this implies that the state transitions can be dependent on any other quantities.

At each output time step, the probabilities are computed, multiplied by the timestep, and a random number is chosen. If it is in the appropriate range, then the transition will be made. Transitions of several such variables are made in parallel and then each is updated.

Auxiliary quantities. In many cases, you might one to keep track of some combination of your variables. For example, you might want to track the potential energy of a damped pendulum as it swings. They are declared as:

```
aux <name>=formula
```

where `<name>` is the name of the quantity and `<formula>` is the formula for it. *Note that a formula cannot refer to an auxiliary named quantity; use fixed or hidden variables for this.* An example using the auxiliary quantity is the damped pendulum:

$$ml\frac{d^2x}{dt^2} = -mg\sin x - \mu\frac{dx}{dt}$$

with potential energy:

$$P.E. = mg(1 - \cos x)$$

and kinetic energy

$$K.E. = \frac{1}{2}ml(\frac{dx}{dt})^2.$$

Since XPP solves systems of first order equations, this is first converted and results in the ODE file:

```
# damped pendulum
dx/dt = xp
dxp/dt = (-mu*xp-m*g*sin(x))/(m*l)
aux P.E.=m*g*(1-cos(x))
aux K.E.=.5*m*l*xp^2
param m=10,mu=.1,g=9.8,l=1
done
```

where I have also given some values to the parameters.

As with differential equations, you can also define many auxialiar variables at once with a statement like:

```
aux r[1..10]=sqrt(x[j]^2+y[j]^2)
```

which will be expanded in the obvious fashion.

Hidden and fixed quantities. XPP allows you to define intermediate quantities that can be used in the right-hand sides of the equations. They are kept internally by XPP and here, the order in which they are declared matters. They are evaluated in the order in which they are defined, so earlier defined ones should not refer to later defined ones. The format is:

```
<name> = <formula>
```

They are most useful if you want to use a complicated quantity in several right-hand sides. The `<name>` is kept internal to XPP and their values are not stored (unlike variables). *Note that they are different from functions which can take arguments and are not hidden from the user.*

For example, in the pendulum model above, you might also want the total energy:

$$T.E. = K.E. + P.E.$$

Now, as I remarked above, you cannot just add another auxiliary variable using `P.E.` and `K.E.` since they are not known to XPP. But why compute them twice. Here is how to use fixed variables in this example.

```
# damped pendulum pend.ode
dx/dt = xp
dxp/dt = (-mu*xp-m*g*sin(x))/(m*l)
pe=m*g*(1-cos(x))
ke=.5*m*l*xp^2
aux P.E.=pe
aux K.E.=ke
aux T.E=pe+ke
param m=10,mu=.1,g=9.8,l=1
done
```

Both energies are only computed once. (For this example, the performance difference for computing the additional quantity is negligible, but for more complex formulae, fixed quantities are useful.)

Hidden variables can also be declared in groups like ODEs:

```
ica[1..10]=gca*minf(v[j])*(v[j]-eca)
```

this is expanded into 10 declarations:

```
ica1=gca*minf(v1)*(v1-eca)
ica2=gca*minf(v2)*(v2-eca)
...
```

Differential-Algebraic Equations. DAEs can be solved with XPP by combining the `0=` statement with the `solv` statement. A general DAE has the form $F(X, X', W, t) = 0$ where X, W are vector quantities and X' is the derivative of X. XPP treats these in generality but currently cannot integrate past singularities that better integrators such as those found in MANPAK will traverse. I plan to add the MANPAK integrator DAEN1 shortly. In any case, the integrator still handles alot of different problems. The syntax is pretty simple. Algebraic constraints are written using the `0=` command and the algebraic quantities (i.e. those that don't involve derivatives) are defined using the `solv` command. For example:

$$\begin{aligned} x' &= -x \\ 0 &= x + y - 1 \end{aligned}$$

with $(x(0) = 1, y(0) = 0)$ would be written as:

```
# dae_ex1.ode
x'=-x
0= x+y-1
x(0)=1
solv y=0
aux yy=y
done
```

The `solv` statement tells XPP that y is an algebraic quantity and its initial value is 0. The `aux` statement will let you also plot the value of y since it is "hidden" from the user. Here is a more complicated equation which could not be solved by XPP without the DAE stuff:

$$x' + exp(x') + x = 0$$

with $x(0) = -(1+e), x'(0) = 1$. Note that the function $x+exp(x)$ has no closed inverse so that we cannot write this in terms of x'. Here is the ode file:

```
# dae_ex2.ode
x'=xp
0= xp+exp(xp)+x
x(0)=-3.7182
solv xp=1
aux xdot=xp
done
```

Note that we create a dummy algebraic variable called `xp` which is the derivative of x. This is because XPP treats the derivatives in a special manner so we have to accomodate its idiosyncrasies by adding an additional algebraic variable. This last example exploits numerical errors to get the DAE solver to go beyond where it should go legally! It is a relaxation oscillator:

$$\begin{aligned} w' &= v \\ 0 &= v(1 - v^2) - w \end{aligned}$$

with $w(0) = 0, v(0) = 1$. Note that the algebraic equation has multiple roots for some values of w and thus as w groes it must "jump" to a new branch. This cannot happen in a true DAE and in fact, one has to set tolerances low to get the numerical errors to let it work. Here is the next DAE example:

```
#dae_ex3.ode
w'=v_
0= v_*(1-v_*v_)-w
solv v_=1
aux v=v_
@ NEWT_ITER=1000,NEWT_TOL=1e-3,JAC_EPS=1e-5,METH=qualrk
done
```

The important numerical parameters for the DAEs are the maximum iterates, the tolerance for Newton's method, and the epsilon value for computing the Jacobian. These are found in the numerics menu under the menu item SingPt Control.

The DAE algebraic variables are initialized in the ODE file as formulae or constants. However, once integrated, the DAEs retain their current values, not their initial values. To change the initial DAE values, you use the Initialconds menu under the DAE sub menu.

Parameters, Wiener parameters, Numbers, derived parameters. Parameters are named quantities that represent constants in your ODE and which you can change from within the program. The format is:

```
parameter <name1>=<value1>, <name2>=<value2>,...
```

There can be many declarations on each line. It is very important that there be *no spaces* between the `<name>` the `=` sign, and the `value` of the parameter. Without an `=` sign and a value, the parameter is set to zero by default.

Numbers are just like parameters but they are "hidden" from the user; they do not appear in the parameters windows once ypu run the program. Their only advantage is that in a problem with many defined constants, of which only a few can be freely chosen, the parameter window is not cluttered by dozens of parameters.

Derived parameters are also "hidden" from the user and allow you to define constants in terms of other parameters through formulas. Each time you change a parameter, these derived parameters are updated. They differ from "fixed" quantities in that they are not updated at every integration step. As an example, suppose you want to define area in terms of radius and length:

```
par length=50,diam=10
!area=pi*length*diam
```

will create a quantity called `area` that will be altered whenever you change the parameters `length,diam`. Note that the `!` in front of the name tells XPP that this is not a fixed variable and should only be updated when parameters are

changed. Their values can be examined using the calculator by just inputting their names.

Wiener parameters are more properly "functions" that return scaled white noise. They are held fixed for t to $t + dt$ during an integration. At each time step, they are then changed and their value is a normally distributed random number with zero mean and unit variance. The program scales them by the appropriate time step as well. Their purpose is so that one can use methods other than Euler for solving noisy problems. In particular, large steps can be taken using backward Euler without loss of stability.

The declarations:

```
par a[1..5]=.25
wiener w[1..5]
number z[1..5]=.123456
```

behave in the obvious fashion. Note that this expression will lead to an error:

```
par a[1..2]=.5, c=.1234
```

as XPP will expand it into 2 lines:

```
par a1=.5, c=.1234
par a2=.5, c=.1234
```

which will give an "duplicate name" error. On the other hand, this expression will work:

```
par a[1..2]=.25,b[j]=.3
```

and is the same as:

```
par a1=.25,b1=.3
par a2=.25,b2=.3
```

User-defined functions. User defined functions have the following form:

```
<name>(x1,x2,...)=<expression>
```

where `<name>` is the name of the function and `x1,x2,...` are the dummy arguments and `<expression>` is a formula defining the function. There can be at most 9 arguments.

Tables are another type of function but (at least as of now) are only of one argument. The "table" declaration takes one of two forms: (i) file based and (ii) function-based. The file based version has the form:

```
table <name> <filename>
```

allows you to declare a function called `<name>` that reads in values from the file, `<filename>` or as a function of one variable over some interval and then is used in your program as a function of 1 variable interpolated from the tabulated values. The values of this table are assumed to be equally spaced and the file is an ASCII file with the format:

```
<number of values>
<xlo>
<xhi>
y1
y2
.
.
.
yn
```

Thus, $f(xlo) = y1$ and $f(xhi) = yn$. (If the number of points in the file description of the table starts with the ASCII character 'i' , (e.g. i50 instead of 50) then the interpolation will be piecewise constant; otherwise it is linear.) These tables can also be read in from within XPP but a valid table must be given to start the program. This table can be arbitrarily long (as memory permits) and thus you can use experimental data as inputs to differential equations or even sketch curves and use that as your nonlinearity.

You can directly input tabulated functions as well to speed up computations with complicated functions. In this case, after the table name put a parenthesis symbol followed by the number of points, the minimum argument and the maximum argument and then the function. This should be written as a function of "t". Thus, the statement

```
table f % 501 -10 10 tanh(t)
```

will produce a table of the hyperbolic tangent function from -10 to 10 consisting of 501 points.

Using the data browser, you can create tabulated data from a simulation to use later in a different simulation as a function or as input or whatever.

Global Flags. The "global" declaration allows you to set some conditions and then if these conditions hold reset dynamic variables according to the conditions. The form of "global" declarations is:

```
global sign {condition} {name1=form1;...}
```

The `condition` is any combination of variables such that if it is zero, then the desired event has occurred. `sign` is either 1,-1, or 0. A sign of 1 means that if the condition goes from less than zero to greater than zero, the event has occurred. A sign of -1 means that if the condition *decreases* through zero that event has occurred. Finally, a sign of 0, means that any crossing of zero signals an event. Each time the condition is met, events occur. There can be up to 20 events per condition and they are delimited by braces and separated by semicolons. Events are always of the form: `variable=formula` where `variable` is one of the differential equation variables or parameters and formula is some formula involving the variables. All formulae are first evaluated and then the variables are updated. Some examples are shown below.**WARNING!** The global flags are *ignored* by the adams integrator. Use GEAR, EULER, RUNGE-KUTTA, BACKWARD EULER, MODIFIED EULER, STIFF, QUALITY-RK,

DORMAND-PRINCE, ROSENBROCK or CVODE. There is one special event that you can put into the list of events: `out_put=val` if `val>0` then the current value of all variables etc is stored. This allows you to, for example, get faster more accurate Poincare maps.

Note. You will sometimes get the rather obscure message that the program is "Working too hard." This is a diagnostic that one of two things is occurring. First, due to round-off, sometimes the extrapolation to zero for the condition is actually not zero but is instead some very small number. Thus XPP checks for the definition of this which is called `s` and if it is less than a user defined value of `smin` (changed in the numerics menu under "sIng pt ctrl") then it is treated as zero. The console reports this number when the error message occurs so that you can change `smin` to be larger, say 1e-13, to avoid this message.

The second situation that causes this to arise is that the time step `DT` is too large and the same condition is occuring twice in that step. Since the interpolation is linear, this means you should try to take smaller steps; otherwise numerical errors will accumulate.

Global flags can also be defined in groups, for example:

```
global 1 x[1..5]-1 {x[j]=0}
```

Initial and Boundary conditions. The default for initial conditions of Markov and continuous variables is zero. Initial data can also be set in the ODE file (and of course easily set from within XPP) in one of two ways:

```
<name>(0)=value
```

will set the variable `<name>` to the specified value. Alternatively, you can initialize many variables on one line by typing

```
init <name1>=value1, <name2>=value2, ...
```

Boundary conditions can be placed anywhere in the file or ignored altogether if you don't plan on solving boundary value problems. They have the form:

```
bndry <expression>
```

where stands for boundary condition. The expression is one involving your variables and which will be set to zero. In order to distinguish left and right boundary conditions, the following notation is used. For the values of the variables at the left end of the interval, use the symbol for the variable. For the values at the right end of the interval, use the symbol for the variable appended by a single quote, '. Thus to specify the boundary conditions $x(0) = 1$, $y(1) = 2$, the following is used:

```
bndry x-1
bndry y'-2
```

Periodic boundary conditions would be written as:

```
bndry x-x'
bndry y-y'
```

Note that it is not necessary to specify the BCs at this point. They can be specified within the program. Also note that the notation I have used allows the specification of mixed boundary conditions such as periodic BCs. (XPP has some additional special commands for periodic boundary conditions that enable the user to specify fewer equations than usual.) The number of boundary conditions must match the number of variables in your problem. Do not use the boundary value solver with Volterra, delay, stochastic, or discrete equations.

Initial and boundary conditions can also be defined *en masse* via:

```
bdry x[1..2]-2
x[1..2](0)=.345
```

and this will be expanded in the expected fashion.

Only command. This is relevant only if you are using XPP in silent model. The `only` declaration will save to the output only the variables specified by this command.

Setting internal options. XPP has many many internal parameters that you can set from within the program and four parameters that can only be set before it is run. Most of these internal parameters can be set from the "Options" files described above and whose format is at the end of this document. However, it is often useful to put the options right into the ODE file. *NOTE: Any options defined in the ODE file override all others such as the onres in the OPTIONS file.* In addition, there are several options not available in the options file. These options are used by the "silent" integrator to produce a file for output when running without X.

The format for changing the options is:

```
@ name1=value1, name2=value2, ...
```

where `name` is one of the following and `value` is either an integer, floating point, or string. (All names can be upper or lower case). The first four options *can only be set outside the program.* They are:

- MAXSTOR=`integer` sets the total number of time steps that will be kept in memory. The default is 5000. If you want to perform very long integrations change this to some large number.
- BACK= {`Black,White`} sets the background to black or white.
- SMALL=`fontname` where `fontname` is some font available to your X-server. This sets the "small" font which is used in the Data Browser and in some other windows.
- BIG=`fontname` sets the font for all the menus and popups.
- SMC={0,...,10} sets the stable manifold color

- UMC={0,...,10} sets the unstable manifold color
- XNC={0,...,10} sets the X-nullcline color
- YNC={0,...,10} sets the Y-nullcline color
- BUT=s1:s2 defines a user button. You can use this many times in the same ODE file. The first string is the name of the button.The second is the set of key stroke short cuts. For example: `BUT=mouse:im` will put a button on the main window labeled `mouse` and when you press it, it will act as though you had clicked `InitConds Mouse`.

The remaining options can be set from within the program. They are

- LT=`int` sets the linetype. It should be less than 2 and greater than -6.
- SEED=`int` sets the random number generator seed.
- XP=name sets the name of the variable to plot on the x-axis. The default is `T`, the time-variable.
- YP=name sets the name of the variable on the y-axis.
- ZP=name sets the name of the variable on the z-axis (if the plot is 3D.)
- NPLOT=`int` tells XPP how many plots will be in the opening screen.
- XP2=name,YP2=name,ZP2=name tells XPP the variables on the axes of the second curve; XP8 etc are for the 8th plot. Up to 8 total plots can be specified on opening. They will be given different colors.
- AXES={`2,3`} determine whether a 2D or 3D plot will be displayed.
- TOTAL=value sets the total amount of time to integrate the equations (default is 20).
- DT=value sets the time step for the integrator (default is 0.05).
- NJMP=`integer`, NOUT=`integer` tell XPP how frequently to output the solution to the ODE. The default is 1, which means at each integration step. It is also used to specify a the period for maps in the continuation package AUTO.
- T0=value sets the starting time (default is 0).
- TRANS=value tells XPP to integrate until `T=TRANS` and then start plotting solutions (default is 0.)
- NMESH=`integer` sets the mesh size for computing nullclines (default is 40).
- {BANDUP=int, BANDLO=int} sets the upper and lower limits for banded systems which use the banded version of the CVODE integrator.

- METH={ `discrete,euler,modeuler,rungekutta,adams,gear,volterra, backeul, qualrk,stiff,cvode,5dp,83dp,2rb, ymp`} sets the integration method (see below; default is Runge-Kutta.) The latter four are the two Dormand-Prince integrators, the Rosenbrock, and the symplectic integrators.

- DTMIN=value sets the minimum allowable timestep for the Gear integrator.

- DTMAX=value sets the maximum allowable timestep for the Gear integrator

- VMAXPTS=value sets the number of points maintained in for the Volterra integral solver. The default is 4000.

- { JAC_EPS=value, NEWT_TOL=value, NEWT_ITER=value} set parameters for the root finders.

- ATOLER=value sets the absolute tolerance for several of the integrators.

- TOLER=value sets the error tolerance for the Gear, adaptive RK, and stiff integrators. It is the relative tolerance for CVODE and the Dormand-Prince integrators.

- BOUND=value sets the maximum bound any plotted variable can reach in magnitude. If any plottable quantity exceeds this, the integrator will halt with a warning. The program will not stop however (default is 100.)

- DELAY=value sets the maximum delay allowed in the integration (default is 0.)

- PHI=value,THETA=value set the angles for the three-dimensional plots.

- XLO=value,YLO=value,XHI=value,YHI=value set the limits for two-dimensional plots (defaults are 0,-2,20,2 respectively.) Note that for three-dimensional plots, the plot is scaled to a cube with vertices that are ± 1 and this cube is rotated and projected onto the plane so setting these to ± 2 works well for 3D plots.

- XMAX=value, XMIN=value, YMAX=value, YMIN=value, ZMAX=value, ZMIN=value set the scaling for three-d plots.

- OUTPUT=filename sets the filename to which you want to write for “silent” integration. The default is “output.dat”.

- POIMAP={ `section,maxmin`} sets up a Poincare map for either sections of a variable or the extrema.

- POIVAR=name sets the variable name whose section you are interested in finding.

- POIPLN=value is the value of the section; it is a floating point.
- POISGN={ `1, -1, 0` } determines the direction of the section.
- POISTOP=1 means to stop the integration when the section is reached.
- RANGE=1 means that you want to run a range integration (in batch mode).
- RANGEOVER=name, RANGESTEP, RANGELOW, RANGEHIGH, RANGERESET=`Yes,No`, RANGEOLDIC=`Yes,No` all correspond to the entries in the range integration option (see below).
- TOR_PER=value, defined the period for a toroidal phasespace and tellx XPP that there will be some variables on the circle.
- FOLD=name, tells XPP that the variable ¡name¿ is to be considered modulo the period. You can repeat this for many variables.
- STOCH=`1,2` is useful in batch mode and allows one to use the RANGE parameters to compute a mean (1) trajectory or its variance (2). That is, suppose you want to average a simulation over 100 trials. Then set RANGESTEP=500,RANGELOW=0,RANGEHIGH=0, STOCH=1.
- AUTOEVAL={`0,1`} tells XPP whether or not to automatically re-evaluate tables everytime a parameter is changed. The default is to do this. However for random tables, you may want this off. Each table can be flagged individually within XPP.
- AUTO-stuff. The following AUTO-specific variables can also be set: `NTST, NMAX, NPR, EPSU, EPSS, EPSL, DSMIN, DSMAX, DS, PARMIN, PARMAX, NORMMIN, NORMMAX, AUTOXMIN, AUTOXMAX, AUTOYMIN, AUTOYMAX, AUTOVAR`. The last is the variable to plot on the y-axis. The x-axis variable is always the first parameter in the ODE file unless you change it within AUTO.

Named parameter sets. Sometimes, you wnat to prepare a bunch of simulations that use different initial data or parameter values or numerical methods, etc. You can, of course, change these within the program by choosing the desired option and changing it. Or, you can do the simulation and save the results in a ".set" file (see below). Another way to do this is by adding a bunch of parameter sets to the ode file. The format for this is:

```
set name {item1=value1, item2=value2, ..., }
```

Then, you tell XPP to use the named set and it will do all of the things inside the brackets. The items are any parameter name, any variable name, or any of the internal options named above. The named sets are accessed through the `Get par set` menu item in the `File` menu. Here is an example:

```
# test
x'=-a*x+c
par a=1,c=1
set set1 {a=1,c=1,x=0,dt=.25}
set set2 {a=.25,c=0,x=1,dt=.1}
done
```

If you load "set1" then the parameters, initial conditions, and `DeltaT` will be set to the values in the brackets. Choosing "set2" sets them differently. The names can be anything you like.

Network functions. The `special` directive allows you to create dense coupled systems of ODEs and is much faster than using the more general summation operator `sum`. There are two types of convolutions and 2 types of "sparse" coupling functions. The synatx is

```
special zip=conv(type,npts,ncon,wgt,root)
```

This will produce an array, `zip` of `npts` is length defined as:

$$\mathrm{zip}[i] = \sum_{j=-\mathrm{ncon}}^{\mathrm{ncon}} \mathrm{wgt}[j + ncon]\mathrm{root}[i + j]$$

for $i = 0, \ldots, npts - 1$. `root` is the name of a variable and thus, there must be at least `npts-1` variables defined after `root.` The array `wgt` is defined as a table using the `tabular` command and must be of length `2 ncon + 1.` The `type` determines the nature of the convolution at the edges. Type `even` reflects the boundaries, `periodic` makes them periodic, and `0` does not include them in the sum. The object `zip` behaves as a function of ne variable with domain 0 to `npts-1`. Here is an example

```
# neural network
tabular wgt % 25 -12 12 1/25
f(u)=1/(1+exp(-beta*u))
special k=conv(even,51,12,wgt,u0)
u[0..50]'=-u[j]+f(a*k([j])-thr)
par a=4,beta=10,thr=1
done
```

The `sparse` network has the syntax:

```
special zip=sparse(npts,ncon,wgt,index,root)
```

where `wgt` and `index` are tables with at least `npts * ncon` entries. The array `index` returns the indices of the offsets to with which to connect and the array `wgt` is the coupling strength. The return is

```
zip[i] = sum(j=0;j<ncon) w[i*ncon+j]*root[k]
k = index[i*ncon+j]
```

Thus one can make complicated types of couplings. The following is a randomly coupled network with 5 random connections of random strength:

```
# junk2.ode
table w % 255 0 255 .4*ran(1)
table ind % 255 0 255 flr(51*ran(1))
special bob=sparse(51,5,w,ind,v0)
v[0..50]'=-v[j]+f(k*bob([j])-thr-c*delay(v[j],tau))
par k=3,thr=1,beta=1,c=2.5,tau=5
f(u)=1/(1+exp(-beta*u))
done
```

The other two types of networks allow more complicated interactions:

```
special zip=fconv(type,npts,ncon,wgt,root1,root2,f)
```

evaluates as

```
zip[i]=sum(j=-ncon;j=ncon) wgt[ncon+j]*f(root1[i+j],root2[i])
```

and

```
special zip=fsparse(npts,ncon,wgt,index,root1,root2,f)
```

evaluates as

```
zip[i]=sum(j=0;j<ncon) wgt[ncon*i+j]*f(root1[k],root2[i])
k = index[i*ncon+j]
```

They are useful for coupled phase oscillator models.

There are two more such functions which are essentially just matric multiplications.

```
special k=mmult(n,m,w,u)
```

returns a vector `k` of length `m` defined as

$$k(j) = \sum_{i=0}^{n-1} w(i+nj)u(i)$$

The associated functional operator:

```
special k=fmmult(n,m,w,u,v,f)
```

returns

$$k(j) = \sum_{i=0}^{n-1} w(i+nj)f(u(i),v(j)).$$

Active comments. A line that starts with a quote mark `"` is treated differently from the normal comments and is included in a special buffer. This is to

separate out comments that are descriptive of the general file as opposed to line by line comments which when separated from the ODE file have no context. **Furthermore** you can add "actions" associated with these comments. That is you can set XPP parameters like integration method, etc and also parameters and initial data. These comments have the form:

```
" {gca=1.2,gk=0} Set the potassium to zero and turn on the calcium
```

Clicking on `File Prt info` brings up a window with the ODE source code. Clicking on `Action` in this window brings up the active comments. The user does not see {`gca=1.2,gk=0`} but instead sees:

```
* Set the potassium to zero and turn on the calcium
```

with an asterisk to indicate there is n action associated with the line. Clicking on the `*` will set `gca=1.2` and `gk=0`. Thus, you can make nice little tutorials within the ODE file. These are limited to 500 lines.

Finishing up. The last line in the file should be "done" telling the ODE reader that the file is over.

Important shortcut. All of the declarations, `markov, parameter, wiener, table, aux, init, bndry, global, done,` can be abbreviated by their first letter.

Reserved words

You should be aware of the following keywords that should not be used in your ODE files for anything other than their meaning here.

```
sin cos tan atan atan2 sinh cosh tanh
exp delay ln log log10 t pi if then else
asin acos heav sign ceil flr ran abs del\_shft
max min normal besselj bessely erf erfc poisson
arg1 ... arg9  @ $ + - / * ^ ** shift
| > < == >= <= != not \# int sum of i'
```

These are mainly self-explanatory. The nonobvious ones are:

- `atan2(x,y)` is the argument of the complex number $x + iy$.
- `heav(arg1)` the step function, zero if `arg1<0` and 1 otherwise.
- `sign(arg)` which is the sign of the argument (zero has sign 0)
- `ran(arg)` produces a uniformly distributed random number between 0 and `arg`.
- `besselj, bessely` take two arguments, n, x and return respectively, $J_n(x)$ and $Y_n(x)$, the Bessel functions.

- erf(x), erfc(x) are the error function and the complementary function.
- normal(arg1,arg2) produces a normally distributed random number with mean arg1 and variance arg2.
- poisson(arg1) produces an integer which is the expected number of events for the value of arg from a Poisson process.
- max(arg1,arg2) produces the maximum of the two arguments and min is the minimum of them.
- if(<exp1>)then(<exp2>)else(<exp3>) evaluates <exp1> If it is nonzero it evaluates to <exp2> otherwise it is <exp3>. E.g. if(x>1)then(ln(x))else(x-1) will lead to ln(2) if x=2 and -1 if x=0.
- delay(<var>,<exp>) returns variable <var> delayed by the result of evaluating <exp>. In order to use the delay you must inform the program of the maximal possible delay so it can allocate storage. (See the section on the NUMERICS menus.)
- ceil(arg),flr(arg) are the integer parts of<arg> returning the smallest integer greater than and the largest integer less than <arg>.
- t is the current time in the integration of the differential equation.
- pi is π.
- arg1, ..., arg9 are the formal arguments for functions
- int, # concern Volterra equations.
- shift(<var>,<exp>) This operator evaluates the expression <exp> converts it to an integer and then uses this to indirectly address a variable whose address is that of <var> plus the integer value of the expression. This is a way to imitate arrays in XPP. For example if you defined the sequence of 5 variables, u0,u1,u2,u3,u4 one right after another, then shift(u0,2) would return the value of u2.
- del_shft(<var>,<shft>,<delay>). This operator combines the delay and the shift operators and returns the value of the variable <var> shifted by <shft> at the delayed time given by <delay>. It is of limited utility as far as I know, but I needed it for a problem, so here it is.
- sum(<ex1>,<ex2>)of(<ex3>) is a way of summing up things. The expressions <ex1>,<ex1> are evaluated and their integer parts are used as the lower and upper limits of the sum. The index of the sum is i' so that you cannot have double sums since there is only one index. <ex3> is the expression to be summed and will generally involve i'. For example sum(1,10)of(i') will be evaluated to 55. Another example combines the sum with the shift operator. sum(0,4)of(shift(u0,i')) will sum up u0

and the next four variables that were defined after it. An example below shows how this can be used to solve large systems of equations that are densely coupled.

Compatibility. The parser in this distribution is a great improvement over the old style parser. The parser distinguishes between the old style and the new style by whether or not the first line of the file contains a number. If the first line is a number, then the old style parser is used. Otherwise, the new style is used.

3 Command line arguments

There are only (at present) four command line arguments. They are

-xorfix This changes the way rubber-band drawing for zooms and other things is done. If you do not see a box when you zoom in, you should run XPP with this argument.

-convert This allows you to convert old style parser format to the new style which is much more readable. The program creates a file with the same name as the input file but with the extension `.new` appended. It works on on the examples I have tried but it is still in beta testing.

-silent This allows you to run XPP's integrators without using the X-windows stuff. The result of the integration is saved to a file called "output.dat" but this can be changed. The length of integration, methods, Poincare sections, etc, are all specified in either the options file (see section 15) or in the internal options. Note that when you run a range integration in silent mode, if the parameter `RANGERESET` is `yes` (the default) then a new output file will be opened for each integration. Thus, if you range over 50 values, you will get 50 output files named e.g. `output.dat.0`, `output.dat.1`, etc. If you have set `RANGERESET=no`, then only one file is produced.

-allwin tells XPP to make the parameter window, browser, etc immediately visible.

-setfile **filename** loads the setfile, **setfile** after loading up the ODE file.

-newseed uses the machine time to re-seed the random number generator.

-ee makes the TAB and RETURN in dialogs act like those in Windoze. By default, they are reversed.

The only other thing on the command line should be the file name. Thus,

```
xppaut test.ode -xorfix -convert
```

will convert `bob.ode` to the new format and run it with the `xorfix`.

4 Examples

Nothing helps one understand how to use a program better than lots of examples.

Morris-Lecar Equations

The Morris-Lecar equations arise as a simplification of a model for barnacle muscle oscillations. They have the form:

$$C\frac{dV}{dt} = g_L(V_L - V) + g_K w(V_k - V) + g_{Ca} m_\infty(V)(V_{Ca} - V) + I$$
$$\frac{dw}{dt} = \phi\lambda_w(V)(w_\infty(V) - w)$$

where

$$m_\infty(V) = .5(1 + \tanh((V - V_1)/V_2))$$
$$w_\infty(V) = .5(1 + \tanh((V - V_3)/V_4))$$
$$\lambda_w = \cosh((V - V3)/(2V_4))$$

This is a very straightforward model and so the equation file is pretty simple:

```
# The Morris-Lecar equations

# Declare the parameters
p gl=.5,gca=1,gk=2
p vk=-.7,vl=-.5,vca=1
p v1=.01,v2=.145,v3=.1,v4=.15
p i=.2,phi=.333

# Define some functions
minf(v)=.5*(1+tanh((v-v1)/v2))
winf(v)= .5*(1+tanh((v-v3)/v4))
lamw(v)= cosh((v-v3)/(2*v4))

# define the right-hand sides
v'= gl*(vl-v)+gk*w*(vk-v)+gca*minf(v)*(vca-v)+i
w'= phi*lamw(v)*(winf(V)-w)

# some initial conditions -- not necessary but for completeness
v(0)=.05
w(0)=0

# Done!!
d
```

Note that some errors are now caught by the parser. For example, duplicate names and illegal syntax are found.

Suppose you want to keep track of the calcium current as an auxiliary variable. Then, the following file will work

```
# The Morris-Lecar equations

# Declare the parameters
p gl=.5,gca=1,gk=2
p vk=-.7,vl=-.5,vca=1
p v1=.01,v2=.145,v3=.1,v4=.15
p i=.2,phi=.333

# Define some functions
minf(v)=.5*(1+tanh((v-v1)/v2))
winf(v)= .5*(1+tanh((v-v3)/v4))
lamw(v)= cosh((v-v3)/(2*v4))

# define the right-hand sides
v'= gl*(vl-v)+gk*w*(vk-v)-gca*minf(v)*(v-vca)+i
w'= phi*lamw(v)*(winf(v)-w)
#
aux ica=gca*minf(v)*(v-vca)

# some initial conditions -- not necessary but for completeness
v(0)=.05
w(0)=0

# Done!!
d
```

Note that we are wasting computational time since we compute I_{Ca} twice; once as a contribution to the potential change and once as an auxiliary variable. In a FORTRAN or C program, one would compute it as a local variable and use it in both instances. This is the purpose of fixed variables. (For the present problem, the computational overhead is trivial, but for coupled arrays and other things, this can be quite substantial.) The last program uses a fixed variable to reduce the computation:

```
# The Morris-Lecar equations ml1.ode

# Declare the parameters
p gl=.5,gca=1,gk=2
p vk=-.7,vl=-.5,vca=1
p v1=.01,v2=.145,v3=.1,v4=.15
p i=.2,phi=.333
```

```
# Define some functions
minf(v)=.5*(1+tanh((v-v1)/v2))
winf(v)= .5*(1+tanh((v-v3)/v4))
lamw(v)= cosh((v-v3)/(2*v4))

# define the right-hand sides
v'= gl*(vl-v)+gk*w*(vk-v)-icaf+i
w'= phi*lamw(v)*(winf(v)-w)

# where
icaf=gca*minf(v)*(v-vca)

# and
aux ica=icaf

# some initial conditions -- not necessary but for completeness
v(0)=.05
w(0)=0


# Done!!
d
```

The calcium current is computed once instead of twice. This is why "fixed" variables are useful.

NOTE: Since fixed quantities are not "visible" to the user and auxiliary quantities are not "visible" to the internal formula compiler, we can use the same name for the fixed as the auxiliary variable; the `aux` declaration essentially makes it visible to the user with almost no computational overhead. However, it does generate an error message (not fatal) so it is best to make all names unique.

A linear cable equation with boundary conditions

In studying a dendrite, one is often interested in the steady-state voltage distribution. Consider a case where the dendrite is held at $V = V_0$ at $x = 0$ and has a leaky boundary condition at $x = 1$. Then the equations are:

$$\begin{aligned} \lambda^2 \frac{d^2 V}{dx^2} &= V(x) \\ V(0) &= V0 \\ a\frac{dV(1)}{dx} + bV(1) &= 0 \end{aligned}$$

When $a = 0, b \neq 0$ the voltage at $x = 1$ is held at 0. When $a \neq 0, b = 0$ there is no leak from the cable and the conditions are for sealed end. Since this is a second order equation and XPP can only handle first order, we write it as a system of two first order equations in the file which is:

```
# Linear Cable Model cable.ode

# define the 4 parameters, a,b,v0,lambda^2
p a=1,b=0,v0=1,lam2=1
# now do the right-hand sides
v'=vx
vx'=v/lam2

# The initial data
v(0)=1
vx(0)=0

# and finally, boundary conditions
# First we want V(0)-V0=0
b v-v0
#
# We also want aV(1)+bVX(1)=0
b a*v'+b*vx'
# Note that the primes tell XPP to evaluate at the right endpoint
d
```

Note that I have initialized V to be 1 which is the appropriate value to agree with the first boundary condition.

The delayed inhibitory feedback net

A simple way to get oscillatory behavior is to introduce delayed inhibition into a neural network. The equation is:

$$\frac{dx(t)}{dt} = -x(t) + f(ax(t) - bx(t-\tau) + P)$$

where $f(u) = 1/(1 + \exp(-u))$ and a, b, τ are nonnegative parameters. Here p is an input. The XPP file is:

```
# delayed feedback

# declare all the parameters, initializing the delay to 3
p tau=3,b=4.8,a=4,p=-.8
# define the nonlinearity
f(x)=1/(1+exp(-x))
# define the right-hand sides; delaying x by tau
dx/dt = -x + f(a*x-b*delay(x,tau)+p)
x(0)=1
# done
d
```

Try this example after first going into the numerics menu and changing the maximal delay from 0 to say, 10. Glass and Mackey describe a few other delay systems some of which have extremely complex behavior. Try them out.

A population problem with random mutation

This example illustrates the use of Markov variables and is due to Tom Kepler. There are two variables, x_1, x_2 and a Markov state variable, z. The equations are:

$$\begin{aligned} x_1' &= x_1(1 - x_1 - x_2) \\ x_2' &= z(ax_2(1 - x_1 - x_2) + \epsilon x_1) \end{aligned}$$

and z switches from 0 to 1 proportionally to x_1. The transition matrix is 0 everywhere except in the 0 to 1 switch where it is ϵx_1. So z=1 is an absorbing state.

Initial conditions should be $x_1(0) = 1.e-4$ or so, $x_2(0)$ the same (this is just for convenience; we really want $x_2(0) = 0$ and then have it jump discontinuously to $1.e-4$ or so when z makes its transition, but this shouldn't matter that much)

This models the population dynamics of two populations x_1, x_2 in competition with each other. x_2, initially absent, is a mutant of x_1. The mutation rate ϵ should be smaller than one. The relative advantage, a, should be larger than one.

One expects that x_1 grows for a while, eventually z makes its transition, x_2 begins to grow and eventually overtakes x_1.

The equation file for this is

```
# Kepler model kepler.ode

init x1=1.e-4,x2=1.e-4,z=0
p eps=.1,a=1
x1' = x1*(1-x1-x2)
x2'= z*(a*x2*(1-x1-x2)+eps*x1)
# the markov variable and its transition matrix
markov z 2
{0} {eps*x1}
{0} {0}
d
```

Some equations with flags

Tyson describes a model which involves cell growth:

$$\begin{aligned} \frac{du}{dt} &= k_4(v - u)(a + u^2) - k_6 u \\ \frac{dv}{dt} &= k_1 m - k_6 u \\ \frac{dm}{dt} &= bm \end{aligned}$$

This is a normal looking model with exponential growth of the mass. However, if u decreases through 0.2, then the "cell" divides in half. That is the mass

is set to half of its value. Thus, we want to flag the event $u = .2$. The file in the *new format* (no sense using the old format since global variables did not appear in earlier versions of XPP)

```
# tyson.ode
i u=.0075,v=.48,m=1
p k1=.015,k4=200,k6=2,a=.0001,b=.005
u'=  k4*(v-u)*(a+u^2) - k6*u
v'= k1*m - k6*u
m'= b*m
global -1 {u-.2} {m=.5*m}
d
```

Everything is fairly straightforward. When $u-.2$ decreases through zero tha is, u is greater than .2 and then less than .2, the mass is cut in half. Integration using any of the integrators *except ADAMS!* yields a regular limit cycle oscillation.

Another example with discontinuities arises in the study of coupled oscillators. Two phase oscillators x, y travel uniformly around the circle. If x hits 2π it is reset to 0 and adds an amount $r(y)$ to the phase of y. The XPP file is

```
# delta coupled oscillators delta.ode
x'=1
y'=w
global 1 {x-2*pi} {x=0;y=b*r(y)+y}
global 1 {y-2*pi} {y=0;x=b*r(x)+x}
r(x)=sin(x+phi)-sin(phi)
par b=-.25,phi=0,w=1.0
done
```

Note that there are 2 conditions and each creates 2 events.

Large coupled systems

Many times you want to solve large coupled systems of differential equations. Writing the ODE files for these can be a tedious exercise. The array declaration described above simplifies the creation of ODE file for this. For example, suppose that you want to solve:

$$\frac{du_j}{dt} = -u_j + f(a \sum_{i=0}^{n-1} \cos \beta (i-j) u_i)$$

which is a discrete convolution. Suppose that `n=20` so that this is 20 equations. Its not very convenient to type these equations so instead you can create a simple file in which the equations are typed just once:

```
# chain of 20 neurons
param a=.25,beta=.31415926
u[0..19]'=-u[j]+f(a*sum(0,19)of(cos(beta*([j]-i'))*shift(u0,i')))
f(x)=tanh(x)
done
```

The u[0..19] line with an index spanning 0 to 19 tells XPP to repeat this from 0 to 19. Thereafter, the string [j] or some simple arithmetic expressions of j are evaluated and substituted verbatim. Note how I have used the shift operator on u0 to make it act like an array. (Recall that shift(x,n) gives the value of the variable that is defined n after the variable x is defined.

Here is one more example of nearest neighbor coupling in an excitable medium. This is a discretization of a PDE. I will use the analogue of "no flux" boundaries so that the end ODEs will be defined separately.

```
# pulse wave
param a=.1,d=.2,eps=.05,gamma=0,i=0
v0(0)=1
v1(0)=1
f(v)=-v+heav(v-a)
#
v0'=i+f(v0)-w0+d*(v1-v0)
v[1..19]'=i+f(v[j])-w[j]+d*(v[j-1]-2*v[j]+v[j+1])
v20'=i+f(v20)-w20+d*(v19-v20)
#
w[0..20]'=eps*(v[j]-gamma*w[j])
@ meth=modeuler,dt=.1,total=100,xhi=100
done
```

I only need to define v0,v20 separately since w does not diffuse. Note how much typing I saved.

XPP comes with some other examples that I urge you to look at. A Volterra example is shown below.

5 Using xpp

XPP runs from the command line by typing

```
xpp <filename>
```

where <filename> is an ODE file as described above. This argument is optional. If you invoke XPP without the argument, you are asked whether you want to read or create a file. Choosing (r) to read the file prompts you for an ODE file name. You can get a directory by pressing <RETURN>. If you choose to create a new file, you must write a set of commands such as above and then save the file. It will bounce you out if you make any mistakes. It is not the recommended way to do it – use an editor and read in the file. XPP will look at the extension of a file and if it is .dif the program will assume that the model is a difference equation and will treat it as a map. The solution, equilibria, stability and listing of difference equations is treated differently from differential equations. The default plot style is also plotting of points instead of lines as for the continuous time case.

Once you get a file loaded and it is valid, the program and all of the windows will pop up. There is the main window with the graphics and menus. This has several regions on it: the graphics window, the menu window, the command window across the top, the message window at the bottom, and three sliders for binding to parameters. Initial conditions and parameters as well as text labels can be assigned values in the command window for those who don't like the mouse. The message window gives the position of the pointer and "tips" about the various menu items. If you click the mouse in the main graph and hold it down as you move it, the coordinates appear in the message window. The left most region is the menu. Pressing the mouse on a command is the same as typing the hot letter for the command and will execute it. As you move through the menu items, a brief hint about its maning appears in the message window. The pointer is a hand in the menu window. *In addition to the main window, there several other windows that will be created. Due to user demand, these are all instantly iconified so that you must open them up if you want to use them.*

In addition to the main window, there is the initial data window which contains the names of the variables and the current initial data. The delay window is similar containing the initial data for delay equations. The parameter window contains the list of all parameters and their current values. The boundary condition window is similar with the list of boundary conditions given. These 4 windows constitute the main user editable windows. All of the quantities and formulae contained within these can be edited within the program. Clicking on any item in these windows will cause the cursor to appear where it can be edited. If you type the `Enter` key after entering the item, the cursor moves to the next item and the edited item is accepted. You can press the `OK` button also to accept the item and the pointer is then redirected to the main window. Pressing the `Default` key in the Parameter Window will reset the parameters to their values when the program boots up. The `Cancel` button doen't do much of anything yet, but maybe someday if I can think of a use! Pressing the `Tab` key is the same as pressing the `Ok` button. Pressing `Esc` exits without changing.

You can also press the letter `p` in the main window to change parameters or simply click on the parameter command in the main menu. Typing `default` will load all the parameters with their values when the program first fires up. The initial data can also be changed from the command window (see below.)

Another window contains the listing of the differential equations. If the file is a difference equation, the listing shows this. These equations can be scrolled up and down by pressing the mouse on the `<Up> <Down>` buttons or using the cursor keys. Resizing lets you see more and closing iconifies the window. Once you are in XPP you cannot alter the differential equations. Thus , to solve a different problem you must exit and start again. I hope to change this in the future. All of these windows can be iconified with no effect on the performance of the program.

The other major window contains listings of the numerical values of the solutions to the differential equations. This browser window allows you to post-process data and save and load numbers to the disk. The first few lines contain a variety of buttons. The next line has hints about the various buttons that

are given as you move the mouse over the buttons. The names of the variables, time, and auxilliary variabls are next. The the numbers themselves are given. Pressing the cursor keys, the HOME, END, PgUp,PgDn keys scrolls you through tthe data either up and down or left and right. Resizing the window lets you see more data. There are many other keys and you can click on the buttons or use the keyboard if the mouse pointer is in the Browser. You can load or save data files by choosing the Load and Save keys. You will be asked for a filename which you should enter. Choosing Get will load the top row of numbers into the initial data. Find will find the data row for which the specified variable is closest to the specified value. Replace allows you to replace a column by some formula involving the column itself and all other columns. You are asked for the column to replace. Type in one of the names across the top row. Then you are asked for the formula. Type it in and the entire column will be replaced. For example if the names of your variables are X and Y and you choose X as the column to replace and type

```
X^2 + T*Y
```

then the column X will be replaced by the function at each row. If the first character in the formula is the & symbol, an integral of the formula will be placed in the specified column. If the formula is anything of the form :

```
@<variable>
```

the numerical derivative of the <variable> will be computed ad placed in the column. If the formula has the form:

```
lo:hi
```

the the column will be replaced by an arithmetic series starting at lo and ending at hi. If the formula has the form:

```
lo;inc
```

the the column will be replaced by an arithmetic series starting at lo incrementing by inc.

Choosing Unreplace will restore the most recent column. Each time you integrate the equations or solve a boundary value problem or look at a range of equilibria, the numbers are replaced by the most recent. You must save the data to a file to use it again. Pressing First and last will mark the data so that you can save a portion or restore a portion. Home takes you to the beginning and End to the end of the list.

The Table key allows you to save a column as a "table" file which can be used in XPP as a function.

These are really cool and I stole the idea from Madonna, a Mac ODE solver. Click on one of them where it says Parameter and fill it in. These bind the slider to a particular parameter. You set the low and high values of the slider and the current value. Click OK to accept. Change them by clicking on the name

window. Then move the mouse to the slider and pressing it, move it around. When done, you have set the parameter to the indicated value.

Other windows are opened on occasion and can remain open throughout the session. The usual methods for resizing windows works on the graphics and listing and browser windows.

When there are multiple graphics windows, only one is active at a given time and only one can be drawn to. The window is made active by clicking the left button when inside the window. A little square appears in the upper left corner of the window to indicate its activity. The variables on the axes are given in the title of the window and are Z-Axis vs Y-Axis vs X-Axis.

Two other windows appear permanently, when needed: the Equilibrium window that gives values and stability of the most recently computed equilibrium and the AUTO window for bifurcation analysis.

The main commands are on the first menu and the numerics menu. Communication to the program is via the keyboard and the mouse and through parameter boxes that are displayed. Almost all commands have keyboard shortcuts so that if you know the original DOS version, you can use the X version with little help. When you are prompted for floating point numbers at the command line, then you can insert either numbers or mathematical expressions provided the first character is the `%` symbol. Thus if you want to input `sin(1.5)` as a floating point number, you would write

```
%sin(1.5)
```

at the command line. You can enter such expressions in the parameter and initial data windows and these are converted to numbers.

6 The main commands

All commands can be invoked by typing the hot key for that command (capitalized on the menu) or clicking on the menu with the mouse. Usually most commands can be aborted by pressing the `Esc` key. Once one of these is chosen, the program begins to calculate and draw the trajectories. If you want to stop prematurely, press the `Esc` key and the integration will stop.

(I)nitial conds This invokes a list of options for integrating the differential equations. The choices are:

- **(R)ange** This lets you integrate multiple times with the results shown in the graphics window. Pressing this option produces a new window with several boxes to fill in. First choose the quantity you want to range over. It can be a parameter or a variable. The integrator will be called and this quantity will be changed at the beginning of each integration. Then choose the starting and ending value and the number of steps. The option Reset storage only stores the last integration. If you choose not to reset, each integration is appended

to storage. Most likely, storage will be exceeded and the integration will overwrite or stop. The option to use last initial conditions will automatically use the final result of the previous integration as initial dat for the next integration. Otherwise, the current ICs will be used at each step (except of course for the variable through which you are ranging.) If you choose `Yes` in the `Movie` item, then after each integration, XPP will take a snapshot of the picture. You can then replay this series of snapshots back using the Kinescope. When you are happy with the parameters, simply press the OK button. Otherwise, press the Cancel button to abort. Assuming that you have accepted, the program will compute the trajectories and plot them storing none of them or all of them. If you press `Esc` it will abort the current trajectory and move on to the next. Pressing the `/` key will abort the whole process.

(2)par range is similar to range integration but allows you to range over two items. The `Crv(1) Array(2)` item determines how the range is done. If you choose `Crv` then the two paramaters are varied in concert, $[a(i), b(i)]$ for $i = 0, \ldots, N$. The more useful `Array` varies them independently as $[a(i), b(j)]$ for $i = 0, \ldots, N$ and $j = 0, \ldots, M$.

(L)ast This uses the end result of the most recent integration as the starting point of the curret integration.

(O)ld This uses the most recent initial data as the current initial data. It is essentially the same as Go.

(G)o which uses the initial data in the IC window and the current numerics parameters to solve the equation. The output is drawn in the current selected graphics window and the data are saved for later use. The solution continues until either the user aborts by pressing `Esc`, the integration is complete, or storage runs out.

(M)ouse allows you to specify the values with the mouse. Click at the desired spot.

(S)hift This is like Last except that the stating time is shifted to the current integration time. This is irrelevant for autonomous systems but is useful for nonautonomous ODEs.

(N)ew This prompts you at the command line for each initial condition. Press `Return` to accept the value presented.

s(H)oot allows you to use initial data that was produced when you last searched for an equilibrium. When a rest state has a single positive or negative eigenvalue, then XPP will ask if you want to approximate the invariant manifold. If you choose `yes` to this, then the initial data that were used to compute the trajectories are remembered. Thus, when you choose this option, you will be asked for a number 1-4. This number is the order in which the invariant trajectories were computed. Note if the invariant set is a stable manifold, then you should integrate backwards in time.

(F)ile prompts you for a file name which has the initial data as a sequence of numerical values.

form(U)la allows you to set all the initial data as a formula. This is good for systems that represent chains of many ODEs. When prompted for the variable, type in `u[2..10]` for example to set the variables `u2,u3, ..., u10` and then put in a formula using the index `[j]`. Note you must use `[j]` and not `j` by itself. For example `sin([j]*2*pi/10)`. Repeat this for different variables hitting enter twice to begin the integration.

M(I)ce allows you to choose multiple points with the mouse. Click Esc when done.

(D)AE guess lets you choose a guess for the algebraic variables of the DAE.

(B)ackward is the same as "Go" but the integration is run backwards in time.

(C)ontinue This allows you to continue integrating appending the data to the current curve. Type in the new ending time.

(N)ullclines This option allows you to draw the nullclines of systems. They are most useful for two-dimensional models, but XPP lets you draw them for any model. The constraints are the same as in the direction fields option above. The menu has 4 items.

(N)ew draws a new set of nullclines.

(R)estore restores the most recently computed set.

(A)uto turns on a flag that makes XPP redraw them every time it is necessary because some other window obscured them.

(M)anual turns this flag off so that you must restore them manually. The X-axis nullcline is blue and the Y-axis nullcline is red.

(F)reeze allows you to freeze and play back multiple nullclines

(F)reeze freezes the current nullclines

(D)elete all deletes all frozen nullclines

(R)ange lets you vary a parameter through some range, computes the nullclines and stores them

(A)nimate redraws all the frozen nullclines erasing the screen between each one. The user specifies a delay between drawing.

(S)ave saves the nullclines into a file. They can then be plotted using other software. **NOTE:** The way that XPP computes nullclines (by computing zero contours of a two-variable function) means that the nullclines are composed of a series of small line segments. This means that if you try to plot them as a continuous curve, your plotting program will produce garbage. Thus, you should plot them as points

and not draw line segments between them. The data file produced has 4 columns. The first two are the "x" nullcline and the second two are the "y" nullcline. Data is as follows:

```
xnx1 xny1 ynx1 yny1
xnx2 xny2 ynx2 yny2
...
```

There are an even number of entries. The "x" nullcline consists of segments `(xnx1,xny1),(xnx2,xny2)` between pairs of points. Similarly for the "y" nullcline.

(D)irection Field/Flow This option is best used for two-dimensional systems however, it can be applied to any system. The current graphics view must be a two-d plot in which both variables are different and neither is the time variable, T. There are five items.

(D)irection fields Choosing the direction field option will prompt you for a grid size. The two dimensional plane is broken into a grid of the size specified and lines are drawn at each point specifying the direction of the flow at that point. The length of the line gives the magnitude. If the system is more than two-dimensional, the other variables will be held at the values in the initial conditions window.

(F)low Choosing the flow, you will be prompted for a grid size and trajectories started at each point on the grid will be integrated according to the numerical parameters. Any given trajectory can be aborted by pressing `Esc` and the whole process stopped by pressing `/`. The remaining variables if in more than two-dimensions are initialized with the values in the IC window.

(N)o Dir Field turns off redrawing of direction fields when you click on (Redraw). Erasing the screen automatically turns this off.

(C)olorize This draws a grid of filled rectangles on the screen whose color is coded by the velocity or some other quantity. (See the Numerics Colorize menu item).

(S)caled Dir. Fld is the same as (D)irection field but the lengths are all scaled to 1 so only directional information is given.

(W)indow allows you to rewindow the current graph. Pressing this presents another menu with the choices:

(W)indow A parameter box pops up prompting you for the values. Press OK or CANCEL when done.

(Z)oom in Use the mouse to expand a region by clicking, dragging and releasing. The view in the rectangle will be expanded to the whole window.

Zoom (O)ut As above but the whole window will be shrunk into the rectangle.

(F)it The most common command will automatically fit the window so the entire curve is contained within it. For three-D stuff the window data will be scaled to fit into a cube and the cube scaled to fit in the window. Use this often.

(NOTE: On some displays, no rubber boxes are drawn for the zooming operations. If this occurs, start XPP with the `-xorfix` command line flag.)

ph(A)se space XPP allows for periodic domains so that you can solve equations on a torus or cylinder. You will be prompted to make (A)ll variables periodic, (N)o variables periodic or (C)hoose the ones you want. You will be asked for the period which is the same for all periodic variables (if they must be different, rescale them) Choose them by clicking the appropriate names from the list presented to you. An `X` will appear next to the selected ones. Clicking toggles the `X`. Type `Esc` when done or CANCEL or DONE. XPP mods your variables by this period and is smart enough when plotting to not join the two ends.

(K)inescope This allows you to capture the bitmap of the active window and play it back. Another menu pops up with the choices:

(C)apture which takes a snapshot of the currently active window

(R)eset which deletes all the snapshots

(P)layback which cycles thru the pictures each time you click the left mouse button and stops if you click the middle.

(A)utoplay continuously plays back snapshots. You tell it how many cycles and how much time between frames in milliseconds.

(S)ave Save the frames in either ppm or gif format

(M)ake anigif Create an animated gif from the frames. The file is always called `anim.gif` and can be played with Netscape, Explorer, xanim, etc.

(G)raphic stuff This induces a popup menu with several choices.

(A)dd curve This lets you add another curve to the picture. A parameter box will appear aking you for the variables on each axis, a color, and line type (1 is solid, 0 is a point, and negative integers are small circles of increasing radii.) All subsequent integrations and restorations will include the new graph. Up to 10 per window are allowed.

(D)elete last Will remove most recent curve from the added list.

(R)emove all Deletes all curves but the first.

(E)dit curve You will be asked for th curve to edit. The first is 0, the second 1, etc. You will get a parameter box like the add curve option.

(P)ostscript This will ask you for a file name and write a postscript representation of the current window. Nullclines, text, and all graphs will be plotted. You will be asked for Black and White or Color. Color tries to match the color on the screen. Black and white will use a variety of dashed curves for the plots. The Land/Port option lets you draw either in Landscape (default) or Portrait style. Note that Portrait is rather distorted and is created in for those who cannot rotate their postscript plots. Font size sets the size of the fonts on the axes.

(F)reeze This will create a permanent curve in the window. Usually, when you reintegrate the equations or load in some new data, the current curve will be replace by the new data. Freeze prevents this. Up to 26 curves can be frozen.

- **(F)reeze** This freezes the current curve 0 for the current plotting window. It will not be plotted in other windows. If you change the axes from 2 to 3 dimensions and it was frozen as a 2D curve (and *vice versa*) then it will also not be plotted. It is better to create another window to work in 3 dimensions so this is avoided. A parameter box pops up that asks you for the color (linetype) as well as the key name and the curve name. The curve name is for easy reference and should be a few characters. The key name is what will be printed on the graph if a key is present.
- **(D)elete** This gives you a choice of available curves to delete.
- **(E)dit** This lets you edit a named curve; the key, name, and linetype can be altered.
- **(R)emove all** This gets rid of all of the frozen curves in the current window.
- **(K)ey** This turns the key on or off. If you turn it on, then you can position it with the mouse on the graph. The key consists of a line followed by some text describing the line. Only about 15 characters are permitted.
- **(B)if.diag** will prompt you for a filename and then using the current view, draw the diagram. The file must be of the same format as is produced by the `Write pts` option in the AUTO menus. (see AUTO below.) The diagram is colored according to whether the points are stable/unstable fixed points or periodics. The diagram is "frozen" and there can only be one diagram at a time.
- **(C)lr. BD** clears out the current bifurcation diagram.
- **(O)n freeze** Toggles a flag that automatically freezes the curves as you integrate them.

a(X)es opts This puts up a window which allows you to tell XPP where you want the axes to be drawn, whether you want them, and what

fontsize to make the PostScript axes labels.

exp(O)rt This lets you save the points that are currently plotted on the screen in XY format. Thus if you have a phaseplane on the screen, only the X and Y values are saved. This makes it compatible with porgrams like XMGR which assume X Y1 Y2 ... data. If you have several traces on the screen at once, it saves the X values of the first trace and the Y values of the first and all subsequent traces.

(C)olormap This lets you choose a different color map from the default. There are a bunch of them; try them all and pick your favorite.

n(U)merics This is so important that a section is devoted to it. See below.

(F)ile This brings up a menu with several options. Type `Esc` to abort.

- **(P)rt info** Brings up a window with the source code for the ODE file. If you click on `Action` it brings up the active comments so you can make little tutorials.
- **(W)rite set** This creates a file with all of the info about the current numerics, etc as well as all of the currently highlighted graphics window. It is readable by the user. It in some sense saves the current state of XPP and can be read in later.
- **(R)ead set** This reads a set that you have previously written. The files are very tightly connected to the current ODE file so you should not load a saved file from one equation for a different problem.
- **(A)uto** This brings up the AUTO window if you have installed AUTO. See below for a description of this.
- **(C)alculator** This pops up a little window. Type formulae in the command line involving your variables and the results are displayed in the popup. Click on Quit or type `Esc` to exit.
- **(E)dit** You can edit the equations from within XPP. *Note that XPP is capable of understanding right-hand sides of up to 256 characters. However, the RHS editor will not accept anything longer than about 72 characters.* This menu item presents a list of four options:
  - **(R)HS** Edit the right-hand sides of the ODEs IDEs, and auxiliary variables. If you are happy with the editing, then type `TAB` or click on `OK.` The program will parse the new equations and if they are syntactically correct, alter the corresponding equation. If there is an error, the you will be told of the offending right-hand-side and that will not be changed.
  - **(F)unctions** This lets you alter any user-defined functions. It is otherwise the same as the above.
  - **(S)ave as** This creates an "ODE" file based on the current parameter values, functions, and right-hand sides. You will be asked for a filename.

(L)oad DLL invokes the dynamic linker. You can load in complicated RHS's that would be awkward to create using XPP's simple language.

(S)ave info This is like `(P)rt info` but saves the info to a file. It is human readable.

(B)ell on/off This toggles the stupid noisy bell on and off.

C-(H)ints spews out stuff to the terminal that represents a skeletal C program for the right-hand sides. Presumably, you could use this to create faster code by replacing the file `myrhs.c` with your compiled version. Hah! I don't khow why this is even here – Good luck!.

(Q)uit This exits XPP first asking if you are sure.

(T)ranspose This is not a very good place to put this but I stuck it here just to get it into the program. The point of this routine is to allow one to transpose chunks of the output. For example, if you are solving the discretization of some spatial problem and find a steady state, there is no way to plot the steady state as a function of the index of the discrete system. This routine lets you do that. The idea is to take something that looks like:

```
t1  x11  x21  x31 ... xm1
t2  x12  x22  x32 ... xm2
...
tn  x1n  x2n  x3n ... xmn
```

and transpose some subset of it. You are prompted for 6 items. They are the name of the first column you want to index, the number of columns (`ncols` and amount you want to skip across columns, `colskip` (so that `colskip = 2` would be every other column. You must also provide the starting row `j1`, the number of rows, `nrows` and the row skip, `rowskip.` the The storage array is temporarily replaced by a new array that has `M=ncols` rows and `nrows+1` columns (since the data is transposed, the rows and columns are as well; confusing ain't it). The form of the array is:

```
1  x(i1,j1) x(i1,j2) x(i1,j3) ...
2  x(i2,j1) x(i2,j2) x(i2,j3) ...
...
M  x(iM,j1) x(iM,j2) x(iM,j3) ...
```

where `i2=i1+colskip, i3=i1+2*colskip, ...` and `i1` is the index corresponding to the name of the first column you provide. Similarly, `j2=j1+rowskip, ...`. As a brief example, suppose that you solve a system of equations of the form:

$$x_j' = f(x_{j-1}, x_j, x_{j+1}, I_j)$$

where $j = 1, \ldots, 20$. Click on transpose and choose `x1` as the first column, `colskip=1, ncols=20` and say `row1=350, nrows=1,rowskip=1`

then a new array will be produced. The first column is the index from 1 to 20 and the second is `xj(350)` where 350 is the index and not the actual value of time. By plotting the second column versus the first you get a "spatial profile."

t(I)ps This toggles the tips on and off that appear in the message line.

(G)et par set This loads one of the parameter sets that you have defined in the ODE file.

(P)arameters Type the name of a parameter to change and enter its value. Repeat for more parameters. Hit `Enter` a few times to exit. Type `default` to get back the values when you started XPP. Use this if you don't want to mess with the mouse.

(E)rase erases the contents of the active window, redraws the axes, deletes all text in the window, and sets the redraw flag to Manual.

(M)ake window The option allows you to create and destroy graphics windows. There are several choices.

(C)reate makes a copy of the currently active window and makes itself active. You can change the graphs in this window without affecting the other windows.

(K)ill all Removes all but the main graphics window.

(D)estroy This destroys the currently active window. The main window cannot be destroyed.

(B)ottom puts the active window on the bottom.

(A)uto turns on a flag so that the window will automatically be redrawn when needed.

(M)anual turns off the flag and the user must restore the picture manually.

(S)imPlot on/off lets you plot the solution in all active windows while the simulation is running. This slows you down quite a bit.

After a window is created, you can use the mouse to find the coordinates by pressing and moving in the window. The coordinates are given near the top of the window.

(T)ext, etc allows you to write text to the display in a variety of sizes and in two different fonts. You can also add other symbols to your graph.

Text This prompts you for the text you want to add. Then you are asked for the size; there are five choices (0-5): 0-8pt, 1-12pt, 2-14pt, 3-18pt, 4-24pt. Text also has several escape sequences:

- \1 – switches to Greek font
- \0 – switches to Roman font

- \s – subscript
- \S – superscript
- \n – neither sub nor superscript
- \{expr} – evaluate the expression in the braces before rendering.

Note that not all X-servers will have these fonts, but the postscript file will still draw them. Finally, place the text with the mouse.

Arrow This lets you draw an arrow-head to indicate a direction on a trajectory. You will be prompted for the size, which should be some positive number, usually less than 1. Then you must move the the mouse and select a direction and starting point. Click on the starting point and holding the mouse button down, drag the mouse to indicate the direction of the arrow-head. Then release the mouse-button and the arrow will be drawn.

Pointer This is like an arrow, but draws the stem as well as the arrow head. It can be used to point to important features of your graph. The prompts are like those for `Arrow.`

Marker This lets you draw little markers, such as triangles, squares, etc on the picture. When prompted to position the marker with the mouse, you can over-ride the mouse and manually type in coordinates if you hit the (Tab) key.

Edit This lets you edit the text, arrows, and pointers in one of three ways:

Move lets you move the object to another location without changing any of its properties. Choose the object with the mouse by clicking near it. You will then be prompted as to whether you want to move the item that XPP selected. If you answer `yes` use the mouse to reposition it.

Change lets you change the properties: for text, the text itself, size, and font can be change; for arrows and pointers, only the size of the arrow head can be changed. As above, select the object with the mouse and then edit the properties.

Delete deletes the object that you select with the mouse.

(D)elete All Deletes all the objects in the current window.

marker(S) This is similar to the Marker command, but allows you to automatically mark a number of points along a computed trajectory. You use the data browser to move the desired starting point of the list to the top line of the browser. Then click on the (Text) (markerS) command and choose a size and color. Then tell XPP how many markers and how many browser lines to jump between markers. (Thus, 10 would put a marker at every 10th data point)..

(S)ing pts This allows you to calculate equilibria for a discrete or continuous system. The program also attempts to determine stability for delay-differential equations (see below in the numerics section.) There are three options.

(G)o begins the calculation using the values in the initial data box as a first guess. Newton's method is applied. If a value is found XPP tries to find the eigenvalues and asks you if you want them printed out. If so, they are written to the console. Then if there is a single real positive or real negative eigenvalue, the program asks you if you want the unstable or stable manifolds to be plotted. Answer yes if so and they will be approximated. The calculation will continue until either a variable goes out of bounds or you press `Esc`. If `Esc` is pressed, the other branch is computed. (Unstable manifolds are yellow and computed first followed by the stable manifolds in color turquoise.) The program continues to find any other invariant sets until it has gotten them all. These are not stored, however, the initial data needed to create them are and can be accessed with the `Initial Conds sHoot` command. Once an equilibrium is computed a window appears with info on the value of the point and its stability. The top of the window tells you the number of complex eigenvalues with positive,`(c+)`, negative `(c-)`, zero `(im)` real parts and the number of real positive `(r+)` and real negative `(r-)` eigenvalues. If the equation is a difference equation, then the symbols correspond the numbers of real or complex eigenvalues outside `(+)` the unit circle or inside `(-)` This window remains and can be iconified.

(M)ouse This is as above but you can specify the initial guess by clicking the mouse. Only the two variables in the two-D window will reflect the mouse values. This is most useful for 2D systems.

(R)ange This allows you to find a set of equilibria over a range of parameters. A parameter box will prompt you for the parameter, starting and ending values, number of steps. Additionally, two other items are requested. Column for stability will record the stability of the equilibrium point in the specified column (Use column number greater than 1). If you elect to shoot at each, the invariant manifolds will be drawn for each equilibrium computed. The stability can be read as a decimal number of the form `u.s` where `s` is the number of stable and `u` the number of unstable eigenvalues. So `2.03` means 3 eigenvalues with negative real parts (or in the unit circle) and 2 with positive real parts (outside the unit circle.) For delay equations, if a root is found, its real part is included in this column rather than the stability summary since there are infinitely many possible eigenvalues. The result of a range calculation is saved in the data array and replaces what ever was there. The value of the parameter is in the time column, the equilibria in the remaining columns and the stability info in whatever column you have specified. `Esc` aborts one step and `/`

aborts the whole procedure. As with the initial data/range option, you can also make a movie. This is useful mainly for systems where invariant sets are to be computed.

(V)iew axes This selects one of different types of graphs: a 2D box or a 3D Box; brings up a threed window; or lets you create animations of your simulation. If you select the 2D curve, you will be asked for limits as in the window command as well as the variables to place on the axes and the labels for the axes. 3D is more complicated. You will be asked for the 3 variables for the 3 axes, their max and min values and 4 more numbers, `XLO`, etc. XPP first scales the data to fit into a cube with corners (-1,-1,-1) and (1,1,1). Rotation of this cube is performed and then projected into the two-D window. `XLO`, etc define the scales of this projection and are thus unrelated to the values of your data. You are also asked for labels of the axes. Use the `(F)it` option if you don't know whats going on. (A)rray plots introduce a new window that lets the user plot many variables at once as a function of time with color coded values. The point is to let one plot, e.g., an array of voltages, `V1, V2, ..., VN` across the horizontal as time varies in the vertical dimension. For example, suppose you have discretized some PDE and want to see the evolution in space and time of the variables. Then use this plotting option. You will be prompted for the first column name of the "array", then number of columns, the first row, then number of rows, and the number of rows to skip. For example, if the discretized PDE variables are `u0,u1, ... ,u50`, then type in `u0` for the first element and `51` for the number of columns. If you want rows 200 through 800 only every 4th time unit, you would put 200 for the first row, 201 as the number of rows, and 4 as the skip value. You can also set the column skip as well. This is useful if you have defined a series of variables with the array blocks. If the array block is for a two-component system, then plot every 2, so you would put 2 in this entry. The `Print` button asks you for a file name and top and bottom labels and a render style. The render styles are:

-1 Grey scale

0 Blue-red

1 Red-Yellow-Green-Blue-Violet

2 Like 1 but periodic

The `Style` button does nothing yet. The `Edit` button lets you change ranges and arrays to plot. The `Redraw` button is obvious.

Since the animation option requires learning lots of new stuff, see section 11 for a description of the animation language and what you can do with it.

(X)i vs t This chooses a certain 2D view and prompts you for the variable name. The window is automatically fitted and the data plotted. It is a shortcut to choosing a view and windowing it.

(R)estore redraws the most recent data in the browser in accordance with the graphics parameters of the active window.

(3)d params This lets you choose rotations of the axes and perspective planes. Play with this to see. You must be have a 3D view in the active graph to use this.

There is another selection: `Movie`. If you choose `Yes` for this, then after you click `Ok`, you will be prompted for some additional parameters. There are two angles you can vary, `theta` and `phi`. Choose one, give an initial value, an increment, and the number of rotations you want to perform. XPP will then use the `Kinescope` to take successive snapshots of the screen after performing each rotation. You can then play these back from the Kinescope or save them as an animated gif.

(B)ndry val This solves boundary value problems by numerical shooting. There are 4 choices.

(S)how This shows the successive results of the shooting and erases the screen at the end and redraws the last solution. The program uses the currently selected numerical integration method, the current starting point, `T0` as the left end time and `T0+TEND` as the right end. Thus, if the interval of interest is `(2.5,6)` then set `T0=2.5` and `TEND=3.5` in the numerics menu.

(N)o show This is as above but will not show successive solutions.

(R)ange This allows you to range over a parameter keeping starting or ending values of each of the variables. A window will appear asking you for the parameter, the start, end, and steps. You will also be asked if you want to cycle color which means that the results of each successful solution to the BVP will appear in different colors. Finally the box labeled`side` tells the program whether to save the initial `(0)` or final`(1)` values of the solution. As the program progresses, you will see the current parameter in the info window under the main screen. You can abort the current step by pressing `Esc` and the whole process by pressing `/`. As in the `Initialconds Range` option, you can also choose `Movie`. Then, as before, after each solution is computed, a snapshot is take. Thus, you can playback the solutions as a function of the range parameter.

(P)eriodic Periodic boundary conditions can be solved thru the usual methods, but one then must write an addition equation for the frequency parameter. This option eliminates that need so that a 2-D autonomous system need not be suspended into a 3D one. You will be asked for the name of the adjustable parameter for frequency. You will also be asked for the section variable and section. This is an additional condition that must be satisfied, namely, $x(0) = x_0$ where x is the section variable and x_0 is the section. Type `yes` if you want the progress shown.

7 Numerical parameters

When you click on the `nUmerics` command in the main menu, a new list appears. This is the numerics menu and allows you to set all of the numerical parameters as well as some post-processing. Some of these may not yet be implemented. Press `Esc` or click on the `exit` to get the main menu back.

The items on this menu are:

(T)otal This is the amount of time to integrate. It is called `TEND` in the documentation. If it is negative then it will be made positive and no data will be stored. Thus you can integrate for very long periods of time without being told that the storage is full.

(S)tart time This is the initial time `T0` For autonomous systems it is usually irrelevant.

tRansient The program will integrate silently for this amount of time before plotting output. It is used to get rid of transients.

(D)t This is the step size used by the fixed step integrators and the output step for Gear,CVODE, Quality RK, Rosen,and Stiff algorithms. It is positive or negative depending on the direction of integration.

n(C)line ctrl will prompt you for the grid size for computing nullclines.

s(I)ng pt ctrl This prompts you for errors and epsilons for eigenvalues and equilibria calculations as well as the maximum iterates. If you have global flags, then you will be asked for `smin` as well.

n(O)ut This sets the number of integration steps to perform between output to storage. Thus, if you output every 10 steps with a `Dt` of .05, XPP will yield output at times that are $10 * .05 = .5$ timesteps apart. The advantage of this is that lengthier records of data can be made without losing accuracy of the integrator. This parameter is also used in the continuation of fixed point for maps in AUTO. If this parameter is $n > 1$, then in AUTO, the fixed point of $F^(n)(x)$ is found where F is the right-hand side of the equations. This allows AUTO to continue periodic points of maps.

(B)ounds This sets a global bound on the integrator. If any variable exceeds this value in magnitude, a message appears and the integration stops.

(M)ethod allows you to choose the methods of integration. DoPri5, DoPri83, Gear, CVODE, Quality RK, Rosen,and Stiff are adaptive. The first two are the Dormand-Prince integrators and are the most modern of the group. Gear,CVODE, Rosen, and Stiff are the best to use for stiff problems. If you choose the adaptive methods, you will be asked for error tolerance, minimum step, and maximum step size. The discrete method should be used for difference equations. If you choose CVODE/Rosen, you can choose to

use the banded version of it. You should set the upper and lower bandwidths. Note that this is recommended for stiff PDEs only and should be used in conjunction with the block arrays. You can get huge speed up in the integration. I have gotten 100 fold on some problems. If the method is adaptive, the output `NOUT` is set to 1. Also the adaptive methods generate several possible error messages that you may have to respond to. These suggest how to fix the error. Markov processes are ignored by GEAR. Backward Euler prompts you for a tolerance and the maximum number of iterates for each step. The Volterra method, described below, prompts for a tolerance, maximum iterates, and a "memory size." You will also be asked if you want the convolution kernels to be re-evaluated after any parameter is changed in either the range integration or through a manual change in parameters. If this flag is 1 then the kernels will be re-computed. The default is to not recompute them. Memory size determines how far back to save the results for the integrator. If this is small, there is a big speed-up in the integration, but you could be neglecting important terms. The symplectic integrator should only be used for systems of the form:

$$\begin{aligned}
x_1' &= v_1 \\
v_1' &= F_1(x_1,\ldots,x_n) \\
x_2' &= v_2 \\
v_2' &= F_2(x_1,\ldots,x_n) \\
\vdots &= \vdots \\
x_n' &= v_n \\
v_n' &= F_n(x_1,\ldots,x_n)
\end{aligned}$$

where $F_j = \partial_{x_j} V(x_1,\ldots,x_n)$. That is, it is for frictionless mechanical problems. The equations must be written in the above order as well or it won't work. Symplectic integrators preserve a discrete analogue of the energy so that unlike other integrators, they preserve the invariants of the flow.

d(E)lay This sets the upper bound for the maximum delay and three other parameters that have to do with stability of delay equations. If in your delay equations, the delay exceeds this, a message will appear and the integration will stop. Each time this is changed all previous delay data is destroyed and you must begin your integration anew. Thus, it should be the first thing you set when solving a delay equation. Since the storage depends on the size of `Dt` when you change this, then the delay storage will also be destroyed. Delay equations require data for $t < t_0$ so that you should edit the `Delay ICs` to achieve this. Use the fixed step integrators for this, althought, adaptive sometimes will work. In addition to the maximal delay, you will be asked for `real part` and `im part`. When the program looks at stability of delay equations, it considers a rectangular region defined by the four points in the complex plane. It also attempts

to find one root (usually the one with the most positive real part. These two quatities provide a “guess” for the root. Once a root is found, these quantities are replaced by that root. So, if you want to find a root that is different, then change the initial guess.(See the section on numerical methods.) Finally, you will be asked for `DelayGrid` which tells the program how many steps to take on each side of the contour to determine stability. Basically, the larger this parameter is the more accurate the stability determination.

(C)olor code If you have a color system, XPP can code the output according to the magnitude of the velocity or the magnitude of another variable. A choice pops up for these two options or for turning off the color. This overrides any color on any other curves in your picture. Once you choose to color code, you are asked to either choose max and min values or have XPP do it for you via optimize. The latter will compute the max and min and set the scales accordingly.

(P)oincare map This sets up parameters for Poincare sections. A choice of four items will appear: the `Max/Min` option the `Poincare section` option, the `Periodic` option, and the option to turn `off` all maps. A parameter box will pop up and you should type in the parameters. They are the variable to check and the section and the direction. That is, a point will be recorded if the variable crosses the section such that it is either positive going to negative or vice versa according to the direction parameter. If the direction is set to zero, the, the point will be recorded from either direction. The flag `Stop on Section` instructs XPP to halt when the section is crossed. Note that automatic interpolation is done. If the section variable is `Time, T` and the section is say `T1`, then each time `T=0 modulo T1` the point is recorded. This is useful for periodically driven systems. If you have opted for the `Max/Min` option, then the section is irrelevant and the point will be recorded when a local maximum (if the direction is 1) minimum (direction=-1) or both (direction=0) of the variable is encountered. The `Periodic` option does the following. When ever the specified variable hits the section, the time of the hit is recorded and the previous time is subtracted from the current time and recorded in the time column. This leads to a list of intervals between hits. If you create an auxiliary variable that is a complicated function of the other variables, you can use this for the section thus allowing you to have sections which are not the coordinate axes.

R(U)elle plot This allows you to retard any of the axes by an integral number of steps. This is useful for chaotic orbits and delayed systems. Choosing a number for any of the axes will result in the variable associated with that axis being delayed by the number of steps inputted. Thus if you plot X vs X then of course you will get a diagonal line, but if you make the Y-axis delayed by say 50 and the output is every .1 timesteps, then the plot will be X(t-5) vs X(t). This does not appear during integration of

the equations and is available only after a computation. You set it up and then click Restore from the main menu.

stoc(H)astic This brings up a series of items that allow you to compute many trajectories and find their mean and variance. It also contains commands for post-simulation data analysis. It is most useful when used with systems that are either Markovian or have noise added to the right-hand sides. The items are:

New seed Use this to reseed the random number generator. If you use the same seed then the results will not change from run to run.

Compute This will put up the same dialog box as the "Integrate" "Range" choice. Two new data sets will be created that will compute the mean and the variance of the point by point values of the trajectories over the number of trial runs you choose. If the system is completely deterministic and the parameters and initial conditions are identical for each run, then this is superfluous. Otherwise, the mean and variance are computed. You can then access these new arrays as described below. If you fire up the sample Markov problem, choose the "Compute" option, and set keep the initial data constant over say 20 runs, then you can look at the mean trajectory and its variance for each of your variables.

Data This puts the results of the most recent run into the data browser and enables plotting of them.

Mean This puts the results of the mean value of the most recently computed set of trials.

Variance This does the same for the variance.

Histogram This computes a histogram for a chosen variable and additional conditions and replaces the "t" column and the first variable column with the bin values and the number per bin respectively. You will be prompted for the number of bins, a maximum and minimum value and the variable on which to perform the histogram. Finally, you will be asked for additional conditions that involve the other stored variables (not the fixed ones though.) For example, suppose you have run an ODE/Markov system and you want the distribution of a continuous variable when the Markov variable is in state 1. Then the additional condition would be `z==1` where `z` is the Markov variable. Multiple conditions are made by using the `&` and `|` expressions. Note that `==` is the logical equal and is not the same as the algebraic one.

Old Hist brings back the most recently computed histogram.

Fourier This prompts you for a data column. It then computes a Fourier transform (FFT). The results are in the Browser. The first column (labeled "T") is the mode. The second, the cosine component and the third, the sine component.

Power computes the power spectrum and the phase using the FFT.

fIt curve This is a routine based on Marquardt-Levenberg algorithm for nonlinear least squares fitting. A description of the method can be found in *Numerical Recipes in C.* In this implementation, one can choose parameters and initial data to vary in an attempt to minimize the least-squares difference between solutions to a dynamical system and data. The data must be in a file in which the first column contains the independent values in increasing order. The remaining columns contain data which are to be fitted to solutions to a DE. Not all the columns need be used. When you choose this option, a window pops up with 10 entries describing the fit parameters. The items are:

File This is the name of the data file. The first column must contain the times at which the data was taken.

NCols This contains the total number of columns in the data file. This includes all columns in the file, even those that you will not use.

Fitvar This is a list separated by commas or spaces of variables that you want to fit to the data. These must not be Markov variables or Auxiliary variables. They are restricted to the items that you define as "Variables" in the ODE file. Due to laziness, you can only have as many variables as you can type in 25 or fewer characters.

To Col This should contain a list of column numbers in the data file associated with each of the variables you want to fit. Thus, for example, if you want to fit "x" to column 5 and "y" to column 2, you would type "x y" in the "Fitvar" entry and "5 2" in the "To Col" entry. The number of columns in this must equal the number of variables to be fit.

Params These items (there are 2 of them in case you have lots of parameters you need to vary) contain the names of parameters and variables. If the name is a variable, then the initial data for that variable will be adjusted. If it is a parameter, then the parameter will be adjusted. On the initial call, the current initial data and parameter values are used. The lists of parameters and initial data can be separated by spaces or commas.

Tolerance This is just a small number that tells the algorithm when the least square error is not changing enough to be significant. That is if either the difference is less than "TOL" or the ratio of the difference with the least square is less than "TOL" then the program will halt successfully. On should not make this too small as such differences are insignificant and a waste of CPU time. The default of .001 seems to work well.

Npts This is the number of points in the data file that you want to fit to.

Epsilon This is used for numerical differentiation. 1e-5 is a good value since we really don't need precise derivatives.

Max iter This is the maximum number of iterates you should use before giving up.

On return, the program will put the best set of parameters that it has found. It currently is quite verbose and prints a lot of stuff to the console. This is mainly info about the current values of the parameters and the least square.

Liapunov exponent attempts to compute the maximum Liapunov exponent of the current simulation. The method is pretty simplistic but works on the examples I have fed it. Given a solution $x(t)$ at a series of points $t_1, \ldots, t_n$ I perturb the solution at each time point, integrate the equation to the next time point, and compute the logarithm of the rate of growth. This is averaged over the whole time series to give an approximation. The size of the perturbation is determined by the numerical parameter `JAC_EPS` which can be set from the numerics menu under Sing pt ctl. You will be prompted as to whether you want to compute the exponent over a range of parameters. If you choose a range, then the range dialog box will appear; fill it in as usual.

loo(K)up This allows you to change the definitions of tabulated functions by reading in a different file or changing the formula. Thus, if you have many experimental sets of data, you can read them in one by one and integrate the equations. You are prompted for the name of a tabulated function. Then you give the filename to read in. You will continue to be prompted and can type a few carriage returns to get out. If the table was defined as a function instead of a file, then you will be prompted for the number of points, the limits of the range (`Xhi,Xlo`) ad finally, the formula of for the function defining the table. Note that it must be a function of `t`. Note that if the function contains parameters and these are changed, it will be automatically recomputed unless the AUTOEVALUATE flag is set to 0. By default, it is set to 1. You would likely set it to zero if you want to create a random table in order to implement "frozen" noise.

bndry(V)al prompts you for the maximum iterates, the error tolerance, and the deviation for the numerical Jacobian for the shooting method for solving BVPs.

(A)veraging This allows you to compute the adjoint and do averaging for weakly coupled oscillators. To use this option, you must successfully compute a periodic orbit. This does not work well with stiff systems so good luck. The menu that pops up is:

(N)ew adj This makes the adjoint from the computed periodic data. Success or failure will be noted. Separate storage is maintained for

the adjoint. The program automatically puts the data from the adjoint into the browser so it can be viewed and plotted or saved.

(A)djoint This will place the adjoint data in the browser,

(O)rbit This places the periodic orbit in the browser.

(M)ake H This will prompt you for the coupling function and the result will be averaged and placed in 2 columns of storage, the first is the time, then the H function. If there are enough columns, the odd and even parts of the averaged function will be retained. As with the adjoint, this list is automatically placed in the browser. The user will be prompted for the coupling functions and should type in the formulas. The coupling is between two identical units. Say you want to couple two oscillators via a variable called `V` Then, you `V` refers to the oscillator getting the input and `V'` refers to the oscillator providing it. For example, suppose you want to study the behavior of two weakly diffusively coupled Fitzhugh-Nagumo equations:

$$dv_1/dt = f(v_1, w_1) + \epsilon(v_2 - v_1) \quad (5)$$
$$dw_1/dt = g(v_1, w_1) \quad (6)$$
$$dv_2/dt = f(v_2, w_2) + \epsilon(v_1 - v_2) \quad (7)$$
$$dw_2/dt = g(v_2, w_2) \quad (8)$$

Then for the coupling you would input

```
v'-v
```

```
0
```

for the required coupling.

(H) function This places the computed H function in the browser.

Anytime you integrate, etc, the data will be placed back into the storage area.

8 The Data Browser

One window that pops up is the Data Browser that allows you to look at the numbers produced by the program as well as save them to a file and otherwise manipulate them. It is a very primitive spread sheet. Once you have computed a trajectory to an equation, you can use the Data Browser (here after, DB) to look at the data. There are some known "bugs." You must sometimes grab it with the mouse and shake it to get the data to show up (no kidding!) Also, sometimes it will not resize properly; in this case, you must continue the integration to the current starting point (in other words, do nothing but press C and then ¡Return¿.) To activate the DB, bring the window to the top and put the pointer inside the box. Across the top is a menu of commands and then

there follows a list of titles for the variables, time and the auxiliary functions. Using the arrow keys, the page keys or clicking on the appropriate commands allows you to scroll through the data. By resizing the DB you can get more or less data. Clicking on Left shifts the data window to the left and Right moves it to the right. The time column always stays fixed. If you do parametric or range calculations, the range variable is kept in the time column. Home takes you to the top of the data and End to the last row. The remaining commands will be described separately. The keyboard shortcuts to invoke them are in parentheses.

(F)ind This pops up a window and asks for a variable and a value. It then looks through the data until it comes to the closest value to the specified that the variable takes. It only moves down the file so that you can find successive values by starting at the top.

(G)et This makes the top row of data the initial conditions for a new run.

Re(p)lace This pops up a window asking you for a column to replace. Then it prompts you for a formula. Suppose you want to replace `AUX1` with `x+y-t` where `x,y` are two variables. Then type this in when prompted and the column that held `AUX1` will be replaced by the values in these columns. Any valid XPP function or user function can be used. Two special symbols can also be used when applied to single variables:

- **@VARIABLE** replaces the column with the numerical derivative of the variable. You cannot use this within a formula, but once the column is replaced, it can be treated as any other column.
- **&VARIABLE** replaces the column with the numerical integral. Note that successive applications of the derivative and then the integral will result in the original plus a constant.

(U)nreplace This undoes the most recent replacement.

Fir(s)t This marks the top row in the DB (nothing is shown)as the start or first row for saving or restoring.

Last (e) This marks the top row as the last or end row for saving or restoring.

(T)able This lets you save data in a tabulated format that can then be used by XPP as a function or inputs. You must use the `First` and `Last` keys to mark the desired data you want to save. You are then prompted for the column name, the minimum and maximum you want your independent variable to range and the file name. The result is a table file of the format shown in the section on tables.

(R)estore replots the data marked by First and Last. The default is the entire data set

(W)rite prompts you for a filename and writes the marked data to a file. The files are of the form:

```
t0 x1(t0) ... xn(t0)
t1 x1(t0) ... xn(t0)
.
.
.
tf x1(tf) ... xn(tf)
```

and reflect the current contents of the DB. They are ASCII readable.

(L)oad Will load in as much of a similarly formatted data file as possible for graphing.

(A)ddcol This allows you to add an additional column to the data browser. You are prompted for the name you want to give the column and for the formula. It is thus, like an auxiliary variable with a name. Thereafter, it will be computed along with any other quantities that you have defined. It is as though you had included another auxiliary variable in your original file.

(D)elcol This lets you delete a column. You can only delete columns which you have created with the `Addcol` command.

Sometimes the names will not appear or go away on the browser. If you iconify it and then open it up again, they will appear as they should. Another problem that can occur is that you can delete a column that is itself referred to by a different column. This will result in wrong answers in the column. Thus, do not delete columns whose contents are used by other columns. Also, if you delete a column, its name is still known by the internal system but it has no real value. For these reasons, you should delete columns with caution. If the purpose of deleting them is to change the formula, use the right-hand-side editor, (File-Edit) instead.

9 Functional equations

In the course of some research problems, I was pursuing, I ran into some Volterra equations of the form:

$$u(t) = f(t) + \int_0^t K(t, s, u(s))ds$$

that I could not convert to ODES. (If K is a convolution with a sum of powers and exponentials, then it can be converted to an ODE. Since XPP is much more efficient with ODEs and has been thoroughly debugged with respect to them, you should always attempt this conversion first.) Thus, I have added the capability to solve equations with this type of term in them. This has necessitated the addition of two new commands for the ODE file and a solver for such problems. The solver is described below in the numerical section. Since

the equation above requires "memory" all the way back to $t = 0$ and one often is interested in long time behavior, XPP truncates this to:

$$u(t) = f(t) + \int_{\max(t-T,0)}^{t} K(t, s, u(s))ds$$

where `T=DT*MaxPoints`, the latter being a parameter that you set in the numerics menu. The default is 4000. Thus, one assumes that the kernel function decays for large t and so the tail will be small. As `MaxPoints` is a parameter, you can always make it larger at the price of taking longer to evaluate right-hand sides. Let us consider the following equation:

$$u(t) = \sin(t) + \frac{1}{2}(\cos(t) - \exp(t) - t\exp(t)) + \int_0^t (t - s)\exp(-(t - s))u(s)ds$$

whose solution is $u(t) = \sin(t)$. The following ODE file will create this model and also add an auxiliary variable with the solution for purposes of comparison.

```
#  voltex1.ode
u(t)=sin(t)+.5*cos(t)-.5*t*exp(-t)-.5*exp(-t)+int{(t-t')*exp(t'-t)*u}
aux utrue=sin(t)
done
```

The `int{K(u,t,t')}` construction tells XPP that this is a Volterra integral. If your problem can be cast as a convolution problem, considerable speedup can be obtained since lookup tables are created. The present example is in fact a convolution problem, so that instead of the full declaration, one could instead write:

```
#  voltex2.ode
u(t)=sin(t)+.5*cos(t)-.5*t*exp(-t)-.5*exp(-t)+int{t*exp(-t)#u}
aux utrue=sin(t)
done
```

which convolves the first expression (of t **only**) with u. For this example, using a time step of .05 and integrating to 40, there is a 3-fold speed-up using the convolution. For more complicated kernels, it will be more.

If one wants to solve, say,

$$u(t) = exp(-t) + \int_0^t (t - t')^{-mu} K(t, t', u(t'))dt'$$

the form is:

```
u(t)= exp(-t) + int[mu]{K(t,t',u}
```

Note that the "mu" must be a number between 0 and 1. If "mu" is greater than or equal to 1, the integral is singular at 0.

Warning

If you have parameters in your definition of the kernel and you change them, then you will have to go into the numerics menu and recompute the kernels by calling the Method command which automatically recomputes the kernels. Alternatively, turn on the AutoEval flag and it will be done automatically.

I close this section with an example of a pair of coupled oscillators in an infinite bath which has diffusion and passive decay. The equations are:

$$\begin{aligned}
u(t) &= \int_0^t k(t-s)F(u(s),v(s))ds + \int_0^t k_d(t-s)F(u_1(s),v_1(s))ds \\
v(t) &= \int_0^t k(t-s)G(u(s),v(s))ds + \int_0^t k_d(t-s)G(u_1(s),v_1(s))ds \\
u_1(t) &= \int_0^t k(t-s)F(u_1(s),v_1(s))ds + \int_0^t k_d(t-s)F(u(s),v(s))ds \\
v_1(t) &= \int_0^t k(t-s)G(u_1(s),v_1(s))ds + \int_0^t k_d(t-s)G(u(s),v(s))ds
\end{aligned}$$

where

$$\begin{aligned}
k(t) &= \exp(-t)/\sqrt(\pi t) \\
k_d(t) &= \exp(-t)\exp(-d/t)/\sqrt(\pi t)
\end{aligned}$$

and

$$\begin{aligned}
F(u,v) &= \lambda u - v - (u+qv)(u^2+v^2) \\
G(u,v) &= \lambda v + u - (v-qu)(u^2+v^2).
\end{aligned}$$

Note that the kernel k is weakly singular and thus $\mu = .5$.

Note that in addition there is a singularity at $t = 0$ for the diffusive kernel (division by zero); this can be rectified by adding a small amount to the denominator. The XPP file is as follows

```
# lamvolt.ode
# the four variables:
init u=0  v=0  u1=0  v1=0
par lam=1.5  q=0.8  d=1  u0=1  u10=0.95
# 1/sqrt(pi)=
number spi=0.56419
# the integral equations; since (0,0,0,0) is a rest point, I
# add a small quickly decaying transient
u(t)=u0*exp(-5*t)+spi*(int[.5]{exp(-t)#f}+int[.5]{exp(-t-d/(t+.0001))#f1})
v(t)=spi*(int[.5]{exp(-t)#g}+int[.5]{exp(-t-d/(t+.0001))#g1})
u1(t0)=u10*exp(-5*t)+spi*(int[.5]{exp(-t)#f1}+int[.5]{exp(-t-d/(t+.0001))#f})
v1(t)=spi*(int[.5]{exp(-t)#g1}+int[.5]{exp(-t-d/(t+.0001))#g})
# the four functions f,g,f1,g1
f=lam*u-v-(u*u+v*v)*(u+q*v)
g=lam*v+u-(u*u+v*v)*(v-q*u)
```

```
f1=lam*u1-v1-(u1*u1+v1*v1)*(u1+q*v1)
g1=lam*v1+u1-(u1*u1+v1*v1)*(v1-q*u1)
done
```

Try it.

10 Auto interface

AUTO is a program that was written several years ago by Eusebius Doedel. It has the ability to track bifurcation curves for steady-state and periodic systems. The program is very powerful particularly for following periodic orbits. A full FORTRAN implementation of it is available along with documentation from Doedel. His Email is doedel@cs.concordia.edu.

The version supported in XPP is a subset of AUTO but allows you to do most of the things you would nornally want to for autonomous ODEs and with BVPS. In particular, you can track fixed points, find turning points and Hopf bifurcation points, compute two-parameter curves of turning points and Hopf points, compute branches of periodic solutions emanating from a Hopf point, track period-doubling bifurcations, torus bifurcations, and two-parameter curves of fixed period orbits. Points can be imported into XPP as well as complete orbits. The bifurcation diagrams are dynamically produced and you can move around them using the arrow keys. Curves can be saved and reloaded for later use. Diagrams can be saved and imported into the main XPP window.

Click on the `File Auto` menu item to bring up AUTO.

The AUTO window consists of several parts. The menus are on the left. A small square in the lower left tells you about stability. The two windows at the bottom are respectively information about the computed points and the hints or tips window. This bottom window also tells you the coordinates of the main graphics window.

10.1 Preparation

Before you can use AUTO, you must prepare your system for it. You must start your bifurcation analysis from either a fixed point of your model, a periodic orbit, or a solution to a boundary value problem. AUTO seems to work best when you start from a steady state, but I have had success starting at periodic orbits. If you want to start at a steady state, find one and integrate so that the system is at rest. If you want to start at a periodic orbit, then find one and make sure that the total integration time is the "period" of your orbit. This is what the AUTO interface uses as an approximate starting period. There are several ways to do this; the best is to use the boundary value solver of XPP but just plain old integration often works fine. To solve a boundary value problem, it is necessary to find an initial set of parameters for which you can solve the problem within XPP. You should arrange the "length" of the interval to always be 1. That is you must scale the problem so that the domain interval of interest is $[0, 1]$. You must then compute a solution using XPP before calling AUTO.

For discrete dynamical systems, I have added the capability of continuation of $n-$periodic orbits by having AUTO find fixed points of $F^n(x)$. To do this, just set the parameter **nOut** in the XPP numerics menu to the desired period. The example `del_log.ode` has set this up for a period 7 orbit.

For the example file `lecar.ode` the parameter of interest is `iapp` and this has been set at a negative value so that the system has a stable rest state. The variables have been initialized to their rest states as well. Once you have prepared the problem as such, you are ready to run.

Click on the "File" item and choose "Auto." A new window will appear which has several regions. At the left are the 9 commands and the "ABORT" key. Below is a small window for stability information. A large window for the bifurcation curve is labeled with the axes and their limits. The bottom window is for information about the points on the diagram.

10.2 Choosing parameters

The first thing you should do is tell AUTO which parameters you might use in the bifurcation analysis. Up to 5 are allowed. Click on "Parameter" and a list of 5 parameters will appear. Type in the names of the parameters you want to use. For `lecar.ode` use `iapp,phi,gk,vk,gna` The default is the first 5 or fewer parameters in your `ode` file. If you have fewer than 5 parameters, only the available ones will appear.

10.3 Diagram axes

Next, you should tell AUTO the axes and the main bifurcation parameters. Click on "Axes" and 6 choices appear:

(H)i This plots the maximum of the chosen variable.

(N)orm This plots the L_2 norm of the solution.

h(I)-lo This plots both the max and min of the chosen variable (convenient for periodic orbits.)

(P)eriod Plot the period versus a parameter

(T)wo par Plot the second parameter versus the primary parameter for two-parameter continuations.

(Z)oom Use the mous to zoom in on a region.

last (1) par Use the plot parameters from the last 1-parameter plot.

last (2) par Use plot parameters from last 2-parameter plot.

(F)requency Plot Frequency vesus parameter.

(A)verage Plot the average of a variable versus the parameter.

After clicking, a new window pops up with the following items:

Y-axis This is the variable for the y-axis of the plot. For two-parameter and period plots, its contents is ignored.

Main Parm This is the principal bifurcation parameter. It must be one of those you specified in the parameter window. The default is the first parameter in the parameter list.

2nd Parm This is the other parameter for two-parameter continuations.

Xmin ... Ymax The plotting dimensions of the diagram.

Once you press `OK` the axes will be redrawn and labeled. For the present model, set `Xmin=-.5, Ymin=-1.5, Xmax=.5, Ymax=1.0.`

10.4 Numerical parameters

Next, set the NUMERICAL parameters. When you click on this, a new window appears with the following items:

Ntst This is the number of mesh intervals for discretization of periodic orbits. If you are getting apparently bad results or not converging, it helps to increase this. For following period doubling bifurcations, it is automatically doubled so you should reset it later.

Nmax The maximum number of steps taken along any branch. If you max out, make this bigger.

Npr Give complete info every `Npr` steps.

Ds This is the initial step size for the bifurcation calculation. *The sign of* `Ds` *tells AUTO the direction to change the parameter.* Since stepsize is adaptive, `Ds` is just a "suggestion."

Dsmin The minimum stepsize (positive).

Dsmax The maximum step size. If this is too big, AUTO will sometimes miss important points.

Par Min This is the left-hand limit of the diagram for the principle parameter. The calculation will stop if the parameter is less than this.

Par Max This is the right-hand limit of the diagram for the principle parameter. The calculation will stop if the parameter is greater than this.

Norm Min The lower bound for the L_2 norm of the solution. If it is less than this the calculation will stop.

Norm Max The upper bound for the L_2 norm of the solution. If it is greater than this the calculation will stop.

For the present model, you should set `Dsmax` to be 0.05, `Par Min` to -0.45 and `Par Max` to 0.45.

10.5 User functions

Suppose you want to get plots at specific values of parameters or at fixed periods of a limit cycle. Then you can click on "User" which produces a menu 0-9 asking you how many points you want to keep. Click on 0 for none or some other number. A new window will appear with slots for 9 items. You can type in anything of the form:

```
<parameter>=<value>
```

or

```
T=<value>
```

AUTO will mark and save complete information for any point that satisfies either of these criteria. The second is used to indicate that you want to keep a point with a particular period, e.g., `T=25` will save the any periodic orbit with period 25.

10.6 Running

At this point, you are probably ready to run. But before doing a run, here is a hint. You can "save" the diagram at this point (see below under "File"). Although it is an "empty" diagram, all parameters axes, and numerics are saved. You can then reload them later on.

Click on "Run" to run the bifurcation. Depending on the situation, a number of menus can come up. For initial exploration, there are three choices, starting at a new steady state, periodic, or boundary value solution. If you are running the example, click on the steady-state option and a nice diagram will show up and a bunch of points will move around in the stability circle. These indicate stability: for fixed points, they represent exponentials of the eigenvalues; for periodics, the Floquet multipliers. Thus those in the circle are stable and those out of the circle are unstable. Bifurcations occur on the circle. The outer ones are "clipped" so that they will always lie in the square, thus you can keep count of them.

The diagram,itself, has two different lines and two different circles. Stable fixed points are thick lines, stable periodics are solid circles, unstable fixed points are thin lines, and unstable periodics are open circles. Additionally, there are crosses occasionally dispersed with numbers associated with them. These represent "special" points that AUTO wants to keep. There are several of them:

EP Endpoint of a branch

LP Limit point or turning point of a branch

TR Torus bifurcation from a periodic

PD Period doubling bifurcation

UZ User defined function

MX Failure to converge

BP Bifurcation or branch point

Output every Npr^{th} point.

10.7 Grabbing

You can use these special points to continue calculations with AUTO. The "Grab" item lets you peruse the diagram at a leisurely pace and to grab special points or regular points for importing into XPP or continuing a bifurcation calculation. Click on "Grab" and stuff appears in the info window and a cross appears on the diagram. Use the left and right arrow keys to cruise through the diagram. The right key goes forward and the left backward. At the bottom, information about the branch, the point number, the type of point, the AUTO label, the parameters, and the period are given. The points marked by crosses have lables and types associated with them. The type is one of the above. The label corresponds to the number on the diagram. If point is positive, it is an unstable solution and if it is negative it is stable. As you traverse the diagram, stability is shown in the circle. (NOTE: On some displays, the cross does not appear; for these displays the zoom option also does not work. You should recompile the program with the `XORFIX` flag defined. See the Makefile.)

You can traverse the diagram very quickly by tapping the `Tab` key which takes you the special points only. Type `Esc` to exit with no action or type `Return` to grab the point. If it is a regular point (i.e., not special) then the parameters and the variables will be set to the values for that point within XPP. You can then integrate the equations or look at nullclines, etc. If you grab a special point, then you can use this as a restart point for more AUTO calculations, such as fixed period, two-parameter studies, and continuations. Then, you can run AUTO again. Bifurcation diagrams are cumulative unless you reset them in the "File" menu. That is, new stuff is continually appended to the old. The only limit is machine memory.

If you grab a special point and click on "Run" several possibilities arise depending on the point:

Regular Point Reset the diagram and begin anew. You will be asked first if you want to do this.

Hopf Point **Periodic** Compute the branch of periodics emanating from the Hopf point

- **Extend** Continue the branch of steady states through this point.
- **New Point** Restart whole calculation using this as a starting point
- **Two Param** Compute a two parameter diagram of Hopf points.

Period doubling **Doubling** Compute the branch of period 2 solutions.

Two-param Compute two-parameter curve of period doubling points.

Limit point Compute two parameter family of limit points (fixed points or periodic.)

Periodic point The point is periodic so

Extend Extend the branch

Fixed Period Two parameter branch of fixed period points.

Torus point Compute two-parameter family of torus bifurcations or extend the branch or compute two-parameter fixed period.

Before running, after a point is grabbed, be sure to set up the correct axes and ranges for the parameters.

10.8 Aborting

Any calculation can be gracefully stopped by clicking on the "Abort" key. This produces a new end point from which you can continue. Note that if there are many branches, you may have to press "Abort" several times.

`Clear` just erases the screen and `reDraw` redraws it.

10.9 Saving diagrams

`File` allows you to do several things:

Import orbit If the grabbed point is a special one and is a periodic orbit, this loads the orbit into XPP for plotting. This is useful for unstable orbits that cant be computed by integrating initial data.

Save diagram Writes a file for the complete diagram which you can use later.

Load Diagram Loads a previously saved one.

Postscript This makes a hard copy of the bifurcation diagram

Reset diagram This clears the whole thing.

Write pts This writes a file specified by the user which has 5 columns and describes the currently visible bifurcation diagram. The first column has the coordinates of the x-axis, the second and third columns hold the contents of the y-axis, (e.g. max and min of the orbit). The fourth column is one of 1-4 meaning stable fixed point, unstable fixed point, stable periodic, unstable periodic, respectively. The fifth column is the branch number. The main window of XPP can import files in this format and plot them

10.10 Homoclinics and heteroclinics

A recent version of AUTO includes a library of routines called HOMCONT which allow the user to track homoclinic and heteroclinic orbits. XPP incorporates some aspects of this package. The hardest part of computing a branch of homoclinics is finding a starting point. Consider a differential equation:

$$x' = f(x, \alpha)$$

where α is a free parameter. Homoclinics are codimension one trajectories; that is, they are expected to occur only at a particular value of a parameter, say, $\alpha = 0$. We suppose that we have computed an approximate homoclinic to the fixed point $\bar{x}$ which has an n_s-dimensional stable manifold and an n_u-dimensional unstable manifold. We assume $n_s + n_u = n$ where n is the dimension of the system. The remaining discussion is based on Sandstede et al. The way that a homoclinic is computed is to approximate it on a finite interval; say $[0, P]$. We rescale time by $t = Ps$. We double the dimension of the system so that we can simultaneously solve for the equilibrium point as the parameters vary. We want to start along the unstable manifold and end on the stable manifold. Let L_u be the projection onto the unstable subspace of the linearization of f about the fixed point and let L_s be the projection onto the stable space. Then we want to solve the following system:

$$\begin{aligned}
\frac{dx}{ds} &= Pf(x, \alpha) \\
\frac{dx_e}{ds} &= 0 \\
f(x_e(0)) &= 0 \\
L_s(x(0) - x_e(0)) &= 0 \\
L_u(x(1) - x_e(1)) &= 0
\end{aligned}$$

Note that there are $2n$ differential equations and $2n$ boundary conditions; n for the equilibrium, n_s at $s = 0$ and n_u at $s = 1$. There is one more condition required. Clearly one solution to this boundary value problem is $x(s) \equiv x_e(s) \equiv \bar{x}$ which is pretty useless. However, any translation in time of the homoclinic is also a homoclinic so we have to somehow define a phase of the homoclinic. Suppose that we have computed a homoclinic, $\hat{x}(s)$. Then we want to minimize the least-squares difference between the new solution and the old solution to set the phase. This leads to the following integral condition:

$$\int_0^1 \hat{x}'(s)(\hat{x}(s) - x(s))\, ds = 0.$$

This is *one* more condition which accounts for the need for an additional free parameter.

XPP allows you to specify the projection boundary conditions and by setting a particular flag on in AUTO, you can implement the integral condition. Since

the XPP version of AUTO does not allow you to have more conditions than there are differential equations, you should pick one parameter which will be slaved to all the other ones you vary and let this satisfy a trivial differential equation,

$$\alpha' = 0.$$

Here is the first example of continuing a homoclinic in two-dimensions.

$$x' = y \quad y' = x(1-x) - ax + \sigma xy$$

When $(a, \sigma) = (0, 0)$ there is a homoclinic orbit (Prove this by integrating the equations; this is a conservative dynamical system.) For small a it is possible to prove that there is a homoclinic orbit for a particular choice of $\sigma(a)$ using Melnikov methods (see Holmes and Guckenheimer). We now write the equations as a 5-dimensional system using σ as the slaved parameter and introducing a parameter, P for the period:

$$\begin{aligned} x' &= Pf(x,y) \\ y' &= Pg(x,y) \\ x_e' &= 0 \\ y_e' &= 0 \\ \sigma' &= 0 \end{aligned}$$

where $f(x,y) = y$, $g(x,y) = x(1-x) - ax + \sigma xy$ and the following boundary conditions

$$\begin{aligned} 0 &= f(x_e, y_e) \\ 0 &= g(x_e, y_e) \\ 0 &= L_s(x(0) - x_e, y(0) - y_e) \\ 0 &= L_u(x(1) - x_e, y(1) - y_e) \end{aligned}$$

and the integral condition. XPP has a defined function for the projection boundary conditions called `hom_bcs(k)` where `k=0,1,...,n-1` corresponding to the total number required. You do not need to be concerned with ordering etc as long as you get them all and you give XPP the required information. Here is the ODE file:

```
# tsthomi.ode
f(x,y)=y
g(x,y)=x*(1-x)-a*y+sig*x*y
x'=f(x,y)*per
y'=g(x,y)*per
# auxiliary ODE for fixed point
xe'=0
ye'=0
# free parameter
```

```
sig'=0
# boundary conditions
b f(xe,ye)
b g(xe,ye)
# project off the fixed point from unstable manifold
b hom_bcs(0)
# project onto the stable manifold
b hom_bcs(1)
par per=8.1,a=0
init x=.1,y=.1
@ total=1.01,meth=8,dt=.001
@ xlo=-.2,xhi=1.6,ylo=-1,yhi=1,xp=x,yp=y
done
```

The only new feature is the projection conditions. *XPP's boundary value solver will not work here since there are more equations than conditions and it doesn't know about the integral condition.* I have set the total integration time to 1 and have added the additional parameter `per` corresponding to the parameter P in the differential equation. I use the Dormand-Prince order 8 integrator as it is pretty accurate. I have also set the view to be the $(x, y)-$plane. Note that this is a pretty rough approximation of the true homoclinic. We will use AUTO to improve this before continuing in the parameter a. Run XPP with this ODE file and integrate the equations. You will get a rough homoclinic pretty far from the fixed point. Click on File Auto to the the AUTO window. Now click on Axes Hi. Choose `xmin=0,xmax=50,ymin=-6,ymax=6` and also select `sig` as the variable in the y-axis. Click on OK and bring up the Auto Numerics dialog. Change `Ntst=35, Dsmin=1e-4,Dsmax=5,Par Max=50,EPSL=EPSU=EPSS=1e-7` and click OK. Now, before you run the program, click on Usr Period and choose 3 for the number. We want AUTO to output at particular values of the parameter `per` corresponding to P. When the dialog comes up, fill the first three entries in as `per=20,per=35,per=50` respectively and click OK. This forces AUTO to output when P reaches these three values. Now, click on Run and choose Homoclinic. A little dialog box appears. Fill it in as follows: `Left Eq: Xe Right Eq: Xe NUnstable: 1 NStable: 1`. You must tell AUTO the dimension of the stable and unstable manifolds as well as the fixed point to which the orbit is homoclinic. (Note that if you ever fill this in wrong or need to change it, you can access it from the main XPP menu under Bndry Value Homoclinic.) Once you click on OK, you should see a straight line across the screen as the homoclinic approximation gets better. Click on Grab and grab the second point corresponding to the point `Per=35`. For fun, in the XPP window, click on Initial Conds Go and you will see a much better homoclinic orbit.

Now that we have a much improved homoclinic orbit, we will continue in the parameter a as desired. First, lets make sure we get the orbits when $a = -6, -4, -2, 2, 4, 6$ so we will click on Usr Period and choose 6. Type in `a=-6,a=-4, etc` for the first 6 entries and then click OK. Click on Axes Hi to change the axes and the continuation parameter. Change the `Main Parm` to

a, Xmin=-7,Xmax=7 and click OK. Click on Numerics and change Par Min=-6, Par Max=6 and then click OK. Now click on Run and you will see a line that is almost diagonal. When done, click on Grab again, and watch the bottom of the AUTO window until you see Per=35 and click Enter. In the Numerics menu, change Ds=-.02 to change directions, and click Ok. Now click Run and there will be another diagonal line that is in the opposite direction. Click on Grab and grab point number 7 corresponding to a=6. In the XPP window, click on Init Conds go and you will see a distorted homoclinic. It is not that great and could be improved probably by continuing with Per some more. Grab the point labeled 11 (a=-6) and in XPP try to integrate it. It doesn't look even close. This is because the homoclinic orbit is unstable and shooting (which is what we are doing when we integrate the equation) is extremely sensitive to the stability of the orbits. In the AUTO window, click on File Import Orbit to get the orbit that AUTO computed using collocation. In the XPP main window, click on Restore and you will see a much better version of the homoclinic orbit. This is because collocation methods are not sensitive to the stability of orbits! In fact, you can verify that the fixed point (0,0) is a saddle-point with a positive eigenvalue, λ_u and a negative one of λ_s whose sum is the trace of the linearized matrix, $-a$. The sum of the eigenvalues is called the saddle-quantity and if it is positive (for us, $a < 0$), then the homoclinic is unstable.

We now describe how to find heteroclinic orbits. The methods are the same except that we must track two *different* fixed points. Thus, we need an additional n equations for the other fixed point. As with homoclinic orbits, we go from the unstable manifold to the stable manifold. In this case, the "left" fixed point is the one emerging from the unstable manifold and the "right" fixed point is the one going into the stable manifold. Thus, the dynamical system is :

$$
\begin{aligned}
\frac{dx}{ds} &= Pf(x,\alpha) \\
\frac{dx_{left}}{ds} &= 0 \\
\frac{dx_{right}}{ds} &= 0 \\
f(x_{left}(0)) &= 0 \\
f(x_{right}(1)) &= 0 \\
L_s(x(0) - x_{left}(0)) &= 0 \\
L_u(x(1) - x_{right}(1)) &= 0.
\end{aligned}
$$

The only difference is that we have the additional n equations for the right fixed point and the n additional boundaty conditions. It is important that you give good values for the initial conditions for the two fixed points since they are different and you need to converge to them. The classic bistable reactrion-diffusion equation provides a nice example of a heteroclinic. The equations are:

$$-cu' = u'' + u(1-u)(u-a)$$

which we rewrite as a system:

$$\begin{aligned} u' &= u_p \equiv f(u,u_p) \\ u_p' &= -cu_p - u(1-u)(u-a) \equiv g(u,u_p) \end{aligned}$$

The fixed point $(1,0)$ has a one-dimensional unstable manifold and $(0,0)$ as a one-dimensional stable manifold. We seek a solution from $(1,0)$ to $(0,0)$. For $a = 0.5$ and $c = 0$, there is an exact solution joining the two saddle points. (Prove this by showing that

$$u_p^2 + u^4/2 - 2u^3/3 + u^4/2$$

is constant along solutions when $a = 0.5, c = 0$.) We will use this as a starting point in our calculation. Here is the ODE file:

```
# tstheti.ode
# a heteroclinic orbit
# unstable at u=1, stable at u=0
f(u,up)=up
g(u,up)=-c*up-u*(1-u)*(u-a)
# the dynamics
u'=up*per
up'=(-c*up-u*(1-u)*(u-a))*per
# dummy equations for the fixed points
uleft'=0
upleft'=0
uright'=0
upright'=0
# the velocity parameter
c'=0
# fixed points
b f(uleft,upleft)
b g(uleft,upleft)
b f(uright,upright)
b g(uright,upright)
# projection conditions
b hom_bcs(0)
b hom_bcs(1)
# parameters
par per=6.67,a=.5
# initial data
init u=.918,up=-.0577,c=0
# initial fixed points
init uleft=1,upleft=0,uright=0,upright=0
@ total=1.01,dt=.01
@ xp=u,yp=up,xlo=-.25,xhi=1.25,ylo=-.75,yhi=.25
# some AUTO parameters
```

```
@ epss=1e-7,epsu=1e-7,epsl=1e-7,parmax=60,dsmax=5,dsmin=1e-4,ntst=35
done
```

I have added a few AUTO numerical settings so that I don't have to set them later. Run XPP with this ODE file and integrate the equations. We will now continue this approximate heteroclinic in the parameter `per`. Fire up AUTO (File Auto) and click on Axes Hi. Put `c` on the y-axis and make `Xmin=0,Xmax=60,Ymin=-2,Ymax=2`. Then click OK. As above, we will also keep solutions at particular values of `per` by clicking on `Usr period 3`, choosing `per=20,per=40,per=60`, and then OK. Now we are ready to run. Click on Run Homoclinic. When the dialog box comes up set the following: `Left Eq: ULEFT, Right Eq: URIGHT, NUnstable:1, NStable:1` and then click OK. You should see a nice straight line go across the screen. Grab the point labeled `per=40` and then click on File Import Orbit. Look at it in the XPP main window and freeze it. Now in the Auto window, click on Axes Hi and change the Main Parm to `a` and `Xmax=1`. Click OK and then click on Numerics to get the Auto Numerics dialog. Change `Dsmax=0.1, Par Max=1` and click OK. Now click on Usr Period 4 and make the four user functions `a=.75,a=.9,a=.25,a=.1` and click OK. Now click on Run and watch a line drawn across the screen. This is the velocity, c as a function of the threshold, a. Click on Grab and looking at the bottom of the screen, wait until you see `per=40` and then click on Enter. Now, open the Numerics dialog box and change `Ds=-.02` to go the other direction. Click on Run and you should see the rest of the line drawn across the screen. Click on Grab and move to the point labeled `a=0.25` Click on File Import Orbit and plot this in the main XPP window.

11 Creating Animations

Many years ago, as a teenager, I used to make animated movies using various objects like Kraft caramels ("Caramel Knowledge") and vegetables ("The Call of the Wild Vegetables"). After I got my first computer, I wanted to develop a language to automate computer animation. As usual, things like jobs, family, etc got in the way and besides many far better programmers have created computer assisted animation programs. Thus, I abandoned this idea until I recently was simulating a simple toy as a project with an undergraduate. I thought it would be really cool if there were a way to pipe the output of a solution to the differential equation into some little cartoon of the toy. This would certainly make the visualization of the object much more intuitive. There were immediately many scientific reasons that would make such visualization useful as well. Watching the gaits of an animal or the waving of cilia or the synchronization of many oscillators could be done much better with animation than two and three dimensional plots of the state variables.

With this in mind, I have developed a simple scripting language that allows the user to make little cartoons and show them in a dedicated window. The following steps are required

- Run the numerical simulation for however long you need it.
- Using a text editor create a description of the animation using the little scripting language described below.
- Click on the `(V)iew axes (T)oon` menu item from the main XPP window.
- Click on the `File` item in the animation window that pops up and give it the name of your script file.
- If the file is OK, click on `Go` item and the animation will begin.

11.1 The animation window

A resizeable window will pop up when you choose the `Toon` item form the `Viewaxes` submenu. This has 6 buttons on it. They are

- `File` Click on this to load a new animation file. Hereafter, I will call these `ani` files. The usual extension will be `filename.ani`.
- `Go` Click on here after you have loaded an ani file and it will run.
- `Reset` This moves the animation back to the beginning
- `Fast` speeds up the animation
- `Slow` slows it down
- `Pause` pauses the animation. `Go` restarts it from where it started. This is very sluggish and it takes a long time to respond to it. I hope to fix this someday.
- `Skip` sets the numbers of frames ot skip
- `>>>>` Move forward one frame
- `<<<<` Move back one frame
- `Mpeg` Store a series of ppm files for use with the mpeg_encode program. This takes a ton of disk space. Better yet, use the `anigif` option which creates an animated gif file. 600 frames can be as little as 500K so it wont eat up disk space.

Some computer systems cannot produce the required amount of graphics memory for a pixmap and if this happens, you cannot currently run the animation. You will be told this and the animation window will never appear.

11.2 DASL: Dynamical Animation Scripting Language

In order to use the animation components of XPP, you must first describe the animation that you want to do. This is done by creating a script with a text editor that describes the animation. You describe the coordinates and colors of a number of simple geometric objects. The coordinates and colors of these objects can depend on the values of the variables (both regular and fixed, but not auxiliary) at a given time. The animation then runs through the output of the numerical solution and draws the objects based on that output. I will first list all the commands and then give some examples.

Basically, there are two different types of objects: (i) transient and (ii) permanent. Transient objects have coordinates that are recomputed at every time as they are changing with the output of the simulation. Permanent objects are computed once and are fixed through the duration of the simulation. The objects themselves are simple geometric figures and text that can be put together to form the animation.

Each line in the script file consists of a command or object followed by a list of coordinates that must be separated by semicolons. For some objects, there are other descriptors which can be optional. Since color is important in visualization, there are two ways a color can be described. Either as a formula which is computed to yield a number between 0 and 1 or as an actual color name started with the dollar sign symbol, $. The `ani` file consists of a lines of commands and objects which are loaded into XPP and played back on the animation window. Here are the commands:

dimension xlo;ylo;xhi;yho

speed delay

transient

permanent

line x1;y1;x2;y2;color;thickness

rline x1;y1;color;thickness

rect x1;y1;x2;y2;color;thickness

frect x1;y1;x2;y2;color

circ x1;y1;rad;color;thickness

fcirc x1;y1;rad;color

ellip x1;y1;rx;ry;color;thickness

fellip x1;y1;rx;ry;color

comet x1;y1;type;n;color

text x1;y1;s

vtext x1;y1;s;z

settext size;font;color

xnull x1;y1;x2;y2;color;id

ynull x1;y1;x2;y2;color;id

end

All commands can be abbreviated to their first three letters and case is ignored. At startup the dimension of the animation window in user coordinates is (0,0) at the bottom left and (1,1) at the top right. Thus the point (0.5,0.5) is the center no matter what the actual size of the window on the screen. **Color** is described by either a floating point number between 0 and 1 with 0 corresponding to red and 1 to violet. When described as a floating point number, it can be a formula that depends on the variables. In all the commands, the color is optional *except* **settext.** The other way of describing color is to use names which all start with the $ symbol. The names are: **$WHITE, $RED, $REDORANGE, $ORANGE, $YELLOWORANGE, $YELLOW, $YELLOWGREEN, $GREEN, $BLUEGREEN, $BLUE,$PURPLE, $BLACK**.

The **transient** and **permanent** declarations tell the animator whether the coordinates have to be evaluated at every time or if they are fixed for all time. The default when the file is loaded is **transient.** Thus, these are just toggles between the two different types of objects.

The number following the **speed** declaration must be a nonnegative integer. It tells tha animator how many milliseconds to wait between pictures.

The **dimension** command requires 4 numbers following it. They are the coordinates of the lower left corner and the upper right. The defaults are (0,0) and (1,1).

The **settext** command tells the animator what size and color to make the next text output. The size must be an integer, **{ 0,1,2,3,4 }** with 0 the smallest and 4 the biggest. The font is either **roman** or **symbol.** The color must be a named color and not one that is evaluated.

The remaining ten commands all put something on the screen.

- **line x1;y1;x2;y2;color;thickness** draws a line from **(x1,y1)** to **(x2,y2)** in user coordinates. These four numbers can be any expression that involves variables and fixed variables from your simulation. They are evaluated at each time step (unless the line is **permanent**) and this is scaled to be drawn in the window. The **color** is optional and can either be a named color or an expression that is to be evaluated. The **thickness** is also optional but if you want to include this, you must include the **color** as well. **thickness** is any nonnegative integer and will result in a thicker line.

- **rline x1;y1;color;thickness** is similar to the **line** command, but a line is drawn from the endpoints of the last line drawn to **(xold+x1,yold+y1)** which becomes then new last point. All other options are the same. This is thus a "relative" line.

- **rect x1;y1;x2;y2;color;thickness** draws a rectangle with lower corner **(x1,y1)** to upper corner **(x2,y2)** with optional color and thickness.

- **frect x1;y1;x2;y2;color** draws a filled rectangle with lower corner **(x1,y1)** to upper corner **(x2,y2)** with optional color.

- **circ x1;y1;rad;color;thick** draws a circle with radius **rad** centered at **(x1,y1)** with optional color and thickness.

- **fcirc x1;y1;rad;color** draws a filled circle with radius **rad** centered at **(x1,y1)** with optional color.

- **ellip x1;y1;rx;ry;color** draws an ellipse with radii **rx,ry** centered at **(x1,y1)** with optional color and thickness.

- **fellip x1;y1;rx;ry;color** draws a filled ellipse with radii **rx,ry** centered at **(x1,y1)** with optional color.

- **comet x1;y1;type;n;color** keeps a history of the last `n` points drawn and renders them in the optional `color`. If `type` is non-negative, then the last n points are drawn as a line with thickness in pixels of the magnitude of `type`. If `type` is negative, filled circles are drawn with a radius of `-thick` in pixels.

- **text x1;y1;s** draws a string **s** at position **(x1,y1)** with the current color and text properties. Only the coordinates can depend on the current values.

- **vtext x1;y1;s;z** draws a string **s** followed by the floating point value **z** at position **(x1,y1)** with the current color and text properties. Thus, you can print out the current time or value of a variable at any given time.

- **xnull x1;y1;x2;y2;color;id** uses the nullclines that you have already computed in your animation. You can use the static nullclines by just choosing **-1** for the **id** parameter. To use dynamic nullclines, you must compute a range of nullclines using the Nullcline Freeze Range command. The parameter **id** runs from 0 to N where N is the number of nullclines that you have computed in the range dialog. The animator converts **id** to an integer and tests whether it is in the range and then loads the appropriate nullcline. There is an example shown below. The **ynull** command is identical. The parameters **x1,y1,x2,y2** tell the animator the window in which the nullclines are defined. These should be the lower-left and upper right corners of the phaseplane where the nullclines were computed.

REMARK. As with lines in ODE files, it is possible to create arrays of commands using the cobination of the **[i1..i2]** construction. Some examples are shown below.

11.3 Examples

I will start out with a simple pendulum example and then a bunch of more interesting examples. Here is the old pendulum again:

```
# damped pendulum pend.ode
dx/dt = xp
dxp/dt = (-mu*xp-m*g*sin(x))/(m*l)
pe=m*g*(1-cos(x))
ke=.5*m*l*xp^2
aux P.E.=pe
aux K.E.=ke
aux T.E=pe+ke
x(0)=2
param m=10,mu=1,g=9.8,l=1
param scale=0.008333
@ bounds=1000
done
```

I have added an initial condition and another parameter used to scale the magnitude of the kinetic energy for a later animation. Fire up XPP and run this simulation. Now we will create a very simple animation:

```
# pend.ani
# simple animation file for pendulum
line .5;.5;.5+.4*sin(x);.5-.4*cos(x);$BLACK;3
fcircle .5+.4*sin(x);.5-.4*cos(x);.05;$RED
end
```

Notice that comments are allowed. This file is included with the distribution as is the ODE file so you don't have to type it in. There are only two lines of code. The first tells the animator to draw a line from the center of the screen at **(.5,.5)** to a point **(5+.4*sin(x), .5-.4*cos(x))**, where **x** is the variable in the ODE file for the pendulum. The line has thickness 3 and is black. The next line of code says to draw a filled circle centered at the same point as the line was with radius **0.05**. This will be colored red. Finally, we tell the interpreter that this is the end of the commands. In case you haven't already done it, click on (Initialconds) (Go) to solve the ODE. You should see a damped oscillation. Now click on (Viewaxes) (Toon). A new window will appear with the "test pattern" on the screen. In this window click on (File) and type in **pend.ani** at the prompt. XPP will tell you that two lines were loaded successfully. Comments are ignored. Click on (Go) in the animation window. You will see a pendulum appear and rock back and forth. It may be somewhat jerky depending on the

server and graphics properties of your computer and graphics. You can stop it by clicking on (Pause), speed it up by clicking on (Fast) and slow it down by clicking on (Slow). The reaction to mouse clicks is terribly slow, so to test animation, I would integrate just for a short time at first until you are satisfied. You can edit the file and reload it with the (File) command.

Now we consider a much more complicated animation file:

```
# pend2.ani
# fancy animation file for pendulum
PERMANENT
settext 3;rom;$PURPLE
text .25;.9;Pendulum
line 0;.5;1;.5;$BLUE;4
SPEED 10
TRANSIENT
line .5;.5;.5+.4*sin(x);.5-.4*cos(x);$BLACK;3
fcircle .5+.4*sin(x);.5-.4*cos(x);.05;1.-scale*ke
settext 1;rom;$BLACK
vtext .05;.1;t=;t
settext 1;sym;$BLACK
vtext .05;.05;q=;x
end
```

The first noncomment tells the animator that what follows will be on every frame exactly as intially defined. The text is made fairly large and purple in Times-Roman font. The **text** command puts the text a quarter away across the screen near the top and write "Pendulum." Next a thick blue line is drawn across the middle of the screen to act as a "tether" for the pendulum. We set the delay between frame to be 10 milliseconds with the **SPEED** command. Then all the remaining objects are to be **TRANSIENT.** The first line drawn is the arm of the pendulum. At the end, we place a filled circle, but the color of the circle is proportional to the kinetic energy. **NOTE:** We have used the **fixed variable** version of the kinetic energy, **ke** and not the **auxiliary variable, K.E.** since the latter is not "known" to the internal formula compiler but the former is. Next we set the text color and font stuff to small black roman letters and use the **vtext** command to tell the user the current time. Finally, we set the text to symbol and plot the value of the angle, θ which is the same key as the letter "q."

Click on the (File) button in the animator. Load the file called **pend2.ani** and run it.

The next example is of a large dynamical system that represents a set of coupled excitable cells. The ODE file is called `wave.ode` and is included in the distribution. Here it is

```
# wave.ode
# pulse wave with diffusional coupling
```

```
param a=.1,d=.2,eps=.05,gamma=0,i=0
vv0(0)=1
vv1(0)=1
f(v)=-v+heav(v-a)
#
vv0'=i+f(vv0)-w0+d*(vv1-vv0)
vv[1..19]'=i+f(vv[j])-w[j]+d*(vv[j-1]-2*vv[j]+vv[j+1])
vv20'=i+f(vv20)-w20+d*(vv19-vv20)
#
w[0..20]'=eps*(vv[j]-gamma*w[j])
@ meth=qualrk,dt=.25,total=150,xhi=150
done
```

Now we will create an animation file that plots the values of the voltages, **v0, ..., v20** as beads along the vertical axis whose horizontal height is proportional to their voltage and whose color is proportional to the value of the recovery variables, **w0, ..., w20**. Since I have run the simulation, I know that the recovery variables are between 0 and 1 and that the voltages are between -1 and 1. Here is the one-line animation file for this effect. It is called `wave.ani` and is included with the distribution:

```
# wave.ani
# animated wave
fcircle .05+.04*[0..20];.5*(vv[j]+1);.02;1-w[j]
end
```

This file uses the "array" capabilities of the XPP parser to expand the single line into 21 lines from 0 to 20. I just draw a filled circle at scaled vertical coordinate and horizontal coordinate with a small radius and colored according to the recovery variable. The horizontal coordinate is expanded to be `.05 + .04*0`, `.05 + .04*1`, etc; the vertical is `.5*(vv0+1)`, `.5*(vv1+1)`, etc; and the color is `1-w0`, 1-w1, etc. Thus this is interpreted as a 21 line animation file. Try it to see what it looks like. It is a simple matter to add a vertical scale and time ticker.

This next example illustrates the use of the relative line command. Here, the model is a chain of 20 oscillators representing the phases of spinal motoneurons which control the muscles of the lamprey, an eel-like animal. We will also compute a cumulative "bend" angle which is dependent on the phase of each oscillation. Here is the ODE file, called `lamprey.ode`

```
# example of a chain of coupled oscillators
par grad=0,phi=.1
h(u)=sin(u+phi)
par af=1,ar=1
par bend=.1
x1'=1+grad+af*h(x2-x1)
x[2..19]'=1+grad*[j]+ar*h(x[j-1]-x[j])+af*h(x[j+1]-x[j])
```

```
x20'=1+20*grad+ar*h(x9-x10)
# here is cumulative bend
an1=bend*sin(x1)
an[2..20]=bend*sin(x[j])+an[j-1]
@ bound=1000
@ total=50
done
```

Fire up XPP and run this. (Note that this should be run on a torus phase-space, but since we are only looking at the animation, it is not important.) Now get the animation window and load in the file `fish.ani` which looks like:

```
# fish.ani
# lamprey swimmer -- oscchain.ode
line 0;.5;.04*cos(an1);.5+.04*sin(an1);$BLACK;3
rline .04*cos(an[2..20]);.04*sin(an[j]);$BLACK;3
END
```

Click on (Go) to watch it swim! I draw a series of short line segments relative to the previous one and at an angle that is determined by the **bend** parameter in the ODE file and on the phase of the controlling oscillator. For fun, rerun the simulation with the parameter **grad** set to 0.1.

The penultimate example revisits the Lorenz equations. These equations can be derived by looking at a simple water wheel which consists of a circle of leaky cups with water dripping into them. (See Strogatz for a nice derivation of the equations.) The angle of one of the cups with respect to the viewer is found by integrating the angular velocity which is proportional to the **x** variable in the equations. Thus, as a final example, I present an animation with 8 cups of the Lorenz water wheel. I have created another ODE file that has additional info that I will use called `lorenz2.ode`. Here it is:

```
# the famous Lorenz equation set up for animated waterwheel and
# some delayed coordinates as well
init x=-7.5  y=-3.6  z=30
par r=27  s=10  b=2.66666
par c=.2  del=.1
x'=s*(-x+y)
y'=r*x-y-x*z
z'=-b*z+x*y
# x is proportional to the angular velocity so integral is angle
theta'=c*x
th[0..7]=theta+2*pi*[j]/8
# approximate the velocity vector in the butterfly coords
z1=z-del*(-b*z+x*y)
x1=x-del*(s*(-x+y))
@ dt=.025, total=40, xplot=x,yplot=y,zplot=z,axes=3d
@ xmin=-20,xmax=20,ymin=-30,ymax=30,zmin=0,zmax=50
```

```
@ xlo=-1.5,ylo=-2,xhi=1.5,yhi=2,bound=10000
done
```

Fire it up with XPP and integrate it. Then load the animation file called `lorenz.ani` and run it. Here is the file:

```
# shows the waterwheel using the integrated ang velocity
# see Strogatz book.  Use lorenz2.ode
PERMANENT
circ .515;.515;.46;$BLACK;2
TRANSIENT
SPEED 20
frect .5+.45*sin(th[0..7]);.5+.45*cos(th[j]);.55+.45*sin(th[j]);.55+.45*cos(th[j]);$BLACK
# plotting the butterfly and a lagged version of it !!
fcirc .5+x/40;z/50;.02;$GREEN
fcirc .5+x1/40;z1/50;.02;$RED
end
```

The waterwheel can be seen when running the animation. In the center of the screen are two colored dots that represent the (x, z) coordinates (green) of the attractor and the approximate velocity of these two variables in red.

The last example shows the use of dynamic nullclines. Here is the ODE file:

```
init u=.0426,v=.0843
u'=-u+f(aee*u-aie*v-te+stim(t))
v'=(-v+f(aei*u-aii*v-ti))/tau
par aee=15,aie=9,te=3
par aei=20,aii=3,ti=3,tau=5
stim(t)=s0+s1*if(t<tdone)then(t/tdone)else(0)
par s0=0,tdone=5,s1=1.2
f(u)=1/(1+exp(-u))
@ xp=u,yp=v,xlo=-.1,ylo=-.1,xhi=1.1,yhi=1.1,total=50
done
```

and here is the animation file

```
xnull -.1;-.1;1.1;1.1;$RED;10*stim(t)
ynull -.1;-.1;1.1;1.1;$GREEN;10*stim(t)
fcircle (u+.1)/1.2;(v+.1)/1.2;.025;$BLACK
end
```

The stimulus goes from 0 to 1.2. Since nullclines are computed with $t = 0$, the stimulus is essentially zero as far as XPP is concerned when the nullclines are computed. However, I have added a dummy parameter `s0` which we will vary between 0 and 1.2 to get a family of nullclines. Thus, click on Nullcline Freeze Range and use `s0` as the range parameter, 12 steps, with a Low value of 0 and a High value of 1.2. You will see 13 nullclines drawn. Think of them as nullclines $0, 1, 2, \ldots, 12$. They correspond to `s0`$=0, 0.1, \ldots, 1.2$. Integrate the ODEs. Click

on Viewaxes Toon and load the above animation file. Click on Go and watch it animate the nullclines as well as show the solution. The first 4 terms in the nullcline directive scale the nullclines to fit on the animator. They are the same values as the phaseplane window. The next term is just the color. The final **id** parameter is coded as 10 times the stimulus. Thus, when the stimulus is at .85, this evaluates to 8.5 and XPP truncates this to the integer, 8, and draws nullcline "8" which is the nullcline for a stimulus of strength 0.8. If you draw enough of them, then it wont appear to jump much.

11.4 MPEG

There is a button called MPEG in the animation window. This does not actually create MPEG movies but will help you to make them. To make a movie from an animation you need the following

1. LOTS OF DISK SPACE!!
2. **mpeg_encode** This is a freely available program that allows you to encode a variety of graphics formats into an mpeg file. You should try to find this on the WWW.
3. An input file which I will call mpgfile. I include a sample below that should be self explanatory.

Here is how to make a permanent movie. (Note that I have not had any success on my fancy monitor and machine as I think that TRUE color or whatever doesent work – however on older machines, with less fancy graphics cards, this works fine.)

1. Make a separate directory and move the ODE file and the ANI file there. Run the simulation and test the animation file to see that it works the way you want.
2. Click on the MPEG button and set the MPEG(0/1) flag to 1, give a base name for the ppm frames and determine the skipping number of frames. 1 means every frame, 2 means every second frame, etc. Click OK and you will be asked if you have enough disk space. If yes, then click OK.
3. Re-run the animation. It will seem slow since XPP is writing lots of stuff to the disk.
4. Do an ls *.ppm to see how many frames were stored. Then use this info to edit the **mpgfile**. Save the input file.
5. Type **mpeg_encode mpgfile** and wait. When done, you will have an mpg file.
6. Delete all the *.ppm files.

Here is my input file **mpgfile**. Look for the lines with the !!! as they are what you likely will change.

```
# parameter file with good default values
#
# use this as a guideline for any parameters you don't really understand
# or don't care about
#
PATTERN I
FORCE_ENCODE_LAST_FRAME
# !!!!! Change this to give your movie a nice name !!!
OUTPUT test.mpg
#
#
#
BASE_FILE_FORMAT  PPM
GOP_SIZE 30
SLICES_PER_FRAME 1

PIXEL FULL
RANGE 10
PSEARCH_ALG LOGARITHMIC
BSEARCH_ALG SIMPLE
IQSCALE 8
PQSCALE 10
BQSCALE 25

#IQSCALE 4
#PQSCALE 6
#BQSCALE 10


FRAME_RATE 50

REFERENCE_FRAME ORIGINAL

#
# you really need to understand the following
#
INPUT_CONVERT *

INPUT_DIR .
# !!!!  change the line here to give the list of ppm files !!!
INPUT
frame_*.ppm [0-599]
END_INPUT
```

12 Creating C-files for faster simulations

12.1 Dynamically linked libraries

If your OS supports dynamically linked libraries, then it is easy to hook a right-hand side defined in C or even partially defined in C. You will first have to edit the Makefile so that it will use a dynamically linked library. I do not distribute it with this option turned on since it is seldom used. In the `CFLAGS` definition, add `-DHAVEDLL` and in the `LIBS` definition, add `-ldl`. Recompile XPP with these options.

Now you will be able to load libraries that contain one or more sets of right-hand sides. I will now present an elementary example. Consider the following ODE file `tstdll.ode`

```
# test of dll
x'=xp
y'=yp
xp=0
yp=0
export {x,y,a,b,c,d,t} {xp,yp}
par a=1,b=1,c=1,d=1
done
```

The code beginning with `export` tells XPP we will call an external function routine, passing the 7 items `x,y,a,b,c,d,t` and returning the two items `xp,yp`. Note that they are fixed variables and set to zero at initialization, but XPP will override this when it is run if a library is loaded. They are just dummy place holders for the true right-hand sides. Now lets define the right-hand sides.

Here is the C-file called `funexample.c`

```
#include <math.h>
/*
 some example functions
*/

lv(double *in,double *out,int nin,int nout,double *v,double *cn)
{
  double x=in[0],y=in[1];
  double a=in[2],b=in[3],c=in[4],d=in[5];
   double t=in[6];
  out[0]=a*x*(b-y);
  out[1]=c*y*(-d+x);
}

vdp(double *in,double *out,int nin,int nout,double *v,double *cn)
{
```

```
  double x=in[0],y=in[1];
  double a=in[2],b=in[3],c=in[4],d=in[5];
   double t=in[6];
  out[0]=y;
  out[1]=-x+a*y*(1-x*x);
}

duff(double *in,double *out,int nin,int nout,double *v,double *cn)
{
  double x=in[0],y=in[1];
  double a=in[2],b=in[3],c=in[4],d=in[5];
 double t=in[6];
  out[0]=y;
  out[1]=x*(1-x*x)+a*sin(b*t)-c*y;
}
```

This defines 3 different models, the Lotka-Volterra equation, the van der Pol equation, and the Duffing equation. Note that in addition to the parameters and variables that are passed by the user, XPP also passes all the variable and parameter information. The order is generally as follows. The array `v` contains `t`, the variables in the order they are defined followed by the fixed variables in the order defined. The array `cn` contains the parameters define in your model. `cn[2]` contains the first parameter and the others are defined in the order created. Thus, for the ode file `tstdll.ode`, we have the following identifications:

```
cn[2]=a,cn[3]=b,cn[4]=c,cn[5]=d
v[0]=t,v[1]=x,v[2]=y,v[3]=xp,v[4]=yp
```

Edit and save the C file and type `make -f Makefile.lib` which will compile the module and then create a shared library. Run XPP using the ode file `tstdll.ode`. Now click on `File Edit` and choose the load dynamic library option. For the library name, use the full path, unless you have put the library in `usr/lib`. Or better yet, before you run XPP, type `export LD_LIBRARY_PATH=.` or `setenv LD_LIBRARY_PATH=.`, depending on your shell and then XPP will look in the current directory for the library. Then for the function, choose one of `lv, vdp, duff` which are the three right-hand sides define above. The main advantage of dynamically linked libraries is that if you have a right-hand side that is three pages of computer output, then you can still use XPP with no problems.

12.2 Completely defining the right-hand sides in C

This is probably not something you want to do very often. It is better to use the above approach. This method forces XPP to be a stand-alone problem for a single ODE. For very complicated models such as discretizations of a PDE you may want to compile the right-hand sides and run a dedicated program for

that particular model. This is not as hard as it seems. You must first create a library called `libxpp.a` which is done by typing `make xpplib` after you have successfully compiled all of XPP. You only need to make this library once. You can then move it to where you usually keep libraries if you want. Now all you do is create a C file for the right-hand sides (I will show you below), say, it is called `lorenzrhs.c` and then type:

```
gcc lorenzrhs.c -o lorenz libxpp.a -lm -lX11
```

and if all goes well, you will have an executable called "lorenz". If the ODE file was called "lorenz.ode" then run this as follows:

```
lorenz lorenz.ode
```

as XPP needs the information contained in the ODE file to tell it the names of variables and parameters, etc.

Since the names of the parameters are interpreted by XPP as references to a certain array, you must communicate their values to the C functions for your right-hand sides. XPP provides a little utility for this. In the (Files) submenu. click on (c-Hints) and a bunch of defines will be pronted to the console. You can use these in your program. All of your c-files must have the following skeleton:

```
#include <math.h>

extern double constants[];
main(argc,argv)
 char **argv;
 int argc;
{
 do_main(argc,argv);
 }



my_rhs(t,y,ydot,neq)
 double t,*y,*ydot;
 int neq;
{

}

extra(y,t,nod,neq)
 double t,*y;
 int nod,neq;
{

}
```

In addition, you must define the parameters for the problem. Clicking on the (c-Hints) will essentially write this skeleton along with the defines for the problem. Lets take the lorenz equation as an example. Here is the ODE file:

```
# the famous Lorenz equation set up for 3d view
init x=-7.5  y=-3.6  z=30
par r=27  s=10  b=2.66666
x'=s*(-x+y)
y'=r*x-y-x*z
z'=-b*z+x*y
@ dt=.025, total=40, xplot=x,yplot=y,zplot=z,axes=3d
@ xmin=-20,xmax=20,ymin=-30,ymax=30,zmin=0,zmax=50
@ xlo=-1.5,ylo=-2,xhi=1.5,yhi=2
done
```

Run XPP with this file as the input file and click on the (File) (c-Hints) and the following will be written to the console:

```
#include <math.h>

 extern double constants[];
main(argc,argv)
 char **argv;
 int argc;
{
 do_main(argc,argv);
 }
/* defines for lorenz.ode  */
#define r constants[2]
#define s constants[3]
#define b constants[4]
#define X y[0]
#define XDOT ydot[0]
#define Y y[1]
#define YDOT ydot[1]
#define Z y[2]
#define ZDOT ydot[2]
my_rhs(t,y,ydot,neq)
 double t,*y,*ydot;
 int neq;
{
  }
extra(y,t,nod,neq)
 double t,*y;
 int nod,neq;
{
  }
```

You just fill in the blanks as follows to produce the required C file:

```
#include <math.h>

extern double constants[];

main(argc,argv)
     char **argv;
     int argc;
{
  do_main(argc,argv);
}

/* defines for lorenz.ode  */
#define r constants[2]
#define s constants[3]
#define b constants[4]
#define X y[0]
#define XDOT ydot[0]
#define Y y[1]
#define YDOT ydot[1]
#define Z y[2]
#define ZDOT ydot[2]

extra(y, t,nod,neq)
     double *y,t;
     int nod,neq;
{
 return;
}

my_rhs( t,y,ydot,neq)
 double t,*y,*ydot;
 int neq;
{
 XDOT=s*(-X+Y);
YDOT=r*X-Y-X*Z;
ZDOT=-b*Z+X*Y;

}
```

Now just compile this and link it with the XPP library and run it with "lorenz.ode" as the input and it will be a dedicated solver of the lorenz equations.

Here is a final example that shows you how to include user-defined functions and auxiliary variables. Here is the ODE file:

```
# The Morris-Lecar model as in our chapter in Koch & Segev
#  A simple membrane oscillator.
#
params v1=-.01,v2=0.15,v3=0.1,v4=0.145,gca=1.33,phi=.333
params vk=-.7,vl=-.5,iapp=.08,gk=2.0,gl=.5,om=1
minf(v)=.5*(1+tanh((v-v1)/v2))
ninf(v)=.5*(1+tanh((v-v3)/v4))
lamn(v)= phi*cosh((v-v3)/(2*v4))
ica=gca*minf(v)*(v-1)
v'=  (iapp+gl*(vl-v)+gk*w*(vk-v)-ica)*om
w'= (lamn(v)*(ninf(v)-w))*om
aux I_ca=ica
b v-v'
b w-w'
@ TOTAL=30,DT=.05,xlo=-.6,xhi=.5,ylo=-.25,yhi=.75
@ xplot=v,yplot=w
set vvst {xplot=t,yplot=v,xlo=0,xhi=100,ylo=-.6,yhi=.5,total=100 \
dt=.5,meth=qualrk}
done
```

and here is the C-file you need:

```
#include <math.h>

extern double constants[];

main(argc,argv)
     char **argv;
     int argc;
{
  do_main(argc,argv);
}


/* defines for lecar.ode  */
#define v1 constants[2]
#define v2 constants[3]
#define v3 constants[4]
#define v4 constants[5]
#define gca constants[6]
#define phi constants[7]
#define vk constants[8]
#define vl constants[9]
#define iapp constants[10]
#define gk constants[11]
#define gl constants[12]
```

```
#define om constants[13]
#define V y[0]
#define VDOT ydot[0]
#define W y[1]
#define WDOT ydot[1]
#define I_CA y[2]



double minf(v)
     double v;
{
  return .5*(1+tanh((v-v1)/v2));
}

double ninf(v)
     double v;
{
  return .5*(1+tanh((v-v3)/v4));
}

double lamn(v)
     double v;
{
  return phi*cosh((v-v3)/(2*v4));
}


extra(y, t,nod,neq)
     double *y,t;
     int nod,neq;
{
  I_CA=gca*minf(V)*(V-1.0);
}

my_rhs( t,y,ydot,neq)
 double t,*y,*ydot;
 int neq;
{
VDOT=om*(iapp+gl*(vl-V)+gk*W*(vk-V)+gca*minf(V)*(1-V));
WDOT=om*lamn(V)*(ninf(V)-W);
}
```

Note that the user-defined functions appear as C functions and the auxiliary variables are almost like regular ones and are defined in the “extra” function.

13 Some comments on the numerical methods

Most are standard.

1. The BVP solve works by shooting and using Newton's method. All Jacobi matrices are computed numerically.

2. The nullclines are found by dividing the window into a grid and evaluating the vector field at each point. Zero contours are found and plotted.

3. Equilibria are found with Newton's method and eigenvalues are found by the QR algorithm. Invariant sets are found by using initial data along an eigenvector for the corresponding eigenvalue. The eigenvector is computed by inverse iteration.

4. The Gear algorithm is out of Gear's text on numerical methods.

5. The two adaptive algorithms qualrk4 and stiff are from Numerical Recipes.

6. CVODE is based on a C-version of LSODE. It was written by Scott D. Cohen and Alan C. Hindmarsh, Numerical Mathematics Group, Center for Computational Sciences and Engineering, L-316, Lawrence Livermore National Lab, Livermore, CA 94551. email: alanh@llnl.gov. You can get full documentation for this powerful package `http://netlib.bell-labs.com/netlib/ode/index.html`.

7. Dormand/Prince are from their book

8. Rosenbrock is based on a Matlab version of the two step Rosenbrock algorithm (see Numerical Recipes again)

9. Delay equations are solved by storing previous data and quadratically interpolating from this data.

10. Stability of delay equations is computed by a method suggested by Tatyana Luzyanina. The linearized stability for a delay equation results in solving a transcendental equation $f(z) = 0$. The idea is to use the argument principle and compute the total change in the argument of $f(z)$ as z goes around a a contour C. The number of times divided by 2π tells us the number of roots of f inside the contour. Thus, XPP simply adds values of the argument of $f(z)$ at discrete points on a large contour defined by the user and which encloses a big chunk of the right-half plane. Obviously the best it can do is give sufficient conditions for instability as there could always be roots outside the contour. But it seems to work pretty well with modest contours except near changes in stability. In addition, XPP tries to find a specific eigenvalue by using Newton's method on the characteristic equation. Since there are infinitely many possible roots to these transcendental equations, the root found can be arbitrary. However, suppose there is a single pair of roots in the right-half plane. Then guessing a positive root will often land you on the desired root. Using the Singular

Point Range option will follow this particular root as a parameter varies. This can often lead to a discovery of the value of the parameter for which there is a Hopf bifurcation.

11. Adjoints are computed for stable periodic orbits by integrating the negative transpose of the numerically computed variational equation backwards in time using backward Euler.

12. The Volterra solver uses essentially an integrator (second order) based on the implicit product scheme described in Peter Linz's book on Volterra equations (SIAM,1985). For ODEs implicit schemes take considerably more time than explicit ones, but since most of the compute time for Volterra equations is in approximating the integral, this time penalty is minimal. Performance is gained primarily by taking advantage of convolution type equations.

13. Normally distributed noise is computed by the Box-Muller transformation of uniform noise.

14. The curve-fitting is done by using a heavily customized version of the Marquardt-Levenberg algorithm taken from Numerical Recipes in C.

15. The FFT is through the usual means

16. The algorithms in the bifurcation package are described in the AUTO manual available from Eusebius Doedel. I just wrote the interface.

17. DAEs of the form $F(X', X, W, t) = 0$ are solved by fixing X and using Newton's method to compute (X', W). The value of X' is sent to the integrator to update X. This apparently is not how DASSL does it. It is essentially the method used by Rheinboldt et al in their package MANPAK. (I must confess that I invented my own way to solve them out of the inability to get DASSL to work.)

14 Colors

The colors used in individual curves take on numbers from 0 to 10. Here is the meaning of the numbers:

0-Black/White; 1-Red; 2-Red Orange; 3-Orange; 4-Yellow Orange; 5-Yellow; 6-Yellow Green; 7-Green; 8-Blue Green; 9-Blue; 10-Purple.

15 The options file

You can have many options files. They are useful for initializing XPP. However, because you can now set options from within the ODE file, options files are probably obsolete. They have the following format:

```
9x15    BIG_FONT_NAME   <-- menu fonts
fixed   SMALL_FONT_NAME <-- IC, browser, etc fonts
0       BACKGROUND (1=white,0=black)
0 IXPLT <--  X-axis variable (0=time)
1 IYPLT <--  Y-axis variable
1 IZPLT <--  Z-axis variable
0 AXES <-- type of axis (0-2d 5-3d)
1 NJMP <-- nOutput
40 NMESH <-- Nullcline mesh
4 METHOD <-- Integration method
1 TIMEPLOT <--- set to zero if one axis is not time
8000 MAXSTOR <--- maximum rows stored
20.0 TEND <-- total integration time
.05 DT <--- time step
0.0 T0 <-- start time
0.0 TRANS <-- transient
100. BOUND <--- bounds
.0001 HMIN <-- min step for GEAR
1.0 HMAX <-- max ‘‘   ‘‘   ‘‘
.00001 TOLER <-- tolerance for GEAR
0.0 DELAY <-- maximal delay
0.0 XLO  <--- 2D window sizes
20.0 XHI
-2.0 YLO
2.0 YHI
```

Within an ODE file, you can call up a different options file by typing

```
option <filename>
```

where `<filename>` is the name of an options file.
